# The global burden of neonatal sepsis attributable to air pollution from 1990 to 2021: findings from the global burden of disease study 2021

**DOI:** 10.3389/fpubh.2025.1644191

**Published:** 2025-09-24

**Authors:** Jiajia Duan, Ying Yu, Zhifeng Qu, Hong Fu, Tao Jiang, Chuanxin Liu, Xiaoyang Bai, Min Wang, Hongxia Hu, Ruyan Chen, Dongxia Liu, Hetao Chen, Qiang Liu, Qizhi Fu

**Affiliations:** ^1^Luoyang Key Laboratory of Transplantation and Immunological Studies for Haematological Diseases, Department of Clinical Laboratory, The First Affiliated Hospital, and College of Clinical Medicine of Henan University of Science and Technology, Luoyang, China; ^2^Department of Laboratory Medicine, First Affiliated Hospital of Chongqing Medical University, Chongqing, China; ^3^Department of Medical Equipment, The First Affiliated Hospital, and College of Clinical Medicine of Henan University of Science and Technology, Luoyang, China; ^4^Department of Rehabilitation, The Third People's Provincial Hospital of Henan Province, Zhengzhou, China; ^5^Henan Key Laboratory of Rare Diseases, Endocrinology and Metabolism Center, The First Affiliated Hospital, and College of Clinical Medicine of Henan University of Science and Technology, Luoyang, China; ^6^Department of Respiratory and Critical Care Medicine, The First Affiliated Hospital, and College of Clinical Medicine of Henan University of Science and Technology, Luoyang, China; ^7^Department of Intensive Care Unit (Internal Medicine), The First Affiliated Hospital, and College of Clinical Medicine of Henan University of Science and Technology, Luoyang, China

**Keywords:** neonatal sepsis, neonatal infections, air pollution, particulate matter pollution, GBD

## Abstract

**Background:**

Neonatal sepsis remains a major disease threatening the lives of newborns. With the escalating global air pollution, substantial evidence indicates that air pollution is among the primary environmental threats to children's health. However, its contribution to the global burden of neonatal sepsis and other neonatal infections remains unclear. Although existing studies have established associations between air pollution and adverse neonatal outcomes, a comprehensive evaluation differentiating pollution types and accounting for socio-economic disparities across geographic regions remains lacking. This study fills this critical evidence gap.

**Methods:**

Based on data from the Global Burden of Disease (GBD) Study 2021, we analyzed associations between air pollution, particulate matter pollution, household air pollution from solid fuels, and ambient particulate matter pollution and neonatal sepsis and other neonatal infections, calculating deaths, disability-adjusted life years (DALYs), and their corresponding age-standardized rates (ASRs). Subsequently, cluster analysis and decomposition analysis were conducted to identify regional patterns and quantify contributing factors. Finally, an autoregressive integrated moving average (ARIMA) model was employed to forecast the disease burden from 2022 to 2050.

**Result:**

In 2021, global deaths from neonatal sepsis and related infections attributable to air pollution numbered 54,026 (95% UI: 45,371–64,084), a 23.48% decrease from 1990, with age-standardized death rates dropping 1.49% annually (EAPC = −1.49). Deaths from household solid fuel pollution fell by 30.65%, while ambient particulate matter pollution caused a 13.05% increase to 13,080 deaths. Low-SDI regions bore the highest death burden with 31,063 cases, and Western Africa showed the highest age-standardized mortality rate of 2.21. African countries like Sierra Leone ranked top globally. Male deaths and DALYs consistently exceeded female figures. Population growth was the primary driver of global burden increase, contributing 621.99% to deaths, mitigated by epidemiological improvements. Projections indicate continuous declines in air/household pollution-related deaths 2022–2050, steeper in females, while ambient particulate matter deaths may peak in 2027 before easing.

**Conclusion:**

Overall, air pollution remains a significant public health challenge threatening neonatal health. Implementing targeted, geographically tailored interventions is essential to reduce disease burden resulting from air pollution.

## 1 Introduction

Neonatal and under-five mortality rates are among the core indicators reflecting child health status, healthcare quality, and social development within countries and regions (1). Despite significant global progress in reducing child mortality over recent decades, the mortality rate among children under five remains alarmingly high. According to 2022 data, ~4.9 million children under the age of five died worldwide (2), with neonatal deaths (occurring within the first 28 days of life) accounting for 47% of this total (1), and this proportion continues to rise.

Neonatal infections, preterm births, and birth complications (including birth asphyxia and trauma) collectively contribute to nearly 90% of neonatal deaths (3, 4). Neonatal sepsis, defined as a systemic inflammatory response syndrome caused by infections (bacterial, viral, or fungal) during the neonatal period (typically within the first 28 days postpartum), is classified as either early-onset or late-onset based on a 72-h threshold post-birth (5). Neonatal sepsis affects ~3 million newborns annually, with a mortality rate as high as 17% (6), making it a significant cause of neonatal death and a considerable global health challenge. Neonatal infections account for ~26% of deaths among children under five (7). Additionally, neonatal sepsis incidence is notably higher in low- and middle-income countries compared to high-income nation (8), with studies identifying Sub-Saharan Africa as having one of the highest neonatal sepsis mortality rates globally (9, 10). Existing research data indicate that sub-Saharan Africa experiences an estimated 380,000 to 2,000,000 cases of neonatal sepsis and 270,000 associated deaths annually (11). Despite this, current prevention strategies exhibit substantial limitations.

Air pollution represents one of the most significant environmental risks to child health, encompassing particulate matter pollution, which can be further categorized into ambient particulate matter pollution and household air pollution from solid fuels (12). According to the Global Air Quality Report 2021, air pollution was associated with more than 700,000 deaths among children under 5 years old globally, making it the second leading global risk factor for mortality within this age group (13). Numerous studies have also demonstrated that exposure to PM_2.5_ during pregnancy is associated with adverse effects on neonatal health, leading to negative outcomes (14–16). Recently, a time stratified, case crossover analyses conducted in the United States found that short-term exposure to PM_2.5_ is associated with increased risk of hospital admissions due to septicaemia (17). Alarmingly, household air pollution due to cooking with polluting fuels accounted for ~500,000 of these deaths (18). Furthermore, research has linked exposure to ambient air pollution, household air pollution, and particulate matter with adverse birth outcomes such as low birth weight, preterm birth, and respiratory infection (19–21). Prenatal air pollution exposure has also been associated with an increased risk of neonatal sepsis (22). Recent studies further indicate a positive correlation between long-term air pollution exposure and sepsis-related mortality in older adults populations (23). However, the direct and indirect roles of these pollutants in neonatal sepsis and other neonatal infections have not been comprehensively examined on a global scale. As climate change and urbanization continue to exacerbate pollution levels, understanding the long-term trends in pollution-associated neonatal mortality is critical for developing effective public health interventions (24).

Therefore, we utilized the Global Burden of Disease (GBD) database to evaluate trends and burdens of neonatal sepsis and other neonatal infections attributable to air pollution, particulate matter pollution, household air pollution from solid fuels, and ambient particulate matter pollution from 1990 to 2021, and forecasted disease burdens from 2022 to 2050. This study aims to inform healthcare professionals and policymakers, facilitating the development of targeted public health strategies to reduce the substantial disease burden caused by air pollution.

## 2 Methods

### 2.1 Overview

This study utilized the latest data from the GBD Study 2021 database (25), analyzing the burden of neonatal sepsis and other neonatal infections (age < 5 years) attributable to air pollution, particulate matter pollution, household air pollution from solid fuels, and ambient particulate matter pollution at global, regional, and national levels. Developed by the Institute for Health Metrics and Evaluation (IHME) in the United States, the GBD database integrates data from 204 countries and territories, covering 371 diseases and injuries with indicators such as prevalence, incidence, deaths, disability-adjusted life years (DALYs), and age-standardized rates (ASRs), stratified by age, gender, geographic region, and socio-demographic index (SDI).

GBD classifies countries and regions into 21 regions and seven super-regions based on geographic location. SDI is a composite measure used to assess socio-economic development, typically encompassing factors such as income, education, and fertility rates. The SDI facilitates the analysis of regional health burdens and helps identify underlying causes of health inequalities (26). Based on SDI, countries and regions are categorized into five groups: high SDI, high-middle SDI, middle SDI, low-middle SDI, and low SDI.

### 2.2 Data acquisition and preliminary analysis

We extracted deaths, DALYs, and their corresponding ASRs for neonatal sepsis and other neonatal infections attributable to air pollution, particulate matter pollution, household air pollution from solid fuels, and ambient particulate matter pollution from the GBD database for the period 1990 to 2021. Disease burden was modeled and estimated by gender, region, and year using the Disease Modeling Meta-Regression (DisMod-MR) tool to ensure data consistency and accuracy. DisMod-MR is a Bayesian statistical modeling and meta-regression tool specifically used for estimating epidemiological parameters in burden-of-disease studies. Subsequently, bias adjustment was performed using MR-BRT.

### 2.3 Statistics

Our study reported global and subgroup-specific data (SDI, region, and country) on deaths, DALYs, and ASRs attributable to neonatal sepsis and other neonatal infections caused by air pollution, particulate matter pollution, household air pollution from solid fuels, and ambient particulate matter pollution in 2021. The estimated annual percentage change (EAPC) was utilized to evaluate temporal trends in disease burden globally and across various subgroups from 1990 to 2021. Furthermore, cluster analyses based on EAPC values were conducted to characterize patterns in disease burden changes across GBD regions and to identify regions exhibiting similar trends. To further investigate the underlying drivers of changes in DALYs and deaths related to neonatal sepsis and other neonatal infections between 1990 and 2021, decomposition analysis was performed, quantifying the contributions of population dynamics and epidemiological shifts (27). Additionally, autoregressive integrated moving average (ARIMA) models, a class of statistical models used for time series analysis and forecasting, were applied to predict gender-specific deaths and DALYs attributable to neonatal sepsis and other neonatal infections associated with air pollution, particulate matter pollution, household air pollution from solid fuels, and ambient particulate matter pollution from 2022 to 2050, thereby providing a scientific basis for future public health interventions. Finally, we conducted a stability analysis, selected the model combination with the smallest AIC/BIC values, and verified its applicability. The white noise test (α = 0.05) was used to evaluate the model residuals. The prediction performance was assessed using root mean square error (RMSE), mean absolute error (MAE), and mean absolute percentage error (MAPE).

## 3 Results

### 3.1 Global epidemiological patterns of air pollution-attributable neonatal sepsis (1990–2021)

In 2021, at the national level, deaths attributable to neonatal sepsis and other neonatal infections caused by air pollution totaled 54,026 (95% UI: 45,371–64,084), representing a decrease of 23.48% from 1990 to 2021. Correspondingly, the age-standardized deaths rate exhibited a declining trend, with an average annual reduction of 1.49% (EAPC = −1.49; 95% *CI*: −1.65to −1.34) during this period (Table 1, Supplementary Figure S1). Deaths due to neonatal sepsis and other neonatal infections associated with particulate matter pollution numbered 54,026 (95% UI: 45,371–64,084), also declining by 23.48% from 1990 to 2021, and the age-standardized deaths rate decreased annually by 1.49% (EAPC = −1.49; 95% *CI*: −1.65 to −1.34; Table 1, Supplementary Figure S4B). Deaths attributed to household air pollution from solid fuels accounted for 40,937 cases (95% UI: 32,377–51,168), reflecting a 30.65% reduction between 1990 and 2021, with an annual decline in the age-standardized deaths rate of 1.92% (EAPC = −1.92; 95% *CI*: −2.09 to −1.74; Table 1, Supplementary Figure S4C). Conversely, deaths due to neonatal sepsis and other neonatal infections associated with ambient particulate matter pollution increased to 13,080 (95% UI: 7,635–20,048), representing a 13.05% rise from 1990 to 2021, accompanied by an upward trend in the age-standardized deaths rate (EAPC = 0.15; 95% *CI*: −0.05 to 0.36; Table 1, Supplementary Figure S4D).

**Table 1:** Deaths of neonatal sepsis and other neonatal infections in 1990 and 2021 by different characteristics.

**Characteristics**	**No. of deaths**				
	**1990**		**2021**		**1990-2021**
	**Number of deaths cases (95% UI)**	**Age-standardized rate per 100,000 population (95% UI)**	**Number of deaths cases (95% UI)**	**Age-standardized rate per 100,000 population (95% UI)**	**EAPC (95%CI)**
**Air pollution**					
Global	70,607 (611,46–81,144)	1.1 (0.96–1.27)	54,026 (45,371–64,084)	0.87 (0.73–1.04)	−1.49 (−1.65 to −1.34)
**Sex**					
Both	70,607 (61,146–81,144)	1.1 (0.96–1.27)	54,026 (45,371–64,084)	0.87 (0.73–1.04)	−0.96 (−1.09 to −0.84)
Female	28,923 (24,656–32,916)	0.94 (0.8–1.07)	22,192 (18,454–25,969)	0.74 (0.62–0.87)	−0.93 (−1.06 to −0.8)
Male	41,684 (34,940–50,016)	1.26 (1.06–1.51)	31,834 (25,703–38,791)	0.99 (0.8–1.21)	−0.99 (−1.1 to −0.87)
**Age**					
< 5 years	70,607 (61,146–81,144)	11.39 (9.86–13.09)	54,026 (45,371–64,084)	8.21 (6.89–9.74)	−1.16 (−1.33 to −0.98)
**Countries categorized by SDI**					
High-middle SDI	1,367 (1,122–1,645)	0.16 (0.13–0.19)	307 (242–380)	0.05 (0.04–0.07)	−3.8 (−4 to −3.61)
High SDI	261 (202–329)	0.04 (0.03–0.05)	63 (50–80)	0.01 (0.01–0.02)	−4.12 (−4.4 to −3.85)
Low-middle SDI	31,428 (26,197–37,324)	1.7 (1.42–2.02)	17,845 (14,328–21,799)	0.96 (0.77–1.17)	−1.96 (−2.04 to −1.87)
Low SDI	26,546 (22,718–30,833)	2.52 (2.15–2.92)	31,063 (25,026–38,875)	1.8 (1.45–2.25)	−0.95 (−1.01 to −0.89)
Middle SDI	10,963 (9,126–13,052)	0.55 (0.46–0.65)	4,711 (3,841–5,782)	0.31 (0.25–0.38)	−2.11 (−2.35 to −1.87)
**Particulate matter pollution**					
Global	70,607 (61,146–81,144)	1.1 (0.96–1.27)	54,026 (45,371–64,084)	0.87 (0.73–1.04)	−1.49 (−1.65 to −1.34)
**Sex**					
Both	70,607 (61,146–81,144)	1.1 (0.96–1.27)	54,026 (45,371–64,084)	0.87 (0.73–1.04)	−0.96 (−1.09 to −0.84)
Female	28,923 (24,656–32,916)	0.94 (0.8–1.07)	22,192 (18,454–25,969)	0.74 (0.62–0.87)	−0.93 (−1.06 to −0.8)
Male	41,684 (34,940–50,016)	1.26 (1.06–1.51)	31,834 (25,703–38,791)	0.99 (0.8–1.21)	−0.99 (−1.1 to −0.87)
**Age**					
< 5 years	70,607 (61,146–81,144)	11.39 (9.86–13.09)	54,026 (45,371–64,084)	8.21 (6.89–9.74)	−1.16 (−1.33 to −0.98)
**Countries categorized by SDI**					
High-middle SDI	1,367 (1,122–1,645)	0.16 (0.13–0.19)	307 (242–380)	0.05 (0.04–0.07)	−3.8 (−4 to −3.61)
High SDI	261 (202–329)	0.04 (0.03–0.05)	63 (50–80)	0.01 (0.01–0.02)	−4.12 (−4.4 to −3.85)
Low-middle SDI	31,428 (26,197–37,324)	1.7 (1.42–2.02)	17,845 (14,328–21,799)	0.96 (0.77–1.17)	−1.96 (−2.04 to −1.87)
Low SDI	26,546 (22,718–30,833)	2.52 (2.15–2.92)	31,063 (25,026–38,875)	1.8 (1.45–2.25)	−0.95 (−1.01 to −0.89)
Middle SDI	10,963 (9,126–13,052)	0.55 (0.46–0.65)	4,711 (3,841–5,782)	0.31 (0.25–0.38)	−2.11 (−2.35 to −1.87)
**Household air pollution from solid fuels**					
Global	59,029 (50,637–68,922)	0.92 (0.79–1.08)	40,937 (32,377–51,168)	0.66 (0.52–0.83)	−1.92 (−2.09 to −1.74)
**Sex**					
Both	59,029 (50,637–68,922)	0.92 (0.79–1.08)	40,937 (32,377–51,168)	0.66 (0.52–0.83)	−1.39 (−1.56 to −1.21)
Female	24,210 (20,368–28,271)	0.79 (0.66–0.92)	16,730 (13,309–20,874)	0.56 (0.45–0.7)	−1.37 (−1.56 to −1.19)
Male	34,819 (28,574–42,267)	1.05 (0.86–1.28)	24,207 (18,427–31,418)	0.76 (0.58–0.98)	−1.39 (−1.56 to −1.22)
**Age**					
< 5 years	59,029 (50,637–68,922)	9.52 (8.17–11.12)	40,937 (32,377–51,168)	6.22 (4.92–7.77)	−1.58 (−1.8 to −1.35)
**Countries categorized by SDI**					
High-middle SDI	576 (366–873)	0.07 (0.04–0.1)	10 (1–55)	0 (0–0.01)	−12.16 (−12.9 to −11.41)
High SDI	23 (8–56)	0 (0–0.01)	0 (0–1)	0 (0–0)	−16.91 (−17.47 to −16.36)
Low-middle SDI	27,253 (22,525–33,061)	1.47 (1.22–1.79)	12,872 (9,442–16,713)	0.69 (0.51–0.9)	−2.62 (−2.78 to −2.46)
Low SDI	23,415 (20,075–27,117)	2.22 (1.9–2.57)	26,378 (20,833–32,927)	1.53 (1.21–1.91)	−1.17 (−1.25 to −1.1)
Middle SDI	7,728 (6,026–9,718)	0.39 (0.3–0.49)	1,647 (868–2,885)	0.11 (0.06–0.19)	−4.44 (−4.93 to −3.96)
**Ambient particulate matter pollution**					
Global	11,570 (7,498–16,496)	0.18 (0.12–0.26)	13,080 (7,635–20,048)	0.21 (0.12–0.32)	0.15 (-0.05 to 0.36)
**Sex**					
Both	11,570 (7,498–16,496)	0.18 (0.12–0.26)	13,080 (7,635–20,048)	0.21 (0.12–0.32)	0.69 (0.48–0.91)
Female	4,710 (3,043–6,928)	0.15 (0.1–0.22)	5,459 (3,251–8,458)	0.18 (0.11–0.28)	0.81 (0.58–1.03)
Male	6,860 (4,514–9,886)	0.21 (0.14–0.3)	7,621 (4,452–11,775)	0.24 (0.14–0.37)	0.62 (0.41–0.82)
**Age**					
< 5 years	11,570 (7,498–16,496)	1.87 (1.21–2.66)	13,080 (7,635–20,048)	1.99 (1.16–3.05)	0.5 (0.27–0.72)
**Countries categorized by SDI**					
High-middle SDI	790 (501–1,051)	0.09 (0.06–0.12)	297 (225–376)	0.05 (0.04–0.07)	−2.07 (−2.33 to −1.82)
High SDI	238 (179–306)	0.04 (0.03–0.05)	63 (50–80)	0.01 (0.01–0.02)	−3.86 (−4.17 to −3.55)
Low-middle SDI	4,174 (2,586–6,523)	0.23 (0.14–0.35)	4,970 (2,557–8,211)	0.27 (0.14–0.44)	0.82 (0.45–1.19)
Low SDI	3,129 (2,077–4,530)	0.3 (0.2–0.43)	4,681 (2,790–7,638)	0.27 (0.16–0.44)	0.47 (0.18–0.77)
Middle SDI	3,232 (1,833–4,798)	0.16 (0.09–0.24)	3,061 (1,837–4,202)	0.2 (0.12–0.27)	0.53 (0.32–0.75)

UI, uncertainty intervals; CI, confidence interval; SDI, socio-demographic index; EAPC, estimated annual percentage change.

In 2021, DALYs attributable to neonatal sepsis and other neonatal infections caused by air pollution were 4,861,948 (95% UI: 4,083,310–5,767,176), with an age-standardized DALYs rate of 78.57 per 100,000 people (95% UI: 65.98–93.19). From 1990 to 2021, the number of DALYs decreased by 23.49%, while the age-standardized DALYs rate declined by 20.96%, with an EAPC of −1.50 (95% *CI*: −1.65 to −1.34; Table 2, Supplementary Figure S4A). DALYs due to particulate matter pollution were also 4,861,948 (95% UI: 4,083,310–5,767,176), with the same corresponding age-standardized DALYs rate and EAPC values as air pollution (Table 2, Supplementary Figure S4B). For household air pollution from solid fuels, DALYs totaled 3,683,938 (95% UI: 2,913,626–4,604,472), and the age-standardized DALYs rate was 59.53 per 100,000 people (95% UI: 47.08–74.41), decreasing by 30.66% for DALY numbers and 28.36% for the age-standardized DALYs rate between 1990 and 2021 (EAPC = −1.92; 95% *CI*: −2.09 to −1.74; Table 2, Supplementary Figure S4C). Conversely, DALYs attributable to ambient particulate matter pollution rose to 1,177,162 (95% UI: 687,185–1,804,264), representing an increase of 13.04%, and the age-standardized DALYs rate increased by 15.76% to 19.02 per 100,000 people (95% UI: 11.1–29.16; EAPC = 0.15; 95% *CI*: −0.05 to 0.36; Table 2, Supplementary Figure S4D).

**Table 2:** DALYs of neonatal sepsis and other neonatal infections in 1990 and 2021 by different characteristics.

**Characteristics**	**DALYs**				
	**1990**		**2021**		**1990–2021**
	**Number of DALYs cases (95% UI)**	**The age-standardized DALYs rate/100,000 (95% UI)**	**Number of DALYs cases (95% UI)**	**The age-standardized DALYs rate/100,000 (95% UI)**	**EAPC (95%CI)**
**Air pollution**					
Global	6,354,500 (5,502,797–7,302,612)	99.4 (86.07–114.19)	4,861,948 (4,083,310–5,767,176)	78.57 (65.98–93.19)	−1.5 (−1.65 to −1.34)
**Sex**					
Both	6,354,500 (5,502,797–7,302,612)	99.4 (86.07–114.19)	4,861,948 (4,083,310–5,767,176)	78.57 (65.98–93.19)	−0.96 (−1.09 to −0.84)
Female	2,602,952 (2,218,990–2,962,247)	84.44 (71.98–96.09)	1,997,088 (1,660,749–2,336,913)	66.81 (55.56–78.18)	−0.93 (−1.06 to −0.8)
Male	3,751,549 (3,145,253–4,501,026)	113.32 (94.99–136)	2,864,860 (2,313,427–3,490,758)	89.54 (72.3–109.1)	−0.99 (−1.1 to −0.87)
**Age**					
< 5 years	6,354,500 (5,502,797–7,302,612)	1025.02 (887.64–1177.96)	4,861,948 (4,083,310–5,767,176)	738.71 (620.4–876.24)	−1.16 (−1.33 to −0.98)
**Countries categorized by SDI**					
High-middle SDI	123,090 (101,095–148,124)	14 (11.5–16.85)	27,660 (21,842–34,238)	4.9 (3.87–6.07)	−3.8 (−4 to −3.61)
High SDI	23,548 (18,190–29,669)	3.91 (3.02–4.92)	5,722 (4,551–7,192)	1.15 (0.92–1.45)	−4.12 (−4.4 to −3.84)
Low-middle SDI	2,828,466 (2,357,734–3,359,088)	152.96 (127.44–181.54)	1,605,989 (128,9547–1,961,737)	86.11 (69.15–105.19)	−1.96 (−2.04 to −1.87)
Low SDI	2,388,995 (2,044,603–2,774,648)	226.36 (193.69–262.87)	2,795,358 (2,252,325–3,498,265)	162.04 (130.58–202.81)	−0.95 (−1.01 to −0.89)
Middle SDI	986,707 (821,329–1,174,574)	49.29 (41.03–58.67)	423,994 (345,796–520,377)	27.65 (22.55–33.94)	−2.11 (−2.35 to −1.87)
**Particulate matter pollution**					
Global	6354,500 (5,502,797–7,302,612)	99.4 (86.07–114.19)	4,861,948 (4,083,310–5,767,176)	78.57 (65.98–93.19)	−1.5 (−1.65 to −1.34)
**Sex**					
Both	6,354,500 (5,502,797–7,302,612)	99.4 (86.07–114.19)	4,861,948 (4,083,310–5,767,176)	78.57 (65.98–93.19)	−0.96 (−1.09 to −0.84)
Female	2,602,952 (2,218,990–2,962,247)	84.44 (71.98–96.09)	1,997,088 (1,660,749–2,336,913)	66.81 (55.56–78.18)	−0.93 (−1.06 to −0.8)
Male	3,751,549 (3,145,253–4,501,026)	113.32 (94.99–136)	2,864,860 (2,313,427–3,490,758)	89.54 (72.3–109.1)	−0.99 (−1.1 to −0.87)
**Age**					
< 5 years	6,354,500 (5,502,797–7,302,612)	1025.02 (887.64–1177.96)	4,861,948 (4,083,310–5,767,176)	738.71 (620.4–876.24)	−1.16 (−1.33 to −0.98)
**Countries categorized by SDI**					
High-middle SDI	123,090 (101,095–148,124)	14 (11.5–16.85)	27,660 (21,842–34,238)	4.9 (3.87–6.07)	−3.8 (−4 to −3.61)
High SDI	23,548 (18,190–29,669)	3.91 (3.02–4.92)	5,722 (4,551–7,192)	1.15 (0.92–1.45)	−4.12 (−4.4 to −3.84)
Low-middle SDI	2,828,466 (2,357,734–3,359,088)	152.96 (127.44–181.54)	1,60,5989 (1,289,547–1,961,737)	86.11 (69.15–105.19)	−1.96 (−2.04 to −1.87)
Low SDI	2,388,995 (2,044,603–2,774,648)	226.36 (193.69–262.87)	2,795,358 (2,252,325–3,498,265)	162.04 (130.58–202.81)	−0.95 (−1.01 to −0.89)
Middle SDI	986,707 (821,329–1,174,574)	49.29 (41.03–58.67)	423,994 (345,796–520,377)	27.65 (22.55–33.94)	−2.11 (−2.35 to −1.87)
Global	5,312,458 (4,557,442–6,202,777)	83.1 (71.28–97.02)	3,683,938 (2,913,626–4,604,472)	59.53 (47.08–74.41)	−1.92 (−2.09 to −1.74)
**Sex**					
Both	5,312,458 (4,557,442–6,202,777)	83.1 (71.28–97.02)	3,683,938 (2,913,626–4,604,472)	59.53 (47.08–74.41)	−1.39 (−1.56 to −1.21)
Female	2,178,805 (1,833,030–2,544,444)	70.68 (59.46–82.56)	1,505,501 (1,197,723–1,878,319)	50.37 (40.07–62.84)	−1.37 (−1.56 to −1.19)
Male	3,133,653 (2,571,578–3,803,592)	94.65 (77.66–114.93)	2,178,436 (1,658,316–2,827,342)	68.09 (51.83–88.37)	−1.39 (−1.56 to −1.22)
**Age**					
< 5 years	5,312,458 (4,557,442–6,202,777)	856.93 (735.14–1,000.55)	3,683,938 (2,913,626–4,604,472)	559.72 (442.68–699.59)	−1.58 (−1.8 to −1.35)
**Countries categorized by SDI**					
High-middle SDI	51,885 (32,982–78,642)	5.9 (3.75–8.95)	873 (93–4,926)	0.15 (0.02–0.87)	−12.16 (−12.9 to −11.41)
High SDI	2,078 (677–5,076)	0.34 (0.11–0.84)	10 (0–77)	0 (0–0.02)	−16.91 (−17.46 to −16.36)
Low-middle SDI	2,452,642 (2,027,062–2,975,288)	132.63 (109.62–160.74)	1,158,441 (849,748–1,504,076)	62.12 (45.56–80.65)	−2.62 (−2.78 to −2.46)
Low SDI	2,107,202 (1,806,626–2,440,180)	199.66 (171.17–231.39)	2,373,779 (1,874,828–2,962,955)	137.6 (108.69–171.75)	−1.18 (−1.25 to −1.1)
Middle SDI	695,529 (542,428–874,690)	34.74 (27.09–43.69)	148,273 (78,157–259,599)	9.67 (5.1–16.93)	−4.44 (−4.93 to −3.96)
**Ambient particulate matter pollution**					
Global	1,041,345 (674,951–1,484,893)	16.29 (10.56–23.23)	1,177,162 (687,185–180,4264)	19.02 (11.1–29.16)	0.15 (-0.05 to 0.36)
**Sex**					
Both	1,041,345 (674,951–1,484,893)	16.29 (10.56–23.23)	1,177,162 (687,185–180,4264)	19.02 (11.1–29.16)	0.69 (0.48–0.91)
Female	423,903 (273,896–623,437)	13.75 (8.89–20.23)	491,254 (292,631–761,062)	16.43 (9.79–25.46)	0.81 (0.58–1.03)
Male	617,442 (406,294–889,898)	18.65 (12.27–26.87)	685,907 (400,662–105,9664)	21.44 (12.52–33.12)	0.62 (0.41–0.82)
**Age**					
< 5 years	1,041,345 (674,951–1,484,893)	167.98 (108.87–239.52)	1,177,162 (687,185–180,4264)	178.85 (104.41–274.13)	0.5 (0.27–0.72)
**Countries categorized by SDI**					
High-middle SDI	71,156 (45,129–94,613)	8.09 (5.13–10.76)	26,778 (20,290–33,813)	4.74 (3.59–5.99)	−2.07 (−2.33-to 1.82)
High SDI	21,464 (16,117–27,563)	3.56 (2.67–4.57)	5,712 (4,515–7,187)	1.15 (0.91–1.45)	−3.86 (−4.17 to −3.55)
Low-middle SDI	375,640 (232,753–587,053)	20.32 (12.59–31.75)	447,264 (230,157–738,913)	23.98 (12.34–39.62)	0.82 (0.45–1.19)
Low SDI	281,597 (186,888–407,649)	26.68 (17.71–38.59)	421,219 (251,042–687,311)	24.42 (14.56–39.84)	0.47 (0.17–0.77)
Middle SDI	290,917 (164,995–431,899)	14.53 (8.24–21.57)	275,527 (165,328–378,224)	17.97 (10.78–24.67)	0.53 (0.32–0.75)

UI, uncertainty intervals; CI, confidence interval; SDI, socio-demographic index; EAPC, estimated annual percentage change.

### 3.2 Sex and socioeconomic disparities

**Figure 1:**
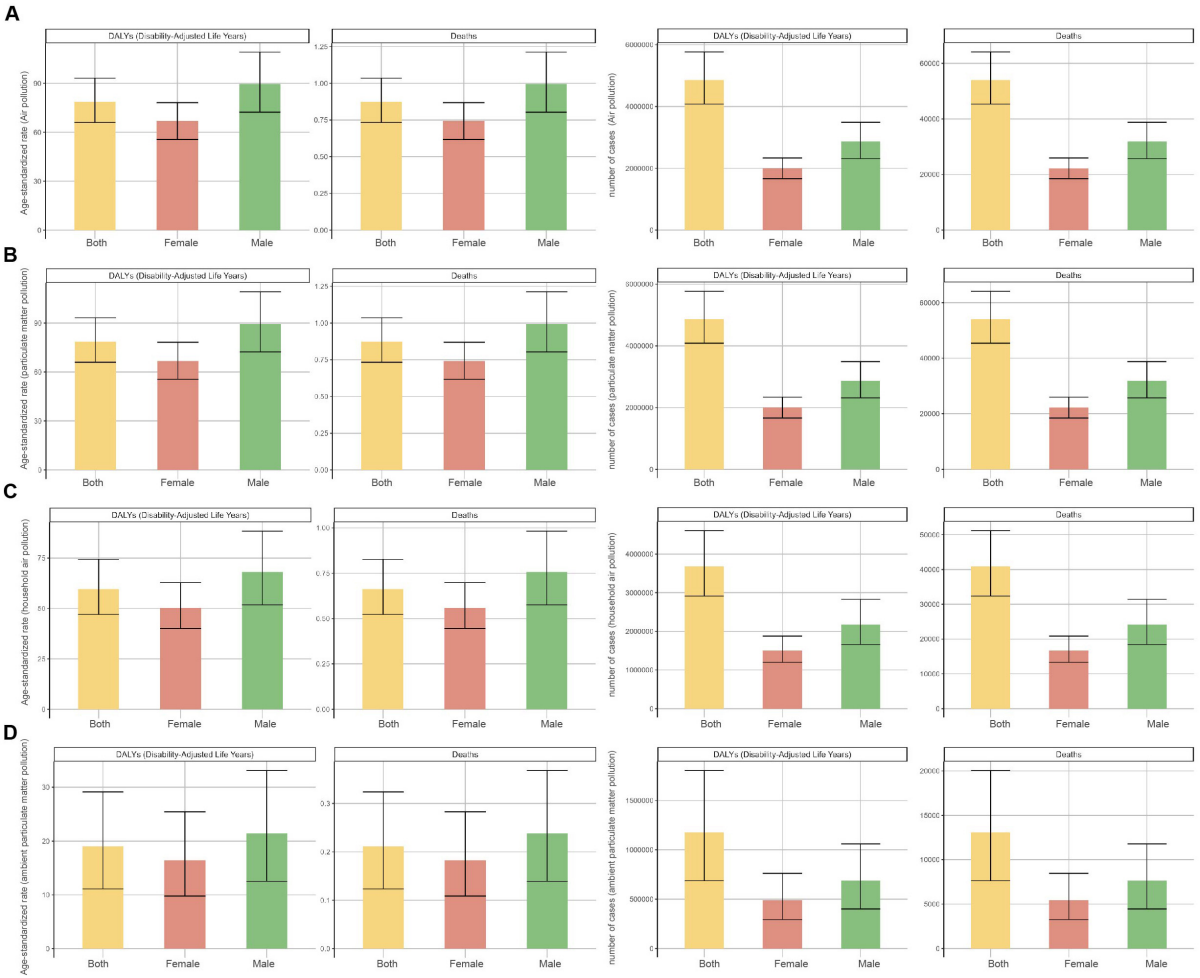
Numbers and age-standardized rates (ASR) of deaths and DALYs attributable to neonatal sepsis and other neonatal infections, by sex, in 2021. **(A)** Air pollution, **(B)** particulate matter pollution, **(C)** household air pollution, and **(D)** ambient particulate matter pollution.

In 2021, both deaths and DALYs attributable to neonatal sepsis and other neonatal infections caused by air pollution, particulate matter pollution, household air pollution from solid fuels, and ambient particulate matter pollution were consistently higher in males than females at the global level, with corresponding ASRs also higher in males (Tables 1, 2, Figure 1). Trends in deaths and DALYs from 1990 to 2021 were consistent across genders and aligned with the overall population trend (Supplementary Figure S2).

**Figure 2:**
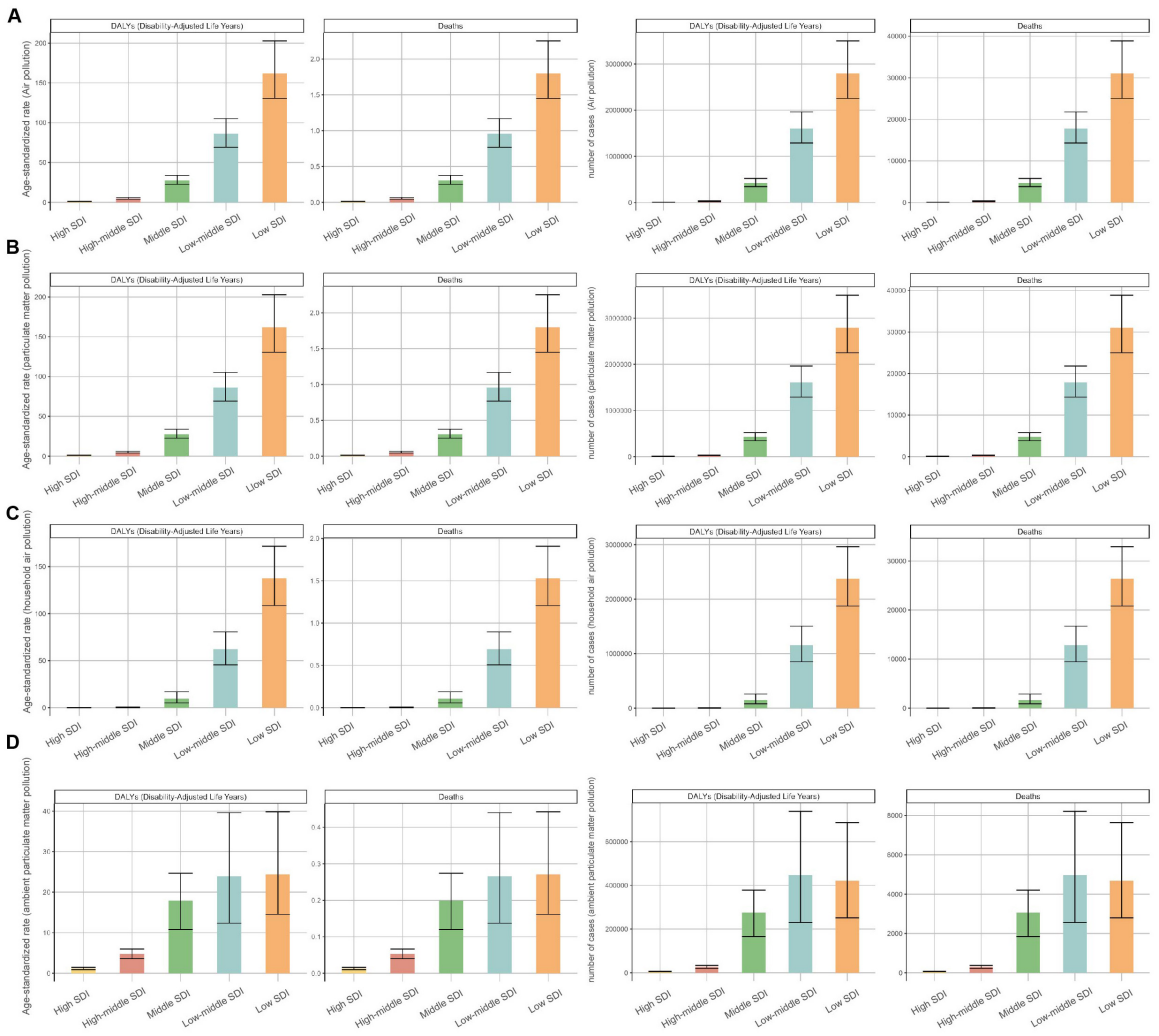
Numbers and age-standardized rates (ASR) of deaths and DALYs attributable to neonatal sepsis and other neonatal infections, by SDI, in 2021. **(A)** Air pollution, **(B)** particulate matter pollution, **(C)** household air pollution, and **(D)** ambient particulate matter pollution.

The global burden of neonatal sepsis and other neonatal infections from various air pollution sources showed notable regional disparities closely associated with SDI levels. In 2021, the low-SDI region had the highest number of deaths from air pollution [31,063 (95% UI: 25,026–38,875)], particulate matter pollution [31,063 (95% UI: 25,026–38,875)], and household air pollution from solid fuels [26,378 (95% UI: 20,833–32,927)] (Tables 1, 2, Figures 2A–C). Ambient particulate matter pollution resulted in the highest number of deaths [4,970 (95% UI: 2,557–8,211)] and age-standardized deaths rate in the low-middle SDI region (Tables 1, 2, Figure 2D). However, high-SDI regions exhibited significant reductions in EAPC for all four types of pollution: air pollution [−4.12 (95% *CI*: −4.4 to −3.85)], particulate matter pollution [−4.12 (95% *CI*: −4.4 to −3.85)], household air pollution from solid fuels [−16.91 (95% *CI*: −17.47 to −16.36)], and ambient particulate matter pollution [−3.86 (95% *CI*: −4.17 to −3.55)], reflecting effective prevention and management strategies. In contrast, the burden in low-middle SDI regions from ambient particulate matter pollution continued to rise significantly, with the highest EAPC of 0.82 (95% *CI*: 0.45–1.19; Tables 1, 2). Regional differences were further emphasized by age-standardized DALYs rate, consistent with death trends described above (Tables 1, 2). Analyzing trends from 1990 to 2021 revealed continuous declines in deaths and DALYs attributable to air pollution, particulate matter pollution, and household air pollution from solid fuels in middle SDI and low-middle SDI regions. High SDI and high-middle SDI regions showed stable trends, whereas low SDI regions initially increased and subsequently decreased (Supplementary Figure S3). Notably, significant intersection points occurred in 1998 between low SDI and low-middle SDI regions for air pollution and particulate matter pollution, and in 1997 for household air pollution from solid fuels. For ambient particulate matter pollution, deaths and DALYs gradually declined to stability in high SDI and high-middle SDI regions, whereas low SDI, low-middle SDI, and middle SDI regions experienced minor fluctuations between 2009 and 2010, followed by rapid increases reaching a peak in 2015 before decreasing. Trends between low SDI and low-middle SDI regions closely overlapped during this rising phase (Supplementary Figure S2D).

### 3.3 Regional hotspots and cluster analysis

Our results indicated that Western Africa experienced the highest global burden of neonatal sepsis and other neonatal infections attributable to air pollution and particulate matter pollution, with an age-standardized deaths rate (air pollution/particulate matter pollution: 2.21, 95% UI: 1.82–2.69) and an age-standardized DALYs rate (air pollution/particulate matter pollution: 199.15, 95% UI: 163.93–241.80). Western Sub-Saharan Africa ranked second (deaths: 2.18, 95% UI: 1.79–2.64; DALYs: 196.06, 95% UI: 160.90–237.16), followed by Eastern Sub-Saharan Africa (deaths: 2.06, 95% UI: 1.55–2.60; DALYs: 184.99, 95% UI: 139.63–233.57; Supplementary Tables S1, S2). Regarding neonatal sepsis and other neonatal infections associated with household air pollution from solid fuels, Eastern Sub-Saharan Africa exhibited the highest age-standardized deaths rate (1.90, 95% UI: 1.42–2.41) and DALYs rate (170.86, 95% UI: 127.88–216.68). Eastern Africa ranked second (deaths: 1.71, 95% UI: 1.27–2.18; DALYs: 153.57, 95% UI: 114.22–196.62), followed by the Commonwealth Low-Income region (deaths: 1.67, 95% UI: 1.33–2.15; DALYs: 150.33, 95% UI: 119.40–193.59; Supplementary Tables S1, S2). For ambient particulate matter pollution, the regions with the highest age-standardized deaths and DALYs rate due to neonatal sepsis and other neonatal infections were Western Africa (deaths: 0.55, 95% UI: 0.28–0.95; DALYs: 49.76, 95% UI: 24.98–85.52), Western Sub-Saharan Africa (deaths: 0.53, 95% UI: 0.27–0.90; DALYs: 47.74, 95% UI: 24.30–80.97), and Commonwealth Middle-Income regions (deaths: 0.39, 95% UI: 0.21–0.63; DALYs: 34.93, 95% UI: 18.91–56.30; Supplementary Tables S1, S2).

**Figure 3:**
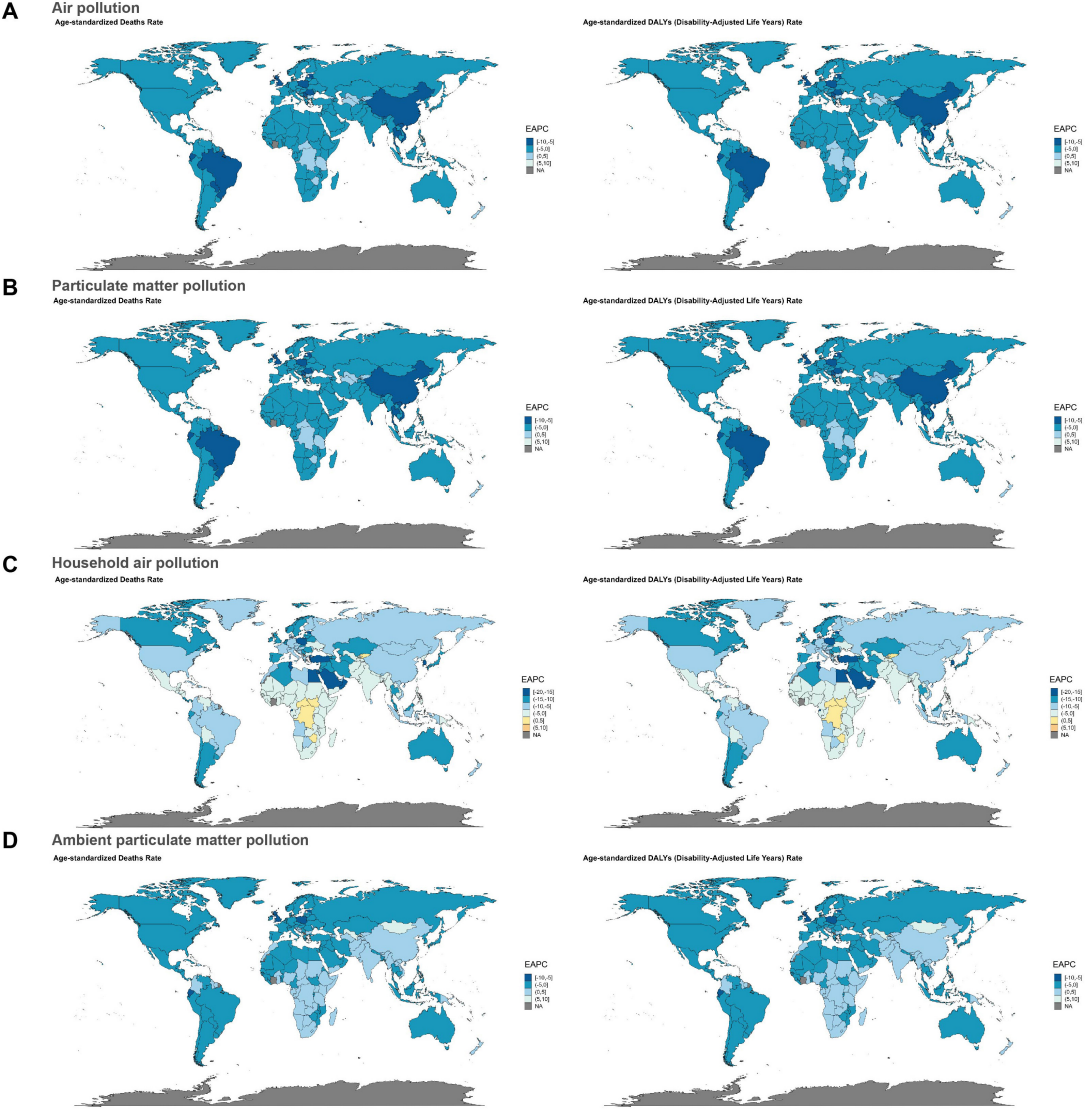
Results of cluster analysis based on the EAPC values from 1990 to 2021. **(A)** Air pollution, **(B)** particulate matter pollution, **(C)** household air pollution, and **(D)** ambient particulate matter pollution.

Subsequently, hierarchical cluster analyses were performed to identify regions with similar changes in disease burden. For neonatal sepsis and other neonatal infections due to air pollution and particulate matter pollution, age-standardized deaths and DALYs rate significantly increased in 19 regions including Southern Sub-Saharan Africa and Central Africa, whereas Tropical Latin America, East Asia, and Central Europe exhibited significant decreases (Figures 3A, B). In relation to household air pollution from solid fuels, 18 regions, including Eastern Sub-Saharan Africa and Eastern Africa, showed significant increases, while 21 regions, including the Middle East and Northern Africa, showed significant decreases (Figure 3C). For ambient particulate matter pollution, Central Asia, the Caribbean, Central Sub-Saharan Africa, and Commonwealth middle-income regions demonstrated significant increases in deaths and DALY rates, whereas 25 regions, including Southern Africa and Africa, showed significant declines (Figure 3D).

### 3.4 National heterogeneity in attributable burden

Among 204 countries, Sierra Leone, Mozambique, and Somalia ranked as the top three nations in terms of age-standardized deaths and DALYs rate due to neonatal sepsis and other neonatal infections attributed to air pollution, particulate matter pollution, and household air pollution from solid fuels (Supplementary Tables S1, S2, Supplementary Figure S4). Notably, all three countries with the highest ASRs are located in Africa-specifically, Sierra Leone in Western Sub-Saharan Africa, Mozambique in southeastern Africa, and Somalia in Eastern Sub-Saharan Africa. Additionally, two of the top three countries with the highest age-standardized deaths and DALYs rate attributable to ambient particulate matter pollution-Ghana and Nigeria-are also in Africa, specifically Western Sub-Saharan Africa (Supplementary Tables S1, S2, Supplementary Figure S3). These findings are consistent with our regional analysis, which identifies Africa as the region experiencing the highest global burden in terms of deaths and DALYs from neonatal sepsis and other neonatal infections attributable to air pollution, particulate matter pollution, household air pollution from solid fuels, and ambient particulate matter pollution. In contrast, high-income countries (such as those in North America, Europe, and the high-income Asia-Pacific region) showed significantly lower deaths and DALYs rate compared to low-income countries.

### 3.5 Decomposition analysis

**Figure 4:**
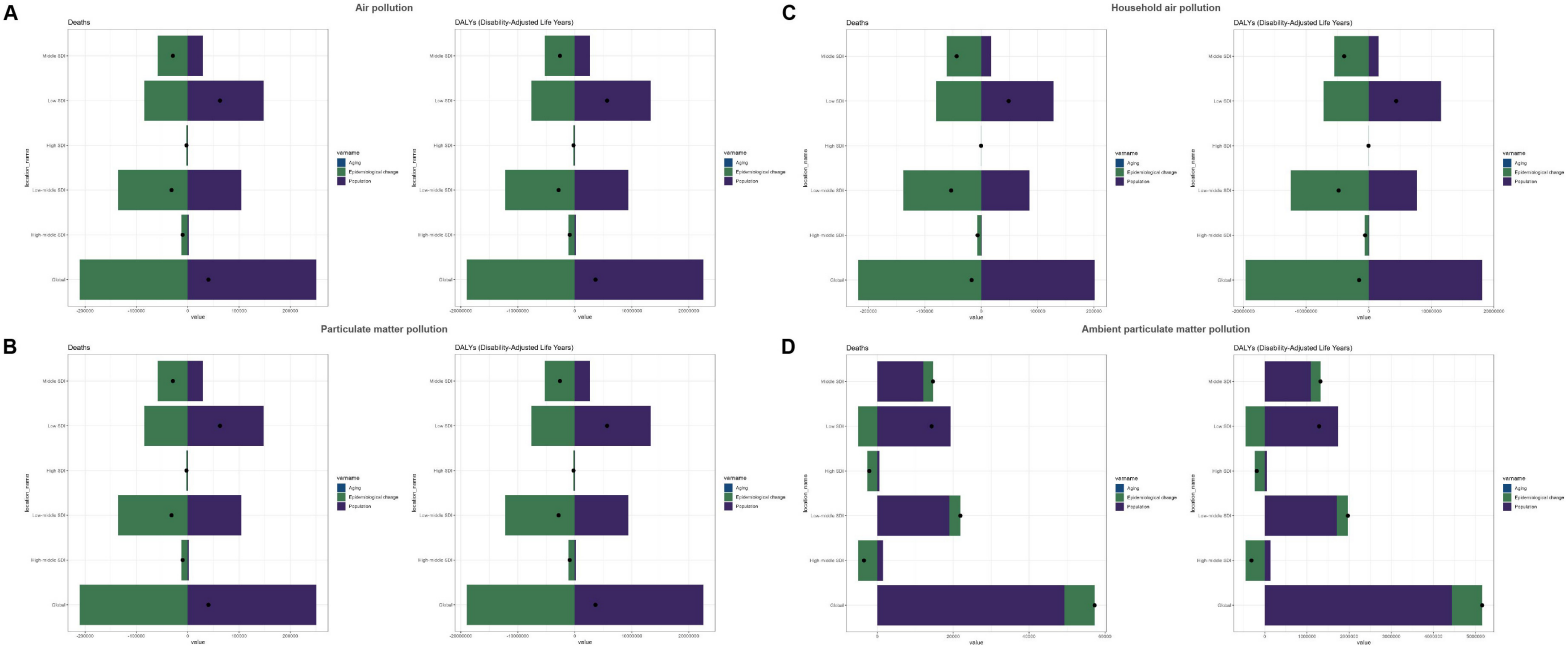
Decomposition analysis results for the global population and five SDI regions. **(A)** Air pollution, **(B)** particulate matter pollution, **(C)** household air pollution, and **(D)** ambient particulate matter pollution.

The decomposition analysis indicated that globally, population was the primary driver of the increase in disease burden associated with air pollution (deaths: 621.99%; DALYs: 622.57%), whereas epidemiological changes significantly reduced this burden (deaths: −521.99%; DALYs: −522.57%). These two factors had opposite effects, but the influence of population was stronger, resulting in a net increase in burden (deaths: 40,294.84; DALYs: 3,623,017.97). Among the different SDI regions, the low-SDI region was most profoundly affected by population (deaths: 234.46%; DALYs: 234.49%), although epidemiological changes partly mitigated this adverse impact (deaths: −134.46%; DALYs: −134.49%). Conversely, the high-SDI region experienced the smallest impact from population (deaths: −23.84%; DALYs: −23.86%), achieving a net reduction in burden due to favorable epidemiological changes (deaths: 123.84%; DALYs: 123.86%; Supplementary Table S3, Figure 4A). For particulate matter pollution, global and regional trends were consistent with those observed for air pollution, with the low-SDI region again identified as a critical target for intervention (Supplementary Table S3, Figure 4B). Regarding household air pollution from solid fuels, globally, population substantially increased the disease burden (deaths: −1,181.79%; DALYs: −1,179.57%), yet epidemiological changes exerted an even stronger counter-effect (deaths: 1,281.79%; DALYs: 1,279.57%), resulting in a net global reduction in burden (deaths: −17,034.57; DALYs: −1,535,914.84). Among SDI regions, the low-SDI region experienced the greatest increase due to population (deaths: 263.70%; DALYs: 263.74%), with epidemiological changes having a smaller mitigating effect (deaths: −163.70%; DALYs: −163.74%), leading to a net increase in burden. The high-SDI region saw minimal impact from population (deaths/DALYs: −12.34%), with epidemiological changes contributing significantly to reductions in burden (deaths/DALYs: 112.34%). The low-middle SDI region showed the greatest benefit from epidemiological changes (deaths: 259.65%; DALYs: 259.63%; Supplementary Table S3, Figure 4C). In terms of ambient particulate matter pollution, globally, population remained the dominant factor driving the increase in disease burden (deaths: 86.03%; DALYs: 86.05%), while epidemiological changes had a smaller impact (deaths: 13.97%; DALYs: 13.95%). The low-SDI region was disproportionately impacted by population (deaths/DALYs: 135.03%), though this was partially alleviated by epidemiological changes (deaths/DALYs: −35.03%). In the high-SDI region, epidemiological changes significantly reduced the disease burden (deaths: 125.63%; DALYs: 125.64%), more than compensating for the adverse effect of population (deaths: −25.63%; DALYs: −25.64%; Supplementary Table S3, Figure 4D).

### 3.6 The predicted results from 2022 to 2050

**Figure 5:**
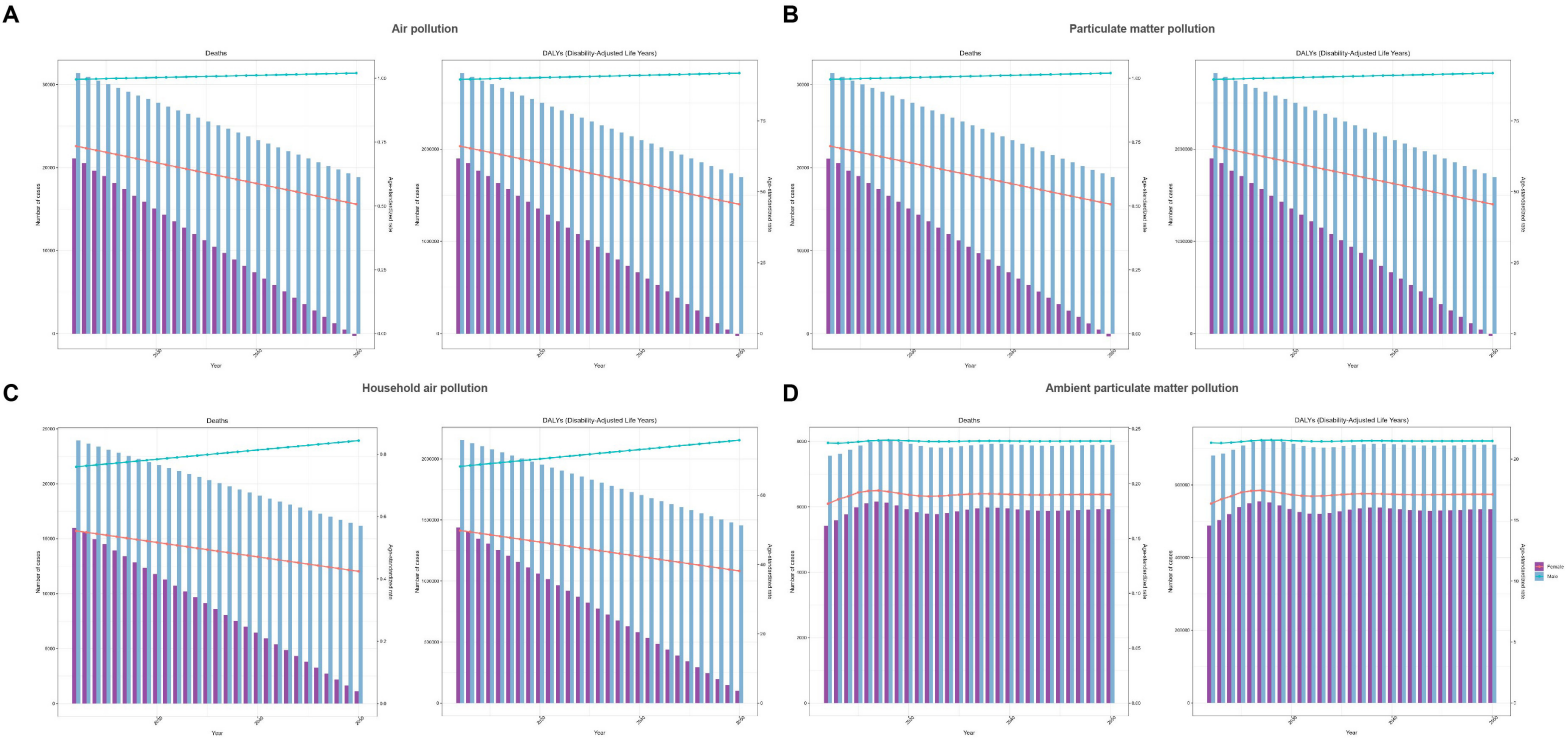
Predicted trends for neonatal sepsis and other neonatal infections deaths and DALYs. **(A)** Air pollution, **(B)** particulate matter pollution, **(C)** household air pollution, and **(D)** ambient particulate matter pollution.

Forecast results from the ARIMA model indicated that from 2022 to 2050, deaths and DALYs attributable to neonatal sepsis and other neonatal infections associated with air pollution, particulate matter pollution, and household air pollution from solid fuels are predicted to decrease annually in both males and females, with females experiencing a more rapid decline. However, age-standardized deaths and DALYs rate in males are projected to slightly increase, while females are expected to show a continuous decreasing trend (Supplementary Tables S4, S6, Figures 5A–C). Conversely, deaths and DALYs associated with ambient particulate matter pollution in males are anticipated to initially increase, reaching a peak in 2027, before subsequently declining, with stable trends observed for corresponding age-standardized deaths and DALYs rate. Females are predicted to exhibit similar trends, with deaths, DALYs, and corresponding ASRs first increasing, peaking in 2027, and then declining thereafter (Supplementary Table S7, Figure 5D).

## 4 Discussion

Air pollution has emerged as a critical global public health issue. With rapid industrialization and ongoing urbanization, emissions of air pollutants continue to rise, posing serious threats to human health and ecosystems (28, 29). Neonates are particularly vulnerable due to their rapid growth stage and immature immune defense systems, making them more susceptible to health damage from exposure to air pollutants (30, 31). Previous studies indicate that inhaled air pollutants may induce the accumulation of free radicals in the lungs, exacerbating oxidative stress and subsequently triggering inflammatory cascades, resulting in substantial cytokine release and eventual damage to overall health (32). Furthermore, prenatal exposure to air pollutants can cross the placental barrier, enter fetal circulation, impair immune system development, and potentially lead to neonatal mortality (33). Prior research also suggests that air pollutants may increase the risk of neonatal sepsis and other neonatal infections, with a gender disparity, as males experience higher DALYs compared to females (34).

Multiple studies have revealed significant associations between prenatal exposure to air pollution and the risk of neonatal sepsis. A large-scale European study encompassing 10 birth cohorts (*n* = 16,059) found that exposure to air pollution at birth increased the risk of pneumonia in neonates. During the 0–1 year period, exposure to pollutants such as PM_2_.5 and NO_2_ exhibited a stronger association with neonatal pneumonia compared to the entire early childhood phase (e.g., 0–5 years). Specifically, each 5 μg/m^3^ increase in PM_2_.5 was associated with a four-fold increased risk of pneumonia (OR = 4.06; 95% CI: 1.93–8.57) (35), which may further progress to sepsis. Another retrospective cohort study focusing on singleton pregnancies further confirmed that elevated prenatal exposure to nitrogen dioxide (NO_2_) significantly increased the risk of neonatal sepsis evaluation, particularly in cases of early preterm birth (22). Additionally, gestational exposure to fine PM_2_.5 may lead to alveolar developmental abnormalities and immune dysregulation (12, 15), thereby impairing the neonate's defense against infections. Mechanistic studies suggest that air pollution promotes the onset and progression of sepsis through multiple pathways. On one hand, pollutants such as diesel exhaust particles can impair pulmonary antibacterial function, reduce macrophage-mediated bacterial clearance efficiency, and enhance the colonization capacity of Streptococcus pneumoniae, increasing its risk of hematogenous dissemination (36–38). Notably, S. pneumoniae is a key pathogen implicated in early-onset neonatal sepsis (39). Importantly, compared to sepsis originating from other sources, pulmonary sepsis is associated with significantly higher mortality. For instance, one study reported a 76% higher mortality rate in sepsis secondary to pneumonia compared to non-pulmonary sepsis (95% CI: 11%−178%) (40). This suggests that the impact of air pollutants on respiratory infections may directly contribute to the increased risk of sepsis-related mortality. Furthermore, air pollution may indirectly exacerbate sepsis mortality through its well-documented systemic inflammatory and oxidative stress effects-both of which disrupt immune responses and impair the body's ability to clear potential infections.

Our study is the first to systematically assess and quantify the global burden of neonatal sepsis and other neonatal infections attributable to air pollution (including particulate matter pollution, household air pollution from solid fuels, and ambient particulate matter pollution), and further forecast future trends. The results highlight air pollution as a major environmental risk factor contributing to neonatal sepsis and other neonatal infections, with significant disparities observed across socio-economic levels and geographic regions. Globally, deaths, DALYs, and corresponding ASRs due to neonatal sepsis and other neonatal infections associated with air pollution (including particulate matter pollution and household air pollution from solid fuels) have generally exhibited a decreasing trend, whereas the burden associated with ambient particulate matter pollution has continued to increase. This declining burden is largely attributed to the widespread implementation of control measures for household air pollution from solid fuels and improvements in healthcare conditions (41). Conversely, the rising burden from ambient particulate matter pollution highlights the inadequacy of environmental management strategies in certain regions (42, 43).

Substantial research evidence indicates that the exposure risks and health impacts of air pollution exhibit significant inequalities, with disproportionately higher burdens experienced by low-income and marginalized communities (44, 45). Existing evidence explicitly demonstrates that air pollution has emerged as a leading cause of mortality in low- and middle-income countries (46). These countries commonly face challenges including lenient air-quality regulations, widespread use of outdated and highly polluting machinery and vehicles, persistent fossil fuel subsidy policies, congested urban transportation systems, rapid industrial sector expansion, and slash-and-burn agricultural practices-collectively driving elevated concentrations of air pollutants (47). Our data similarly indicate that the burden of neonatal sepsis and other neonatal infections attributable to air pollution (including particulate matter pollution, household air pollution from solid fuels, and ambient particulate matter pollution) is significantly greater in low SDI regions compared to high SDI regions. This aligns with previous findings reporting high prevalence rates of neonatal sepsis in Sub-Saharan Africa (48), and also matches the spatial distribution of PM_2.5_ exposure (25). Furthermore, earlier studies combining satellite-derived atmospheric PM_2.5_ data with household survey data found a strong linear association between infant mortality rates and PM_2.5_ levels in Sub-Saharan Africa, with infant mortality under 1 year increasing by 9.2% for every 10 μg/m^3^ rise in PM_2.5_ concentration (49). These studies collectively suggest that socioeconomic factors play a crucial role in mediating pollution-related health outcomes. The global air quality report highlighted that African countries rank among the highest globally in terms of deaths attributed to PM_2.5_ pollution-a phenomenon compounded by limited access to medical resources, socioeconomic development disparities, and demographic shifts (50). Notably, even in regions experiencing modest declines in PM_2.5_ concentrations, continued population growth may increase the overall burden of air pollution-related diseases, potentially explaining the persistently high child mortality rates observed in Africa (51).

Additionally, gender disparities were evident in our results, revealing higher deaths, DALYs, and corresponding ASRs for neonatal sepsis and other neonatal infections attributable to air pollution among males compared to females, suggesting males may face greater risk of neonatal sepsis and related deaths associated with air pollution. Prior research has shown clear variations in air pollution-related public health impacts across different age groups and genders, noting heightened vulnerability among older adults individuals and children under five, with males experiencing more severe disease burdens from air pollution than females (52). Furthermore, forecasting results using the ARIMA model for the next 29 years warn that without stronger intervention measures, the burden of neonatal infections caused by ambient particulate matter pollution will continue to rise, peaking notably around 2027. These findings underscore the necessity of prioritizing air pollution-targeted interventions-particularly in low-income countries-as essential to advancing progress toward achieving the United Nations Sustainable Development Goal (SDG 3.2) of reducing childhood mortality (53). Results from decomposition analyses revealed a complex interaction between population dynamics and epidemiological changes across different environmental risk factors (air pollution, particulate matter pollution, household air pollution from solid fuels, and ambient particulate matter pollution). Globally, population was identified as the main driving force behind the rising disease burden attributable to air pollution (54). Conversely, epidemiological changes partially counteracted this increase, indicating the effectiveness of public health interventions such as clean air policies and improved healthcare (43, 55). Low SDI regions, notably Sub-Saharan Africa, experienced the most pronounced negative impact from population, although epidemiological improvements provided some mitigation (56). High SDI regions, such as countries in Europe and North America, experienced relatively minor impacts, reflecting their advanced pollution control measures and mature healthcare systems (55). For particulate matter pollution, global trends mirrored those observed for overall air pollution, with low SDI regions again bearing the heaviest burden, underscoring particulate matter pollution as a significant health threat in developing countries, possibly due to rapid urbanization, industrialization, and increasing vehicular emissions (47). In terms of household air pollution from solid fuels, population contributed substantially to the global disease burden, likely related to continued reliance on solid fuels (such as wood and coal) in certain low-income countries (57, 58). Regarding ambient particulate matter pollution, population remained the dominant driver, especially in low SDI nations (e.g., India and parts of Africa), suggesting that urbanization and industrial expansion continue to exacerbate pollution (59, 60). Although global air pollution control efforts have made some progress, ambient particulate matter pollution continues to pose a significant challenge. For example, almost all African cities with available PM_2.5_ monitoring data exceed World Health Organization Air Quality Guidelines (WHO AQG) (61). Additionally, research specifically addressing neonatal vulnerability to PM_2.5_ exposure remains limited, leading to insufficient evidence to guide targeted policy interventions.

Our projections suggest that the disease burden from ambient particulate matter pollution will reach its maximum level in 2027. Therefore, countries and regions should establish air pollution control frameworks tailored to their respective developmental stages. Strengthening emissions regulation in manufacturing and transportation sectors, promoting electrified public transportation infrastructure, advancing clean energy transitions, and progressively replacing high-carbon energy sources with cleaner alternatives are critical strategies. Furthermore, implementing clean household fuel programs, reducing solid fuel and kerosene usage, and expanding access to clean domestic energy could significantly decrease exposure to indoor and outdoor PM_2.5_ and CO. Additionally, governments must develop differentiated air-quality-based management policies and strengthen public education to raise awareness of the health hazards associated with environmental pollution, systematically reducing air pollution levels (62, 63).

Based on the identified association between air pollution exposure and neonatal sepsis burden and its regional disparities, the following integrated intervention strategies are proposed: in low-SDI regions (e.g., Sub-Saharan Africa), priority should be given to promoting clean cooking stoves to replace solid fuels, while concurrently strengthening early diagnostic capability for neonatal sepsis. In middle- and high-SDI countries, industrial and transportation emission regulations must be enhanced, particularly targeting particulate matter restrictions for diesel vehicles. All regions should establish pollution-health co-monitoring systems, integrating real-time PM_2_.5 data into maternal and child health platforms to enable risk warnings for pregnant women and newborns. The international community should support clean energy transitions in low-SDI countries through the Green Climate Fund and incorporate neonatal PM_2_.5 exposure metrics into revised WHO Air Quality Guidelines. Additionally, sex-differentiated research is needed to elucidate the mechanisms underlying higher risks in male neonates, while emergency response plans should be developed for the projected peak pollution burden in 2027.These measures must be adapted to local socioeconomic conditions and implemented through cross-sector collaboration to achieve SDG 3.2's child health targets.

This study has certain limitations. First, our analyses were conducted at the national level and did not incorporate provincial or state-level variations. Given substantial heterogeneity in air pollution levels, demographic structures, and healthcare resource distributions within countries, findings might have limited applicability at subnational scales. Additionally, our analyses relied exclusively on the GBD database without integrating other internationally recognized datasets. Differences in data collection methodologies, coverage, and quality control across databases could introduce systematic biases or underestimations of region-specific impacts. Finally, the selection of the ARIMA model may have certain limitations. For instance, when making long-term predictions, the ARIMA model may exhibit certain deviations. Subsequently, the prediction model needs to be optimized and further research should be conducted.

## 5 Conclusion

In summary, at a global level, deaths and DALYs attributable to neonatal sepsis and other neonatal infections caused by air pollution (including particulate matter pollution and household air pollution from solid fuels) have declined. However, the disease burden from ambient particulate matter pollution is projected to rise continuously, particularly in low-income countries. This underscores ambient particulate matter pollution as an ongoing critical public health issue requiring targeted institutional strategies and interventions to further alleviate the burden in low SDI regions.

## Data Availability

The original contributions presented in the study are included in the article/Supplementary material, further inquiries can be directed to the corresponding authors.
