# Supplementary material for: The global burden of neonatal sepsis attributable to air pollution from 1990 to 2021: findings from the global burden of disease study 2021
Source: Front Public Health. 2025 Sep 24;13:1644191. doi: 10.3389/fpubh.2025.1644191 (PMC12504511; doi:10.3389/fpubh.2025.1644191)
Supplement: Supplementary file 1 [file Table_1.docx]

**Supplementary material**

Table S1: The number of death and the age-standardized deaths rate of neonatal sepsis and other neonatal infections and changes in regions and 204 countries.

| Characteristics | No. of deaths | | | | |
| --- | --- | --- | --- | --- | --- |
|  | 1990 | | 2021 | | 1990-2021 |
|  | Number of deaths cases (95% UI) | Age-standardised rate per 100,000 population (95% UI) | Number of deaths cases (95% UI) | Age-standardised rate per 100,000 population (95% UI) | EAPC (95%CI) |
| **Air pollution** |  |  |  |  |  |
| **region_eapc** |  |  |  |  |  |
| Advanced Health System | 674 (523-865) | 0.07 (0.06-0.1) | 160 (120-213) | 0.02 (0.02-0.03) | -4.39 (-5.14--3.63) |
| Africa | 28559 (24134-33103) | 2.23 (1.89-2.59) | 34020 (27605-41543) | 1.65 (1.34-2.01) | -1.35 (-2.15--0.54) |
| African Region | 27777 (23640-32090) | 2.59 (2.2-2.99) | 32700 (26482-39710) | 1.82 (1.48-2.21) | -1.47 (-2.32--0.61) |
| America | 3747 (3260-4269) | 0.49 (0.43-0.56) | 1327 (1002-1702) | 0.19 (0.15-0.25) | -3.64 (-3.93--3.34) |
| Andean Latin America | 806 (564-1118) | 1.43 (1-1.99) | 220 (130-352) | 0.37 (0.22-0.59) | -4.47 (-4.8--4.13) |
| Asia | 37715 (31875-44362) | 0.99 (0.84-1.16) | 18517 (14907-22603) | 0.61 (0.49-0.74) | -2.74 (-3--2.49) |
| Australasia | 2 (0-4) | 0.01 (0-0.03) | 1 (0-2) | 0.01 (0-0.01) | -2.85 (-3.69--2.01) |
| Basic Health System | 11255 (9335-13441) | 0.44 (0.36-0.52) | 3577 (2905-4433) | 0.19 (0.15-0.24) | -3.73 (-4.1--3.36) |
| Caribbean | 462 (328-650) | 1.07 (0.76-1.51) | 455 (276-688) | 1.19 (0.72-1.8) | -0.19 (-0.37-0) |
| Central Africa | 2150 (1598-2847) | 1.44 (1.07-1.9) | 3315 (2264-4643) | 1.23 (0.84-1.72) | -0.67 (-1.62-0.28) |
| Central Asia | 123 (90-173) | 0.13 (0.1-0.18) | 134 (98-176) | 0.14 (0.1-0.18) | -0.07 (-0.28-0.14) |
| Central Europe | 48 (36-62) | 0.06 (0.04-0.07) | 4 (3-5) | 0.01 (0.01-0.01) | -7.63 (-8.63--6.62) |
| Central Latin America | 1131 (978-1295) | 0.47 (0.41-0.54) | 441 (323-575) | 0.23 (0.17-0.31) | -3.34 (-3.67--3.01) |
| Central Sub-Saharan Africa | 1132 (721-1716) | 0.92 (0.59-1.4) | 1349 (728-2349) | 0.63 (0.34-1.1) | -1.31 (-2.27--0.34) |
| Commonwealth High Income | 15 (11-19) | 0.02 (0.01-0.02) | 6 (4-9) | 0.01 (0.01-0.01) | -3.15 (-3.84--2.44) |
| Commonwealth Low Income | 13355 (10210-17436) | 3.11 (2.38-4.06) | 8672 (6976-11009) | 1.85 (1.49-2.35) | -2.29 (-2.98--1.6) |
| Commonwealth Middle Income | 28046 (23989-32733) | 1.58 (1.35-1.84) | 23804 (19522-28666) | 1.2 (0.98-1.45) | -1.68 (-2.03--1.33) |
| East Asia | 1382 (1061-1808) | 0.12 (0.09-0.16) | 135 (96-175) | 0.02 (0.02-0.03) | -6.02 (-6.76--5.26) |
| East Asia & Pacific - WB | 9311 (7270-11778) | 0.5 (0.39-0.64) | 3204 (2493-4231) | 0.27 (0.21-0.36) | -3.31 (-3.86--2.77) |
| Eastern Africa | 10447 (8420-12948) | 2.7 (2.18-3.35) | 10751 (8040-13629) | 1.86 (1.39-2.35) | -1.65 (-2.52--0.78) |
| Eastern Europe | 137 (112-163) | 0.1 (0.08-0.11) | 38 (30-47) | 0.04 (0.04-0.05) | -3.3 (-4.27--2.31) |
| Eastern Mediterranean Region | 3995 (2944-5365) | 0.63 (0.46-0.84) | 4775 (3542-6361) | 0.58 (0.43-0.77) | -1.16 (-1.64--0.67) |
| Eastern Sub-Saharan Africa | 12963 (10860-15508) | 3.03 (2.54-3.62) | 13474 (10169-17013) | 2.06 (1.55-2.6) | -1.69 (-2.59--0.78) |
| Europe | 543 (409-711) | 0.1 (0.08-0.13) | 126 (92-171) | 0.03 (0.02-0.04) | -4.19 (-5--3.38) |
| Europe & Central Asia - WB | 617 (472-783) | 0.1 (0.08-0.13) | 238 (185-301) | 0.05 (0.04-0.06) | -2.8 (-3.51--2.08) |
| European Region | 619 (474-785) | 0.1 (0.08-0.13) | 239 (186-302) | 0.05 (0.04-0.06) | -2.81 (-3.51--2.1) |
| High-income Asia Pacific | 27 (15-43) | 0.03 (0.02-0.05) | 4 (3-6) | 0.01 (0-0.01) | -5.61 (-6.71--4.49) |
| High-income North America | 48 (40-57) | 0.02 (0.02-0.03) | 18 (13-23) | 0.01 (0.01-0.01) | -3.68 (-4.34--3.01) |
| Latin America & Caribbean - WB | 3700 (3211-4221) | 0.67 (0.58-0.77) | 1310 (984-1682) | 0.27 (0.2-0.35) | -3.69 (-3.93--3.46) |
| Limited Health System | 50750 (43720-58870) | 1.95 (1.68-2.26) | 39249 (32655-46673) | 1.32 (1.09-1.56) | -1.99 (-2.44--1.54) |
| Middle East & North Africa - WB | 704 (521-939) | 0.18 (0.13-0.24) | 390 (270-516) | 0.09 (0.06-0.12) | -2.88 (-3.24--2.52) |
| Minimal Health System | 7886 (6219-9768) | 2.55 (2.01-3.16) | 11004 (8400-14292) | 1.79 (1.37-2.33) | -1.35 (-2.36--0.34) |
| North Africa and Middle East | 1127 (866-1438) | 0.22 (0.17-0.27) | 692 (497-924) | 0.12 (0.09-0.16) | -2.68 (-3.03--2.33) |
| North America | 48 (40-57) | 0.02 (0.02-0.03) | 18 (13-23) | 0.01 (0.01-0.01) | -3.68 (-4.34--3.01) |
| Northern Africa | 285 (192-399) | 0.15 (0.1-0.21) | 178 (120-252) | 0.08 (0.06-0.12) | -2.51 (-2.91--2.1) |
| Oceania | 42 (25-70) | 0.39 (0.23-0.65) | 80 (43-133) | 0.39 (0.21-0.65) | -0.2 (-0.81-0.41) |
| Region of the Americas | 3747 (3260-4269) | 0.49 (0.43-0.56) | 1327 (1002-1702) | 0.19 (0.15-0.25) | -3.64 (-3.93--3.34) |
| South-East Asia Region | 29656 (24936-35466) | 1.64 (1.38-1.96) | 13330 (10327-16717) | 0.87 (0.67-1.09) | -3.13 (-3.39--2.86) |
| South Asia | 27591 (23091-33460) | 1.68 (1.41-2.04) | 14685 (11591-18401) | 0.97 (0.77-1.22) | -2.86 (-3.17--2.55) |
| South Asia - WB | 27788 (23252-33643) | 1.66 (1.39-2) | 14903 (11771-18616) | 0.94 (0.74-1.17) | -2.87 (-3.18--2.56) |
| Southeast Asia | 7985 (6123-10483) | 1.35 (1.03-1.77) | 3009 (2305-4007) | 0.56 (0.43-0.74) | -3.65 (-3.89--3.42) |
| Southern Africa | 3215 (2528-3995) | 1.86 (1.46-2.31) | 3123 (2294-4229) | 1.27 (0.94-1.72) | -1.62 (-2.29--0.95) |
| Southern Latin America | 94 (54-138) | 0.18 (0.11-0.27) | 22 (10-34) | 0.06 (0.03-0.09) | -5.14 (-5.52--4.75) |
| Southern Sub-Saharan Africa | 599 (443-760) | 0.77 (0.57-0.98) | 536 (399-746) | 0.69 (0.51-0.96) | -0.6 (-0.93--0.27) |
| Sub-Saharan Africa - WB | 28398 (24049-32957) | 2.59 (2.19-3.01) | 33928 (27523-41437) | 1.83 (1.49-2.24) | -1.45 (-2.33--0.57) |
| Tropical Latin America | 1223 (1022-1467) | 0.76 (0.63-0.91) | 188 (135-247) | 0.11 (0.08-0.15) | -6.43 (-6.85--6.01) |
| Western Africa | 12462 (9981-15007) | 3.25 (2.6-3.91) | 16654 (13705-20224) | 2.21 (1.82-2.69) | -1.5 (-2.45--0.54) |
| Western Europe | 58 (45-74) | 0.03 (0.02-0.03) | 17 (11-24) | 0.01 (0.01-0.01) | -3.59 (-4.45--2.71) |
| Western Pacific Region | 4769 (3901-5841) | 0.32 (0.26-0.4) | 1617 (1300-1973) | 0.19 (0.15-0.23) | -3.11 (-3.73--2.48) |
| Western Sub-Saharan Africa | 13627 (11063-16278) | 3.2 (2.6-3.82) | 18524 (15196-22413) | 2.18 (1.79-2.64) | -1.51 (-2.46--0.54) |
| **country_eapc** |  |  |  |  |  |
| Afghanistan | 79 (42-130) | 0.37 (0.2-0.62) | 196 (107-331) | 0.34 (0.18-0.57) | -0.88 (-1.9-0.16) |
| Albania | 0 (0-0) | 0 (0-0.01) | 0 (0-0) | 0 (0-0.01) | -2.54 (-3.53--1.53) |
| Algeria | 16 (6-33) | 0.04 (0.02-0.09) | 11 (3-30) | 0.02 (0.01-0.07) | -2.42 (-2.68--2.17) |
| American Samoa | 0 (0-0) | 0.02 (0-0.05) | 0 (0-0) | 0.01 (0-0.02) | -5.86 (-6.52--5.2) |
| Andorra | 0 (0-0) | 0.01 (0-0.03) | 0 (0-0) | 0 (0-0.01) | -6.02 (-7.32--4.71) |
| Angola | 359 (202-583) | 1.5 (0.85-2.44) | 263 (144-451) | 0.45 (0.25-0.78) | -4.51 (-5.53--3.47) |
| Antigua and Barbuda | 0 (0-0) | 0.08 (0.02-0.14) | 0 (0-0) | 0.09 (0.03-0.16) | -0.54 (-1.02--0.06) |
| Argentina | 72 (35-116) | 0.22 (0.11-0.35) | 16 (6-28) | 0.06 (0.02-0.11) | -5.51 (-5.86--5.15) |
| Armenia | 24 (13-38) | 0.65 (0.35-1.05) | 7 (4-11) | 0.41 (0.22-0.64) | -2.8 (-3.38--2.22) |
| Australia | 2 (0-4) | 0.01 (0-0.03) | 1 (0-2) | 0.01 (0-0.01) | -3.65 (-4.57--2.72) |
| Austria | 1 (0-1) | 0.01 (0.01-0.02) | 0 (0-0) | 0 (0-0.01) | -3.71 (-4.69--2.72) |
| Azerbaijan | 16 (7-30) | 0.18 (0.08-0.33) | 7 (3-16) | 0.12 (0.05-0.24) | -2.6 (-2.99--2.21) |
| Bahamas | 1 (0-1) | 0.21 (0.09-0.37) | 0 (0-1) | 0.17 (0.07-0.31) | -1.65 (-2.24--1.06) |
| Bahrain | 1 (0-1) | 0.11 (0.06-0.17) | 0 (0-0) | 0.03 (0.02-0.05) | -4.88 (-5.29--4.47) |
| Bangladesh | 7136 (4365-11015) | 3.53 (2.16-5.42) | 2020 (1109-3277) | 1.5 (0.83-2.44) | -3.87 (-4.33--3.41) |
| Barbados | 0 (0-1) | 0.23 (0.11-0.37) | 0 (0-1) | 0.3 (0.13-0.56) | 0.41 (-0.38-1.21) |
| Belarus | 8 (4-15) | 0.12 (0.06-0.22) | 2 (0-3) | 0.04 (0.01-0.08) | -3.77 (-4.71--2.82) |
| Belgium | 2 (1-3) | 0.03 (0.01-0.05) | 1 (0-1) | 0.01 (0-0.02) | -4 (-4.81--3.19) |
| Belize | 1 (1-2) | 0.44 (0.24-0.68) | 1 (1-2) | 0.3 (0.15-0.47) | -1.88 (-2.33--1.42) |
| Benin | 379 (223-572) | 3.34 (1.96-5.04) | 541 (336-852) | 2.16 (1.34-3.4) | -1.58 (-2.58--0.56) |
| Bermuda | 0 (0-0) | 0.01 (0-0.03) | 0 (0-0) | 0 (0-0.01) | -4.75 (-5.6--3.89) |
| Bhutan | 12 (7-19) | 1.11 (0.67-1.8) | 1 (1-2) | 0.23 (0.11-0.42) | -6.29 (-6.65--5.93) |
| Bolivia (Plurinational State of) | 150 (88-239) | 1.37 (0.81-2.19) | 52 (25-92) | 0.44 (0.21-0.78) | -4.58 (-4.96--4.19) |
| Bosnia and Herzegovina | 2 (1-6) | 0.07 (0.02-0.17) | 0 (0-1) | 0.03 (0.01-0.05) | -4.44 (-5.43--3.44) |
| Botswana | 22 (14-35) | 1 (0.62-1.56) | 11 (5-20) | 0.49 (0.23-0.86) | -2.77 (-3.19--2.36) |
| Brazil | 1156 (959-1394) | 0.75 (0.62-0.9) | 180 (129-239) | 0.11 (0.08-0.15) | -6.34 (-6.76--5.91) |
| Brunei Darussalam | 0 (0-0) | 0.04 (0.01-0.08) | 0 (0-0) | 0.04 (0.01-0.09) | -1.07 (-1.54--0.59) |
| Bulgaria | 0 (0-0) | 0.01 (0-0.01) | 0 (0-0) | 0.01 (0-0.02) | 0.91 (-0.94-2.79) |
| Burkina Faso | 768 (455-1217) | 3.48 (2.06-5.5) | 1147 (690-1796) | 2.52 (1.52-3.95) | -1.09 (-2.14--0.03) |
| Burundi | 350 (217-537) | 2.82 (1.74-4.32) | 461 (278-745) | 2.06 (1.24-3.33) | -1.18 (-2.12--0.24) |
| Cabo Verde | 6 (3-10) | 1.06 (0.57-1.71) | 2 (1-4) | 0.55 (0.31-0.96) | -3.44 (-3.87--3.02) |
| Cambodia | 347 (191-548) | 1.69 (0.93-2.68) | 134 (75-216) | 0.78 (0.44-1.26) | -3.99 (-4.45--3.54) |
| Cameroon | 535 (330-822) | 2.35 (1.45-3.6) | 738 (440-1152) | 1.48 (0.88-2.32) | -1.84 (-2.73--0.95) |
| Canada | 2 (0-4) | 0.01 (0-0.02) | 1 (0-2) | 0.01 (0-0.01) | -2.32 (-3.12--1.51) |
| Central African Republic | 79 (47-136) | 1.31 (0.79-2.26) | 120 (66-208) | 1.32 (0.73-2.29) | -0.17 (-1.11-0.79) |
| Chad | 488 (288-734) | 3.2 (1.89-4.8) | 1029 (603-1642) | 2.49 (1.46-3.97) | -0.92 (-2.12-0.29) |
| Chile | 18 (10-27) | 0.12 (0.07-0.18) | 5 (2-8) | 0.05 (0.02-0.08) | -3.58 (-4.23--2.92) |
| China | 1349 (1031-1756) | 0.12 (0.09-0.16) | 124 (88-161) | 0.02 (0.02-0.03) | -6.19 (-6.94--5.43) |
| Colombia | 99 (57-148) | 0.22 (0.13-0.33) | 42 (16-76) | 0.13 (0.05-0.23) | -2.33 (-3.02--1.65) |
| Comoros | 29 (17-44) | 3.08 (1.82-4.72) | 16 (10-26) | 1.99 (1.18-3.17) | -2.36 (-2.95--1.75) |
| Congo | 35 (16-63) | 0.79 (0.36-1.44) | 38 (17-66) | 0.62 (0.28-1.08) | -1.3 (-2.03--0.56) |
| Cook Islands | 0 (0-0) | 0.03 (0-0.08) | 0 (0-0) | 0.01 (0-0.03) | -6.15 (-6.72--5.58) |
| Costa Rica | 4 (2-6) | 0.09 (0.05-0.15) | 1 (0-2) | 0.03 (0.01-0.06) | -5.13 (-5.55--4.7) |
| C么te d'Ivoire | 880 (558-1344) | 3.33 (2.12-5.09) | 895 (503-1456) | 1.97 (1.11-3.21) | -2.1 (-2.95--1.24) |
| Croatia | 0 (0-1) | 0.01 (0.01-0.02) | 0 (0-1) | 0.02 (0.01-0.04) | -0.5 (-2.17-1.2) |
| Cuba | 9 (3-15) | 0.1 (0.04-0.17) | 2 (1-4) | 0.04 (0.01-0.07) | -3.58 (-4.37--2.79) |
| Cyprus | 0 (0-1) | 0.04 (0.01-0.08) | 0 (0-0) | 0.01 (0-0.02) | -5.98 (-6.64--5.32) |
| Czechia | 1 (0-1) | 0.01 (0-0.02) | 0 (0-1) | 0.01 (0-0.01) | -0.83 (-2.21-0.56) |
| Democratic People's Republic of Korea | 32 (16-57) | 0.13 (0.06-0.22) | 10 (4-21) | 0.07 (0.03-0.15) | -2.9 (-3.35--2.45) |
| Democratic Republic of the Congo | 641 (345-1081) | 0.75 (0.4-1.26) | 913 (402-1829) | 0.67 (0.29-1.34) | -0.13 (-1.11-0.86) |
| Denmark | 0 (0-0) | 0 (0-0.01) | 0 (0-0) | 0 (0-0) | -3.29 (-4.04--2.52) |
| Djibouti | 14 (8-24) | 1.91 (1.13-3.23) | 16 (8-27) | 1.08 (0.52-1.86) | -2.15 (-2.73--1.57) |
| Dominica | 0 (0-1) | 0.53 (0.27-0.9) | 0 (0-0) | 0.63 (0.27-1.21) | -0.37 (-0.99-0.26) |
| Dominican Republic | 151 (91-237) | 1.41 (0.84-2.2) | 79 (34-136) | 0.77 (0.33-1.33) | -2.3 (-2.64--1.97) |
| Ecuador | 85 (51-126) | 0.59 (0.35-0.87) | 17 (6-31) | 0.11 (0.04-0.2) | -7.33 (-8.01--6.65) |
| Egypt | 84 (37-172) | 0.09 (0.04-0.19) | 48 (24-85) | 0.04 (0.02-0.07) | -2.81 (-3.4--2.21) |
| El Salvador | 66 (41-96) | 0.79 (0.5-1.15) | 6 (3-12) | 0.11 (0.05-0.21) | -7.14 (-7.49--6.78) |
| Equatorial Guinea | 11 (5-20) | 1.08 (0.55-2.06) | 7 (3-13) | 0.4 (0.19-0.7) | -5.08 (-5.96--4.19) |
| Eritrea | 159 (90-262) | 2.24 (1.28-3.7) | 148 (84-233) | 1.58 (0.9-2.49) | -1.54 (-2.27--0.81) |
| Estonia | 1 (0-1) | 0.07 (0.02-0.12) | 0 (0-0) | 0.01 (0-0.03) | -7.7 (-8.67--6.72) |
| Eswatini | 13 (7-22) | 0.86 (0.48-1.43) | 7 (3-13) | 0.54 (0.25-0.93) | -1.88 (-2.5--1.26) |
| Ethiopia | 4886 (3592-6479) | 4.22 (3.11-5.6) | 3782 (2519-5189) | 2.27 (1.51-3.11) | -2.61 (-3.48--1.74) |
| Fiji | 3 (2-5) | 0.32 (0.18-0.52) | 1 (0-2) | 0.12 (0.04-0.23) | -3.74 (-4.15--3.32) |
| Finland | 0 (0-0) | 0.01 (0-0.01) | 0 (0-0) | 0 (0-0.01) | -3.14 (-4.02--2.26) |
| France | 12 (3-23) | 0.03 (0.01-0.06) | 4 (0-9) | 0.01 (0-0.03) | -3.24 (-3.97--2.51) |
| Gabon | 8 (3-14) | 0.44 (0.19-0.84) | 7 (3-13) | 0.35 (0.16-0.62) | -0.67 (-1.26--0.07) |
| Gambia | 74 (47-115) | 3.44 (2.16-5.31) | 76 (45-117) | 2.05 (1.22-3.15) | -2.24 (-3.13--1.34) |
| Georgia | 3 (1-5) | 0.08 (0.03-0.13) | 3 (1-5) | 0.14 (0.06-0.24) | 2.14 (0.86-3.44) |
| Germany | 10 (4-17) | 0.02 (0.01-0.04) | 3 (0-6) | 0.01 (0-0.02) | -4.04 (-5.1--2.96) |
| Ghana | 1054 (670-1585) | 3.65 (2.33-5.49) | 962 (531-1589) | 2.07 (1.14-3.42) | -1.96 (-2.63--1.3) |
| Greece | 1 (0-1) | 0.02 (0.01-0.03) | 0 (0-1) | 0.01 (0-0.01) | -2.47 (-3.59--1.34) |
| Greenland | 0 (0-0) | 0 (0-0.01) | 0 (0-0) | 0 (0-0.01) | -2.5 (-3.37--1.63) |
| Grenada | 0 (0-1) | 0.29 (0.15-0.46) | 0 (0-0) | 0.23 (0.08-0.41) | -1.25 (-1.6--0.89) |
| Guam | 0 (0-0) | 0 (0-0.01) | 0 (0-0) | 0.03 (0-0.07) | 6.6 (5.15-8.06) |
| Guatemala | 171 (112-249) | 1.03 (0.68-1.5) | 68 (41-103) | 0.48 (0.28-0.72) | -3.83 (-4.39--3.27) |
| Guinea | 490 (306-771) | 3.54 (2.21-5.57) | 512 (317-805) | 2.15 (1.33-3.38) | -1.88 (-2.83--0.92) |
| Guinea-Bissau | 98 (60-146) | 4.5 (2.73-6.67) | 80 (46-135) | 2.34 (1.35-3.92) | -2.46 (-3.37--1.54) |
| Guyana | 8 (4-13) | 0.63 (0.35-1.04) | 4 (2-7) | 0.57 (0.25-1.03) | -1.69 (-2.19--1.19) |
| Haiti | 253 (142-403) | 2.1 (1.17-3.35) | 342 (191-547) | 2.09 (1.17-3.34) | -0.6 (-1.18--0.02) |
| Honduras | 101 (59-156) | 1.17 (0.69-1.82) | 65 (33-108) | 0.61 (0.31-1.01) | -2.88 (-3.37--2.4) |
| Hungary | 1 (0-1) | 0.01 (0.01-0.02) | 0 (0-1) | 0.01 (0-0.01) | -2.71 (-4.01--1.4) |
| Iceland | 0 (0-0) | 0.01 (0-0.02) | 0 (0-0) | 0 (0-0) | -4.62 (-5.1--4.14) |
| India | 17311 (14282-21260) | 1.45 (1.19-1.77) | 9485 (6808-12601) | 0.9 (0.65-1.2) | -2.71 (-2.99--2.42) |
| Indonesia | 2683 (1725-4128) | 1.19 (0.77-1.84) | 804 (497-1415) | 0.38 (0.23-0.67) | -4.51 (-4.78--4.23) |
| Iran (Islamic Republic of) | 60 (44-79) | 0.08 (0.06-0.11) | 13 (7-19) | 0.03 (0.01-0.04) | -2.54 (-3.1--1.97) |
| Iraq | 244 (140-375) | 0.74 (0.42-1.13) | 153 (77-260) | 0.38 (0.19-0.65) | -3.17 (-3.73--2.62) |
| Ireland | 0 (0-0) | 0.01 (0-0.01) | 0 (0-0) | 0 (0-0.01) | -2.98 (-3.53--2.43) |
| Israel | 1 (1-2) | 0.03 (0.01-0.04) | 1 (0-2) | 0.01 (0-0.02) | -3.32 (-3.56--3.08) |
| Italy | 10 (8-12) | 0.04 (0.03-0.04) | 3 (2-5) | 0.02 (0.01-0.02) | -2.17 (-3.34--0.98) |
| Jamaica | 13 (7-19) | 0.46 (0.26-0.69) | 5 (2-9) | 0.32 (0.15-0.58) | -1.48 (-2.1--0.86) |
| Japan | 6 (5-7) | 0.01 (0.01-0.01) | 1 (1-2) | 0 (0-0) | -4.95 (-6.14--3.75) |
| Jordan | 19 (9-31) | 0.3 (0.15-0.48) | 19 (9-33) | 0.19 (0.09-0.32) | -2.45 (-2.99--1.91) |
| Kazakhstan | 13 (6-25) | 0.08 (0.03-0.14) | 8 (3-15) | 0.04 (0.02-0.08) | -1.74 (-2.05--1.44) |
| Kenya | 879 (637-1172) | 1.87 (1.35-2.49) | 815 (611-1069) | 1.44 (1.08-1.89) | -1.31 (-2.04--0.58) |
| Kiribati | 0 (0-1) | 0.34 (0.2-0.51) | 0 (0-0) | 0.16 (0.08-0.26) | -3.09 (-3.6--2.58) |
| Kuwait | 2 (1-3) | 0.11 (0.06-0.16) | 1 (1-2) | 0.05 (0.03-0.08) | -2.73 (-3.48--1.98) |
| Kyrgyzstan | 4 (2-6) | 0.06 (0.03-0.1) | 9 (5-13) | 0.11 (0.06-0.17) | 2.3 (1.93-2.68) |
| Lao People's Democratic Republic | 160 (87-271) | 1.94 (1.05-3.26) | 76 (42-124) | 0.9 (0.5-1.47) | -3.63 (-4.25--3.01) |
| Latvia | 1 (1-2) | 0.07 (0.04-0.11) | 0 (0-0) | 0.02 (0-0.03) | -5.48 (-6.5--4.46) |
| Lebanon | 3 (1-6) | 0.07 (0.02-0.14) | 1 (1-3) | 0.03 (0.01-0.07) | -3.09 (-3.39--2.79) |
| Lesotho | 32 (17-57) | 1.26 (0.66-2.24) | 24 (14-38) | 1.19 (0.67-1.88) | -0.66 (-1.09--0.22) |
| Liberia | 191 (112-308) | 3.57 (2.08-5.76) | 153 (87-242) | 1.96 (1.11-3.1) | -2.58 (-3.45--1.69) |
| Libya | 3 (2-6) | 0.05 (0.02-0.09) | 1 (0-5) | 0.04 (0.01-0.12) | -2.64 (-3.03--2.24) |
| Lithuania | 1 (1-2) | 0.05 (0.02-0.08) | 0 (0-0) | 0.01 (0-0.03) | -5.1 (-6.04--4.16) |
| Luxembourg | 0 (0-0) | 0.01 (0-0.02) | 0 (0-0) | 0 (0-0.01) | -5.05 (-5.81--4.28) |
| Madagascar | 482 (309-725) | 1.93 (1.24-2.9) | 688 (420-1089) | 1.7 (1.04-2.69) | -0.68 (-1.55-0.2) |
| Malawi | 651 (396-972) | 2.73 (1.66-4.07) | 493 (317-735) | 1.81 (1.16-2.69) | -2.19 (-3.16--1.22) |
| Malaysia | 70 (34-121) | 0.29 (0.14-0.5) | 18 (6-32) | 0.08 (0.03-0.14) | -5.42 (-5.87--4.96) |
| Maldives | 4 (2-6) | 0.9 (0.46-1.44) | 0 (0-0) | 0.08 (0.02-0.16) | -10.23 (-10.64--9.81) |
| Mali | 1023 (618-1634) | 4.82 (2.92-7.66) | 1144 (684-1765) | 2.25 (1.34-3.46) | -2.72 (-3.83--1.6) |
| Malta | 0 (0-0) | 0.01 (0-0.02) | 0 (0-0) | 0 (0-0.01) | -2.61 (-3.51--1.71) |
| Marshall Islands | 0 (0-0) | 0.15 (0.07-0.27) | 0 (0-0) | 0.11 (0.05-0.2) | -1.75 (-2.23--1.27) |
| Mauritania | 137 (85-203) | 3.32 (2.07-4.91) | 102 (57-163) | 1.57 (0.87-2.52) | -2.78 (-3.58--1.97) |
| Mauritius | 1 (0-2) | 0.1 (0.02-0.18) | 0 (0-1) | 0.06 (0.02-0.13) | -2.29 (-2.99--1.58) |
| Mexico | 476 (395-552) | 0.39 (0.32-0.45) | 185 (139-235) | 0.2 (0.15-0.26) | -3.56 (-3.93--3.2) |
| Micronesia (Federated States of) | 0 (0-1) | 0.28 (0.14-0.48) | 0 (0-0) | 0.09 (0.04-0.19) | -4.69 (-4.99--4.39) |
| Monaco | 0 (0-0) | 0.01 (0-0.02) | 0 (0-0) | 0.01 (0-0.01) | -2.11 (-3.35--0.86) |
| Mongolia | 6 (3-12) | 0.18 (0.08-0.34) | 5 (2-8) | 0.12 (0.06-0.22) | -1.69 (-1.99--1.39) |
| Montenegro | 0 (0-1) | 0.09 (0.04-0.16) | 0 (0-0) | 0.02 (0.01-0.04) | -6.12 (-6.86--5.39) |
| Morocco | 38 (17-65) | 0.1 (0.05-0.17) | 14 (6-24) | 0.04 (0.02-0.08) | -3.13 (-3.62--2.64) |
| Mozambique | 1232 (741-1787) | 4.24 (2.55-6.15) | 1349 (783-2137) | 2.55 (1.48-4.04) | -1.97 (-2.94--1) |
| Myanmar | 1441 (852-2198) | 2.7 (1.6-4.11) | 720 (409-1205) | 1.4 (0.79-2.34) | -2.84 (-3.03--2.66) |
| Namibia | 26 (16-42) | 1.05 (0.63-1.71) | 15 (7-27) | 0.54 (0.26-0.96) | -2.32 (-2.83--1.81) |
| Nauru | 0 (0-0) | 0.03 (0-0.09) | 0 (0-0) | 0.02 (0-0.07) | -2.45 (-3.33--1.55) |
| Nepal | 539 (311-848) | 1.44 (0.83-2.26) | 217 (119-363) | 0.7 (0.39-1.18) | -3.37 (-3.84--2.89) |
| Netherlands | 4 (1-7) | 0.04 (0.02-0.07) | 2 (0-4) | 0.02 (0-0.04) | -2.82 (-3.6--2.03) |
| New Zealand | 0 (0-0) | 0.01 (0-0.01) | 0 (0-0) | 0.01 (0-0.01) | 1.81 (0.8-2.83) |
| Nicaragua | 98 (65-142) | 1.44 (0.95-2.07) | 31 (16-49) | 0.5 (0.26-0.8) | -4.19 (-4.57--3.81) |
| Niger | 795 (486-1252) | 3.74 (2.29-5.89) | 1362 (743-2320) | 2.41 (1.31-4.1) | -2.05 (-3.26--0.82) |
| Nigeria | 5527 (4277-7028) | 2.84 (2.2-3.61) | 8771 (6609-11344) | 2.22 (1.67-2.88) | -1.04 (-2.01--0.06) |
| Niue | 0 (0-0) | 0.07 (0.03-0.13) | 0 (0-0) | 0.06 (0-0.17) | -4.14 (-5--3.26) |
| North Macedonia | 0 (0-0) | 0.01 (0-0.02) | 0 (0-0) | 0.01 (0-0.02) | -0.11 (-1.2-0.99) |
| Northern Mariana Islands | 0 (0-0) | 0.01 (0-0.03) | 0 (0-0) | 0 (0-0.01) | -3.78 (-4.41--3.15) |
| Norway | 0 (0-0) | 0.01 (0-0.01) | 0 (0-0) | 0 (0-0) | -4.93 (-6.39--3.45) |
| Oman | 4 (2-7) | 0.13 (0.06-0.22) | 2 (1-4) | 0.06 (0.03-0.1) | -2.78 (-3.26--2.3) |
| Pakistan | 2594 (1742-3817) | 1.29 (0.87-1.9) | 2962 (2039-4337) | 0.99 (0.69-1.46) | -1.63 (-2.22--1.03) |
| Palau | 0 (0-0) | 0.01 (0-0.03) | 0 (0-0) | 0.01 (0-0.02) | -2.15 (-2.7--1.6) |
| Palestine | 7 (3-12) | 0.17 (0.08-0.29) | 6 (3-11) | 0.11 (0.05-0.19) | -2.21 (-2.94--1.46) |
| Panama | 11 (6-17) | 0.38 (0.2-0.6) | 4 (1-7) | 0.1 (0.03-0.2) | -4.77 (-5.01--4.52) |
| Papua New Guinea | 31 (17-58) | 0.44 (0.23-0.82) | 71 (36-123) | 0.43 (0.22-0.75) | -0.25 (-0.93-0.44) |
| Paraguay | 67 (41-101) | 1.08 (0.66-1.63) | 8 (3-15) | 0.13 (0.05-0.25) | -8.34 (-8.87--7.81) |
| Peru | 570 (356-860) | 1.85 (1.15-2.79) | 152 (73-267) | 0.47 (0.23-0.83) | -3.99 (-4.37--3.6) |
| Philippines | 1637 (1308-2006) | 1.69 (1.35-2.07) | 964 (731-1225) | 0.88 (0.67-1.12) | -2.57 (-2.99--2.13) |
| Poland | 32 (25-43) | 0.12 (0.09-0.16) | 1 (1-2) | 0.01 (0.01-0.01) | -10.36 (-11.39--9.31) |
| Portugal | 2 (1-4) | 0.04 (0.01-0.08) | 0 (0-1) | 0.01 (0-0.02) | -5.35 (-6.37--4.32) |
| Puerto Rico | 1 (0-2) | 0.04 (0.01-0.08) | 0 (0-1) | 0.03 (0-0.08) | -1.72 (-2.83--0.59) |
| Qatar | 0 (0-0) | 0.03 (0.02-0.05) | 0 (0-0) | 0.01 (0.01-0.02) | -4.85 (-5.24--4.46) |
| Republic of Korea | 21 (9-37) | 0.06 (0.03-0.11) | 2 (1-4) | 0.02 (0.01-0.03) | -5.61 (-6.67--4.53) |
| Republic of Moldova | 20 (12-29) | 0.51 (0.31-0.75) | 3 (1-6) | 0.22 (0.09-0.41) | -4.37 (-5.34--3.4) |
| Romania | 3 (2-5) | 0.02 (0.01-0.03) | 0 (0-1) | 0 (0-0.01) | -7.11 (-8.23--5.97) |
| Russian Federation | 79 (67-89) | 0.08 (0.07-0.09) | 27 (22-33) | 0.04 (0.03-0.05) | -3.11 (-4.11--2.11) |
| Rwanda | 490 (312-715) | 3.23 (2.05-4.71) | 248 (143-381) | 1.4 (0.81-2.15) | -3.28 (-4.06--2.49) |
| Saint Kitts and Nevis | 0 (0-0) | 0.03 (0.01-0.07) | 0 (0-0) | 0.03 (0-0.06) | -1.91 (-2.49--1.32) |
| Saint Lucia | 0 (0-1) | 0.27 (0.16-0.44) | 0 (0-0) | 0.25 (0.1-0.45) | -1.36 (-2--0.71) |
| Saint Vincent and the Grenadines | 1 (0-1) | 0.42 (0.24-0.65) | 0 (0-0) | 0.31 (0.13-0.53) | -1.68 (-2.14--1.23) |
| Samoa | 0 (0-1) | 0.14 (0.07-0.26) | 0 (0-0) | 0.07 (0.03-0.14) | -1.95 (-2.46--1.44) |
| San Marino | 0 (0-0) | 0.03 (0.01-0.07) | 0 (0-0) | 0.01 (0-0.02) | -5.1 (-6.1--4.08) |
| Sao Tome and Principe | 4 (2-6) | 1.8 (1.06-2.88) | 1 (1-3) | 0.62 (0.29-1.12) | -4.18 (-4.92--3.43) |
| Saudi Arabia | 87 (41-156) | 0.36 (0.17-0.64) | 20 (10-34) | 0.09 (0.04-0.15) | -6.23 (-6.74--5.72) |
| Senegal | 516 (316-756) | 3.13 (1.92-4.59) | 419 (252-654) | 1.82 (1.09-2.84) | -2.03 (-2.85--1.2) |
| Serbia | 6 (3-11) | 0.1 (0.05-0.17) | 1 (0-1) | 0.02 (0.01-0.03) | -7.8 (-8.89--6.69) |
| Seychelles | 0 (0-0) | 0.12 (0.02-0.26) | 0 (0-0) | 0.09 (0.01-0.2) | -1.78 (-2.12--1.45) |
| Sierra Leone | 432 (248-684) | 4.47 (2.57-7.07) | 392 (244-623) | 2.71 (1.69-4.31) | -2.01 (-2.95--1.06) |
| Singapore | 0 (0-0) | 0.01 (0.01-0.02) | 0 (0-0) | 0 (0-0.01) | -4.61 (-5.49--3.72) |
| Slovakia | 0 (0-0) | 0.01 (0-0.01) | 0 (0-0) | 0 (0-0.01) | -3.09 (-4.06--2.12) |
| Slovenia | 0 (0-0) | 0 (0-0.01) | 0 (0-0) | 0 (0-0.01) | 0.08 (-1.43-1.61) |
| Solomon Islands | 3 (1-5) | 0.41 (0.22-0.69) | 3 (1-5) | 0.29 (0.14-0.5) | -1.64 (-2.38--0.9) |
| Somalia | 537 (280-848) | 2.91 (1.52-4.6) | 1167 (547-1991) | 2.54 (1.19-4.33) | -0.44 (-1.56-0.68) |
| South Africa | 305 (216-406) | 0.61 (0.43-0.81) | 220 (154-303) | 0.47 (0.33-0.64) | -1.6 (-1.83--1.37) |
| South Sudan | 335 (184-553) | 2.75 (1.51-4.52) | 442 (217-858) | 2.42 (1.19-4.7) | -0.34 (-1.31-0.64) |
| Spain | 8 (3-12) | 0.04 (0.02-0.06) | 2 (0-4) | 0.01 (0-0.02) | -3.92 (-5.01--2.83) |
| Sri Lanka | 114 (77-161) | 0.66 (0.45-0.93) | 21 (11-36) | 0.15 (0.07-0.25) | -5.58 (-6.03--5.13) |
| Sudan | 100 (53-167) | 0.24 (0.13-0.4) | 72 (36-124) | 0.13 (0.06-0.22) | -2.59 (-3.37--1.81) |
| Suriname | 4 (2-6) | 0.87 (0.44-1.45) | 3 (1-5) | 0.61 (0.28-1.06) | -1.84 (-2.05--1.62) |
| Sweden | 1 (0-2) | 0.01 (0-0.03) | 0 (0-0) | 0 (0-0.01) | -4.63 (-5.52--3.74) |
| Switzerland | 1 (0-2) | 0.02 (0.01-0.04) | 0 (0-1) | 0.01 (0-0.02) | -3.36 (-4.28--2.43) |
| Syrian Arab Republic | 28 (12-53) | 0.13 (0.06-0.24) | 4 (2-9) | 0.04 (0.02-0.09) | -4.28 (-4.85--3.7) |
| Taiwan (Province of China) | 0 (0-1) | 0 (0-0) | 2 (0-3) | 0.02 (0.01-0.04) | 7.59 (6.3-8.89) |
| Tajikistan | 20 (9-38) | 0.2 (0.09-0.38) | 20 (9-37) | 0.15 (0.07-0.27) | -1.48 (-2.05--0.91) |
| Thailand | 354 (186-601) | 0.71 (0.37-1.21) | 32 (16-52) | 0.12 (0.06-0.2) | -6.74 (-7.48--5.99) |
| Timor-Leste | 32 (18-50) | 1.91 (1.09-3.02) | 18 (10-29) | 0.93 (0.53-1.47) | -3.68 (-4.41--2.95) |
| Togo | 228 (143-354) | 2.98 (1.87-4.62) | 198 (115-324) | 1.7 (0.99-2.78) | -2.25 (-3.06--1.43) |
| Tokelau | 0 (0-0) | 0.04 (0-0.11) | 0 (0-0) | 0.14 (0-0.43) | -1.4 (-2.59--0.19) |
| Tonga | 0 (0-0) | 0.17 (0.07-0.31) | 0 (0-0) | 0.08 (0.03-0.15) | -2.64 (-3.18--2.1) |
| Trinidad and Tobago | 4 (1-7) | 0.32 (0.12-0.6) | 2 (1-4) | 0.3 (0.11-0.57) | -0.8 (-1.38--0.23) |
| Tunisia | 6 (3-12) | 0.06 (0.03-0.11) | 2 (1-5) | 0.03 (0.01-0.06) | -2.93 (-3.22--2.63) |
| T眉rkiye | 259 (133-427) | 0.37 (0.19-0.6) | 50 (20-91) | 0.1 (0.04-0.19) | -5.06 (-5.37--4.75) |
| Turkmenistan | 4 (1-7) | 0.06 (0.02-0.12) | 4 (1-6) | 0.07 (0.03-0.12) | 0.44 (-0.03-0.91) |
| Tuvalu | 0 (0-0) | 0.15 (0.06-0.32) | 0 (0-0) | 0.03 (0-0.06) | -6.93 (-7.28--6.59) |
| Uganda | 1048 (684-1570) | 2.38 (1.56-3.57) | 1330 (847-2113) | 1.76 (1.12-2.79) | -1.4 (-2.48--0.31) |
| Ukraine | 28 (13-46) | 0.09 (0.04-0.14) | 7 (1-13) | 0.05 (0.01-0.1) | -2.44 (-3.54--1.33) |
| United Arab Emirates | 0 (0-1) | 0.02 (0.01-0.05) | 0 (0-1) | 0.01 (0-0.02) | -2.25 (-3.05--1.45) |
| United Kingdom | 5 (5-6) | 0.01 (0.01-0.02) | 1 (1-1) | 0 (0-0) | -6.33 (-7.09--5.56) |
| United Republic of Tanzania | 1486 (917-2268) | 2.59 (1.6-3.94) | 2026 (1168-3256) | 2.24 (1.29-3.61) | -0.5 (-1.42-0.44) |
| United States of America | 47 (39-55) | 0.02 (0.02-0.03) | 17 (12-22) | 0.01 (0.01-0.01) | -3.73 (-4.38--3.08) |
| United States Virgin Islands | 0 (0-0) | 0.03 (0.01-0.07) | 0 (0-0) | 0.02 (0-0.04) | -2.7 (-3.3--2.09) |
| Uruguay | 4 (1-7) | 0.15 (0.05-0.25) | 1 (0-1) | 0.04 (0.01-0.09) | -5.44 (-5.99--4.9) |
| Uzbekistan | 33 (19-59) | 0.1 (0.06-0.17) | 71 (40-106) | 0.19 (0.1-0.28) | 2.29 (1.87-2.7) |
| Vanuatu | 1 (0-1) | 0.27 (0.14-0.43) | 1 (0-1) | 0.2 (0.11-0.35) | -1.57 (-2.29--0.85) |
| Venezuela (Bolivarian Republic of) | 105 (47-175) | 0.4 (0.18-0.66) | 39 (13-73) | 0.18 (0.06-0.34) | -2.77 (-3.11--2.43) |
| Viet Nam | 1132 (688-1883) | 1.22 (0.74-2.03) | 216 (109-353) | 0.29 (0.15-0.47) | -5.76 (-5.98--5.54) |
| Yemen | 86 (50-137) | 0.27 (0.16-0.44) | 77 (39-129) | 0.17 (0.08-0.28) | -2.25 (-3.15--1.34) |
| Zambia | 374 (224-548) | 2.05 (1.23-3) | 481 (270-766) | 1.66 (0.93-2.64) | -1.08 (-2.06--0.08) |
| Zimbabwe | 200 (116-305) | 1.07 (0.63-1.64) | 258 (157-397) | 1.15 (0.7-1.77) | 0.91 (0.18-1.65) |
| **Particulate matter pollution** |  |  |  |  |  |
| **region_eapc** |  |  |  |  |  |
| Advanced Health System | 674 (523-865) | 0.07 (0.06-0.1) | 160 (120-213) | 0.02 (0.02-0.03) | -4.39 (-5.14--3.63) |
| Africa | 28559 (24134-33103) | 2.23 (1.89-2.59) | 34020 (27605-41543) | 1.65 (1.34-2.01) | -1.35 (-2.15--0.54) |
| African Region | 27777 (23640-32090) | 2.59 (2.2-2.99) | 32700 (26482-39710) | 1.82 (1.48-2.21) | -1.47 (-2.32--0.61) |
| America | 3747 (3260-4269) | 0.49 (0.43-0.56) | 1327 (1002-1702) | 0.19 (0.15-0.25) | -3.64 (-3.93--3.34) |
| Andean Latin America | 806 (564-1118) | 1.43 (1-1.99) | 220 (130-352) | 0.37 (0.22-0.59) | -4.47 (-4.8--4.13) |
| Asia | 37715 (31875-44362) | 0.99 (0.84-1.16) | 18517 (14907-22603) | 0.61 (0.49-0.74) | -2.74 (-3--2.49) |
| Australasia | 2 (0-4) | 0.01 (0-0.03) | 1 (0-2) | 0.01 (0-0.01) | -2.85 (-3.69--2.01) |
| Basic Health System | 11255 (9335-13441) | 0.44 (0.36-0.52) | 3577 (2905-4433) | 0.19 (0.15-0.24) | -3.73 (-4.1--3.36) |
| Caribbean | 462 (328-650) | 1.07 (0.76-1.51) | 455 (276-688) | 1.19 (0.72-1.8) | -0.19 (-0.37-0) |
| Central Africa | 2150 (1598-2847) | 1.44 (1.07-1.9) | 3315 (2264-4643) | 1.23 (0.84-1.72) | -0.67 (-1.62-0.28) |
| Central Asia | 123 (90-173) | 0.13 (0.1-0.18) | 134 (98-176) | 0.14 (0.1-0.18) | -0.07 (-0.28-0.14) |
| Central Europe | 48 (36-62) | 0.06 (0.04-0.07) | 4 (3-5) | 0.01 (0.01-0.01) | -7.63 (-8.63--6.62) |
| Central Latin America | 1131 (978-1295) | 0.47 (0.41-0.54) | 441 (323-575) | 0.23 (0.17-0.31) | -3.34 (-3.67--3.01) |
| Central Sub-Saharan Africa | 1132 (721-1716) | 0.92 (0.59-1.4) | 1349 (728-2349) | 0.63 (0.34-1.1) | -1.31 (-2.27--0.34) |
| Commonwealth High Income | 15 (11-19) | 0.02 (0.01-0.02) | 6 (4-9) | 0.01 (0.01-0.01) | -3.15 (-3.84--2.44) |
| Commonwealth Low Income | 13355 (10210-17436) | 3.11 (2.38-4.06) | 8672 (6976-11009) | 1.85 (1.49-2.35) | -2.29 (-2.98--1.6) |
| Commonwealth Middle Income | 28046 (23989-32733) | 1.58 (1.35-1.84) | 23804 (19522-28666) | 1.2 (0.98-1.45) | -1.68 (-2.03--1.33) |
| East Asia | 1382 (1061-1808) | 0.12 (0.09-0.16) | 135 (96-175) | 0.02 (0.02-0.03) | -6.02 (-6.76--5.26) |
| East Asia & Pacific - WB | 9311 (7270-11778) | 0.5 (0.39-0.64) | 3204 (2493-4231) | 0.27 (0.21-0.36) | -3.31 (-3.86--2.77) |
| Eastern Africa | 10447 (8420-12948) | 2.7 (2.18-3.35) | 10751 (8040-13629) | 1.86 (1.39-2.35) | -1.65 (-2.52--0.78) |
| Eastern Europe | 137 (112-163) | 0.1 (0.08-0.11) | 38 (30-47) | 0.04 (0.04-0.05) | -3.3 (-4.27--2.31) |
| Eastern Mediterranean Region | 3995 (2944-5365) | 0.63 (0.46-0.84) | 4775 (3542-6361) | 0.58 (0.43-0.77) | -1.16 (-1.64--0.67) |
| Eastern Sub-Saharan Africa | 12963 (10860-15508) | 3.03 (2.54-3.62) | 13474 (10169-17013) | 2.06 (1.55-2.6) | -1.69 (-2.59--0.78) |
| Europe | 543 (409-711) | 0.1 (0.08-0.13) | 126 (92-171) | 0.03 (0.02-0.04) | -4.19 (-5--3.38) |
| Europe & Central Asia - WB | 617 (472-783) | 0.1 (0.08-0.13) | 238 (185-301) | 0.05 (0.04-0.06) | -2.8 (-3.51--2.08) |
| European Region | 619 (474-785) | 0.1 (0.08-0.13) | 239 (186-302) | 0.05 (0.04-0.06) | -2.81 (-3.51--2.1) |
| High-income Asia Pacific | 27 (15-43) | 0.03 (0.02-0.05) | 4 (3-6) | 0.01 (0-0.01) | -5.61 (-6.71--4.49) |
| High-income North America | 48 (40-57) | 0.02 (0.02-0.03) | 18 (13-23) | 0.01 (0.01-0.01) | -3.68 (-4.34--3.01) |
| Latin America & Caribbean - WB | 3700 (3211-4221) | 0.67 (0.58-0.77) | 1310 (984-1682) | 0.27 (0.2-0.35) | -3.69 (-3.93--3.46) |
| Limited Health System | 50750 (43720-58870) | 1.95 (1.68-2.26) | 39249 (32655-46673) | 1.32 (1.09-1.56) | -1.99 (-2.44--1.54) |
| Middle East & North Africa - WB | 704 (521-939) | 0.18 (0.13-0.24) | 390 (270-516) | 0.09 (0.06-0.12) | -2.88 (-3.24--2.52) |
| Minimal Health System | 7886 (6219-9768) | 2.55 (2.01-3.16) | 11004 (8400-14292) | 1.79 (1.37-2.33) | -1.35 (-2.36--0.34) |
| North Africa and Middle East | 1127 (866-1438) | 0.22 (0.17-0.27) | 692 (497-924) | 0.12 (0.09-0.16) | -2.68 (-3.03--2.33) |
| North America | 48 (40-57) | 0.02 (0.02-0.03) | 18 (13-23) | 0.01 (0.01-0.01) | -3.68 (-4.34--3.01) |
| Northern Africa | 285 (192-399) | 0.15 (0.1-0.21) | 178 (120-252) | 0.08 (0.06-0.12) | -2.51 (-2.91--2.1) |
| Oceania | 42 (25-70) | 0.39 (0.23-0.65) | 80 (43-133) | 0.39 (0.21-0.65) | -0.2 (-0.81-0.41) |
| Region of the Americas | 3747 (3260-4269) | 0.49 (0.43-0.56) | 1327 (1002-1702) | 0.19 (0.15-0.25) | -3.64 (-3.93--3.34) |
| South-East Asia Region | 29656 (24936-35466) | 1.64 (1.38-1.96) | 13330 (10327-16717) | 0.87 (0.67-1.09) | -3.13 (-3.39--2.86) |
| South Asia | 27591 (23091-33460) | 1.68 (1.41-2.04) | 14685 (11591-18401) | 0.97 (0.77-1.22) | -2.86 (-3.17--2.55) |
| South Asia - WB | 27788 (23252-33643) | 1.66 (1.39-2) | 14903 (11771-18616) | 0.94 (0.74-1.17) | -2.87 (-3.18--2.56) |
| Southeast Asia | 7985 (6123-10483) | 1.35 (1.03-1.77) | 3009 (2305-4007) | 0.56 (0.43-0.74) | -3.65 (-3.89--3.42) |
| Southern Africa | 3215 (2528-3995) | 1.86 (1.46-2.31) | 3123 (2294-4229) | 1.27 (0.94-1.72) | -1.62 (-2.29--0.95) |
| Southern Latin America | 94 (54-138) | 0.18 (0.11-0.27) | 22 (10-34) | 0.06 (0.03-0.09) | -5.14 (-5.52--4.75) |
| Southern Sub-Saharan Africa | 599 (443-760) | 0.77 (0.57-0.98) | 536 (399-746) | 0.69 (0.51-0.96) | -0.6 (-0.93--0.27) |
| Sub-Saharan Africa - WB | 28398 (24049-32957) | 2.59 (2.19-3.01) | 33928 (27523-41437) | 1.83 (1.49-2.24) | -1.45 (-2.33--0.57) |
| Tropical Latin America | 1223 (1022-1467) | 0.76 (0.63-0.91) | 188 (135-247) | 0.11 (0.08-0.15) | -6.43 (-6.85--6.01) |
| Western Africa | 12462 (9981-15007) | 3.25 (2.6-3.91) | 16654 (13705-20224) | 2.21 (1.82-2.69) | -1.5 (-2.45--0.54) |
| Western Europe | 58 (45-74) | 0.03 (0.02-0.03) | 17 (11-24) | 0.01 (0.01-0.01) | -3.59 (-4.45--2.71) |
| Western Pacific Region | 4769 (3901-5841) | 0.32 (0.26-0.4) | 1617 (1300-1973) | 0.19 (0.15-0.23) | -3.11 (-3.73--2.48) |
| Western Sub-Saharan Africa | 13627 (11063-16278) | 3.2 (2.6-3.82) | 18524 (15196-22413) | 2.18 (1.79-2.64) | -1.51 (-2.46--0.54) |
| **country_eapc** |  |  |  |  |  |
| Afghanistan | 79 (42-130) | 0.37 (0.2-0.62) | 196 (107-331) | 0.34 (0.18-0.57) | -0.88 (-1.9-0.16) |
| Albania | 0 (0-0) | 0 (0-0.01) | 0 (0-0) | 0 (0-0.01) | -2.54 (-3.53--1.53) |
| Algeria | 16 (6-33) | 0.04 (0.02-0.09) | 11 (3-30) | 0.02 (0.01-0.07) | -2.42 (-2.68--2.17) |
| American Samoa | 0 (0-0) | 0.02 (0-0.05) | 0 (0-0) | 0.01 (0-0.02) | -5.86 (-6.52--5.2) |
| Andorra | 0 (0-0) | 0.01 (0-0.03) | 0 (0-0) | 0 (0-0.01) | -6.02 (-7.32--4.71) |
| Angola | 359 (202-583) | 1.5 (0.85-2.44) | 263 (144-451) | 0.45 (0.25-0.78) | -4.51 (-5.53--3.47) |
| Antigua and Barbuda | 0 (0-0) | 0.08 (0.02-0.14) | 0 (0-0) | 0.09 (0.03-0.16) | -0.54 (-1.02--0.06) |
| Argentina | 72 (35-116) | 0.22 (0.11-0.35) | 16 (6-28) | 0.06 (0.02-0.11) | -5.51 (-5.86--5.15) |
| Armenia | 24 (13-38) | 0.65 (0.35-1.05) | 7 (4-11) | 0.41 (0.22-0.64) | -2.8 (-3.38--2.22) |
| Australia | 2 (0-4) | 0.01 (0-0.03) | 1 (0-2) | 0.01 (0-0.01) | -3.65 (-4.57--2.72) |
| Austria | 1 (0-1) | 0.01 (0.01-0.02) | 0 (0-0) | 0 (0-0.01) | -3.71 (-4.69--2.72) |
| Azerbaijan | 16 (7-30) | 0.18 (0.08-0.33) | 7 (3-16) | 0.12 (0.05-0.24) | -2.6 (-2.99--2.21) |
| Bahamas | 1 (0-1) | 0.21 (0.09-0.37) | 0 (0-1) | 0.17 (0.07-0.31) | -1.65 (-2.24--1.06) |
| Bahrain | 1 (0-1) | 0.11 (0.06-0.17) | 0 (0-0) | 0.03 (0.02-0.05) | -4.88 (-5.29--4.47) |
| Bangladesh | 7136 (4365-11015) | 3.53 (2.16-5.42) | 2020 (1109-3277) | 1.5 (0.83-2.44) | -3.87 (-4.33--3.41) |
| Barbados | 0 (0-1) | 0.23 (0.11-0.37) | 0 (0-1) | 0.3 (0.13-0.56) | 0.41 (-0.38-1.21) |
| Belarus | 8 (4-15) | 0.12 (0.06-0.22) | 2 (0-3) | 0.04 (0.01-0.08) | -3.77 (-4.71--2.82) |
| Belgium | 2 (1-3) | 0.03 (0.01-0.05) | 1 (0-1) | 0.01 (0-0.02) | -4 (-4.81--3.19) |
| Belize | 1 (1-2) | 0.44 (0.24-0.68) | 1 (1-2) | 0.3 (0.15-0.47) | -1.88 (-2.33--1.42) |
| Benin | 379 (223-572) | 3.34 (1.96-5.04) | 541 (336-852) | 2.16 (1.34-3.4) | -1.58 (-2.58--0.56) |
| Bermuda | 0 (0-0) | 0.01 (0-0.03) | 0 (0-0) | 0 (0-0.01) | -4.75 (-5.6--3.89) |
| Bhutan | 12 (7-19) | 1.11 (0.67-1.8) | 1 (1-2) | 0.23 (0.11-0.42) | -6.29 (-6.65--5.93) |
| Bolivia (Plurinational State of) | 150 (88-239) | 1.37 (0.81-2.19) | 52 (25-92) | 0.44 (0.21-0.78) | -4.58 (-4.96--4.19) |
| Bosnia and Herzegovina | 2 (1-6) | 0.07 (0.02-0.17) | 0 (0-1) | 0.03 (0.01-0.05) | -4.44 (-5.43--3.44) |
| Botswana | 22 (14-35) | 1 (0.62-1.56) | 11 (5-20) | 0.49 (0.23-0.86) | -2.77 (-3.19--2.36) |
| Brazil | 1156 (959-1394) | 0.75 (0.62-0.9) | 180 (129-239) | 0.11 (0.08-0.15) | -6.34 (-6.76--5.91) |
| Brunei Darussalam | 0 (0-0) | 0.04 (0.01-0.08) | 0 (0-0) | 0.04 (0.01-0.09) | -1.07 (-1.54--0.59) |
| Bulgaria | 0 (0-0) | 0.01 (0-0.01) | 0 (0-0) | 0.01 (0-0.02) | 0.91 (-0.94-2.79) |
| Burkina Faso | 768 (455-1217) | 3.48 (2.06-5.5) | 1147 (690-1796) | 2.52 (1.52-3.95) | -1.09 (-2.14--0.03) |
| Burundi | 350 (217-537) | 2.82 (1.74-4.32) | 461 (278-745) | 2.06 (1.24-3.33) | -1.18 (-2.12--0.24) |
| Cabo Verde | 6 (3-10) | 1.06 (0.57-1.71) | 2 (1-4) | 0.55 (0.31-0.96) | -3.44 (-3.87--3.02) |
| Cambodia | 347 (191-548) | 1.69 (0.93-2.68) | 134 (75-216) | 0.78 (0.44-1.26) | -3.99 (-4.45--3.54) |
| Cameroon | 535 (330-822) | 2.35 (1.45-3.6) | 738 (440-1152) | 1.48 (0.88-2.32) | -1.84 (-2.73--0.95) |
| Canada | 2 (0-4) | 0.01 (0-0.02) | 1 (0-2) | 0.01 (0-0.01) | -2.32 (-3.12--1.51) |
| Central African Republic | 79 (47-136) | 1.31 (0.79-2.26) | 120 (66-208) | 1.32 (0.73-2.29) | -0.17 (-1.11-0.79) |
| Chad | 488 (288-734) | 3.2 (1.89-4.8) | 1029 (603-1642) | 2.49 (1.46-3.97) | -0.92 (-2.12-0.29) |
| Chile | 18 (10-27) | 0.12 (0.07-0.18) | 5 (2-8) | 0.05 (0.02-0.08) | -3.58 (-4.23--2.92) |
| China | 1349 (1031-1756) | 0.12 (0.09-0.16) | 124 (88-161) | 0.02 (0.02-0.03) | -6.19 (-6.94--5.43) |
| Colombia | 99 (57-148) | 0.22 (0.13-0.33) | 42 (16-76) | 0.13 (0.05-0.23) | -2.33 (-3.02--1.65) |
| Comoros | 29 (17-44) | 3.08 (1.82-4.72) | 16 (10-26) | 1.99 (1.18-3.17) | -2.36 (-2.95--1.75) |
| Congo | 35 (16-63) | 0.79 (0.36-1.44) | 38 (17-66) | 0.62 (0.28-1.08) | -1.3 (-2.03--0.56) |
| Cook Islands | 0 (0-0) | 0.03 (0-0.08) | 0 (0-0) | 0.01 (0-0.03) | -6.15 (-6.72--5.58) |
| Costa Rica | 4 (2-6) | 0.09 (0.05-0.15) | 1 (0-2) | 0.03 (0.01-0.06) | -5.13 (-5.55--4.7) |
| Croatia | 0 (0-1) | 0.01 (0.01-0.02) | 0 (0-1) | 0.02 (0.01-0.04) | -0.5 (-2.17-1.2) |
| Cuba | 9 (3-15) | 0.1 (0.04-0.17) | 2 (1-4) | 0.04 (0.01-0.07) | -3.58 (-4.37--2.79) |
| Cyprus | 0 (0-1) | 0.04 (0.01-0.08) | 0 (0-0) | 0.01 (0-0.02) | -5.98 (-6.64--5.32) |
| Czechia | 1 (0-1) | 0.01 (0-0.02) | 0 (0-1) | 0.01 (0-0.01) | -0.83 (-2.21-0.56) |
| Democratic People's Republic of Korea | 32 (16-57) | 0.13 (0.06-0.22) | 10 (4-21) | 0.07 (0.03-0.15) | -2.9 (-3.35--2.45) |
| Democratic Republic of the Congo | 641 (345-1081) | 0.75 (0.4-1.26) | 913 (402-1829) | 0.67 (0.29-1.34) | -0.13 (-1.11-0.86) |
| Denmark | 0 (0-0) | 0 (0-0.01) | 0 (0-0) | 0 (0-0) | -3.29 (-4.04--2.52) |
| Djibouti | 14 (8-24) | 1.91 (1.13-3.23) | 16 (8-27) | 1.08 (0.52-1.86) | -2.15 (-2.73--1.57) |
| Dominica | 0 (0-1) | 0.53 (0.27-0.9) | 0 (0-0) | 0.63 (0.27-1.21) | -0.37 (-0.99-0.26) |
| Dominican Republic | 151 (91-237) | 1.41 (0.84-2.2) | 79 (34-136) | 0.77 (0.33-1.33) | -2.3 (-2.64--1.97) |
| Ecuador | 85 (51-126) | 0.59 (0.35-0.87) | 17 (6-31) | 0.11 (0.04-0.2) | -7.33 (-8.01--6.65) |
| Egypt | 84 (37-172) | 0.09 (0.04-0.19) | 48 (24-85) | 0.04 (0.02-0.07) | -2.81 (-3.4--2.21) |
| El Salvador | 66 (41-96) | 0.79 (0.5-1.15) | 6 (3-12) | 0.11 (0.05-0.21) | -7.14 (-7.49--6.78) |
| Equatorial Guinea | 11 (5-20) | 1.08 (0.55-2.06) | 7 (3-13) | 0.4 (0.19-0.7) | -5.08 (-5.96--4.19) |
| Eritrea | 159 (90-262) | 2.24 (1.28-3.7) | 148 (84-233) | 1.58 (0.9-2.49) | -1.54 (-2.27--0.81) |
| Estonia | 1 (0-1) | 0.07 (0.02-0.12) | 0 (0-0) | 0.01 (0-0.03) | -7.7 (-8.67--6.72) |
| Eswatini | 13 (7-22) | 0.86 (0.48-1.43) | 7 (3-13) | 0.54 (0.25-0.93) | -1.88 (-2.5--1.26) |
| Ethiopia | 4886 (3592-6479) | 4.22 (3.11-5.6) | 3782 (2519-5189) | 2.27 (1.51-3.11) | -2.61 (-3.48--1.74) |
| Fiji | 3 (2-5) | 0.32 (0.18-0.52) | 1 (0-2) | 0.12 (0.04-0.23) | -3.74 (-4.15--3.32) |
| Finland | 0 (0-0) | 0.01 (0-0.01) | 0 (0-0) | 0 (0-0.01) | -3.14 (-4.02--2.26) |
| France | 12 (3-23) | 0.03 (0.01-0.06) | 4 (0-9) | 0.01 (0-0.03) | -3.24 (-3.97--2.51) |
| Gabon | 8 (3-14) | 0.44 (0.19-0.84) | 7 (3-13) | 0.35 (0.16-0.62) | -0.67 (-1.26--0.07) |
| Gambia | 74 (47-115) | 3.44 (2.16-5.31) | 76 (45-117) | 2.05 (1.22-3.15) | -2.24 (-3.13--1.34) |
| Georgia | 3 (1-5) | 0.08 (0.03-0.13) | 3 (1-5) | 0.14 (0.06-0.24) | 2.14 (0.86-3.44) |
| Germany | 10 (4-17) | 0.02 (0.01-0.04) | 3 (0-6) | 0.01 (0-0.02) | -4.04 (-5.1--2.96) |
| Ghana | 1054 (670-1585) | 3.65 (2.33-5.49) | 962 (531-1589) | 2.07 (1.14-3.42) | -1.96 (-2.63--1.3) |
| Greece | 1 (0-1) | 0.02 (0.01-0.03) | 0 (0-1) | 0.01 (0-0.01) | -2.47 (-3.59--1.34) |
| Greenland | 0 (0-0) | 0 (0-0.01) | 0 (0-0) | 0 (0-0.01) | -2.5 (-3.37--1.63) |
| Grenada | 0 (0-1) | 0.29 (0.15-0.46) | 0 (0-0) | 0.23 (0.08-0.41) | -1.25 (-1.6--0.89) |
| Guam | 0 (0-0) | 0 (0-0.01) | 0 (0-0) | 0.03 (0-0.07) | 6.6 (5.15-8.06) |
| Guatemala | 171 (112-249) | 1.03 (0.68-1.5) | 68 (41-103) | 0.48 (0.28-0.72) | -3.83 (-4.39--3.27) |
| Guinea | 490 (306-771) | 3.54 (2.21-5.57) | 512 (317-805) | 2.15 (1.33-3.38) | -1.88 (-2.83--0.92) |
| Guinea-Bissau | 98 (60-146) | 4.5 (2.73-6.67) | 80 (46-135) | 2.34 (1.35-3.92) | -2.46 (-3.37--1.54) |
| Guyana | 8 (4-13) | 0.63 (0.35-1.04) | 4 (2-7) | 0.57 (0.25-1.03) | -1.69 (-2.19--1.19) |
| Haiti | 253 (142-403) | 2.1 (1.17-3.35) | 342 (191-547) | 2.09 (1.17-3.34) | -0.6 (-1.18--0.02) |
| Honduras | 101 (59-156) | 1.17 (0.69-1.82) | 65 (33-108) | 0.61 (0.31-1.01) | -2.88 (-3.37--2.4) |
| Hungary | 1 (0-1) | 0.01 (0.01-0.02) | 0 (0-1) | 0.01 (0-0.01) | -2.71 (-4.01--1.4) |
| Iceland | 0 (0-0) | 0.01 (0-0.02) | 0 (0-0) | 0 (0-0) | -4.62 (-5.1--4.14) |
| India | 17311 (14282-21260) | 1.45 (1.19-1.77) | 9485 (6808-12601) | 0.9 (0.65-1.2) | -2.71 (-2.99--2.42) |
| Indonesia | 2683 (1725-4128) | 1.19 (0.77-1.84) | 804 (497-1415) | 0.38 (0.23-0.67) | -4.51 (-4.78--4.23) |
| Iran (Islamic Republic of) | 60 (44-79) | 0.08 (0.06-0.11) | 13 (7-19) | 0.03 (0.01-0.04) | -2.54 (-3.1--1.97) |
| Iraq | 244 (140-375) | 0.74 (0.42-1.13) | 153 (77-260) | 0.38 (0.19-0.65) | -3.17 (-3.73--2.62) |
| Ireland | 0 (0-0) | 0.01 (0-0.01) | 0 (0-0) | 0 (0-0.01) | -2.98 (-3.53--2.43) |
| Israel | 1 (1-2) | 0.03 (0.01-0.04) | 1 (0-2) | 0.01 (0-0.02) | -3.32 (-3.56--3.08) |
| Italy | 10 (8-12) | 0.04 (0.03-0.04) | 3 (2-5) | 0.02 (0.01-0.02) | -2.17 (-3.34--0.98) |
| Jamaica | 13 (7-19) | 0.46 (0.26-0.69) | 5 (2-9) | 0.32 (0.15-0.58) | -1.48 (-2.1--0.86) |
| Japan | 6 (5-7) | 0.01 (0.01-0.01) | 1 (1-2) | 0 (0-0) | -4.95 (-6.14--3.75) |
| Jordan | 19 (9-31) | 0.3 (0.15-0.48) | 19 (9-33) | 0.19 (0.09-0.32) | -2.45 (-2.99--1.91) |
| Kazakhstan | 13 (6-25) | 0.08 (0.03-0.14) | 8 (3-15) | 0.04 (0.02-0.08) | -1.74 (-2.05--1.44) |
| Kenya | 879 (637-1172) | 1.87 (1.35-2.49) | 815 (611-1069) | 1.44 (1.08-1.89) | -1.31 (-2.04--0.58) |
| Kiribati | 0 (0-1) | 0.34 (0.2-0.51) | 0 (0-0) | 0.16 (0.08-0.26) | -3.09 (-3.6--2.58) |
| Kuwait | 2 (1-3) | 0.11 (0.06-0.16) | 1 (1-2) | 0.05 (0.03-0.08) | -2.73 (-3.48--1.98) |
| Kyrgyzstan | 4 (2-6) | 0.06 (0.03-0.1) | 9 (5-13) | 0.11 (0.06-0.17) | 2.3 (1.93-2.68) |
| Lao People's Democratic Republic | 160 (87-271) | 1.94 (1.05-3.26) | 76 (42-124) | 0.9 (0.5-1.47) | -3.63 (-4.25--3.01) |
| Latvia | 1 (1-2) | 0.07 (0.04-0.11) | 0 (0-0) | 0.02 (0-0.03) | -5.48 (-6.5--4.46) |
| Lebanon | 3 (1-6) | 0.07 (0.02-0.14) | 1 (1-3) | 0.03 (0.01-0.07) | -3.09 (-3.39--2.79) |
| Lesotho | 32 (17-57) | 1.26 (0.66-2.24) | 24 (14-38) | 1.19 (0.67-1.88) | -0.66 (-1.09--0.22) |
| Liberia | 191 (112-308) | 3.57 (2.08-5.76) | 153 (87-242) | 1.96 (1.11-3.1) | -2.58 (-3.45--1.69) |
| Libya | 3 (2-6) | 0.05 (0.02-0.09) | 1 (0-5) | 0.04 (0.01-0.12) | -2.64 (-3.03--2.24) |
| Lithuania | 1 (1-2) | 0.05 (0.02-0.08) | 0 (0-0) | 0.01 (0-0.03) | -5.1 (-6.04--4.16) |
| Luxembourg | 0 (0-0) | 0.01 (0-0.02) | 0 (0-0) | 0 (0-0.01) | -5.05 (-5.81--4.28) |
| Madagascar | 482 (309-725) | 1.93 (1.24-2.9) | 688 (420-1089) | 1.7 (1.04-2.69) | -0.68 (-1.55-0.2) |
| Malawi | 651 (396-972) | 2.73 (1.66-4.07) | 493 (317-735) | 1.81 (1.16-2.69) | -2.19 (-3.16--1.22) |
| Malaysia | 70 (34-121) | 0.29 (0.14-0.5) | 18 (6-32) | 0.08 (0.03-0.14) | -5.42 (-5.87--4.96) |
| Maldives | 4 (2-6) | 0.9 (0.46-1.44) | 0 (0-0) | 0.08 (0.02-0.16) | -10.23 (-10.64--9.81) |
| Mali | 1023 (618-1634) | 4.82 (2.92-7.66) | 1144 (684-1765) | 2.25 (1.34-3.46) | -2.72 (-3.83--1.6) |
| Malta | 0 (0-0) | 0.01 (0-0.02) | 0 (0-0) | 0 (0-0.01) | -2.61 (-3.51--1.71) |
| Marshall Islands | 0 (0-0) | 0.15 (0.07-0.27) | 0 (0-0) | 0.11 (0.05-0.2) | -1.75 (-2.23--1.27) |
| Mauritania | 137 (85-203) | 3.32 (2.07-4.91) | 102 (57-163) | 1.57 (0.87-2.52) | -2.78 (-3.58--1.97) |
| Mauritius | 1 (0-2) | 0.1 (0.02-0.18) | 0 (0-1) | 0.06 (0.02-0.13) | -2.29 (-2.99--1.58) |
| Mexico | 476 (395-552) | 0.39 (0.32-0.45) | 185 (139-235) | 0.2 (0.15-0.26) | -3.56 (-3.93--3.2) |
| Micronesia (Federated States of) | 0 (0-1) | 0.28 (0.14-0.48) | 0 (0-0) | 0.09 (0.04-0.19) | -4.69 (-4.99--4.39) |
| Monaco | 0 (0-0) | 0.01 (0-0.02) | 0 (0-0) | 0.01 (0-0.01) | -2.11 (-3.35--0.86) |
| Mongolia | 6 (3-12) | 0.18 (0.08-0.34) | 5 (2-8) | 0.12 (0.06-0.22) | -1.69 (-1.99--1.39) |
| Montenegro | 0 (0-1) | 0.09 (0.04-0.16) | 0 (0-0) | 0.02 (0.01-0.04) | -6.12 (-6.86--5.39) |
| Morocco | 38 (17-65) | 0.1 (0.05-0.17) | 14 (6-24) | 0.04 (0.02-0.08) | -3.13 (-3.62--2.64) |
| Mozambique | 1232 (741-1787) | 4.24 (2.55-6.15) | 1349 (783-2137) | 2.55 (1.48-4.04) | -1.97 (-2.94--1) |
| Myanmar | 1441 (852-2198) | 2.7 (1.6-4.11) | 720 (409-1205) | 1.4 (0.79-2.34) | -2.84 (-3.03--2.66) |
| Namibia | 26 (16-42) | 1.05 (0.63-1.71) | 15 (7-27) | 0.54 (0.26-0.96) | -2.32 (-2.83--1.81) |
| Nauru | 0 (0-0) | 0.03 (0-0.09) | 0 (0-0) | 0.02 (0-0.07) | -2.45 (-3.33--1.55) |
| Nepal | 539 (311-848) | 1.44 (0.83-2.26) | 217 (119-363) | 0.7 (0.39-1.18) | -3.37 (-3.84--2.89) |
| Netherlands | 4 (1-7) | 0.04 (0.02-0.07) | 2 (0-4) | 0.02 (0-0.04) | -2.82 (-3.6--2.03) |
| New Zealand | 0 (0-0) | 0.01 (0-0.01) | 0 (0-0) | 0.01 (0-0.01) | 1.81 (0.8-2.83) |
| Nicaragua | 98 (65-142) | 1.44 (0.95-2.07) | 31 (16-49) | 0.5 (0.26-0.8) | -4.19 (-4.57--3.81) |
| Niger | 795 (486-1252) | 3.74 (2.29-5.89) | 1362 (743-2320) | 2.41 (1.31-4.1) | -2.05 (-3.26--0.82) |
| Nigeria | 5527 (4277-7028) | 2.84 (2.2-3.61) | 8771 (6609-11344) | 2.22 (1.67-2.88) | -1.04 (-2.01--0.06) |
| Niue | 0 (0-0) | 0.07 (0.03-0.13) | 0 (0-0) | 0.06 (0-0.17) | -4.14 (-5--3.26) |
| North Macedonia | 0 (0-0) | 0.01 (0-0.02) | 0 (0-0) | 0.01 (0-0.02) | -0.11 (-1.2-0.99) |
| Northern Mariana Islands | 0 (0-0) | 0.01 (0-0.03) | 0 (0-0) | 0 (0-0.01) | -3.78 (-4.41--3.15) |
| Norway | 0 (0-0) | 0.01 (0-0.01) | 0 (0-0) | 0 (0-0) | -4.93 (-6.39--3.45) |
| Oman | 4 (2-7) | 0.13 (0.06-0.22) | 2 (1-4) | 0.06 (0.03-0.1) | -2.78 (-3.26--2.3) |
| Pakistan | 2594 (1742-3817) | 1.29 (0.87-1.9) | 2962 (2039-4337) | 0.99 (0.69-1.46) | -1.63 (-2.22--1.03) |
| Palau | 0 (0-0) | 0.01 (0-0.03) | 0 (0-0) | 0.01 (0-0.02) | -2.15 (-2.7--1.6) |
| Palestine | 7 (3-12) | 0.17 (0.08-0.29) | 6 (3-11) | 0.11 (0.05-0.19) | -2.21 (-2.94--1.46) |
| Panama | 11 (6-17) | 0.38 (0.2-0.6) | 4 (1-7) | 0.1 (0.03-0.2) | -4.77 (-5.01--4.52) |
| Papua New Guinea | 31 (17-58) | 0.44 (0.23-0.82) | 71 (36-123) | 0.43 (0.22-0.75) | -0.25 (-0.93-0.44) |
| Paraguay | 67 (41-101) | 1.08 (0.66-1.63) | 8 (3-15) | 0.13 (0.05-0.25) | -8.34 (-8.87--7.81) |
| Peru | 570 (356-860) | 1.85 (1.15-2.79) | 152 (73-267) | 0.47 (0.23-0.83) | -3.99 (-4.37--3.6) |
| Philippines | 1637 (1308-2006) | 1.69 (1.35-2.07) | 964 (731-1225) | 0.88 (0.67-1.12) | -2.57 (-2.99--2.13) |
| Poland | 32 (25-43) | 0.12 (0.09-0.16) | 1 (1-2) | 0.01 (0.01-0.01) | -10.36 (-11.39--9.31) |
| Portugal | 2 (1-4) | 0.04 (0.01-0.08) | 0 (0-1) | 0.01 (0-0.02) | -5.35 (-6.37--4.32) |
| Puerto Rico | 1 (0-2) | 0.04 (0.01-0.08) | 0 (0-1) | 0.03 (0-0.08) | -1.72 (-2.83--0.59) |
| Qatar | 0 (0-0) | 0.03 (0.02-0.05) | 0 (0-0) | 0.01 (0.01-0.02) | -4.85 (-5.24--4.46) |
| Republic of C么te d'Ivoire | 880 (558-1344) | 3.33 (2.12-5.09) | 895 (503-1456) | 1.97 (1.11-3.21) | -2.1 (-2.95--1.24) |
| Republic of Korea | 21 (9-37) | 0.06 (0.03-0.11) | 2 (1-4) | 0.02 (0.01-0.03) | -5.61 (-6.67--4.53) |
| Republic of Moldova | 20 (12-29) | 0.51 (0.31-0.75) | 3 (1-6) | 0.22 (0.09-0.41) | -4.37 (-5.34--3.4) |
| Romania | 3 (2-5) | 0.02 (0.01-0.03) | 0 (0-1) | 0 (0-0.01) | -7.11 (-8.23--5.97) |
| Russian Federation | 79 (67-89) | 0.08 (0.07-0.09) | 27 (22-33) | 0.04 (0.03-0.05) | -3.11 (-4.11--2.11) |
| Rwanda | 490 (312-715) | 3.23 (2.05-4.71) | 248 (143-381) | 1.4 (0.81-2.15) | -3.28 (-4.06--2.49) |
| Saint Kitts and Nevis | 0 (0-0) | 0.03 (0.01-0.07) | 0 (0-0) | 0.03 (0-0.06) | -1.91 (-2.49--1.32) |
| Saint Lucia | 0 (0-1) | 0.27 (0.16-0.44) | 0 (0-0) | 0.25 (0.1-0.45) | -1.36 (-2--0.71) |
| Saint Vincent and the Grenadines | 1 (0-1) | 0.42 (0.24-0.65) | 0 (0-0) | 0.31 (0.13-0.53) | -1.68 (-2.14--1.23) |
| Samoa | 0 (0-1) | 0.14 (0.07-0.26) | 0 (0-0) | 0.07 (0.03-0.14) | -1.95 (-2.46--1.44) |
| San Marino | 0 (0-0) | 0.03 (0.01-0.07) | 0 (0-0) | 0.01 (0-0.02) | -5.1 (-6.1--4.08) |
| Sao Tome and Principe | 4 (2-6) | 1.8 (1.06-2.88) | 1 (1-3) | 0.62 (0.29-1.12) | -4.18 (-4.92--3.43) |
| Saudi Arabia | 87 (41-156) | 0.36 (0.17-0.64) | 20 (10-34) | 0.09 (0.04-0.15) | -6.23 (-6.74--5.72) |
| Senegal | 516 (316-756) | 3.13 (1.92-4.59) | 419 (252-654) | 1.82 (1.09-2.84) | -2.03 (-2.85--1.2) |
| Serbia | 6 (3-11) | 0.1 (0.05-0.17) | 1 (0-1) | 0.02 (0.01-0.03) | -7.8 (-8.89--6.69) |
| Seychelles | 0 (0-0) | 0.12 (0.02-0.26) | 0 (0-0) | 0.09 (0.01-0.2) | -1.78 (-2.12--1.45) |
| Sierra Leone | 432 (248-684) | 4.47 (2.57-7.07) | 392 (244-623) | 2.71 (1.69-4.31) | -2.01 (-2.95--1.06) |
| Singapore | 0 (0-0) | 0.01 (0.01-0.02) | 0 (0-0) | 0 (0-0.01) | -4.61 (-5.49--3.72) |
| Slovakia | 0 (0-0) | 0.01 (0-0.01) | 0 (0-0) | 0 (0-0.01) | -3.09 (-4.06--2.12) |
| Slovenia | 0 (0-0) | 0 (0-0.01) | 0 (0-0) | 0 (0-0.01) | 0.08 (-1.43-1.61) |
| Solomon Islands | 3 (1-5) | 0.41 (0.22-0.69) | 3 (1-5) | 0.29 (0.14-0.5) | -1.64 (-2.38--0.9) |
| Somalia | 537 (280-848) | 2.91 (1.52-4.6) | 1167 (547-1991) | 2.54 (1.19-4.33) | -0.44 (-1.56-0.68) |
| South Africa | 305 (216-406) | 0.61 (0.43-0.81) | 220 (154-303) | 0.47 (0.33-0.64) | -1.6 (-1.83--1.37) |
| South Sudan | 335 (184-553) | 2.75 (1.51-4.52) | 442 (217-858) | 2.42 (1.19-4.7) | -0.34 (-1.31-0.64) |
| Spain | 8 (3-12) | 0.04 (0.02-0.06) | 2 (0-4) | 0.01 (0-0.02) | -3.92 (-5.01--2.83) |
| Sri Lanka | 114 (77-161) | 0.66 (0.45-0.93) | 21 (11-36) | 0.15 (0.07-0.25) | -5.58 (-6.03--5.13) |
| Sudan | 100 (53-167) | 0.24 (0.13-0.4) | 72 (36-124) | 0.13 (0.06-0.22) | -2.59 (-3.37--1.81) |
| Suriname | 4 (2-6) | 0.87 (0.44-1.45) | 3 (1-5) | 0.61 (0.28-1.06) | -1.84 (-2.05--1.62) |
| Sweden | 1 (0-2) | 0.01 (0-0.03) | 0 (0-0) | 0 (0-0.01) | -4.63 (-5.52--3.74) |
| Switzerland | 1 (0-2) | 0.02 (0.01-0.04) | 0 (0-1) | 0.01 (0-0.02) | -3.36 (-4.28--2.43) |
| Syrian Arab Republic | 28 (12-53) | 0.13 (0.06-0.24) | 4 (2-9) | 0.04 (0.02-0.09) | -4.28 (-4.85--3.7) |
| Taiwan (Province of China) | 0 (0-1) | 0 (0-0) | 2 (0-3) | 0.02 (0.01-0.04) | 7.59 (6.3-8.89) |
| Tajikistan | 20 (9-38) | 0.2 (0.09-0.38) | 20 (9-37) | 0.15 (0.07-0.27) | -1.48 (-2.05--0.91) |
| Thailand | 354 (186-601) | 0.71 (0.37-1.21) | 32 (16-52) | 0.12 (0.06-0.2) | -6.74 (-7.48--5.99) |
| Timor-Leste | 32 (18-50) | 1.91 (1.09-3.02) | 18 (10-29) | 0.93 (0.53-1.47) | -3.68 (-4.41--2.95) |
| Togo | 228 (143-354) | 2.98 (1.87-4.62) | 198 (115-324) | 1.7 (0.99-2.78) | -2.25 (-3.06--1.43) |
| Tokelau | 0 (0-0) | 0.04 (0-0.11) | 0 (0-0) | 0.14 (0-0.43) | -1.4 (-2.59--0.19) |
| Tonga | 0 (0-0) | 0.17 (0.07-0.31) | 0 (0-0) | 0.08 (0.03-0.15) | -2.64 (-3.18--2.1) |
| Trinidad and Tobago | 4 (1-7) | 0.32 (0.12-0.6) | 2 (1-4) | 0.3 (0.11-0.57) | -0.8 (-1.38--0.23) |
| Tunisia | 6 (3-12) | 0.06 (0.03-0.11) | 2 (1-5) | 0.03 (0.01-0.06) | -2.93 (-3.22--2.63) |
| Turkey | 259 (133-427) | 0.37 (0.19-0.6) | 50 (20-91) | 0.1 (0.04-0.19) | -5.06 (-5.37--4.75) |
| Turkmenistan | 4 (1-7) | 0.06 (0.02-0.12) | 4 (1-6) | 0.07 (0.03-0.12) | 0.44 (-0.03-0.91) |
| Tuvalu | 0 (0-0) | 0.15 (0.06-0.32) | 0 (0-0) | 0.03 (0-0.06) | -6.93 (-7.28--6.59) |
| Uganda | 1048 (684-1570) | 2.38 (1.56-3.57) | 1330 (847-2113) | 1.76 (1.12-2.79) | -1.4 (-2.48--0.31) |
| Ukraine | 28 (13-46) | 0.09 (0.04-0.14) | 7 (1-13) | 0.05 (0.01-0.1) | -2.44 (-3.54--1.33) |
| United Arab Emirates | 0 (0-1) | 0.02 (0.01-0.05) | 0 (0-1) | 0.01 (0-0.02) | -2.25 (-3.05--1.45) |
| United Kingdom | 5 (5-6) | 0.01 (0.01-0.02) | 1 (1-1) | 0 (0-0) | -6.33 (-7.09--5.56) |
| United Republic of Tanzania | 1486 (917-2268) | 2.59 (1.6-3.94) | 2026 (1168-3256) | 2.24 (1.29-3.61) | -0.5 (-1.42-0.44) |
| United States of America | 47 (39-55) | 0.02 (0.02-0.03) | 17 (12-22) | 0.01 (0.01-0.01) | -3.73 (-4.38--3.08) |
| United States Virgin Islands | 0 (0-0) | 0.03 (0.01-0.07) | 0 (0-0) | 0.02 (0-0.04) | -2.7 (-3.3--2.09) |
| Uruguay | 4 (1-7) | 0.15 (0.05-0.25) | 1 (0-1) | 0.04 (0.01-0.09) | -5.44 (-5.99--4.9) |
| Uzbekistan | 33 (19-59) | 0.1 (0.06-0.17) | 71 (40-106) | 0.19 (0.1-0.28) | 2.29 (1.87-2.7) |
| Vanuatu | 1 (0-1) | 0.27 (0.14-0.43) | 1 (0-1) | 0.2 (0.11-0.35) | -1.57 (-2.29--0.85) |
| Venezuela (Bolivarian Republic of) | 105 (47-175) | 0.4 (0.18-0.66) | 39 (13-73) | 0.18 (0.06-0.34) | -2.77 (-3.11--2.43) |
| Viet Nam | 1132 (688-1883) | 1.22 (0.74-2.03) | 216 (109-353) | 0.29 (0.15-0.47) | -5.76 (-5.98--5.54) |
| Yemen | 86 (50-137) | 0.27 (0.16-0.44) | 77 (39-129) | 0.17 (0.08-0.28) | -2.25 (-3.15--1.34) |
| Zambia | 374 (224-548) | 2.05 (1.23-3) | 481 (270-766) | 1.66 (0.93-2.64) | -1.08 (-2.06--0.08) |
| Zimbabwe | 200 (116-305) | 1.07 (0.63-1.64) | 258 (157-397) | 1.15 (0.7-1.77) | 0.91 (0.18-1.65) |
| **Household air pollution from solid fuels** |  |  |  |  |  |
| **region_eapc** |  |  |  |  |  |
| Advanced Health System | 102 (22-269) | 0.01 (0-0.03) | 2 (0-12) | 0 (0-0) | -12.9 (-13.74--12.05) |
| Africa | 24707 (20782-28946) | 1.93 (1.63-2.26) | 27928 (21790-35100) | 1.35 (1.06-1.7) | -1.66 (-2.46--0.85) |
| African Region | 24053 (20361-28234) | 2.24 (1.9-2.63) | 26772 (20946-33522) | 1.49 (1.17-1.87) | -1.79 (-2.65--0.93) |
| America | 2212 (1572-2953) | 0.29 (0.21-0.39) | 577 (350-906) | 0.08 (0.05-0.13) | -4.78 (-5.06--4.51) |
| Andean Latin America | 405 (203-668) | 0.72 (0.36-1.19) | 44 (8-134) | 0.07 (0.01-0.23) | -7.15 (-7.68--6.61) |
| Asia | 31952 (26523-38260) | 0.84 (0.7-1) | 12399 (9058-16363) | 0.41 (0.3-0.54) | -3.54 (-3.83--3.25) |
| Australasia | 0 (0-0) | 0 (0-0) | 0 (0-0) | 0 (0-0) | -12.57 (-13.36--11.79) |
| Basic Health System | 7975 (6155-10049) | 0.31 (0.24-0.39) | 1369 (770-2262) | 0.07 (0.04-0.12) | -5.72 (-6.15--5.28) |
| Caribbean | 383 (246-570) | 0.89 (0.57-1.32) | 337 (191-533) | 0.88 (0.5-1.4) | -0.64 (-0.84--0.44) |
| Central Africa | 1897 (1415-2515) | 1.27 (0.95-1.68) | 2887 (1926-4127) | 1.07 (0.72-1.53) | -0.77 (-1.72-0.19) |
| Central Asia | 67 (34-108) | 0.07 (0.04-0.11) | 36 (15-75) | 0.04 (0.02-0.08) | -3.26 (-3.76--2.76) |
| Central Europe | 17 (5-34) | 0.02 (0.01-0.04) | 0 (0-2) | 0 (0-0) | -13.3 (-14.35--12.24) |
| Central Latin America | 527 (320-816) | 0.22 (0.13-0.34) | 158 (80-284) | 0.08 (0.04-0.15) | -4.38 (-4.7--4.06) |
| Central Sub-Saharan Africa | 1023 (661-1577) | 0.83 (0.54-1.28) | 1132 (560-2060) | 0.53 (0.26-0.96) | -1.61 (-2.57--0.64) |
| Commonwealth High Income | 0 (0-1) | 0 (0-0) | 0 (0-0) | 0 (0-0) | -12.1 (-12.84--11.36) |
| Commonwealth Low Income | 12374 (9442-16192) | 2.89 (2.2-3.77) | 7837 (6224-10093) | 1.67 (1.33-2.15) | -2.41 (-3.09--1.71) |
| Commonwealth Middle Income | 23046 (19289-27666) | 1.3 (1.08-1.55) | 16101 (11606-21620) | 0.81 (0.59-1.09) | -2.49 (-2.85--2.12) |
| East Asia | 1159 (842-1542) | 0.1 (0.07-0.13) | 36 (14-84) | 0.01 (0-0.02) | -10.05 (-10.78--9.31) |
| East Asia & Pacific - WB | 7774 (5938-9995) | 0.42 (0.32-0.54) | 1950 (1264-2822) | 0.17 (0.11-0.24) | -4.29 (-4.86--3.72) |
| Eastern Africa | 9817 (7922-12164) | 2.54 (2.05-3.14) | 9882 (7350-12653) | 1.71 (1.27-2.18) | -1.78 (-2.65--0.9) |
| Eastern Europe | 22 (10-47) | 0.02 (0.01-0.03) | 2 (1-8) | 0 (0-0.01) | -7.71 (-8.86--6.55) |
| Eastern Mediterranean Region | 2952 (2043-4219) | 0.46 (0.32-0.66) | 3275 (2257-4560) | 0.4 (0.27-0.55) | -1.54 (-2.02--1.05) |
| Eastern Sub-Saharan Africa | 12207 (10238-14670) | 2.85 (2.39-3.43) | 12444 (9313-15783) | 1.9 (1.42-2.41) | -1.8 (-2.7--0.89) |
| Europe | 124 (37-292) | 0.02 (0.01-0.05) | 5 (1-17) | 0 (0-0) | -10.82 (-11.77--9.86) |
| Europe & Central Asia - WB | 166 (62-345) | 0.03 (0.01-0.06) | 37 (16-84) | 0.01 (0-0.02) | -5.22 (-5.96--4.48) |
| European Region | 166 (62-345) | 0.03 (0.01-0.06) | 37 (16-84) | 0.01 (0-0.02) | -5.24 (-5.97--4.51) |
| High-income Asia Pacific | 1 (0-5) | 0 (0-0) | 0 (0-0) | 0 (0-0) | -18.18 (-19.44--16.9) |
| High-income North America | 0 (0-0) | 0 (0-0) | 0 (0-0) | 0 (0-0) | -6.22 (-6.85--5.59) |
| Latin America & Caribbean - WB | 2212 (1572-2953) | 0.4 (0.29-0.54) | 577 (350-906) | 0.12 (0.07-0.19) | -4.84 (-5.05--4.62) |
| Limited Health System | 43913 (37599-51926) | 1.69 (1.44-1.99) | 29755 (23165-37445) | 1 (0.78-1.26) | -2.53 (-2.98--2.07) |
| Middle East & North Africa - WB | 182 (100-314) | 0.05 (0.03-0.08) | 53 (27-93) | 0.01 (0.01-0.02) | -5.97 (-6.36--5.57) |
| Minimal Health System | 7004 (5543-8705) | 2.27 (1.8-2.82) | 9782 (7324-12893) | 1.59 (1.19-2.1) | -1.41 (-2.41--0.39) |
| North Africa and Middle East | 388 (230-633) | 0.07 (0.04-0.12) | 259 (164-403) | 0.05 (0.03-0.07) | -3.1 (-3.46--2.74) |
| North America | 0 (0-0) | 0 (0-0) | 0 (0-0) | 0 (0-0) | -6.23 (-6.86--5.59) |
| Northern Africa | 151 (94-225) | 0.08 (0.05-0.12) | 59 (29-100) | 0.03 (0.01-0.05) | -4.29 (-4.67--3.91) |
| Oceania | 39 (23-65) | 0.36 (0.21-0.6) | 72 (38-121) | 0.35 (0.18-0.59) | -0.28 (-0.89-0.34) |
| Region of the Americas | 2212 (1572-2953) | 0.29 (0.21-0.39) | 577 (350-906) | 0.08 (0.05-0.13) | -4.78 (-5.06--4.51) |
| South-East Asia Region | 25601 (20933-31315) | 1.41 (1.16-1.73) | 9210 (6701-12356) | 0.6 (0.44-0.8) | -3.86 (-4.15--3.57) |
| South Asia | 23842 (19605-29635) | 1.45 (1.2-1.81) | 10191 (7353-13448) | 0.67 (0.49-0.89) | -3.61 (-3.93--3.28) |
| South Asia - WB | 24014 (19802-29823) | 1.43 (1.18-1.78) | 10371 (7520-13683) | 0.65 (0.47-0.86) | -3.62 (-3.94--3.29) |
| Southeast Asia | 6686 (5017-8794) | 1.13 (0.85-1.49) | 1856 (1193-2702) | 0.34 (0.22-0.5) | -4.52 (-4.81--4.23) |
| Southern Africa | 2874 (2202-3625) | 1.66 (1.28-2.1) | 2612 (1884-3634) | 1.06 (0.77-1.48) | -1.9 (-2.57--1.22) |
| Southern Latin America | 23 (6-60) | 0.05 (0.01-0.12) | 1 (0-5) | 0 (0-0.01) | -12.13 (-12.56--11.7) |
| Southern Sub-Saharan Africa | 419 (279-562) | 0.54 (0.36-0.72) | 312 (198-479) | 0.4 (0.25-0.61) | -1.22 (-1.55--0.89) |
| Sub-Saharan Africa - WB | 24646 (20725-28879) | 2.25 (1.89-2.63) | 27919 (21784-35082) | 1.51 (1.18-1.89) | -1.76 (-2.64--0.88) |
| Tropical Latin America | 887 (628-1151) | 0.55 (0.39-0.71) | 48 (17-94) | 0.03 (0.01-0.06) | -9.58 (-10.07--9.08) |
| Western Africa | 9968 (7760-12201) | 2.6 (2.02-3.18) | 12488 (9424-16111) | 1.66 (1.25-2.14) | -1.96 (-2.91--1) |
| Western Europe | 1 (0-4) | 0 (0-0) | 0 (0-0) | 0 (0-0) | -11.34 (-12.29--10.38) |
| Western Pacific Region | 4010 (3188-5064) | 0.27 (0.22-0.34) | 1037 (725-1411) | 0.12 (0.08-0.16) | -4.05 (-4.68--3.41) |
| Western Sub-Saharan Africa | 10932 (8532-13303) | 2.57 (2-3.12) | 14009 (10680-17909) | 1.65 (1.26-2.11) | -1.93 (-2.89--0.97) |
| **country_eapc** |  |  |  |  |  |
| Afghanistan | 69 (37-111) | 0.33 (0.18-0.53) | 172 (91-285) | 0.3 (0.16-0.49) | -0.93 (-1.97-0.11) |
| Albania | 0 (0-0) | 0 (0-0.01) | 0 (0-0) | 0 (0-0) | -6.67 (-7.9--5.42) |
| Algeria | 1 (0-6) | 0 (0-0.02) | 0 (0-0) | 0 (0-0) | -15.02 (-15.59--14.45) |
| American Samoa | 0 (0-0) | 0 (0-0.02) | 0 (0-0) | 0 (0-0.01) | -4.89 (-5.54--4.24) |
| Andorra | 0 (0-0) | 0 (0-0) | 0 (0-0) | 0 (0-0) | -9.15 (-10.49--7.78) |
| Angola | 316 (178-518) | 1.32 (0.75-2.17) | 143 (47-290) | 0.25 (0.08-0.5) | -6.31 (-7.37--5.24) |
| Antigua and Barbuda | 0 (0-0) | 0 (0-0.02) | 0 (0-0) | 0 (0-0) | -7.66 (-8.14--7.17) |
| Argentina | 12 (1-42) | 0.03 (0-0.13) | 0 (0-3) | 0 (0-0.01) | -11.85 (-12.28--11.41) |
| Armenia | 10 (4-21) | 0.29 (0.1-0.59) | 0 (0-1) | 0.02 (0-0.08) | -11.61 (-12.78--10.41) |
| Australia | 0 (0-0) | 0 (0-0) | 0 (0-0) | 0 (0-0) | -13.98 (-14.9--13.05) |
| Austria | 0 (0-0) | 0 (0-0) | 0 (0-0) | 0 (0-0) | -8.99 (-10.02--7.94) |
| Azerbaijan | 8 (2-18) | 0.09 (0.02-0.2) | 0 (0-2) | 0.01 (0-0.03) | -12.46 (-13.82--11.08) |
| Bahamas | 0 (0-0) | 0 (0-0.02) | 0 (0-0) | 0 (0-0.01) | -5.73 (-6.33--5.13) |
| Bahrain | 0 (0-0) | 0 (0-0) | 0 (0-0) | 0 (0-0) | -14.91 (-15.25--14.56) |
| Bangladesh | 6524 (3957-9986) | 3.22 (1.95-4.92) | 1723 (944-2780) | 1.28 (0.7-2.07) | -4.09 (-4.56--3.63) |
| Barbados | 0 (0-0) | 0 (0-0) | 0 (0-0) | 0 (0-0) | -3.41 (-4.22--2.61) |
| Belarus | 0 (0-2) | 0.01 (0-0.02) | 0 (0-0) | 0 (0-0) | -11.59 (-12.89--10.27) |
| Belgium | 0 (0-0) | 0 (0-0) | 0 (0-0) | 0 (0-0) | -10.29 (-11.15--9.43) |
| Belize | 1 (0-1) | 0.24 (0.08-0.48) | 0 (0-1) | 0.07 (0.01-0.19) | -4.49 (-4.99--3.98) |
| Benin | 322 (190-481) | 2.84 (1.67-4.24) | 459 (290-735) | 1.83 (1.16-2.93) | -1.7 (-2.7--0.69) |
| Bermuda | 0 (0-0) | 0 (0-0) | 0 (0-0) | 0 (0-0) | -11.92 (-12.8--11.02) |
| Bhutan | 10 (6-17) | 0.99 (0.6-1.62) | 0 (0-1) | 0.06 (0.01-0.17) | -10.17 (-10.73--9.6) |
| Bolivia (Plurinational State of) | 90 (45-154) | 0.82 (0.41-1.41) | 21 (6-44) | 0.18 (0.05-0.38) | -5.56 (-5.99--5.13) |
| Bosnia and Herzegovina | 2 (1-5) | 0.06 (0.02-0.15) | 0 (0-0) | 0.01 (0-0.02) | -8.8 (-9.77--7.81) |
| Botswana | 17 (10-28) | 0.78 (0.45-1.27) | 2 (0-8) | 0.09 (0-0.35) | -7.73 (-8.16--7.3) |
| Brazil | 832 (586-1081) | 0.54 (0.38-0.7) | 44 (16-87) | 0.03 (0.01-0.05) | -9.56 (-10.05--9.08) |
| Brunei Darussalam | 0 (0-0) | 0 (0-0) | 0 (0-0) | 0 (0-0) | -5.2 (-5.77--4.63) |
| Bulgaria | 0 (0-0) | 0 (0-0) | 0 (0-0) | 0 (0-0) | -3.66 (-5.88--1.38) |
| Burkina Faso | 671 (389-1077) | 3.04 (1.76-4.87) | 986 (583-1552) | 2.17 (1.28-3.41) | -1.25 (-2.3--0.19) |
| Burundi | 326 (203-509) | 2.63 (1.63-4.09) | 435 (258-706) | 1.94 (1.15-3.15) | -1.14 (-2.07--0.2) |
| Cabo Verde | 5 (3-9) | 0.89 (0.46-1.42) | 1 (0-2) | 0.17 (0.05-0.38) | -7.05 (-7.52--6.57) |
| Cambodia | 321 (173-510) | 1.57 (0.85-2.49) | 116 (66-192) | 0.68 (0.39-1.12) | -4.2 (-4.65--3.75) |
| Cameroon | 433 (257-676) | 1.9 (1.13-2.96) | 588 (323-936) | 1.18 (0.65-1.88) | -2.03 (-2.92--1.13) |
| Canada | 0 (0-0) | 0 (0-0) | 0 (0-0) | 0 (0-0) | -12.09 (-13.11--11.06) |
| Central African Republic | 73 (43-126) | 1.21 (0.72-2.09) | 111 (61-193) | 1.23 (0.67-2.13) | -0.17 (-1.12-0.78) |
| Chad | 427 (251-643) | 2.8 (1.65-4.21) | 873 (505-1422) | 2.12 (1.23-3.45) | -1.12 (-2.32-0.09) |
| Chile | 11 (4-19) | 0.07 (0.03-0.13) | 0 (0-2) | 0 (0-0.02) | -12.49 (-13.04--11.93) |
| China | 1131 (821-1509) | 0.1 (0.07-0.14) | 27 (7-73) | 0.01 (0-0.01) | -10.7 (-11.46--9.93) |
| Colombia | 46 (14-87) | 0.1 (0.03-0.2) | 5 (0-21) | 0.01 (0-0.06) | -6.79 (-7.77--5.8) |
| Comoros | 28 (16-42) | 2.95 (1.75-4.49) | 15 (9-25) | 1.89 (1.11-3.03) | -2.39 (-2.98--1.78) |
| Congo | 30 (13-55) | 0.68 (0.31-1.25) | 25 (11-48) | 0.41 (0.17-0.79) | -2.29 (-3.05--1.52) |
| Cook Islands | 0 (0-0) | 0.01 (0-0.04) | 0 (0-0) | 0 (0-0) | -12.48 (-12.98--11.98) |
| Costa Rica | 2 (0-3) | 0.04 (0.01-0.09) | 0 (0-0) | 0 (0-0.01) | -11.14 (-11.55--10.73) |
| Croatia | 0 (0-0) | 0 (0-0.01) | 0 (0-0) | 0 (0-0) | -8.01 (-9.73--6.27) |
| Cuba | 2 (0-5) | 0.02 (0-0.05) | 0 (0-0) | 0 (0-0.01) | -8.77 (-9.65--7.89) |
| Cyprus | 0 (0-0) | 0 (0-0) | 0 (0-0) | 0 (0-0) | -14.87 (-15.79--13.95) |
| Czechia | 0 (0-0) | 0 (0-0) | 0 (0-0) | 0 (0-0) | -6.5 (-7.86--5.11) |
| Democratic People's Republic of Korea | 28 (14-49) | 0.11 (0.05-0.19) | 9 (4-20) | 0.06 (0.03-0.14) | -2.79 (-3.24--2.34) |
| Democratic Republic of the Congo | 594 (320-1005) | 0.69 (0.37-1.17) | 852 (373-1713) | 0.62 (0.27-1.25) | -0.13 (-1.11-0.87) |
| Denmark | 0 (0-0) | 0 (0-0) | 0 (0-0) | 0 (0-0) | -8.52 (-9.43--7.59) |
| Djibouti | 10 (5-17) | 1.33 (0.66-2.22) | 7 (3-14) | 0.48 (0.19-0.98) | -3.82 (-4.38--3.26) |
| Dominica | 0 (0-1) | 0.34 (0.13-0.63) | 0 (0-0) | 0.08 (0.01-0.3) | -5.87 (-6.44--5.29) |
| Dominican Republic | 114 (59-190) | 1.06 (0.55-1.77) | 6 (0-35) | 0.06 (0-0.34) | -10.04 (-10.39--9.69) |
| Ecuador | 31 (9-66) | 0.22 (0.06-0.46) | 2 (0-7) | 0.01 (0-0.05) | -11.2 (-11.86--10.53) |
| Egypt | 25 (8-65) | 0.03 (0.01-0.07) | 0 (0-0) | 0 (0-0) | -19.66 (-20.25--19.07) |
| El Salvador | 52 (32-79) | 0.62 (0.38-0.94) | 2 (0-6) | 0.04 (0.01-0.1) | -9.88 (-10.32--9.44) |
| Equatorial Guinea | 9 (5-18) | 0.97 (0.49-1.86) | 0 (0-2) | 0.01 (0-0.11) | -19.29 (-20.87--17.68) |
| Eritrea | 142 (82-238) | 2.01 (1.15-3.35) | 128 (71-198) | 1.37 (0.76-2.12) | -1.63 (-2.36--0.9) |
| Estonia | 0 (0-1) | 0.02 (0-0.06) | 0 (0-0) | 0 (0-0) | -15.11 (-16.19--14.02) |
| Eswatini | 11 (6-19) | 0.7 (0.39-1.24) | 4 (1-9) | 0.29 (0.08-0.63) | -3.02 (-3.64--2.4) |
| Ethiopia | 4641 (3425-6157) | 4.01 (2.96-5.31) | 3505 (2347-4871) | 2.1 (1.41-2.92) | -2.73 (-3.6--1.86) |
| Fiji | 3 (1-4) | 0.28 (0.15-0.46) | 1 (0-1) | 0.06 (0.01-0.14) | -5.49 (-5.96--5.02) |
| Finland | 0 (0-0) | 0 (0-0) | 0 (0-0) | 0 (0-0) | -9.52 (-10.44--8.59) |
| France | 0 (0-0) | 0 (0-0) | 0 (0-0) | 0 (0-0) | -9.07 (-9.93--8.2) |
| Gabon | 2 (0-6) | 0.09 (0-0.38) | 0 (0-2) | 0.02 (0-0.12) | -5.42 (-6.02--4.82) |
| Gambia | 61 (37-93) | 2.85 (1.72-4.32) | 64 (37-98) | 1.72 (1-2.65) | -2.23 (-3.12--1.33) |
| Georgia | 1 (0-3) | 0.04 (0.01-0.08) | 1 (0-3) | 0.04 (0.01-0.12) | -0.66 (-2.5-1.21) |
| Germany | 0 (0-0) | 0 (0-0) | 0 (0-0) | 0 (0-0) | -8.08 (-9.13--7.01) |
| Ghana | 856 (529-1317) | 2.97 (1.84-4.56) | 640 (327-1096) | 1.38 (0.7-2.36) | -2.8 (-3.47--2.12) |
| Greece | 0 (0-0) | 0 (0-0) | 0 (0-0) | 0 (0-0) | -9.87 (-11.38--8.34) |
| Greenland | 0 (0-0) | 0 (0-0) | 0 (0-0) | 0 (0-0) | -9.45 (-10.36--8.53) |
| Grenada | 0 (0-0) | 0.15 (0.05-0.3) | 0 (0-0) | 0.01 (0-0.03) | -11.84 (-12.22--11.46) |
| Guam | 0 (0-0) | 0 (0-0) | 0 (0-0) | 0 (0-0) | 4.68 (3.05-6.33) |
| Guatemala | 124 (74-191) | 0.75 (0.45-1.15) | 43 (19-73) | 0.3 (0.14-0.51) | -4.22 (-4.77--3.66) |
| Guinea | 421 (261-670) | 3.04 (1.88-4.83) | 443 (271-724) | 1.86 (1.14-3.04) | -1.91 (-2.86--0.95) |
| Guinea-Bissau | 84 (50-125) | 3.83 (2.31-5.68) | 69 (39-115) | 2.02 (1.14-3.35) | -2.44 (-3.35--1.51) |
| Guyana | 5 (2-9) | 0.41 (0.17-0.72) | 1 (0-2) | 0.09 (0.01-0.26) | -6.39 (-7.08--5.69) |
| Haiti | 237 (126-378) | 1.96 (1.05-3.14) | 317 (178-510) | 1.94 (1.09-3.11) | -0.63 (-1.21--0.05) |
| Honduras | 84 (48-133) | 0.98 (0.56-1.54) | 51 (26-88) | 0.48 (0.24-0.82) | -3.1 (-3.58--2.61) |
| Hungary | 0 (0-1) | 0 (0-0.01) | 0 (0-0) | 0 (0-0) | -8.08 (-9.36--6.78) |
| Iceland | 0 (0-0) | 0 (0-0) | 0 (0-0) | 0 (0-0) | -9.99 (-10.62--9.35) |
| India | 14730 (11864-18494) | 1.23 (0.99-1.54) | 6397 (4345-8975) | 0.61 (0.41-0.86) | -3.53 (-3.83--3.23) |
| Indonesia | 2178 (1367-3367) | 0.97 (0.61-1.5) | 368 (165-730) | 0.17 (0.08-0.34) | -6.08 (-6.53--5.62) |
| Iran (Islamic Republic of) | 10 (4-20) | 0.01 (0.01-0.03) | 0 (0-0) | 0 (0-0) | -14.73 (-15.09--14.36) |
| Iraq | 34 (4-115) | 0.1 (0.01-0.35) | 1 (0-7) | 0 (0-0.02) | -14.95 (-16.34--13.53) |
| Ireland | 0 (0-0) | 0 (0-0) | 0 (0-0) | 0 (0-0) | -14.33 (-15.21--13.44) |
| Israel | 0 (0-0) | 0 (0-0) | 0 (0-0) | 0 (0-0) | -9.71 (-9.95--9.47) |
| Italy | 0 (0-1) | 0 (0-0) | 0 (0-0) | 0 (0-0) | -8.4 (-9.73--7.04) |
| Jamaica | 10 (5-15) | 0.35 (0.17-0.55) | 1 (0-3) | 0.08 (0.01-0.22) | -4.75 (-5.5--4) |
| Japan | 0 (0-0) | 0 (0-0) | 0 (0-0) | 0 (0-0) | -12.03 (-13.16--10.89) |
| Jordan | 0 (0-1) | 0.01 (0-0.02) | 0 (0-0) | 0 (0-0) | -13.26 (-13.79--12.73) |
| Kazakhstan | 4 (0-11) | 0.02 (0-0.06) | 0 (0-2) | 0 (0-0.01) | -12.42 (-13.7--11.11) |
| Kenya | 823 (594-1088) | 1.75 (1.26-2.31) | 736 (543-979) | 1.3 (0.96-1.73) | -1.48 (-2.2--0.76) |
| Kiribati | 0 (0-1) | 0.33 (0.19-0.5) | 0 (0-0) | 0.15 (0.08-0.24) | -3.18 (-3.69--2.67) |
| Kuwait | 0 (0-0) | 0 (0-0) | 0 (0-0) | 0 (0-0) | -11.97 (-12.92--11) |
| Kyrgyzstan | 3 (1-5) | 0.04 (0.02-0.08) | 5 (3-9) | 0.07 (0.04-0.12) | 1.71 (1.28-2.15) |
| Lao People's Democratic Republic | 148 (79-254) | 1.78 (0.96-3.06) | 59 (28-101) | 0.7 (0.34-1.2) | -4.15 (-4.79--3.51) |
| Latvia | 0 (0-1) | 0.01 (0-0.05) | 0 (0-0) | 0 (0-0.01) | -12.2 (-13.56--10.82) |
| Lebanon | 0 (0-1) | 0.01 (0-0.03) | 0 (0-0) | 0 (0-0) | -14.41 (-14.8--14.02) |
| Lesotho | 29 (15-52) | 1.15 (0.6-2.03) | 21 (12-33) | 1.04 (0.58-1.65) | -0.82 (-1.25--0.38) |
| Liberia | 171 (99-271) | 3.19 (1.84-5.07) | 138 (79-216) | 1.77 (1.01-2.77) | -2.68 (-3.55--1.79) |
| Libya | 0 (0-0) | 0 (0-0) | 0 (0-0) | 0 (0-0) | -6.66 (-8.01--5.3) |
| Lithuania | 0 (0-1) | 0.01 (0-0.03) | 0 (0-0) | 0 (0-0) | -13.85 (-14.8--12.88) |
| Luxembourg | 0 (0-0) | 0 (0-0) | 0 (0-0) | 0 (0-0) | -11.27 (-12.07--10.46) |
| Madagascar | 469 (302-707) | 1.88 (1.21-2.83) | 663 (403-1042) | 1.64 (0.99-2.57) | -0.74 (-1.61-0.13) |
| Malawi | 618 (370-923) | 2.59 (1.55-3.87) | 465 (303-686) | 1.71 (1.11-2.51) | -2.24 (-3.2--1.27) |
| Malaysia | 6 (1-25) | 0.03 (0-0.11) | 0 (0-0) | 0 (0-0) | -14.97 (-15.6--14.35) |
| Maldives | 3 (2-5) | 0.73 (0.36-1.28) | 0 (0-0) | 0.01 (0-0.04) | -16.74 (-17.26--16.21) |
| Mali | 877 (541-1426) | 4.13 (2.55-6.69) | 954 (570-1480) | 1.87 (1.12-2.9) | -2.91 (-4.02--1.8) |
| Malta | 0 (0-0) | 0 (0-0) | 0 (0-0) | 0 (0-0) | -14.32 (-15.24--13.39) |
| Marshall Islands | 0 (0-0) | 0.13 (0.06-0.24) | 0 (0-0) | 0.09 (0.04-0.16) | -1.85 (-2.32--1.37) |
| Mauritania | 100 (60-153) | 2.42 (1.46-3.69) | 58 (28-99) | 0.9 (0.44-1.52) | -3.61 (-4.41--2.8) |
| Mauritius | 0 (0-1) | 0.04 (0.01-0.09) | 0 (0-0) | 0 (0-0.01) | -12.01 (-12.66--11.36) |
| Mexico | 126 (33-285) | 0.1 (0.03-0.23) | 32 (6-90) | 0.03 (0.01-0.1) | -5.21 (-5.62--4.8) |
| Micronesia (Federated States of) | 0 (0-1) | 0.26 (0.13-0.46) | 0 (0-0) | 0.08 (0.03-0.16) | -5.07 (-5.37--4.76) |
| Monaco | 0 (0-0) | 0 (0-0) | 0 (0-0) | 0 (0-0) | -3.06 (-5.13--0.95) |
| Mongolia | 5 (2-10) | 0.16 (0.07-0.3) | 1 (0-4) | 0.04 (0-0.11) | -5.59 (-6.14--5.03) |
| Montenegro | 0 (0-0) | 0.02 (0-0.08) | 0 (0-0) | 0 (0-0.02) | -9.17 (-10.21--8.12) |
| Morocco | 22 (10-42) | 0.06 (0.03-0.11) | 1 (0-3) | 0 (0-0.01) | -9.85 (-10.57--9.12) |
| Mozambique | 1180 (713-1718) | 4.06 (2.45-5.92) | 1278 (738-2021) | 2.41 (1.39-3.82) | -2.03 (-2.99--1.05) |
| Myanmar | 1303 (773-2017) | 2.44 (1.45-3.77) | 520 (264-883) | 1.01 (0.51-1.72) | -3.53 (-3.77--3.29) |
| Namibia | 21 (11-34) | 0.84 (0.45-1.37) | 7 (1-17) | 0.24 (0.04-0.6) | -4.52 (-5.07--3.96) |
| Nauru | 0 (0-0) | 0.01 (0-0.03) | 0 (0-0) | 0.01 (0-0.02) | -2.66 (-4.53--0.75) |
| Nepal | 469 (273-742) | 1.25 (0.73-1.97) | 167 (92-288) | 0.54 (0.3-0.94) | -3.75 (-4.23--3.28) |
| Netherlands | 0 (0-0) | 0 (0-0) | 0 (0-0) | 0 (0-0) | -10.79 (-11.62--9.94) |
| New Zealand | 0 (0-0) | 0 (0-0) | 0 (0-0) | 0 (0-0) | -7.52 (-8.28--6.76) |
| Nicaragua | 85 (55-122) | 1.24 (0.8-1.78) | 24 (12-40) | 0.39 (0.19-0.64) | -4.6 (-4.98--4.22) |
| Niger | 665 (400-1019) | 3.13 (1.89-4.79) | 1169 (642-2021) | 2.07 (1.14-3.57) | -2.07 (-3.28--0.84) |
| Nigeria | 4174 (3046-5521) | 2.14 (1.57-2.84) | 6003 (3901-8479) | 1.52 (0.99-2.15) | -1.76 (-2.73--0.77) |
| Niue | 0 (0-0) | 0.05 (0.02-0.11) | 0 (0-0) | 0.01 (0-0.05) | -9.42 (-10.36--8.47) |
| North Macedonia | 0 (0-0) | 0 (0-0.01) | 0 (0-0) | 0 (0-0.01) | -4.32 (-5.65--2.98) |
| Northern Mariana Islands | 0 (0-0) | 0 (0-0) | 0 (0-0) | 0 (0-0) | -0.38 (-1.29-0.54) |
| Norway | 0 (0-0) | 0 (0-0) | 0 (0-0) | 0 (0-0) | -11.56 (-12.92--10.18) |
| Oman | 0 (0-1) | 0 (0-0.03) | 0 (0-0) | 0 (0-0) | -16.62 (-17.27--15.97) |
| Pakistan | 2109 (1361-3168) | 1.05 (0.68-1.58) | 1903 (1081-3004) | 0.64 (0.36-1.01) | -2.47 (-3.07--1.86) |
| Palau | 0 (0-0) | 0 (0-0) | 0 (0-0) | 0 (0-0) | -5.88 (-6.45--5.32) |
| Palestine | 3 (1-6) | 0.07 (0.03-0.14) | 0 (0-1) | 0.01 (0-0.02) | -7.43 (-8.12--6.74) |
| Panama | 6 (2-11) | 0.2 (0.07-0.4) | 0 (0-1) | 0 (0-0.04) | -12.86 (-13.54--12.17) |
| Papua New Guinea | 29 (15-52) | 0.41 (0.21-0.74) | 64 (32-110) | 0.39 (0.19-0.67) | -0.31 (-1-0.38) |
| Paraguay | 55 (30-85) | 0.89 (0.49-1.36) | 4 (1-10) | 0.06 (0.01-0.16) | -9.9 (-10.56--9.23) |
| Peru | 284 (128-507) | 0.92 (0.41-1.64) | 22 (1-89) | 0.07 (0-0.27) | -7.56 (-8.26--6.85) |
| Philippines | 1286 (995-1649) | 1.33 (1.03-1.7) | 631 (416-910) | 0.58 (0.38-0.83) | -3 (-3.45--2.55) |
| Poland | 11 (3-22) | 0.04 (0.01-0.08) | 0 (0-0) | 0 (0-0) | -17.98 (-19.04--16.91) |
| Portugal | 0 (0-1) | 0 (0-0.01) | 0 (0-0) | 0 (0-0) | -14.25 (-15.28--13.21) |
| Puerto Rico | 0 (0-0) | 0 (0-0) | 0 (0-0) | 0 (0-0) | -8.04 (-8.95--7.11) |
| Qatar | 0 (0-0) | 0 (0-0) | 0 (0-0) | 0 (0-0) | -13.91 (-14.61--13.2) |
| Republic of C么te d'Ivoire | 693 (417-1080) | 2.63 (1.58-4.08) | 685 (372-1132) | 1.51 (0.82-2.5) | -2.35 (-3.21--1.49) |
| Republic of Korea | 1 (0-4) | 0 (0-0.01) | 0 (0-0) | 0 (0-0) | -21.01 (-22.21--19.79) |
| Republic of Moldova | 15 (8-22) | 0.38 (0.21-0.59) | 1 (0-3) | 0.1 (0.03-0.21) | -6.65 (-7.59--5.69) |
| Romania | 1 (0-3) | 0.01 (0-0.02) | 0 (0-0) | 0 (0-0) | -14.89 (-16.31--13.45) |
| Russian Federation | 4 (1-18) | 0 (0-0.02) | 0 (0-3) | 0 (0-0.01) | -8.92 (-10.64--7.16) |
| Rwanda | 455 (288-666) | 3 (1.9-4.38) | 233 (133-359) | 1.31 (0.75-2.03) | -3.28 (-4.06--2.49) |
| Saint Kitts and Nevis | 0 (0-0) | 0.01 (0-0.03) | 0 (0-0) | 0 (0-0) | -10.9 (-11.49--10.31) |
| Saint Lucia | 0 (0-0) | 0.13 (0.03-0.27) | 0 (0-0) | 0.01 (0-0.06) | -8.95 (-9.61--8.29) |
| Saint Vincent and the Grenadines | 0 (0-1) | 0.22 (0.07-0.42) | 0 (0-0) | 0.02 (0-0.07) | -9.6 (-9.97--9.22) |
| Samoa | 0 (0-1) | 0.13 (0.06-0.24) | 0 (0-0) | 0.06 (0.03-0.11) | -2.29 (-2.8--1.78) |
| San Marino | 0 (0-0) | 0 (0-0) | 0 (0-0) | 0 (0-0) | -7.39 (-8.88--5.88) |
| Sao Tome and Principe | 4 (2-6) | 1.65 (0.97-2.62) | 1 (1-2) | 0.49 (0.23-0.92) | -4.78 (-5.55--4.01) |
| Saudi Arabia | 1 (0-7) | 0 (0-0.03) | 0 (0-0) | 0 (0-0) | -19.53 (-20.2--18.85) |
| Senegal | 393 (245-586) | 2.38 (1.49-3.55) | 358 (208-563) | 1.55 (0.9-2.44) | -1.6 (-2.43--0.76) |
| Serbia | 2 (0-6) | 0.03 (0-0.08) | 0 (0-0) | 0 (0-0.01) | -12.18 (-13.55--10.78) |
| Seychelles | 0 (0-0) | 0.02 (0-0.08) | 0 (0-0) | 0 (0-0) | -12.34 (-12.73--11.95) |
| Sierra Leone | 382 (221-613) | 3.95 (2.28-6.35) | 350 (215-556) | 2.42 (1.49-3.85) | -2.06 (-3.01--1.1) |
| Singapore | 0 (0-0) | 0 (0-0) | 0 (0-0) | 0 (0-0) | -18.74 (-19.59--17.88) |
| Slovakia | 0 (0-0) | 0 (0-0) | 0 (0-0) | 0 (0-0) | -11.66 (-12.78--10.52) |
| Slovenia | 0 (0-0) | 0 (0-0) | 0 (0-0) | 0 (0-0) | -5.79 (-7.15--4.4) |
| Solomon Islands | 3 (1-4) | 0.39 (0.21-0.66) | 3 (1-5) | 0.27 (0.13-0.47) | -1.68 (-2.41--0.93) |
| Somalia | 511 (267-812) | 2.77 (1.45-4.4) | 1107 (515-1878) | 2.41 (1.12-4.09) | -0.49 (-1.61-0.63) |
| South Africa | 163 (90-253) | 0.33 (0.18-0.51) | 42 (11-98) | 0.09 (0.02-0.21) | -5.47 (-5.85--5.1) |
| South Sudan | 262 (137-426) | 2.14 (1.13-3.49) | 394 (197-748) | 2.16 (1.08-4.1) | -0.06 (-1.03-0.91) |
| Spain | 0 (0-2) | 0 (0-0.01) | 0 (0-0) | 0 (0-0) | -12.49 (-13.54--11.42) |
| Sri Lanka | 100 (67-145) | 0.58 (0.39-0.84) | 9 (1-21) | 0.06 (0.01-0.14) | -7.76 (-8.4--7.12) |
| Sudan | 83 (43-142) | 0.2 (0.1-0.34) | 40 (18-77) | 0.07 (0.03-0.14) | -4.12 (-4.91--3.33) |
| Suriname | 1 (0-3) | 0.3 (0.05-0.78) | 0 (0-1) | 0.03 (0-0.18) | -8.63 (-9.13--8.12) |
| Sweden | 0 (0-0) | 0 (0-0) | 0 (0-0) | 0 (0-0) | -10.57 (-11.46--9.67) |
| Switzerland | 0 (0-0) | 0 (0-0) | 0 (0-0) | 0 (0-0) | -6.81 (-7.82--5.79) |
| Syrian Arab Republic | 6 (2-12) | 0.03 (0.01-0.06) | 0 (0-0) | 0 (0-0) | -15.83 (-16.26--15.39) |
| Taiwan (Province of China) | 0 (0-0) | 0 (0-0) | 0 (0-0) | 0 (0-0) | -4.2 (-5.38--3.01) |
| Tajikistan | 16 (7-32) | 0.16 (0.07-0.32) | 13 (6-26) | 0.1 (0.04-0.19) | -2.37 (-2.98--1.75) |
| Thailand | 227 (104-412) | 0.45 (0.21-0.83) | 2 (0-14) | 0.01 (0-0.06) | -13.39 (-14.17--12.61) |
| Timor-Leste | 29 (17-46) | 1.76 (1.03-2.78) | 14 (7-24) | 0.73 (0.37-1.22) | -4.43 (-5.18--3.67) |
| Togo | 193 (119-301) | 2.52 (1.56-3.93) | 169 (98-271) | 1.45 (0.84-2.33) | -2.31 (-3.12--1.49) |
| Tokelau | 0 (0-0) | 0 (0-0.01) | 0 (0-0) | 0 (0-0.01) | -9.13 (-10.22--8.02) |
| Tonga | 0 (0-0) | 0.15 (0.06-0.29) | 0 (0-0) | 0.06 (0.02-0.13) | -3.19 (-3.74--2.64) |
| Trinidad and Tobago | 0 (0-0) | 0 (0-0.03) | 0 (0-0) | 0 (0-0) | -15.84 (-16.71--14.96) |
| Tunisia | 2 (1-4) | 0.02 (0.01-0.04) | 0 (0-0) | 0 (0-0) | -17.55 (-17.87--17.23) |
| Turkey | 64 (8-186) | 0.09 (0.01-0.26) | 0 (0-3) | 0 (0-0.01) | -16.77 (-17.39--16.16) |
| Turkmenistan | 0 (0-0) | 0 (0-0.01) | 0 (0-0) | 0 (0-0) | -11.53 (-12.9--10.15) |
| Tuvalu | 0 (0-0) | 0.15 (0.06-0.32) | 0 (0-0) | 0.02 (0-0.06) | -7.4 (-7.74--7.06) |
| Uganda | 977 (635-1469) | 2.22 (1.44-3.34) | 1197 (764-1905) | 1.58 (1.01-2.52) | -1.55 (-2.63--0.46) |
| Ukraine | 3 (0-10) | 0.01 (0-0.03) | 1 (0-3) | 0 (0-0.02) | -3.96 (-5.38--2.53) |
| United Arab Emirates | 0 (0-0) | 0 (0-0) | 0 (0-0) | 0 (0-0) | -9.24 (-10.15--8.32) |
| United Kingdom | 0 (0-0) | 0 (0-0) | 0 (0-0) | 0 (0-0) | -11.17 (-11.98--10.36) |
| United Republic of Tanzania | 1416 (878-2163) | 2.46 (1.53-3.76) | 1856 (1067-2979) | 2.06 (1.18-3.3) | -0.69 (-1.61-0.24) |
| United States of America | 0 (0-0) | 0 (0-0) | 0 (0-0) | 0 (0-0) | -5.57 (-6.19--4.95) |
| United States Virgin Islands | 0 (0-0) | 0 (0-0.01) | 0 (0-0) | 0 (0-0) | -10 (-11.28--8.69) |
| Uruguay | 1 (0-3) | 0.04 (0-0.11) | 0 (0-0) | 0 (0-0.01) | -12.22 (-12.86--11.58) |
| Uzbekistan | 20 (9-37) | 0.06 (0.03-0.11) | 14 (2-40) | 0.04 (0.01-0.1) | -1.93 (-2.54--1.31) |
| Vanuatu | 1 (0-1) | 0.26 (0.13-0.41) | 1 (0-1) | 0.19 (0.1-0.33) | -1.69 (-2.41--0.96) |
| Venezuela (Bolivarian Republic of) | 3 (0-14) | 0.01 (0-0.05) | 1 (0-5) | 0 (0-0.02) | -4.81 (-5.27--4.34) |
| Viet Nam | 1075 (655-1774) | 1.16 (0.71-1.91) | 134 (57-245) | 0.18 (0.08-0.33) | -7.1 (-7.37--6.83) |
| Yemen | 67 (38-111) | 0.21 (0.12-0.35) | 44 (21-77) | 0.09 (0.05-0.16) | -3.5 (-4.39--2.6) |
| Zambia | 341 (201-500) | 1.87 (1.1-2.74) | 414 (229-663) | 1.43 (0.79-2.29) | -1.26 (-2.26--0.26) |
| Zimbabwe | 177 (104-271) | 0.96 (0.56-1.46) | 236 (140-369) | 1.06 (0.63-1.65) | 1.06 (0.32-1.81) |
| **Ambient particulate matter pollution** |  |  |  |  |  |
| **region_eapc** |  |  |  |  |  |
| Advanced Health System | 572 (391-770) | 0.06 (0.04-0.08) | 158 (119-212) | 0.02 (0.02-0.03) | -3.89 (-4.65--3.12) |
| Africa | 3850 (2362-5664) | 0.3 (0.18-0.44) | 6087 (3354-9770) | 0.3 (0.16-0.47) | 0.32 (-0.51-1.17) |
| African Region | 3722 (2260-5475) | 0.35 (0.21-0.51) | 5922 (3224-9557) | 0.33 (0.18-0.53) | 0.27 (-0.62-1.17) |
| America | 1533 (867-2257) | 0.2 (0.11-0.29) | 750 (457-1026) | 0.11 (0.07-0.15) | -2.55 (-2.89--2.2) |
| Andean Latin America | 400 (184-706) | 0.71 (0.33-1.25) | 176 (84-294) | 0.3 (0.14-0.49) | -3.07 (-3.4--2.74) |
| Asia | 5760 (3672-8858) | 0.15 (0.1-0.23) | 6114 (3451-9528) | 0.2 (0.11-0.31) | -0.06 (-0.36-0.24) |
| Australasia | 2 (0-4) | 0.01 (0-0.03) | 1 (0-2) | 0.01 (0-0.01) | -2.8 (-3.63--1.96) |
| Basic Health System | 3277 (1869-4821) | 0.13 (0.07-0.19) | 2206 (1341-3014) | 0.12 (0.07-0.16) | -1.32 (-1.7--0.95) |
| Caribbean | 79 (31-161) | 0.18 (0.07-0.37) | 117 (57-186) | 0.31 (0.15-0.49) | 1.33 (1.11-1.56) |
| Central Africa | 253 (143-415) | 0.17 (0.1-0.28) | 428 (244-728) | 0.16 (0.09-0.27) | 0.04 (-0.92-1.01) |
| Central Asia | 55 (24-96) | 0.06 (0.03-0.1) | 98 (61-144) | 0.1 (0.06-0.15) | 2.67 (2.17-3.17) |
| Central Europe | 31 (13-47) | 0.04 (0.02-0.06) | 4 (2-5) | 0.01 (0-0.01) | -6.25 (-7.28--5.2) |
| Central Latin America | 604 (338-838) | 0.25 (0.14-0.35) | 282 (171-398) | 0.15 (0.09-0.21) | -2.65 (-3.01--2.3) |
| Central Sub-Saharan Africa | 108 (53-204) | 0.09 (0.04-0.17) | 217 (103-372) | 0.1 (0.05-0.17) | 0.91 (-0.07-1.9) |
| Commonwealth High Income | 14 (11-19) | 0.02 (0.01-0.02) | 6 (4-9) | 0.01 (0.01-0.01) | -3.12 (-3.81--2.42) |
| Commonwealth Low Income | 981 (578-1601) | 0.23 (0.13-0.37) | 835 (482-1349) | 0.18 (0.1-0.29) | -1.05 (-1.77--0.32) |
| Commonwealth Middle Income | 4996 (3116-7841) | 0.28 (0.18-0.44) | 7697 (4167-12419) | 0.39 (0.21-0.63) | 0.83 (0.39-1.26) |
| East Asia | 223 (104-409) | 0.02 (0.01-0.04) | 99 (57-144) | 0.02 (0.01-0.03) | -0.39 (-1.23-0.46) |
| East Asia & Pacific - WB | 1535 (796-2580) | 0.08 (0.04-0.14) | 1252 (559-2080) | 0.11 (0.05-0.18) | -0.62 (-1.17--0.08) |
| Eastern Africa | 630 (417-958) | 0.16 (0.11-0.25) | 869 (539-1374) | 0.15 (0.09-0.24) | 0.03 (-0.87-0.95) |
| Eastern Europe | 115 (84-143) | 0.08 (0.06-0.1) | 36 (27-46) | 0.04 (0.03-0.05) | -2.67 (-3.65--1.69) |
| Eastern Mediterranean Region | 1043 (655-1546) | 0.16 (0.1-0.24) | 1500 (912-2490) | 0.18 (0.11-0.3) | -0.24 (-0.74-0.25) |
| Eastern Sub-Saharan Africa | 756 (503-1145) | 0.18 (0.12-0.27) | 1029 (628-1646) | 0.16 (0.1-0.25) | -0.16 (-1.09-0.77) |
| Europe | 419 (247-594) | 0.08 (0.05-0.11) | 121 (86-167) | 0.03 (0.02-0.04) | -3.33 (-4.14--2.51) |
| Europe & Central Asia - WB | 451 (260-639) | 0.07 (0.04-0.11) | 201 (138-264) | 0.04 (0.03-0.06) | -2.15 (-2.88--1.42) |
| European Region | 452 (261-641) | 0.07 (0.04-0.11) | 201 (139-265) | 0.04 (0.03-0.06) | -2.17 (-2.88--1.45) |
| High-income Asia Pacific | 26 (14-41) | 0.03 (0.02-0.04) | 4 (3-6) | 0.01 (0-0.01) | -5.54 (-6.64--4.42) |
| High-income North America | 48 (40-57) | 0.02 (0.02-0.03) | 18 (13-23) | 0.01 (0.01-0.01) | -3.68 (-4.34--3.01) |
| Latin America & Caribbean - WB | 1486 (821-2212) | 0.27 (0.15-0.4) | 732 (441-1009) | 0.15 (0.09-0.21) | -2.57 (-2.87--2.28) |
| Limited Health System | 6834 (4462-10348) | 0.26 (0.17-0.4) | 9487 (5192-15193) | 0.32 (0.17-0.51) | 0.41 (-0.11-0.93) |
| Middle East & North Africa - WB | 522 (352-726) | 0.13 (0.09-0.18) | 336 (226-465) | 0.08 (0.05-0.11) | -2.14 (-2.51--1.76) |
| Minimal Health System | 881 (553-1287) | 0.29 (0.18-0.42) | 1221 (793-1895) | 0.2 (0.13-0.31) | -0.92 (-1.94-0.11) |
| North Africa and Middle East | 738 (454-1035) | 0.14 (0.09-0.2) | 433 (300-588) | 0.08 (0.05-0.1) | -2.47 (-2.83--2.1) |
| North America | 48 (40-57) | 0.02 (0.02-0.03) | 18 (13-23) | 0.01 (0.01-0.01) | -3.68 (-4.34--3.01) |
| Northern Africa | 134 (70-226) | 0.07 (0.04-0.12) | 118 (76-172) | 0.05 (0.03-0.08) | -1.26 (-1.72--0.8) |
| Oceania | 3 (1-8) | 0.03 (0.01-0.08) | 8 (2-20) | 0.04 (0.01-0.1) | 0.62 (-0.01-1.25) |
| Region of the Americas | 1533 (867-2257) | 0.2 (0.11-0.29) | 750 (457-1026) | 0.11 (0.07-0.15) | -2.55 (-2.89--2.2) |
| South-East Asia Region | 4053 (2463-6510) | 0.22 (0.14-0.36) | 4118 (2296-6313) | 0.27 (0.15-0.41) | -0.36 (-0.73-0.01) |
| South Asia | 3748 (2089-6232) | 0.23 (0.13-0.38) | 4492 (2506-7036) | 0.3 (0.17-0.47) | 0.01 (-0.42-0.43) |
| South Asia - WB | 3772 (2107-6267) | 0.22 (0.13-0.37) | 4529 (2527-7086) | 0.29 (0.16-0.45) | -0.03 (-0.45-0.4) |
| Southeast Asia | 1298 (599-2347) | 0.22 (0.1-0.4) | 1152 (501-1968) | 0.21 (0.09-0.36) | -1.16 (-1.38--0.94) |
| Southern Africa | 341 (204-513) | 0.2 (0.12-0.3) | 510 (286-771) | 0.21 (0.12-0.31) | 0.24 (-0.45-0.93) |
| Southern Latin America | 70 (31-116) | 0.14 (0.06-0.23) | 21 (10-34) | 0.06 (0.03-0.09) | -4.47 (-4.87--4.07) |
| Southern Sub-Saharan Africa | 180 (90-297) | 0.23 (0.12-0.38) | 224 (132-322) | 0.29 (0.17-0.41) | 0.48 (0.14-0.83) |
| Sub-Saharan Africa - WB | 3749 (2276-5502) | 0.34 (0.21-0.5) | 6004 (3304-9672) | 0.32 (0.18-0.52) | 0.26 (-0.65-1.19) |
| Tropical Latin America | 335 (141-564) | 0.21 (0.09-0.35) | 140 (80-200) | 0.08 (0.05-0.12) | -3.37 (-3.9--2.83) |
| Western Africa | 2492 (1434-3831) | 0.65 (0.37-1) | 4162 (2089-7152) | 0.55 (0.28-0.95) | 0.07 (-0.93-1.08) |
| Western Europe | 58 (44-74) | 0.03 (0.02-0.03) | 17 (11-24) | 0.01 (0.01-0.01) | -3.57 (-4.43--2.69) |
| Western Pacific Region | 759 (452-1186) | 0.05 (0.03-0.08) | 579 (297-895) | 0.07 (0.03-0.1) | -0.32 (-0.96-0.32) |
| Western Sub-Saharan Africa | 2693 (1565-4123) | 0.63 (0.37-0.97) | 4511 (2295-7651) | 0.53 (0.27-0.9) | 0 (-1-1.01) |
| **country_eapc** |  |  |  |  |  |
| location_name | Num_1990 | ASR_1990 | Num_2021 | ASR_2021 | EAPC_CI |
| Afghanistan | 10 (4-22) | 0.05 (0.02-0.1) | 25 (11-47) | 0.04 (0.02-0.08) | -0.44 (-1.51-0.65) |
| Albania | 0 (0-0) | 0 (0-0) | 0 (0-0) | 0 (0-0.01) | 3.76 (2.8-4.73) |
| Algeria | 15 (5-32) | 0.04 (0.01-0.09) | 11 (3-30) | 0.02 (0.01-0.07) | -2.12 (-2.38--1.86) |
| American Samoa | 0 (0-0) | 0.02 (0-0.05) | 0 (0-0) | 0.01 (0-0.02) | -6.06 (-6.72--5.4) |
| Andorra | 0 (0-0) | 0.01 (0-0.03) | 0 (0-0) | 0 (0-0.01) | -6.02 (-7.32--4.7) |
| Angola | 43 (18-89) | 0.18 (0.07-0.37) | 120 (41-238) | 0.21 (0.07-0.41) | 1.07 (-0.02-2.17) |
| Antigua and Barbuda | 0 (0-0) | 0.07 (0.02-0.14) | 0 (0-0) | 0.08 (0.03-0.16) | -0.42 (-0.9-0.06) |
| Argentina | 60 (27-105) | 0.18 (0.08-0.31) | 16 (5-27) | 0.06 (0.02-0.11) | -5.09 (-5.46--4.73) |
| Armenia | 13 (5-25) | 0.36 (0.13-0.7) | 6 (3-10) | 0.38 (0.21-0.62) | 0.35 (-0.31-1.02) |
| Australia | 2 (0-4) | 0.01 (0-0.03) | 1 (0-2) | 0.01 (0-0.01) | -3.6 (-4.52--2.66) |
| Austria | 1 (0-1) | 0.01 (0.01-0.02) | 0 (0-0) | 0 (0-0.01) | -3.7 (-4.68--2.71) |
| Azerbaijan | 8 (2-18) | 0.09 (0.02-0.21) | 7 (3-15) | 0.11 (0.04-0.24) | 1.61 (0.88-2.34) |
| Bahamas | 1 (0-1) | 0.21 (0.09-0.37) | 0 (0-1) | 0.17 (0.07-0.31) | -1.63 (-2.21--1.04) |
| Bahrain | 1 (0-1) | 0.11 (0.06-0.17) | 0 (0-0) | 0.03 (0.02-0.05) | -4.87 (-5.28--4.45) |
| Bangladesh | 612 (276-1174) | 0.3 (0.14-0.58) | 297 (122-594) | 0.22 (0.09-0.44) | -2.11 (-2.68--1.54) |
| Barbados | 0 (0-1) | 0.23 (0.11-0.37) | 0 (0-1) | 0.3 (0.13-0.56) | 0.42 (-0.37-1.22) |
| Belarus | 8 (4-14) | 0.12 (0.05-0.21) | 1 (0-3) | 0.04 (0.01-0.07) | -3.56 (-4.5--2.6) |
| Belgium | 2 (1-3) | 0.03 (0.01-0.05) | 1 (0-1) | 0.01 (0-0.02) | -4 (-4.8--3.18) |
| Belize | 1 (0-1) | 0.19 (0.06-0.38) | 1 (0-1) | 0.22 (0.1-0.4) | -0.31 (-0.78-0.17) |
| Benin | 57 (27-99) | 0.5 (0.24-0.87) | 81 (38-161) | 0.32 (0.15-0.64) | -0.95 (-1.99-0.11) |
| Bermuda | 0 (0-0) | 0.01 (0-0.03) | 0 (0-0) | 0 (0-0.01) | -4.7 (-5.55--3.85) |
| Bhutan | 1 (1-3) | 0.13 (0.05-0.26) | 1 (0-2) | 0.17 (0.06-0.31) | 0.58 (0.13-1.03) |
| Bolivia (Plurinational State of) | 60 (25-117) | 0.55 (0.23-1.07) | 31 (12-57) | 0.27 (0.11-0.48) | -3.62 (-4.05--3.18) |
| Bosnia and Herzegovina | 0 (0-1) | 0.01 (0-0.03) | 0 (0-1) | 0.02 (0-0.05) | 2.19 (0.99-3.41) |
| Botswana | 5 (1-11) | 0.22 (0.07-0.5) | 9 (3-18) | 0.4 (0.12-0.75) | 1.33 (0.75-1.91) |
| Brazil | 323 (138-540) | 0.21 (0.09-0.35) | 135 (79-194) | 0.08 (0.05-0.12) | -3.32 (-3.87--2.77) |
| Brunei Darussalam | 0 (0-0) | 0.04 (0.01-0.08) | 0 (0-0) | 0.04 (0.01-0.09) | -1.05 (-1.53--0.58) |
| Bulgaria | 0 (0-0) | 0 (0-0.01) | 0 (0-0) | 0.01 (0-0.02) | 1.74 (-0.05-3.55) |
| Burkina Faso | 97 (47-176) | 0.44 (0.21-0.8) | 160 (79-300) | 0.35 (0.17-0.66) | -0.12 (-1.2-0.96) |
| Burundi | 24 (10-49) | 0.19 (0.08-0.4) | 26 (12-52) | 0.12 (0.05-0.23) | -1.82 (-2.74--0.89) |
| Cabo Verde | 1 (0-2) | 0.17 (0.08-0.31) | 2 (1-3) | 0.38 (0.17-0.72) | 1.8 (1.26-2.34) |
| Cambodia | 26 (9-59) | 0.13 (0.04-0.29) | 18 (7-38) | 0.11 (0.04-0.22) | -2.08 (-2.58--1.58) |
| Cameroon | 102 (41-192) | 0.45 (0.18-0.84) | 149 (65-306) | 0.3 (0.13-0.62) | -0.99 (-1.91--0.06) |
| Canada | 2 (0-4) | 0.01 (0-0.02) | 1 (0-2) | 0.01 (0-0.01) | -2.3 (-3.1--1.49) |
| Central African Republic | 6 (3-11) | 0.1 (0.05-0.19) | 9 (4-17) | 0.1 (0.04-0.18) | -0.09 (-1.04-0.86) |
| Chad | 61 (30-111) | 0.4 (0.2-0.73) | 156 (72-304) | 0.38 (0.17-0.74) | 0.28 (-0.95-1.51) |
| Chile | 7 (2-15) | 0.05 (0.01-0.1) | 5 (2-8) | 0.05 (0.02-0.08) | -1.05 (-1.61--0.49) |
| China | 218 (101-397) | 0.02 (0.01-0.04) | 97 (54-140) | 0.02 (0.01-0.03) | -0.4 (-1.25-0.46) |
| Colombia | 53 (17-101) | 0.12 (0.04-0.23) | 37 (13-71) | 0.11 (0.04-0.22) | -0.76 (-1.43--0.1) |
| Comoros | 1 (1-2) | 0.13 (0.05-0.26) | 1 (0-2) | 0.09 (0.04-0.19) | -1.73 (-2.36--1.1) |
| Congo | 5 (2-12) | 0.11 (0.03-0.28) | 13 (4-28) | 0.21 (0.06-0.45) | 2.21 (1.48-2.94) |
| Cook Islands | 0 (0-0) | 0.02 (0-0.06) | 0 (0-0) | 0.01 (0-0.03) | -5.46 (-6.04--4.88) |
| Costa Rica | 2 (1-4) | 0.05 (0.01-0.1) | 1 (0-1) | 0.03 (0.01-0.06) | -3.6 (-4.09--3.1) |
| Croatia | 0 (0-1) | 0.01 (0-0.02) | 0 (0-1) | 0.02 (0.01-0.04) | 0 (-1.69-1.72) |
| Cuba | 7 (2-13) | 0.08 (0.03-0.15) | 2 (1-3) | 0.04 (0.01-0.07) | -2.86 (-3.65--2.06) |
| Cyprus | 0 (0-1) | 0.04 (0.01-0.08) | 0 (0-0) | 0.01 (0-0.02) | -5.97 (-6.62--5.31) |
| Czechia | 1 (0-1) | 0.01 (0-0.02) | 0 (0-1) | 0.01 (0-0.01) | -0.79 (-2.17-0.6) |
| Democratic People's Republic of Korea | 5 (2-11) | 0.02 (0.01-0.04) | 1 (0-2) | 0.01 (0-0.02) | -3.68 (-4.16--3.2) |
| Democratic Republic of the Congo | 47 (20-97) | 0.06 (0.02-0.11) | 61 (25-133) | 0.04 (0.02-0.1) | -0.17 (-1.15-0.81) |
| Denmark | 0 (0-0) | 0 (0-0.01) | 0 (0-0) | 0 (0-0) | -3.28 (-4.04--2.52) |
| Djibouti | 4 (2-8) | 0.58 (0.25-1.12) | 9 (4-16) | 0.6 (0.26-1.09) | 0.08 (-0.59-0.75) |
| Dominica | 0 (0-0) | 0.19 (0.04-0.49) | 0 (0-0) | 0.55 (0.2-1.11) | 2.3 (1.7-2.9) |
| Dominican Republic | 37 (9-92) | 0.35 (0.09-0.86) | 73 (28-126) | 0.71 (0.28-1.23) | 1.97 (1.59-2.35) |
| Ecuador | 54 (25-92) | 0.37 (0.17-0.63) | 15 (5-27) | 0.1 (0.03-0.18) | -6.33 (-7.04--5.6) |
| Egypt | 58 (21-127) | 0.06 (0.02-0.14) | 48 (24-84) | 0.04 (0.02-0.07) | -1.79 (-2.44--1.13) |
| El Salvador | 14 (5-27) | 0.17 (0.06-0.32) | 4 (1-8) | 0.07 (0.02-0.14) | -3.46 (-3.88--3.04) |
| Equatorial Guinea | 1 (0-3) | 0.11 (0.04-0.26) | 7 (3-12) | 0.38 (0.17-0.68) | 3.8 (2.56-5.05) |
| Eritrea | 16 (8-32) | 0.23 (0.11-0.45) | 20 (9-40) | 0.21 (0.1-0.42) | -0.89 (-1.69--0.08) |
| Estonia | 1 (0-1) | 0.05 (0.01-0.1) | 0 (0-0) | 0.01 (0-0.03) | -6.53 (-7.48--5.57) |
| Eswatini | 3 (1-6) | 0.16 (0.06-0.36) | 3 (1-8) | 0.24 (0.06-0.55) | 0.52 (-0.12-1.16) |
| Ethiopia | 245 (156-377) | 0.21 (0.13-0.33) | 276 (157-463) | 0.17 (0.09-0.28) | -0.63 (-1.52-0.27) |
| Fiji | 0 (0-1) | 0.04 (0.01-0.12) | 1 (0-1) | 0.06 (0.01-0.15) | 0.42 (-0.15-0.98) |
| Finland | 0 (0-0) | 0.01 (0-0.01) | 0 (0-0) | 0 (0-0.01) | -3.13 (-4.01--2.25) |
| France | 12 (3-22) | 0.03 (0.01-0.06) | 4 (0-9) | 0.01 (0-0.03) | -3.23 (-3.96--2.5) |
| Gabon | 6 (1-13) | 0.35 (0.08-0.74) | 7 (3-13) | 0.33 (0.15-0.61) | -0.01 (-0.61-0.6) |
| Gambia | 13 (6-24) | 0.59 (0.26-1.11) | 12 (6-24) | 0.33 (0.15-0.65) | -2.32 (-3.21--1.42) |
| Georgia | 2 (0-4) | 0.04 (0.01-0.1) | 2 (1-4) | 0.1 (0.03-0.2) | 5.79 (4.51-7.08) |
| Germany | 10 (4-17) | 0.02 (0.01-0.04) | 3 (0-6) | 0.01 (0-0.02) | -4.04 (-5.1--2.96) |
| Ghana | 198 (98-360) | 0.69 (0.34-1.25) | 322 (118-673) | 0.69 (0.25-1.45) | 0.54 (-0.21-1.3) |
| Greece | 1 (0-1) | 0.02 (0.01-0.03) | 0 (0-1) | 0.01 (0-0.01) | -2.42 (-3.54--1.3) |
| Greenland | 0 (0-0) | 0 (0-0.01) | 0 (0-0) | 0 (0-0.01) | -2.5 (-3.36--1.63) |
| Grenada | 0 (0-0) | 0.13 (0.04-0.28) | 0 (0-0) | 0.23 (0.08-0.4) | 0.78 (0.38-1.19) |
| Guam | 0 (0-0) | 0 (0-0.01) | 0 (0-0) | 0.03 (0-0.07) | 6.61 (5.17-8.08) |
| Guatemala | 47 (18-95) | 0.28 (0.11-0.57) | 25 (7-52) | 0.17 (0.05-0.37) | -3.01 (-3.59--2.42) |
| Guinea | 69 (33-125) | 0.5 (0.24-0.9) | 68 (32-132) | 0.29 (0.13-0.55) | -1.73 (-2.7--0.75) |
| Guinea-Bissau | 15 (7-27) | 0.67 (0.31-1.24) | 11 (5-20) | 0.32 (0.16-0.59) | -2.61 (-3.52--1.69) |
| Guyana | 3 (1-6) | 0.22 (0.08-0.51) | 3 (1-6) | 0.48 (0.19-0.89) | 1.24 (0.69-1.8) |
| Haiti | 17 (4-42) | 0.14 (0.04-0.35) | 24 (9-54) | 0.15 (0.05-0.33) | -0.15 (-0.74-0.45) |
| Honduras | 17 (7-33) | 0.19 (0.08-0.39) | 14 (5-30) | 0.13 (0.04-0.28) | -1.98 (-2.48--1.47) |
| Hungary | 1 (0-1) | 0.01 (0-0.02) | 0 (0-1) | 0.01 (0-0.01) | -2.12 (-3.44--0.78) |
| Iceland | 0 (0-0) | 0.01 (0-0.02) | 0 (0-0) | 0 (0-0) | -4.62 (-5.1--4.14) |
| India | 2580 (1422-4263) | 0.22 (0.12-0.36) | 3087 (1793-4731) | 0.29 (0.17-0.45) | 0.13 (-0.27-0.54) |
| Indonesia | 504 (200-1022) | 0.22 (0.09-0.45) | 436 (163-876) | 0.21 (0.08-0.41) | -1.53 (-1.76--1.29) |
| Iran (Islamic Republic of) | 50 (34-68) | 0.07 (0.05-0.09) | 13 (7-19) | 0.03 (0.01-0.04) | -2.03 (-2.6--1.44) |
| Iraq | 210 (102-333) | 0.63 (0.31-1.01) | 152 (77-257) | 0.38 (0.19-0.64) | -2.18 (-2.74--1.61) |
| Ireland | 0 (0-0) | 0.01 (0-0.01) | 0 (0-0) | 0 (0-0.01) | -2.96 (-3.51--2.4) |
| Israel | 1 (1-2) | 0.03 (0.01-0.04) | 1 (0-2) | 0.01 (0-0.02) | -3.31 (-3.56--3.07) |
| Italy | 9 (7-11) | 0.04 (0.03-0.04) | 3 (2-5) | 0.02 (0.01-0.02) | -2.14 (-3.32--0.96) |
| Jamaica | 3 (1-8) | 0.12 (0.03-0.27) | 4 (1-7) | 0.24 (0.08-0.47) | 1.53 (1.04-2.02) |
| Japan | 6 (5-7) | 0.01 (0.01-0.01) | 1 (1-2) | 0 (0-0) | -4.93 (-6.13--3.73) |
| Jordan | 19 (9-30) | 0.29 (0.14-0.48) | 19 (9-33) | 0.19 (0.09-0.32) | -2.39 (-2.93--1.84) |
| Kazakhstan | 10 (3-20) | 0.05 (0.01-0.11) | 8 (3-15) | 0.04 (0.02-0.07) | 0.08 (-0.38-0.54) |
| Kenya | 57 (32-96) | 0.12 (0.07-0.2) | 78 (42-142) | 0.14 (0.07-0.25) | 0.52 (-0.25-1.3) |
| Kiribati | 0 (0-0) | 0.01 (0-0.03) | 0 (0-0) | 0.01 (0-0.03) | -1.14 (-1.64--0.64) |
| Kuwait | 2 (1-3) | 0.11 (0.06-0.16) | 1 (1-2) | 0.05 (0.03-0.08) | -2.73 (-3.48--1.97) |
| Kyrgyzstan | 1 (0-2) | 0.01 (0-0.03) | 3 (1-6) | 0.04 (0.02-0.08) | 3.94 (3.05-4.85) |
| Lao People's Democratic Republic | 13 (4-28) | 0.15 (0.05-0.34) | 17 (5-40) | 0.2 (0.06-0.48) | -0.16 (-0.77-0.46) |
| Latvia | 1 (0-2) | 0.05 (0.02-0.09) | 0 (0-0) | 0.02 (0-0.03) | -4.21 (-5.21--3.18) |
| Lebanon | 2 (1-5) | 0.06 (0.02-0.12) | 1 (1-3) | 0.03 (0.01-0.07) | -2.64 (-2.94--2.34) |
| Lesotho | 3 (1-6) | 0.12 (0.06-0.23) | 3 (1-6) | 0.16 (0.07-0.3) | 0.62 (0.14-1.12) |
| Liberia | 21 (10-39) | 0.38 (0.19-0.73) | 15 (8-27) | 0.19 (0.1-0.35) | -1.64 (-2.58--0.69) |
| Libya | 3 (1-6) | 0.05 (0.02-0.09) | 1 (0-4) | 0.04 (0.01-0.12) | -2.62 (-3.02--2.23) |
| Lithuania | 1 (1-2) | 0.04 (0.02-0.07) | 0 (0-0) | 0.01 (0-0.03) | -4.57 (-5.51--3.61) |
| Luxembourg | 0 (0-0) | 0.01 (0-0.02) | 0 (0-0) | 0 (0-0.01) | -5.04 (-5.81--4.27) |
| Madagascar | 13 (7-22) | 0.05 (0.03-0.09) | 25 (14-45) | 0.06 (0.03-0.11) | 1.41 (0.46-2.36) |
| Malawi | 34 (18-59) | 0.14 (0.08-0.25) | 27 (14-49) | 0.1 (0.05-0.18) | -1.44 (-2.43--0.45) |
| Malaysia | 64 (29-109) | 0.27 (0.12-0.46) | 17 (6-32) | 0.08 (0.03-0.14) | -5.2 (-5.64--4.76) |
| Maldives | 1 (0-2) | 0.17 (0.04-0.4) | 0 (0-0) | 0.07 (0.02-0.15) | -5.12 (-5.6--4.63) |
| Mali | 146 (69-262) | 0.69 (0.32-1.23) | 190 (91-358) | 0.37 (0.18-0.7) | -1.69 (-2.83--0.55) |
| Malta | 0 (0-0) | 0.01 (0-0.01) | 0 (0-0) | 0 (0-0.01) | -2.54 (-3.43--1.63) |
| Marshall Islands | 0 (0-0) | 0.02 (0-0.05) | 0 (0-0) | 0.02 (0.01-0.05) | -1.16 (-1.72--0.6) |
| Mauritania | 37 (17-67) | 0.9 (0.41-1.61) | 44 (20-77) | 0.67 (0.31-1.19) | -1.23 (-2.04--0.41) |
| Mauritius | 1 (0-1) | 0.06 (0.01-0.12) | 0 (0-1) | 0.06 (0.02-0.12) | -1.23 (-1.96--0.49) |
| Mexico | 350 (183-478) | 0.29 (0.15-0.39) | 154 (91-209) | 0.17 (0.1-0.23) | -3.11 (-3.47--2.75) |
| Micronesia (Federated States of) | 0 (0-0) | 0.02 (0-0.05) | 0 (0-0) | 0.02 (0-0.04) | -1.81 (-2.14--1.49) |
| Monaco | 0 (0-0) | 0.01 (0-0.02) | 0 (0-0) | 0.01 (0-0.01) | -2.11 (-3.35--0.86) |
| Mongolia | 1 (0-2) | 0.02 (0.01-0.06) | 3 (1-6) | 0.09 (0.02-0.17) | 5.99 (5.24-6.74) |
| Montenegro | 0 (0-1) | 0.06 (0.01-0.13) | 0 (0-0) | 0.02 (0-0.04) | -4.85 (-5.51--4.18) |
| Morocco | 16 (5-32) | 0.04 (0.01-0.08) | 13 (5-23) | 0.04 (0.02-0.07) | -0.51 (-1.1-0.08) |
| Mozambique | 52 (29-84) | 0.18 (0.1-0.29) | 72 (35-128) | 0.14 (0.07-0.24) | -0.88 (-1.86-0.11) |
| Myanmar | 138 (55-291) | 0.26 (0.1-0.55) | 200 (61-432) | 0.39 (0.12-0.84) | 0.84 (0.56-1.13) |
| Namibia | 5 (2-12) | 0.21 (0.06-0.51) | 8 (2-18) | 0.3 (0.07-0.66) | 1.81 (1.2-2.42) |
| Nauru | 0 (0-0) | 0.03 (0-0.08) | 0 (0-0) | 0.02 (0-0.05) | -2.39 (-2.98--1.79) |
| Nepal | 70 (28-144) | 0.19 (0.08-0.38) | 50 (23-90) | 0.16 (0.07-0.29) | -1.56 (-2.12--1) |
| Netherlands | 4 (1-7) | 0.04 (0.02-0.07) | 2 (0-4) | 0.02 (0-0.04) | -2.81 (-3.6--2.02) |
| New Zealand | 0 (0-0) | 0.01 (0-0.01) | 0 (0-0) | 0.01 (0-0.01) | 1.88 (0.87-2.91) |
| Nicaragua | 14 (5-29) | 0.2 (0.08-0.42) | 7 (2-15) | 0.11 (0.04-0.24) | -2.15 (-2.56--1.75) |
| Niger | 130 (63-248) | 0.61 (0.29-1.17) | 193 (82-378) | 0.34 (0.15-0.67) | -1.96 (-3.18--0.73) |
| Nigeria | 1352 (676-2250) | 0.69 (0.35-1.16) | 2766 (1172-4950) | 0.7 (0.3-1.25) | 0.82 (-0.21-1.86) |
| Niue | 0 (0-0) | 0.02 (0-0.05) | 0 (0-0) | 0.05 (0-0.14) | 0.06 (-0.69-0.81) |
| North Macedonia | 0 (0-0) | 0.01 (0-0.01) | 0 (0-0) | 0.01 (0-0.02) | 1.53 (0.47-2.6) |
| Northern Mariana Islands | 0 (0-0) | 0.01 (0-0.03) | 0 (0-0) | 0 (0-0.01) | -3.87 (-4.5--3.24) |
| Norway | 0 (0-0) | 0.01 (0-0.01) | 0 (0-0) | 0 (0-0) | -4.93 (-6.39--3.44) |
| Oman | 4 (2-7) | 0.12 (0.06-0.21) | 2 (1-4) | 0.06 (0.03-0.1) | -2.71 (-3.19--2.24) |
| Pakistan | 485 (215-876) | 0.24 (0.11-0.44) | 1058 (480-1968) | 0.36 (0.16-0.66) | 0.69 (0.06-1.33) |
| Palau | 0 (0-0) | 0.01 (0-0.03) | 0 (0-0) | 0.01 (0-0.02) | -2.14 (-2.69--1.58) |
| Palestine | 4 (2-7) | 0.1 (0.04-0.18) | 6 (3-10) | 0.1 (0.05-0.18) | -1 (-1.78--0.22) |
| Panama | 5 (1-9) | 0.17 (0.04-0.33) | 3 (1-7) | 0.1 (0.03-0.2) | -2.24 (-2.54--1.93) |
| Papua New Guinea | 2 (0-7) | 0.03 (0.01-0.09) | 6 (1-17) | 0.04 (0.01-0.11) | 0.56 (-0.13-1.25) |
| Paraguay | 12 (2-32) | 0.19 (0.04-0.51) | 4 (1-10) | 0.07 (0.01-0.16) | -4.95 (-5.34--4.56) |
| Peru | 286 (114-540) | 0.93 (0.37-1.75) | 130 (51-229) | 0.4 (0.16-0.71) | -2.34 (-2.69--1.99) |
| Philippines | 351 (167-612) | 0.36 (0.17-0.63) | 333 (155-541) | 0.31 (0.14-0.49) | -1.43 (-1.87--0.99) |
| Poland | 22 (9-34) | 0.08 (0.03-0.13) | 1 (1-2) | 0.01 (0.01-0.01) | -9.17 (-10.25--8.08) |
| Portugal | 2 (1-4) | 0.04 (0.01-0.08) | 0 (0-1) | 0.01 (0-0.02) | -5.21 (-6.23--4.18) |
| Puerto Rico | 1 (0-2) | 0.04 (0.01-0.08) | 0 (0-1) | 0.03 (0-0.08) | -1.71 (-2.83--0.58) |
| Qatar | 0 (0-0) | 0.03 (0.02-0.05) | 0 (0-0) | 0.01 (0.01-0.02) | -4.85 (-5.24--4.46) |
| Republic of C么te d'Ivoire | 187 (85-336) | 0.71 (0.32-1.27) | 210 (90-434) | 0.46 (0.2-0.96) | -1.16 (-2.05--0.26) |
| Republic of Korea | 20 (8-35) | 0.06 (0.03-0.11) | 2 (1-4) | 0.02 (0.01-0.03) | -5.52 (-6.59--4.45) |
| Republic of Moldova | 5 (1-11) | 0.13 (0.04-0.29) | 2 (1-3) | 0.12 (0.04-0.25) | 0.09 (-1.14-1.33) |
| Romania | 2 (1-4) | 0.01 (0-0.03) | 0 (0-0) | 0 (0-0.01) | -5.13 (-6.26--3.99) |
| Russian Federation | 74 (60-86) | 0.08 (0.06-0.09) | 27 (21-33) | 0.04 (0.03-0.05) | -2.82 (-3.79--1.83) |
| Rwanda | 35 (16-67) | 0.23 (0.1-0.44) | 16 (6-31) | 0.09 (0.03-0.18) | -3.35 (-4.13--2.56) |
| Saint Kitts and Nevis | 0 (0-0) | 0.03 (0.01-0.06) | 0 (0-0) | 0.03 (0-0.06) | -1.3 (-1.87--0.73) |
| Saint Lucia | 0 (0-0) | 0.15 (0.04-0.28) | 0 (0-0) | 0.24 (0.1-0.43) | 0.34 (-0.33-1.02) |
| Saint Vincent and the Grenadines | 0 (0-0) | 0.2 (0.06-0.4) | 0 (0-0) | 0.29 (0.12-0.51) | 0.39 (-0.19-0.97) |
| Samoa | 0 (0-0) | 0.01 (0-0.04) | 0 (0-0) | 0.01 (0-0.03) | 0.68 (0.15-1.22) |
| San Marino | 0 (0-0) | 0.03 (0.01-0.07) | 0 (0-0) | 0.01 (0-0.02) | -5.1 (-6.1--4.08) |
| Sao Tome and Principe | 0 (0-1) | 0.15 (0.07-0.27) | 0 (0-1) | 0.13 (0.05-0.29) | -0.1 (-0.83-0.63) |
| Saudi Arabia | 87 (41-154) | 0.35 (0.17-0.63) | 20 (10-34) | 0.09 (0.04-0.15) | -6.2 (-6.71--5.69) |
| Senegal | 122 (57-219) | 0.74 (0.34-1.33) | 61 (25-127) | 0.27 (0.11-0.55) | -4.04 (-4.99--3.09) |
| Serbia | 4 (1-8) | 0.06 (0.01-0.13) | 0 (0-1) | 0.02 (0.01-0.03) | -6 (-7.01--4.98) |
| Seychelles | 0 (0-0) | 0.1 (0.02-0.24) | 0 (0-0) | 0.09 (0.01-0.2) | -1.47 (-1.79--1.15) |
| Sierra Leone | 50 (24-95) | 0.52 (0.25-0.99) | 42 (20-77) | 0.29 (0.14-0.53) | -1.63 (-2.59--0.67) |
| Singapore | 0 (0-0) | 0.01 (0.01-0.02) | 0 (0-0) | 0 (0-0.01) | -4.56 (-5.44--3.67) |
| Slovakia | 0 (0-0) | 0.01 (0-0.01) | 0 (0-0) | 0 (0-0.01) | -3.01 (-3.98--2.03) |
| Slovenia | 0 (0-0) | 0 (0-0.01) | 0 (0-0) | 0 (0-0.01) | 0.32 (-1.21-1.87) |
| Solomon Islands | 0 (0-0) | 0.02 (0-0.05) | 0 (0-0) | 0.01 (0-0.04) | -0.86 (-1.6--0.12) |
| Somalia | 26 (11-45) | 0.14 (0.06-0.24) | 60 (26-110) | 0.13 (0.06-0.24) | 0.63 (-0.54-1.82) |
| South Africa | 142 (71-230) | 0.28 (0.14-0.46) | 178 (109-258) | 0.38 (0.23-0.55) | 0.54 (0.28-0.8) |
| South Sudan | 74 (21-171) | 0.6 (0.17-1.4) | 47 (18-111) | 0.26 (0.1-0.61) | -1.87 (-2.98--0.76) |
| Spain | 7 (3-12) | 0.04 (0.02-0.06) | 2 (0-4) | 0.01 (0-0.02) | -3.84 (-4.92--2.74) |
| Sri Lanka | 14 (5-30) | 0.08 (0.03-0.17) | 12 (2-26) | 0.09 (0.02-0.18) | -0.27 (-0.63-0.1) |
| Sudan | 17 (7-32) | 0.04 (0.02-0.08) | 32 (14-63) | 0.06 (0.03-0.11) | 1.54 (0.71-2.38) |
| Suriname | 2 (1-5) | 0.57 (0.15-1.08) | 2 (1-4) | 0.57 (0.25-1.02) | -0.35 (-0.57--0.13) |
| Sweden | 1 (0-2) | 0.01 (0-0.03) | 0 (0-0) | 0 (0-0.01) | -4.63 (-5.51--3.74) |
| Switzerland | 1 (0-2) | 0.02 (0.01-0.04) | 0 (0-1) | 0.01 (0-0.02) | -3.36 (-4.28--2.43) |
| Syrian Arab Republic | 22 (10-43) | 0.1 (0.04-0.2) | 4 (2-9) | 0.04 (0.02-0.09) | -3.68 (-4.26--3.1) |
| Taiwan (Province of China) | 0 (0-1) | 0 (0-0) | 2 (0-3) | 0.02 (0.01-0.04) | 8.15 (6.84-9.47) |
| Tajikistan | 4 (1-10) | 0.04 (0.01-0.1) | 7 (3-14) | 0.05 (0.02-0.1) | 1.63 (0.76-2.51) |
| Thailand | 127 (38-278) | 0.25 (0.08-0.56) | 30 (13-49) | 0.12 (0.05-0.19) | -4.19 (-5.02--3.35) |
| Timor-Leste | 2 (1-5) | 0.14 (0.04-0.32) | 4 (1-10) | 0.2 (0.05-0.5) | 0.82 (0.04-1.6) |
| Togo | 35 (17-63) | 0.46 (0.23-0.83) | 29 (13-54) | 0.25 (0.11-0.47) | -1.93 (-2.79--1.07) |
| Tokelau | 0 (0-0) | 0.04 (0-0.1) | 0 (0-0) | 0.14 (0-0.43) | -1.13 (-2.32-0.07) |
| Tonga | 0 (0-0) | 0.01 (0-0.04) | 0 (0-0) | 0.02 (0-0.05) | 0.45 (-0.15-1.05) |
| Trinidad and Tobago | 4 (1-7) | 0.31 (0.12-0.59) | 2 (1-4) | 0.3 (0.11-0.57) | -0.75 (-1.33--0.17) |
| Tunisia | 4 (2-8) | 0.04 (0.02-0.07) | 2 (1-5) | 0.03 (0.01-0.06) | -1.84 (-2.23--1.45) |
| Turkey | 195 (76-347) | 0.28 (0.11-0.49) | 49 (20-91) | 0.1 (0.04-0.19) | -4.22 (-4.53--3.9) |
| Turkmenistan | 4 (1-7) | 0.06 (0.02-0.12) | 4 (1-6) | 0.07 (0.03-0.12) | 0.56 (0.09-1.04) |
| Tuvalu | 0 (0-0) | 0 (0-0.01) | 0 (0-0) | 0 (0-0.01) | -0.72 (-1.15--0.29) |
| Uganda | 71 (40-125) | 0.16 (0.09-0.28) | 133 (65-245) | 0.18 (0.09-0.32) | 0.15 (-0.96-1.27) |
| Ukraine | 25 (10-42) | 0.08 (0.03-0.13) | 6 (1-11) | 0.04 (0.01-0.09) | -2.17 (-3.28--1.04) |
| United Arab Emirates | 0 (0-1) | 0.02 (0.01-0.05) | 0 (0-1) | 0.01 (0-0.02) | -2.25 (-3.05--1.45) |
| United Kingdom | 5 (5-6) | 0.01 (0.01-0.02) | 1 (1-1) | 0 (0-0) | -6.32 (-7.08--5.56) |
| United Republic of Tanzania | 70 (37-126) | 0.12 (0.06-0.22) | 171 (76-340) | 0.19 (0.08-0.38) | 2.32 (1.34-3.3) |
| United States of America | 47 (38-55) | 0.02 (0.02-0.03) | 17 (12-22) | 0.01 (0.01-0.01) | -3.73 (-4.38--3.08) |
| United States Virgin Islands | 0 (0-0) | 0.03 (0.01-0.07) | 0 (0-0) | 0.02 (0-0.04) | -2.67 (-3.27--2.06) |
| Uruguay | 3 (1-6) | 0.11 (0.03-0.21) | 1 (0-1) | 0.04 (0.01-0.09) | -4.81 (-5.37--4.25) |
| Uzbekistan | 13 (5-28) | 0.04 (0.01-0.08) | 58 (29-95) | 0.15 (0.08-0.25) | 5.22 (4.63-5.82) |
| Vanuatu | 0 (0-0) | 0.01 (0-0.03) | 0 (0-0) | 0.01 (0-0.03) | 0.92 (0.18-1.66) |
| Venezuela (Bolivarian Republic of) | 102 (44-172) | 0.39 (0.17-0.65) | 38 (12-72) | 0.18 (0.06-0.33) | -2.73 (-3.07--2.38) |
| Viet Nam | 56 (21-116) | 0.06 (0.02-0.13) | 81 (27-169) | 0.11 (0.04-0.23) | 1.79 (1.46-2.12) |
| Yemen | 18 (8-33) | 0.06 (0.02-0.1) | 33 (15-62) | 0.07 (0.03-0.13) | 0.39 (-0.57-1.35) |
| Zambia | 33 (15-58) | 0.18 (0.08-0.32) | 67 (27-144) | 0.23 (0.09-0.5) | 0.45 (-0.64-1.55) |
| Zimbabwe | 22 (9-44) | 0.12 (0.05-0.24) | 22 (11-42) | 0.1 (0.05-0.19) | -0.36 (-1.05-0.34) |

Abbreviations: UI: uncertainty intervals; CI: confidence interval; EAPC: estimated annual percentage change.

Table S2: The number of DALYs and the age-standardized DALYs rate of neonatal sepsis and other neonatal infections and changes in regions and 204 countries.

| Characteristics | DALYs | | | | |
| --- | --- | --- | --- | --- | --- |
|  | 1990 | | 2021 | | 1990-2021 |
|  | Number of DALYs cases (95% UI) | The age-standardized DALYs rate/100000(95% UI) | Number of DALYs cases (95% UI) | The age-standardized DALYs rate/100000(95% UI) | EAPC (95%CI) |
| **Air pollution** |  |  |  |  |  |
| **region_eapc** |  |  |  |  |  |
| Advanced Health System | 60716 (47132-77932) | 6.68 (5.18-8.57) | 14419 (10818-19148) | 2.05 (1.54-2.72) | -4.39 (-5.14--3.63) |
| Africa | 2570165 (2171960-2978937) | 201.07 (169.91-232.97) | 3061427 (2484149-3738270) | 148.41 (120.44-181.22) | -1.35 (-2.15--0.54) |
| African Region | 2499741 (2127539-2887831) | 233.03 (198.29-269.03) | 2942610 (2383139-3573321) | 164.05 (132.86-199.2) | -1.47 (-2.32--0.61) |
| America | 337206 (293349-384139) | 44.02 (38.29-50.15) | 119417 (90197-153149) | 17.52 (13.23-22.47) | -3.64 (-3.93--3.34) |
| Andean Latin America | 72493 (50776-100591) | 128.96 (90.36-178.98) | 19841 (11694-31683) | 33.33 (19.63-53.25) | -4.47 (-4.8--4.13) |
| Asia | 3394384 (2868573-3992521) | 89.1 (75.28-104.8) | 1666534 (1341858-2034136) | 54.56 (43.93-66.6) | -2.74 (-3--2.49) |
| Australasia | 168 (19-379) | 1.1 (0.13-2.48) | 86 (19-178) | 0.5 (0.11-1.03) | -2.85 (-3.69--2.01) |
| Basic Health System | 1013055 (840231-1209641) | 39.47 (32.74-47.13) | 321978 (261528-399024) | 17.09 (13.89-21.18) | -3.73 (-4.1--3.36) |
| Caribbean | 41606 (29506-58515) | 96.43 (68.39-135.59) | 40905 (24874-61928) | 107.25 (65.21-162.36) | -0.19 (-0.37-0) |
| Central Africa | 193515 (143785-256215) | 129.5 (96.26-171.28) | 298328 (203777-417846) | 110.8 (75.7-155.19) | -0.67 (-1.62-0.28) |
| Central Asia | 11064 (8081-15593) | 11.73 (8.57-16.53) | 12078 (8802-15883) | 12.28 (8.95-16.15) | -0.07 (-0.28-0.14) |
| Central Europe | 4330 (3294-5581) | 5.23 (3.98-6.75) | 379 (288-496) | 0.75 (0.57-0.98) | -7.61 (-8.61--6.6) |
| Central Latin America | 101811 (88044-116600) | 42.51 (36.76-48.68) | 39654 (29108-51702) | 21.11 (15.5-27.53) | -3.34 (-3.67--3.01) |
| Central Sub-Saharan Africa | 101864 (64929-154430) | 83.06 (52.99-125.81) | 121420 (65498-211392) | 56.85 (30.66-98.97) | -1.31 (-2.27--0.34) |
| Commonwealth High Income | 1309 (994-1727) | 1.66 (1.26-2.2) | 545 (356-794) | 0.74 (0.48-1.08) | -3.14 (-3.84--2.44) |
| Commonwealth Low Income | 1201785 (918833-1568925) | 280.29 (214.63-365.27) | 780418 (627787-990641) | 166.35 (133.82-211.15) | -2.29 (-2.98--1.6) |
| Commonwealth Middle Income | 2524128 (2159134-2945794) | 141.91 (121.35-165.5) | 2142164 (1756860-2579633) | 108.02 (88.59-130.07) | -1.68 (-2.03--1.33) |
| East Asia | 124425 (95628-162772) | 10.84 (8.33-14.18) | 12194 (8694-15769) | 2.2 (1.57-2.85) | -6.01 (-6.76--5.26) |
| East Asia & Pacific - WB | 837995 (654320-1059946) | 45.44 (35.48-57.48) | 288335 (224393-380738) | 24.54 (19.09-32.4) | -3.31 (-3.86--2.77) |
| Eastern Africa | 940171 (757763-1165157) | 243.12 (196.1-301.14) | 967472 (723608-1226452) | 167.07 (124.96-211.8) | -1.65 (-2.52--0.78) |
| Eastern Europe | 12377 (10104-14680) | 8.6 (7.02-10.2) | 3463 (2741-4273) | 4 (3.17-4.94) | -3.3 (-4.27--2.31) |
| Eastern Mediterranean Region | 359615 (264988-482924) | 56.6 (41.71-76.01) | 429793 (318794-572475) | 52.07 (38.62-69.35) | -1.16 (-1.64--0.67) |
| Eastern Sub-Saharan Africa | 1166573 (977328-1395502) | 272.72 (228.66-326) | 1212522 (915140-1530894) | 184.99 (139.63-233.57) | -1.69 (-2.59--0.78) |
| Europe | 48946 (36871-64025) | 9.22 (6.95-12.06) | 11315 (8252-15377) | 2.95 (2.15-4.01) | -4.19 (-5--3.38) |
| Europe & Central Asia - WB | 55611 (42580-70515) | 9.22 (7.06-11.69) | 21424 (16647-27140) | 4.58 (3.56-5.81) | -2.8 (-3.51--2.08) |
| European Region | 55746 (42716-70649) | 9.14 (7-11.58) | 21508 (16710-27219) | 4.51 (3.5-5.7) | -2.81 (-3.51--2.1) |
| High-income Asia Pacific | 2411 (1307-3870) | 2.53 (1.37-4.06) | 363 (228-553) | 0.63 (0.4-0.96) | -5.6 (-6.71--4.49) |
| High-income North America | 4356 (3589-5136) | 1.98 (1.63-2.33) | 1591 (1180-2056) | 0.81 (0.6-1.05) | -3.68 (-4.34--3.01) |
| Latin America & Caribbean - WB | 332954 (288982-379831) | 60.63 (52.62-69.16) | 117853 (88541-151382) | 24.19 (18.17-31.07) | -3.69 (-3.93--3.46) |
| Limited Health System | 4567356 (3934425-5298052) | 175.44 (151.07-203.32) | 3532111 (2938812-4200127) | 118.39 (98.51-140.78) | -1.99 (-2.44--1.54) |
| Middle East & North Africa - WB | 63417 (46965-84516) | 16.01 (11.86-21.34) | 35099 (24369-46508) | 8.32 (5.78-11.02) | -2.88 (-3.23--2.52) |
| Minimal Health System | 709680 (559693-878927) | 229.82 (181.21-284.39) | 990215 (755978-1286023) | 161.38 (123.22-209.54) | -1.35 (-2.36--0.34) |
| North Africa and Middle East | 101507 (78048-129481) | 19.38 (14.9-24.71) | 62330 (44752-83225) | 10.87 (7.8-14.51) | -2.68 (-3.03--2.33) |
| North America | 4356 (3589-5136) | 1.98 (1.63-2.33) | 1591 (1180-2056) | 0.81 (0.6-1.05) | -3.68 (-4.34--3.01) |
| Northern Africa | 25650 (17305-35924) | 13.81 (9.32-19.34) | 15997 (10808-22674) | 7.37 (4.98-10.45) | -2.51 (-2.91--2.1) |
| Oceania | 3785 (2223-6341) | 35.14 (20.64-58.86) | 7177 (3911-12010) | 34.96 (19.06-58.51) | -0.2 (-0.81-0.41) |
| Region of the Americas | 337206 (293349-384139) | 44.02 (38.29-50.15) | 119417 (90197-153149) | 17.52 (13.23-22.47) | -3.64 (-3.93--3.34) |
| South-East Asia Region | 2669054 (2244398-3191975) | 147.3 (123.84-176.05) | 1199716 (929418-1504303) | 78.15 (60.54-97.99) | -3.13 (-3.39--2.86) |
| South Asia | 2483219 (2078503-3011236) | 151.48 (126.9-183.48) | 1321612 (1043227-1655988) | 87.36 (68.96-109.46) | -2.86 (-3.17--2.55) |
| South Asia - WB | 2500889 (2092991-3027843) | 149.04 (124.84-180.17) | 1341228 (1059395-1675358) | 84.59 (66.81-105.65) | -2.87 (-3.18--2.56) |
| Southeast Asia | 718620 (551009-943308) | 121.42 (93.1-159.39) | 270803 (207428-360576) | 50.16 (38.41-66.79) | -3.65 (-3.89--3.42) |
| Southern Africa | 289369 (227528-359488) | 167.63 (131.85-208.27) | 281018 (206508-380599) | 114.58 (84.2-155.19) | -1.62 (-2.29--0.95) |
| Southern Latin America | 8419 (4825-12411) | 16.59 (9.5-24.46) | 1942 (930-3086) | 5.2 (2.5-8.27) | -5.13 (-5.52--4.74) |
| Southern Sub-Saharan Africa | 53899 (39891-68444) | 69.51 (51.45-88.26) | 48276 (35955-67185) | 61.78 (46.02-85.98) | -0.6 (-0.93--0.27) |
| Sub-Saharan Africa - WB | 2555569 (2164291-2965735) | 233.2 (197.48-270.51) | 3053186 (2476785-3728702) | 164.94 (133.81-201.42) | -1.45 (-2.33--0.57) |
| Tropical Latin America | 110034 (91936-131978) | 68.33 (57.08-81.95) | 16896 (12185-22239) | 10.19 (7.35-13.42) | -6.43 (-6.85--6.01) |
| Western Africa | 1121460 (898244-1350408) | 292.25 (233.96-352.17) | 1498613 (1233356-1819812) | 199.15 (163.93-241.8) | -1.5 (-2.45--0.54) |
| Western Europe | 5256 (4095-6712) | 2.36 (1.83-3.01) | 1540 (1028-2178) | 0.78 (0.52-1.1) | -3.59 (-4.45--2.71) |
| Western Pacific Region | 429302 (351120-525705) | 29.15 (23.84-35.7) | 145505 (116995-177562) | 16.88 (13.57-20.6) | -3.11 (-3.73--2.48) |
| Western Sub-Saharan Africa | 1226283 (995498-1464765) | 287.76 (233.62-343.58) | 1666878 (1367418-2016820) | 196.06 (160.9-237.16) | -1.51 (-2.46--0.54) |
| **country_eapc** |  |  |  |  |  |
| Afghanistan | 7082 (3773-11709) | 33.78 (17.96-55.92) | 17662 (9655-29780) | 30.43 (16.64-51.3) | -0.88 (-1.91-0.16) |
| Albania | 18 (9-31) | 0.45 (0.24-0.8) | 5 (2-11) | 0.38 (0.13-0.8) | -2.75 (-3.71--1.78) |
| Algeria | 1478 (518-3015) | 4 (1.4-8.16) | 977 (293-2685) | 2.23 (0.67-6.14) | -2.42 (-2.68--2.17) |
| American Samoa | 1 (0-4) | 1.7 (0.1-4.34) | 0 (0-1) | 0.61 (0.02-1.8) | -5.85 (-6.5--5.19) |
| Andorra | 0 (0-1) | 1.25 (0.33-2.89) | 0 (0-0) | 0.16 (0-0.51) | -6.02 (-7.32--4.7) |
| Angola | 32316 (18222-52477) | 135.4 (76.38-219.81) | 23701 (12942-40601) | 40.92 (22.35-70.07) | -4.51 (-5.53--3.47) |
| Antigua and Barbuda | 4 (1-8) | 6.82 (1.96-12.96) | 4 (1-7) | 7.67 (2.29-14.32) | -0.54 (-1.02--0.06) |
| Argentina | 6486 (3173-10404) | 19.43 (9.5-31.17) | 1442 (509-2512) | 5.58 (1.96-9.73) | -5.51 (-5.86--5.15) |
| Armenia | 2121 (1149-3437) | 58.4 (31.64-94.65) | 607 (333-960) | 36.49 (20.01-57.65) | -2.8 (-3.38--2.22) |
| Australia | 154 (6-365) | 1.25 (0.05-2.95) | 70 (5-155) | 0.49 (0.03-1.09) | -3.65 (-4.57--2.71) |
| Austria | 52 (21-86) | 1.16 (0.47-1.93) | 15 (2-33) | 0.37 (0.05-0.81) | -3.67 (-4.65--2.68) |
| Azerbaijan | 1409 (657-2662) | 15.86 (7.4-29.96) | 674 (267-1410) | 10.45 (4.14-21.87) | -2.6 (-2.99--2.21) |
| Bahamas | 50 (22-89) | 18.93 (8.37-33.5) | 29 (12-53) | 15.26 (6.22-27.5) | -1.65 (-2.24--1.06) |
| Bahrain | 63 (36-100) | 9.9 (5.65-15.67) | 23 (13-39) | 2.68 (1.53-4.59) | -4.87 (-5.28--4.46) |
| Bangladesh | 642106 (392843-991158) | 317.25 (194.44-487.87) | 181733 (99767-294868) | 135.4 (74.33-219.69) | -3.87 (-4.33--3.41) |
| Barbados | 41 (20-67) | 20.58 (9.82-33.44) | 34 (15-64) | 27.18 (11.71-50.46) | 0.41 (-0.38-1.21) |
| Belarus | 751 (352-1375) | 10.95 (5.13-20.06) | 136 (35-271) | 3.38 (0.86-6.76) | -3.77 (-4.71--2.82) |
| Belgium | 144 (54-250) | 2.39 (0.89-4.14) | 46 (8-100) | 0.84 (0.14-1.82) | -4 (-4.81--3.19) |
| Belize | 119 (66-185) | 39.35 (21.98-61.11) | 98 (48-155) | 26.56 (13.13-42.03) | -1.88 (-2.33--1.42) |
| Benin | 34139 (20028-51478) | 300.78 (176.62-453.15) | 48642 (30225-76649) | 194.23 (120.77-305.96) | -1.58 (-2.58--0.56) |
| Bermuda | 0 (0-1) | 1.1 (0.07-2.57) | 0 (0-0) | 0.33 (0-0.89) | -4.74 (-5.59--3.89) |
| Bhutan | 1063 (638-1722) | 100.26 (60.26-162.12) | 123 (60-224) | 20.52 (9.94-37.43) | -6.29 (-6.65--5.93) |
| Bolivia (Plurinational State of) | 13495 (7917-21515) | 123.68 (72.54-196.99) | 4675 (2231-8264) | 39.9 (19.04-70.54) | -4.58 (-4.96--4.19) |
| Bosnia and Herzegovina | 213 (59-504) | 6.54 (1.83-15.51) | 30 (11-66) | 2.27 (0.81-4.89) | -4.43 (-5.42--3.43) |
| Botswana | 2009 (1232-3114) | 90.46 (55.5-140.16) | 1018 (487-1795) | 43.75 (20.91-77.13) | -2.77 (-3.19--2.36) |
| Brazil | 103994 (86323-125481) | 67.17 (55.75-81.04) | 16157 (11642-21500) | 10.13 (7.3-13.48) | -6.34 (-6.76--5.91) |
| Brunei Darussalam | 11 (2-24) | 3.3 (0.48-7.22) | 10 (2-23) | 3.27 (0.55-7.85) | -1.07 (-1.54--0.59) |
| Bulgaria | 26 (13-41) | 0.54 (0.28-0.86) | 24 (8-41) | 0.84 (0.29-1.45) | 0.81 (-1.02-2.67) |
| Burkina Faso | 69101 (40977-109512) | 312.78 (185.43-494.7) | 103187 (62089-161611) | 227 (136.77-355.35) | -1.09 (-2.14--0.03) |
| Burundi | 31521 (19490-48306) | 253.71 (156.89-388.4) | 41525 (25025-67018) | 185.43 (111.78-299.24) | -1.18 (-2.12--0.24) |
| Cabo Verde | 575 (308-930) | 95.48 (51.09-154.23) | 202 (112-352) | 49.45 (27.55-86.3) | -3.44 (-3.87--3.02) |
| Cambodia | 31220 (17186-49286) | 152.5 (84.04-240.8) | 12088 (6791-19422) | 70.6 (39.66-113.44) | -3.99 (-4.45--3.54) |
| Cameroon | 48151 (29664-73928) | 211.25 (130.13-324.08) | 66376 (39555-103627) | 133.57 (79.6-208.51) | -1.84 (-2.73--0.95) |
| Canada | 158 (27-327) | 0.81 (0.14-1.67) | 82 (0-189) | 0.46 (0-1.06) | -2.32 (-3.12--1.51) |
| Central African Republic | 7067 (4247-12252) | 117.57 (70.84-203.39) | 10787 (5928-18680) | 119.16 (65.48-206.32) | -0.17 (-1.11-0.79) |
| Chad | 43937 (25889-66001) | 288.3 (169.79-432.22) | 92573 (54275-147736) | 224.36 (131.57-357.63) | -0.92 (-2.12-0.29) |
| Chile | 1582 (906-2398) | 10.77 (6.17-16.33) | 436 (189-704) | 4.46 (1.93-7.2) | -3.58 (-4.23--2.92) |
| China | 121478 (92924-158193) | 10.98 (8.4-14.3) | 11128 (7969-14462) | 2.09 (1.5-2.72) | -6.19 (-6.94--5.43) |
| Colombia | 8941 (5157-13307) | 20.22 (11.66-30.09) | 3744 (1447-6841) | 11.54 (4.45-21.09) | -2.33 (-3.02--1.65) |
| Comoros | 2611 (1542-3996) | 277.57 (164.18-424.4) | 1458 (862-2327) | 179.03 (105.93-285.6) | -2.36 (-2.95--1.75) |
| Congo | 3111 (1434-5665) | 71.23 (32.84-129.6) | 3423 (1553-5964) | 56.03 (25.42-97.62) | -1.3 (-2.03--0.56) |
| Cook Islands | 1 (0-1) | 2.89 (0.44-7.03) | 0 (0-0) | 0.95 (0.03-2.72) | -6.15 (-6.71--5.58) |
| Costa Rica | 335 (181-531) | 8.55 (4.62-13.54) | 75 (19-137) | 2.87 (0.73-5.22) | -5.13 (-5.55--4.7) |
| C么te d'Ivoire | 79188 (50261-120981) | 299.91 (190.49-457.89) | 80507 (45279-130991) | 177.73 (99.99-289.22) | -2.1 (-2.95--1.24) |
| Croatia | 35 (15-55) | 1.29 (0.57-2.07) | 33 (10-65) | 1.95 (0.56-3.84) | -0.52 (-2.19-1.17) |
| Cuba | 770 (294-1330) | 8.97 (3.41-15.5) | 176 (54-316) | 3.65 (1.11-6.53) | -3.58 (-4.37--2.79) |
| Cyprus | 24 (8-50) | 3.59 (1.26-7.49) | 6 (2-12) | 0.8 (0.26-1.64) | -5.97 (-6.63--5.32) |
| Czechia | 53 (26-86) | 0.86 (0.42-1.39) | 30 (8-58) | 0.58 (0.15-1.14) | -0.85 (-2.22-0.54) |
| Democratic People's Republic of Korea | 2912 (1473-5127) | 11.28 (5.71-19.86) | 921 (389-1896) | 6.5 (2.75-13.4) | -2.9 (-3.35--2.45) |
| Democratic Republic of the Congo | 57733 (31039-97271) | 67.35 (36.24-113.34) | 82217 (36185-164639) | 60.17 (26.48-120.48) | -0.13 (-1.11-0.86) |
| Denmark | 14 (5-25) | 0.44 (0.15-0.81) | 6 (0-13) | 0.18 (0.01-0.43) | -3.29 (-4.05--2.52) |
| Djibouti | 1282 (757-2172) | 171.96 (101.59-291.04) | 1408 (678-2422) | 97.2 (46.82-167.27) | -2.15 (-2.73--1.57) |
| Dominica | 43 (22-73) | 47.58 (24.51-81.42) | 18 (8-34) | 56.68 (24.34-109.25) | -0.37 (-0.99-0.26) |
| Dominican Republic | 13624 (8175-21294) | 126.68 (76.02-197.98) | 7084 (3019-12248) | 69.09 (29.44-119.47) | -2.3 (-2.64--1.97) |
| Ecuador | 7687 (4563-11307) | 53.15 (31.56-78.18) | 1486 (578-2804) | 9.62 (3.75-18.15) | -7.33 (-8.01--6.65) |
| Egypt | 7571 (3332-15556) | 8.41 (3.7-17.27) | 4303 (2129-7649) | 3.46 (1.71-6.15) | -2.8 (-3.4--2.2) |
| El Salvador | 5909 (3725-8614) | 70.72 (44.59-103.1) | 558 (238-1079) | 9.96 (4.23-19.26) | -7.13 (-7.49--6.78) |
| Equatorial Guinea | 956 (486-1820) | 97.62 (49.74-185.58) | 640 (306-1127) | 35.62 (17-62.67) | -5.08 (-5.96--4.19) |
| Eritrea | 14290 (8137-23603) | 201.96 (115.04-333.07) | 13334 (7544-20941) | 142.48 (80.6-223.76) | -1.54 (-2.27--0.81) |
| Estonia | 66 (22-113) | 6.32 (2.11-10.93) | 6 (0-15) | 1 (0.05-2.32) | -7.69 (-8.66--6.72) |
| Eswatini | 1200 (672-1990) | 77.57 (43.47-128.69) | 670 (309-1162) | 48.25 (22.29-83.77) | -1.88 (-2.5--1.26) |
| Ethiopia | 439664 (323264-583064) | 379.96 (279.87-503.66) | 340310 (226672-466941) | 204.14 (135.95-280.12) | -2.61 (-3.48--1.74) |
| Fiji | 261 (147-422) | 28.88 (16.26-46.56) | 97 (34-180) | 11.11 (3.87-20.76) | -3.74 (-4.15--3.32) |
| Finland | 16 (1-38) | 0.5 (0.03-1.22) | 6 (0-15) | 0.24 (0-0.63) | -3.14 (-4.01--2.26) |
| France | 1087 (302-2042) | 2.94 (0.82-5.54) | 336 (32-792) | 1.01 (0.1-2.38) | -3.24 (-3.97--2.51) |
| Gabon | 680 (293-1284) | 39.91 (17.27-75.33) | 653 (301-1158) | 31.6 (14.58-56.07) | -0.67 (-1.26--0.07) |
| Gambia | 6674 (4194-10308) | 309.61 (194.68-477.52) | 6823 (4055-10512) | 184.25 (109.52-283.87) | -2.24 (-3.13--1.34) |
| Georgia | 291 (124-493) | 7.01 (2.98-11.88) | 271 (108-464) | 12.58 (4.99-21.5) | 2.14 (0.86-3.43) |
| Germany | 914 (400-1497) | 2.15 (0.94-3.53) | 247 (40-518) | 0.65 (0.11-1.37) | -4.04 (-5.1--2.96) |
| Ghana | 94806 (60302-142621) | 328.86 (209.41-494.03) | 86575 (47749-142968) | 186.47 (102.88-307.92) | -1.96 (-2.63--1.3) |
| Greece | 73 (23-134) | 1.45 (0.46-2.64) | 25 (8-48) | 0.63 (0.2-1.2) | -2.47 (-3.58--1.34) |
| Greenland | 0 (0-1) | 0.36 (0.01-1.16) | 0 (0-0) | 0.19 (0-0.59) | -2.51 (-3.37--1.65) |
| Grenada | 30 (16-48) | 25.71 (13.36-41.77) | 13 (5-24) | 20.73 (7.49-36.57) | -1.25 (-1.6--0.89) |
| Guam | 1 (0-2) | 0.36 (0.05-0.85) | 3 (0-7) | 2.48 (0.33-5.89) | 6.57 (5.13-8.02) |
| Guatemala | 15420 (10108-22407) | 93.01 (60.97-135.16) | 6137 (3657-9307) | 42.95 (25.61-65.13) | -3.83 (-4.38--3.27) |
| Guinea | 44130 (27580-69374) | 318.68 (199-501.31) | 46065 (28544-72411) | 193.69 (120.05-304.31) | -1.88 (-2.83--0.92) |
| Guinea-Bissau | 8847 (5379-13135) | 404.62 (245.98-600.43) | 7240 (4170-12113) | 210.89 (121.52-352.63) | -2.46 (-3.37--1.54) |
| Guyana | 712 (391-1173) | 57 (31.3-93.81) | 366 (162-665) | 50.99 (22.59-92.76) | -1.69 (-2.19--1.19) |
| Haiti | 22786 (12742-36298) | 189 (105.71-301.28) | 30750 (17214-49230) | 187.84 (105.15-300.5) | -0.6 (-1.18--0.02) |
| Honduras | 9078 (5307-14057) | 105.73 (61.81-163.71) | 5845 (2967-9704) | 54.66 (27.74-90.73) | -2.88 (-3.37--2.4) |
| Hungary | 77 (39-125) | 1.27 (0.65-2.06) | 26 (7-51) | 0.61 (0.16-1.19) | -2.73 (-4.02--1.42) |
| Iceland | 1 (0-3) | 0.63 (0.05-1.48) | 0 (0-1) | 0.16 (0-0.41) | -4.62 (-5.1--4.13) |
| India | 1558124 (1285572-1913466) | 130.06 (107.43-159.64) | 853693 (612848-1134005) | 81.36 (58.41-108.07) | -2.71 (-2.99--2.42) |
| Indonesia | 241430 (155216-371460) | 107.48 (69.11-165.34) | 72388 (44725-127353) | 34.15 (21.09-60.08) | -4.51 (-4.78--4.23) |
| Iran (Islamic Republic of) | 5389 (3940-7113) | 7.26 (5.31-9.58) | 1166 (629-1703) | 2.34 (1.26-3.42) | -2.54 (-3.1--1.97) |
| Iraq | 21929 (12558-33740) | 66.24 (37.93-101.92) | 13747 (6953-23429) | 34.47 (17.43-58.77) | -3.17 (-3.73--2.62) |
| Ireland | 17 (4-34) | 0.66 (0.16-1.31) | 8 (0-21) | 0.28 (0-0.76) | -2.98 (-3.53--2.43) |
| Israel | 119 (54-197) | 2.35 (1.07-3.89) | 75 (25-137) | 0.84 (0.28-1.54) | -3.32 (-3.56--3.08) |
| Italy | 859 (689-1043) | 3.18 (2.55-3.86) | 294 (203-407) | 1.51 (1.04-2.09) | -2.17 (-3.34--0.98) |
| Jamaica | 1152 (652-1710) | 41.62 (23.55-61.8) | 460 (210-831) | 28.99 (13.21-52.34) | -1.48 (-2.1--0.86) |
| Japan | 521 (421-625) | 0.87 (0.7-1.04) | 126 (99-156) | 0.3 (0.24-0.38) | -4.95 (-6.14--3.74) |
| Jordan | 1724 (838-2779) | 27 (13.11-43.51) | 1737 (847-2989) | 16.74 (8.16-28.81) | -2.45 (-2.99--1.91) |
| Kazakhstan | 1213 (544-2220) | 6.82 (3.06-12.48) | 746 (291-1375) | 3.78 (1.48-6.98) | -1.75 (-2.05--1.44) |
| Kenya | 79144 (57305-105486) | 167.93 (121.61-223.77) | 73321 (55012-96201) | 129.4 (97.09-169.78) | -1.31 (-2.04--0.59) |
| Kiribati | 39 (23-60) | 30.28 (17.59-46.26) | 20 (11-33) | 14.57 (7.64-23.17) | -3.09 (-3.6--2.58) |
| Kuwait | 156 (85-233) | 9.66 (5.28-14.42) | 113 (61-175) | 4.72 (2.54-7.28) | -2.72 (-3.47--1.97) |
| Kyrgyzstan | 322 (173-568) | 5.08 (2.72-8.95) | 779 (409-1169) | 10.34 (5.43-15.51) | 2.3 (1.92-2.67) |
| Lao People's Democratic Republic | 14444 (7819-24361) | 174.37 (94.39-293.66) | 6849 (3775-11116) | 81.32 (44.82-131.97) | -3.63 (-4.25--3.01) |
| Latvia | 108 (58-168) | 6.11 (3.26-9.49) | 13 (3-25) | 1.57 (0.34-3.05) | -5.48 (-6.49--4.45) |
| Lebanon | 240 (78-513) | 5.87 (1.9-12.55) | 117 (47-232) | 3.06 (1.24-6.09) | -3.09 (-3.39--2.79) |
| Lesotho | 2911 (1514-5170) | 113.77 (59.3-201.89) | 2167 (1218-3413) | 107.16 (60.23-168.91) | -0.66 (-1.09--0.22) |
| Liberia | 17217 (10052-27753) | 321.39 (187.63-518.12) | 13745 (7804-21738) | 176.38 (100.18-278.83) | -2.58 (-3.45--1.69) |
| Libya | 293 (136-523) | 4.68 (2.17-8.37) | 130 (42-407) | 3.37 (1.1-10.54) | -2.64 (-3.04--2.25) |
| Lithuania | 122 (58-193) | 4.49 (2.14-7.1) | 15 (2-34) | 1.34 (0.19-2.99) | -5.1 (-6.04--4.16) |
| Luxembourg | 2 (1-4) | 0.92 (0.26-1.64) | 1 (0-2) | 0.21 (0.01-0.46) | -5.04 (-5.81--4.27) |
| Madagascar | 43401 (27780-65202) | 174.13 (111.54-261.39) | 61941 (37814-98021) | 153 (93.4-242.06) | -0.68 (-1.55-0.2) |
| Malawi | 58606 (35603-87462) | 245.53 (149.35-366.06) | 44337 (28492-66110) | 162.58 (104.48-242.4) | -2.2 (-3.16--1.22) |
| Malaysia | 6329 (3040-10860) | 26.45 (12.7-45.4) | 1577 (568-2898) | 6.85 (2.47-12.6) | -5.41 (-5.87--4.96) |
| Maldives | 343 (175-551) | 80.76 (41.11-129.51) | 21 (6-42) | 7.15 (2.08-14.42) | -10.22 (-10.64--9.81) |
| Mali | 92084 (55606-146995) | 433.31 (262.61-689.64) | 102940 (61516-158814) | 202.19 (120.86-311.68) | -2.72 (-3.83--1.6) |
| Malta | 2 (1-4) | 0.79 (0.3-1.38) | 1 (0-2) | 0.38 (0.05-0.79) | -2.61 (-3.5--1.71) |
| Marshall Islands | 10 (5-18) | 13.48 (6.57-24.61) | 5 (2-10) | 9.57 (4.1-17.65) | -1.75 (-2.23--1.27) |
| Mauritania | 12336 (7684-18280) | 298.43 (186.03-441.56) | 9163 (5096-14703) | 141.23 (78.55-226.62) | -2.78 (-3.58--1.97) |
| Mauritius | 97 (22-177) | 8.72 (1.99-15.89) | 35 (9-70) | 5.68 (1.5-11.36) | -2.29 (-2.99--1.58) |
| Mexico | 42852 (35586-49712) | 35.19 (29.22-40.82) | 16693 (12544-21120) | 18.37 (13.8-23.23) | -3.56 (-3.93--3.2) |
| Micronesia (Federated States of) | 38 (19-66) | 25.28 (12.67-43.49) | 8 (3-15) | 8.51 (3.68-16.99) | -4.69 (-4.99--4.39) |
| Monaco | 0 (0-0) | 0.8 (0.07-2.02) | 0 (0-0) | 0.51 (0.03-1.25) | -2.11 (-3.35--0.86) |
| Mongolia | 562 (265-1066) | 16.23 (7.64-30.79) | 408 (185-723) | 11.09 (5.02-19.67) | -1.69 (-1.99--1.39) |
| Montenegro | 39 (19-67) | 8.07 (3.88-14.04) | 6 (2-13) | 1.75 (0.57-3.78) | -6.11 (-6.84--5.37) |
| Morocco | 3432 (1563-5890) | 9.1 (4.14-15.62) | 1232 (514-2173) | 4.01 (1.68-7.07) | -3.13 (-3.61--2.64) |
| Mozambique | 110866 (66679-160789) | 381.2 (229.52-553.74) | 121423 (70471-192310) | 229.3 (133.04-363.25) | -1.97 (-2.94--1) |
| Myanmar | 129629 (76643-197739) | 242.78 (143.57-370.05) | 64814 (36795-108442) | 126.01 (71.54-210.83) | -2.84 (-3.03--2.66) |
| Namibia | 2315 (1396-3786) | 94.33 (56.91-154.32) | 1349 (650-2388) | 48.98 (23.6-86.7) | -2.32 (-2.83--1.81) |
| Nauru | 1 (0-1) | 3.12 (0.31-8.21) | 0 (0-1) | 2.21 (0.04-5.88) | -2.45 (-3.33--1.55) |
| Nepal | 48517 (27994-76353) | 129.53 (74.98-203.29) | 19532 (10677-32685) | 63.43 (34.67-106.13) | -3.37 (-3.84--2.89) |
| Netherlands | 369 (134-634) | 3.88 (1.4-6.66) | 148 (16-338) | 1.72 (0.19-3.94) | -2.82 (-3.6--2.03) |
| New Zealand | 14 (3-29) | 0.48 (0.12-0.99) | 16 (3-35) | 0.54 (0.09-1.19) | 1.8 (0.8-2.82) |
| Nicaragua | 8837 (5831-12740) | 129.46 (85.44-186.6) | 2768 (1459-4433) | 44.89 (23.66-71.89) | -4.19 (-4.57--3.81) |
| Niger | 71537 (43747-112665) | 336.69 (206.36-529.74) | 122590 (66877-208802) | 216.61 (118.19-368.89) | -2.05 (-3.26--0.82) |
| Nigeria | 497371 (384880-632436) | 255.59 (197.76-325.09) | 789303 (594684-1020817) | 200.04 (150.72-258.73) | -1.04 (-2.01--0.06) |
| Niue | 0 (0-0) | 6.6 (2.38-12.07) | 0 (0-0) | 5.76 (0.21-14.99) | -4.14 (-5--3.27) |
| North Macedonia | 15 (5-28) | 0.9 (0.31-1.72) | 8 (2-17) | 0.9 (0.27-1.89) | -0.13 (-1.22-0.97) |
| Northern Mariana Islands | 1 (0-1) | 1.02 (0.12-2.53) | 0 (0-0) | 0.41 (0.04-0.99) | -3.78 (-4.4--3.14) |
| Norway | 18 (12-26) | 0.63 (0.42-0.89) | 3 (1-5) | 0.11 (0.05-0.2) | -4.9 (-6.34--3.45) |
| Oman | 389 (185-675) | 11.28 (5.35-19.56) | 200 (100-348) | 5.23 (2.63-9.1) | -2.78 (-3.26--2.3) |
| Pakistan | 233409 (156834-343528) | 116.5 (78.34-171.15) | 266531 (183473-390314) | 89.54 (61.65-131.02) | -1.63 (-2.22--1.03) |
| Palau | 0 (0-0) | 1.2 (0.05-2.95) | 0 (0-0) | 0.77 (0.05-1.99) | -2.15 (-2.7--1.6) |
| Palestine | 654 (306-1095) | 15.46 (7.25-25.89) | 576 (279-1004) | 9.94 (4.81-17.34) | -2.21 (-2.94--1.47) |
| Panama | 969 (499-1541) | 34.14 (17.58-54.29) | 316 (86-626) | 9.28 (2.52-18.44) | -4.77 (-5.01--4.52) |
| Papua New Guinea | 2823 (1487-5228) | 39.95 (21.05-73.96) | 6360 (3264-11026) | 38.84 (19.93-67.32) | -0.25 (-0.93-0.44) |
| Paraguay | 6040 (3662-9131) | 97.27 (58.97-147.04) | 739 (282-1379) | 11.9 (4.54-22.21) | -8.34 (-8.87--7.81) |
| Peru | 51310 (32026-77372) | 166.36 (103.88-250.91) | 13680 (6573-24050) | 42.25 (20.28-74.25) | -3.99 (-4.37--3.6) |
| Philippines | 147298 (117737-180544) | 151.91 (121.43-186.2) | 86755 (65752-110262) | 79.45 (60.22-100.98) | -2.57 (-2.99--2.13) |
| Poland | 2906 (2251-3846) | 11.04 (8.55-14.61) | 124 (88-171) | 0.75 (0.54-1.04) | -10.35 (-11.39--9.31) |
| Portugal | 214 (53-402) | 3.87 (0.95-7.25) | 28 (3-68) | 0.7 (0.08-1.71) | -5.35 (-6.37--4.32) |
| Puerto Rico | 100 (15-217) | 3.17 (0.48-6.88) | 26 (0-60) | 2.95 (0-6.81) | -1.72 (-2.83--0.59) |
| Qatar | 16 (9-27) | 2.91 (1.56-4.93) | 17 (9-28) | 0.92 (0.47-1.5) | -4.82 (-5.21--4.43) |
| Republic of Korea | 1855 (779-3345) | 5.73 (2.41-10.33) | 219 (81-404) | 1.7 (0.62-3.13) | -5.61 (-6.67--4.53) |
| Republic of Moldova | 1759 (1057-2569) | 46.12 (27.71-67.39) | 269 (108-502) | 19.75 (7.91-36.91) | -4.37 (-5.34--3.4) |
| Romania | 288 (155-456) | 1.98 (1.06-3.12) | 27 (11-47) | 0.32 (0.12-0.55) | -7.01 (-8.13--5.88) |
| Russian Federation | 7081 (6083-8032) | 7.42 (6.37-8.42) | 2436 (1948-2953) | 3.73 (2.98-4.52) | -3.11 (-4.11--2.11) |
| Rwanda | 44111 (28036-64309) | 290.65 (184.83-423.77) | 22353 (12859-34281) | 126.23 (72.62-193.6) | -3.28 (-4.06--2.49) |
| Saint Kitts and Nevis | 1 (0-3) | 3.14 (1-5.93) | 1 (0-2) | 2.46 (0.17-5.68) | -1.91 (-2.49--1.32) |
| Saint Lucia | 42 (25-68) | 24.69 (14.58-39.79) | 18 (8-33) | 22.49 (9.34-40.91) | -1.36 (-2--0.71) |
| Saint Vincent and the Grenadines | 47 (26-72) | 38.05 (21.19-58.14) | 17 (7-30) | 27.63 (11.35-47.52) | -1.68 (-2.14--1.23) |
| Samoa | 32 (15-61) | 12.41 (5.91-23.39) | 20 (9-37) | 6.69 (3.01-12.41) | -1.95 (-2.46--1.44) |
| San Marino | 0 (0-1) | 3.06 (0.63-6.73) | 0 (0-0) | 0.63 (0.04-2.07) | -5.1 (-6.1--4.08) |
| Sao Tome and Principe | 358 (212-574) | 161.78 (95.63-259.28) | 133 (63-240) | 56.22 (26.51-101.11) | -4.18 (-4.92--3.43) |
| Saudi Arabia | 7874 (3723-14032) | 32.07 (15.17-57.15) | 1788 (872-3078) | 7.99 (3.89-13.76) | -6.23 (-6.74--5.71) |
| Senegal | 46406 (28470-68019) | 281.32 (172.82-412.66) | 37743 (22637-58879) | 163.77 (98.24-255.47) | -2.03 (-2.85--1.2) |
| Serbia | 568 (270-1025) | 8.58 (4.08-15.49) | 51 (21-84) | 1.58 (0.65-2.6) | -7.79 (-8.88--6.68) |
| Seychelles | 8 (2-19) | 10.54 (1.9-23.82) | 6 (1-14) | 8.03 (1.19-18.21) | -1.78 (-2.12--1.45) |
| Sierra Leone | 38870 (22345-61565) | 402.16 (231.09-635.82) | 35253 (21921-56022) | 244.07 (151.91-388.02) | -2.01 (-2.95--1.06) |
| Singapore | 24 (13-38) | 1.01 (0.53-1.55) | 8 (3-14) | 0.3 (0.1-0.52) | -4.61 (-5.49--3.72) |
| Slovakia | 22 (10-37) | 0.58 (0.26-0.98) | 7 (2-14) | 0.25 (0.08-0.51) | -3.14 (-4.11--2.17) |
| Slovenia | 2 (0-5) | 0.19 (0-0.5) | 2 (0-5) | 0.21 (0.02-0.56) | 0.07 (-1.44-1.59) |
| Solomon Islands | 239 (129-406) | 36.57 (19.78-62.02) | 253 (123-443) | 25.81 (12.53-45.27) | -1.64 (-2.38--0.9) |
| Somalia | 48304 (25215-76307) | 262.07 (136.66-413.84) | 105023 (49234-179120) | 228.55 (107.26-389.67) | -0.45 (-1.56-0.68) |
| South Africa | 27498 (19490-36581) | 54.78 (38.84-72.88) | 19807 (13869-27305) | 41.91 (29.34-57.77) | -1.6 (-1.83--1.37) |
| South Sudan | 30173 (16570-49741) | 247.18 (135.72-406.91) | 39753 (19515-77237) | 217.82 (107.05-422.56) | -0.34 (-1.31-0.64) |
| Spain | 682 (298-1109) | 3.55 (1.55-5.77) | 157 (24-327) | 0.97 (0.14-2.02) | -3.92 (-5--2.83) |
| Sri Lanka | 10245 (6933-14457) | 59.31 (40.14-83.69) | 1933 (950-3226) | 13.29 (6.53-22.19) | -5.58 (-6.03--5.13) |
| Sudan | 9002 (4742-14993) | 21.63 (11.42-35.95) | 6531 (3210-11165) | 11.85 (5.83-20.26) | -2.59 (-3.37--1.81) |
| Suriname | 342 (175-574) | 78 (39.91-130.76) | 233 (108-407) | 54.63 (25.36-95.24) | -1.84 (-2.05--1.62) |
| Sweden | 79 (25-149) | 1.31 (0.41-2.49) | 19 (2-42) | 0.34 (0.04-0.77) | -4.63 (-5.51--3.74) |
| Switzerland | 78 (24-138) | 1.92 (0.6-3.4) | 34 (2-78) | 0.8 (0.04-1.83) | -3.36 (-4.28--2.43) |
| Syrian Arab Republic | 2496 (1108-4730) | 11.33 (5.03-21.46) | 343 (139-777) | 3.62 (1.46-8.19) | -4.28 (-4.85--3.7) |
| Taiwan (Province of China) | 35 (15-60) | 0.22 (0.1-0.38) | 145 (44-276) | 1.91 (0.58-3.63) | 7.46 (6.18-8.76) |
| Tajikistan | 1823 (815-3427) | 18.24 (8.16-34.27) | 1829 (829-3355) | 13.45 (6.1-24.67) | -1.49 (-2.05--0.91) |
| Thailand | 31831 (16765-54129) | 63.79 (33.6-108.47) | 2915 (1477-4646) | 11.25 (5.7-17.93) | -6.74 (-7.48--5.98) |
| Timor-Leste | 2854 (1635-4529) | 171.57 (98.3-272.03) | 1644 (932-2589) | 83.94 (47.55-132.18) | -3.68 (-4.41--2.95) |
| Togo | 20515 (12910-31860) | 267.78 (168.62-415.48) | 17797 (10342-29150) | 153.02 (88.94-250.57) | -2.25 (-3.06--1.43) |
| Tokelau | 0 (0-0) | 3.58 (0.19-9.86) | 0 (0-0) | 12.41 (0-38.43) | -1.4 (-2.59--0.19) |
| Tonga | 24 (10-43) | 15.24 (6.32-27.58) | 10 (4-19) | 7.13 (2.51-13.55) | -2.64 (-3.18--2.1) |
| Trinidad and Tobago | 330 (126-617) | 28.71 (10.93-53.7) | 192 (73-366) | 26.81 (10.19-51.2) | -0.8 (-1.38--0.23) |
| Tunisia | 540 (245-1037) | 5.13 (2.32-9.85) | 192 (72-450) | 2.38 (0.89-5.59) | -2.93 (-3.22--2.63) |
| T眉rkiye | 23355 (11974-38404) | 32.95 (16.9-54.15) | 4463 (1798-8177) | 9.17 (3.69-16.81) | -5.06 (-5.36--4.75) |
| Turkmenistan | 346 (123-654) | 5.75 (2.05-10.87) | 331 (133-580) | 6.25 (2.51-10.95) | 0.43 (-0.04-0.91) |
| Tuvalu | 2 (1-5) | 13.72 (5.61-28.9) | 0 (0-1) | 2.28 (0.33-5.65) | -6.93 (-7.28--6.59) |
| Uganda | 94308 (61566-141332) | 214.32 (140.04-321.02) | 119666 (76195-190107) | 158.31 (100.82-251.48) | -1.4 (-2.48--0.31) |
| Ukraine | 2490 (1148-4112) | 7.71 (3.55-12.73) | 588 (120-1152) | 4.44 (0.9-8.71) | -2.44 (-3.54--1.33) |
| United Arab Emirates | 42 (14-105) | 1.8 (0.61-4.5) | 34 (15-72) | 0.92 (0.42-1.95) | -2.26 (-3.05--1.46) |
| United Kingdom | 486 (412-571) | 1.27 (1.07-1.49) | 86 (67-110) | 0.26 (0.2-0.33) | -6.3 (-7.05--5.53) |
| United Republic of Tanzania | 133775 (82521-204097) | 232.82 (143.73-354.96) | 182333 (105117-293007) | 201.97 (116.46-324.49) | -0.5 (-1.42-0.44) |
| United States of America | 4198 (3469-4963) | 2.09 (1.73-2.48) | 1509 (1112-1946) | 0.85 (0.63-1.09) | -3.73 (-4.38--3.08) |
| United States Virgin Islands | 3 (1-7) | 2.71 (0.52-6.12) | 1 (0-1) | 1.55 (0.13-3.81) | -2.7 (-3.3--2.09) |
| Uruguay | 351 (132-600) | 13.06 (4.89-22.3) | 63 (14-133) | 3.69 (0.83-7.78) | -5.44 (-5.99--4.9) |
| Uzbekistan | 2977 (1693-5293) | 8.73 (4.97-15.53) | 6432 (3601-9535) | 16.78 (9.39-24.88) | 2.28 (1.87-2.7) |
| Vanuatu | 70 (37-112) | 24.14 (12.63-38.38) | 77 (42-134) | 18.39 (9.91-31.94) | -1.57 (-2.29--0.85) |
| Venezuela (Bolivarian Republic of) | 9469 (4194-15733) | 35.85 (15.87-59.57) | 3517 (1140-6608) | 16.19 (5.25-30.41) | -2.77 (-3.11--2.43) |
| Viet Nam | 101852 (61880-169405) | 109.69 (66.65-182.45) | 19400 (9820-31806) | 25.98 (13.16-42.61) | -5.76 (-5.98--5.54) |
| Yemen | 7728 (4514-12351) | 24.66 (14.42-39.38) | 6922 (3558-11616) | 14.86 (7.64-24.94) | -2.25 (-3.15--1.34) |
| Zambia | 33683 (20148-49329) | 184.58 (110.51-270.08) | 43281 (24314-68908) | 149.45 (83.97-237.95) | -1.08 (-2.06--0.08) |
| Zimbabwe | 17967 (10471-27465) | 96.77 (56.4-147.88) | 23265 (14159-35739) | 103.96 (63.28-159.61) | 0.91 (0.18-1.65) |
| **Particulate matter pollution** |  |  |  |  |  |
| **region_eapc** |  |  |  |  |  |
| Advanced Health System | 60716 (47132-77932) | 6.68 (5.18-8.57) | 14419 (10818-19148) | 2.05 (1.54-2.72) | -4.39 (-5.14--3.63) |
| Africa | 2570165 (2171960-2978937) | 201.07 (169.91-232.97) | 3061427 (2484149-3738270) | 148.41 (120.44-181.22) | -1.35 (-2.15--0.54) |
| African Region | 2499741 (2127539-2887831) | 233.03 (198.29-269.03) | 2942610 (2383139-3573321) | 164.05 (132.86-199.2) | -1.47 (-2.32--0.61) |
| America | 337206 (293349-384139) | 44.02 (38.29-50.15) | 119417 (90197-153149) | 17.52 (13.23-22.47) | -3.64 (-3.93--3.34) |
| Andean Latin America | 72493 (50776-100591) | 128.96 (90.36-178.98) | 19841 (11694-31683) | 33.33 (19.63-53.25) | -4.47 (-4.8--4.13) |
| Asia | 3394384 (2868573-3992521) | 89.1 (75.28-104.8) | 1666534 (1341858-2034136) | 54.56 (43.93-66.6) | -2.74 (-3--2.49) |
| Australasia | 168 (19-379) | 1.1 (0.13-2.48) | 86 (19-178) | 0.5 (0.11-1.03) | -2.85 (-3.69--2.01) |
| Basic Health System | 1013055 (840231-1209641) | 39.47 (32.74-47.13) | 321978 (261528-399024) | 17.09 (13.89-21.18) | -3.73 (-4.1--3.36) |
| Caribbean | 41606 (29506-58515) | 96.43 (68.39-135.59) | 40905 (24874-61928) | 107.25 (65.21-162.36) | -0.19 (-0.37-0) |
| Central Africa | 193515 (143785-256215) | 129.5 (96.26-171.28) | 298328 (203777-417846) | 110.8 (75.7-155.19) | -0.67 (-1.62-0.28) |
| Central Asia | 11064 (8081-15593) | 11.73 (8.57-16.53) | 12078 (8802-15883) | 12.28 (8.95-16.15) | -0.07 (-0.28-0.14) |
| Central Europe | 4330 (3294-5581) | 5.23 (3.98-6.75) | 379 (288-496) | 0.75 (0.57-0.98) | -7.61 (-8.61--6.6) |
| Central Latin America | 101811 (88044-116600) | 42.51 (36.76-48.68) | 39654 (29108-51702) | 21.11 (15.5-27.53) | -3.34 (-3.67--3.01) |
| Central Sub-Saharan Africa | 101864 (64929-154430) | 83.06 (52.99-125.81) | 121420 (65498-211392) | 56.85 (30.66-98.97) | -1.31 (-2.27--0.34) |
| Commonwealth High Income | 1309 (994-1727) | 1.66 (1.26-2.2) | 545 (356-794) | 0.74 (0.48-1.08) | -3.14 (-3.84--2.44) |
| Commonwealth Low Income | 1201785 (918833-1568925) | 280.29 (214.63-365.27) | 780418 (627787-990641) | 166.35 (133.82-211.15) | -2.29 (-2.98--1.6) |
| Commonwealth Middle Income | 2524128 (2159134-2945794) | 141.91 (121.35-165.5) | 2142164 (1756860-2579633) | 108.02 (88.59-130.07) | -1.68 (-2.03--1.33) |
| East Asia | 124425 (95628-162772) | 10.84 (8.33-14.18) | 12194 (8694-15769) | 2.2 (1.57-2.85) | -6.01 (-6.76--5.26) |
| East Asia & Pacific - WB | 837995 (654320-1059946) | 45.44 (35.48-57.48) | 288335 (224393-380738) | 24.54 (19.09-32.4) | -3.31 (-3.86--2.77) |
| Eastern Africa | 940171 (757763-1165157) | 243.12 (196.1-301.14) | 967472 (723608-1226452) | 167.07 (124.96-211.8) | -1.65 (-2.52--0.78) |
| Eastern Europe | 12377 (10104-14680) | 8.6 (7.02-10.2) | 3463 (2741-4273) | 4 (3.17-4.94) | -3.3 (-4.27--2.31) |
| Eastern Mediterranean Region | 359615 (264988-482924) | 56.6 (41.71-76.01) | 429793 (318794-572475) | 52.07 (38.62-69.35) | -1.16 (-1.64--0.67) |
| Eastern Sub-Saharan Africa | 1166573 (977328-1395502) | 272.72 (228.66-326) | 1212522 (915140-1530894) | 184.99 (139.63-233.57) | -1.69 (-2.59--0.78) |
| Europe | 48946 (36871-64025) | 9.22 (6.95-12.06) | 11315 (8252-15377) | 2.95 (2.15-4.01) | -4.19 (-5--3.38) |
| Europe & Central Asia - WB | 55611 (42580-70515) | 9.22 (7.06-11.69) | 21424 (16647-27140) | 4.58 (3.56-5.81) | -2.8 (-3.51--2.08) |
| European Region | 55746 (42716-70649) | 9.14 (7-11.58) | 21508 (16710-27219) | 4.51 (3.5-5.7) | -2.81 (-3.51--2.1) |
| High-income Asia Pacific | 2411 (1307-3870) | 2.53 (1.37-4.06) | 363 (228-553) | 0.63 (0.4-0.96) | -5.6 (-6.71--4.49) |
| High-income North America | 4356 (3589-5136) | 1.98 (1.63-2.33) | 1591 (1180-2056) | 0.81 (0.6-1.05) | -3.68 (-4.34--3.01) |
| Latin America & Caribbean - WB | 332954 (288982-379831) | 60.63 (52.62-69.16) | 117853 (88541-151382) | 24.19 (18.17-31.07) | -3.69 (-3.93--3.46) |
| Limited Health System | 4567356 (3934425-5298052) | 175.44 (151.07-203.32) | 3532111 (2938812-4200127) | 118.39 (98.51-140.78) | -1.99 (-2.44--1.54) |
| Middle East & North Africa - WB | 63417 (46965-84516) | 16.01 (11.86-21.34) | 35099 (24369-46508) | 8.32 (5.78-11.02) | -2.88 (-3.23--2.52) |
| Minimal Health System | 709680 (559693-878927) | 229.82 (181.21-284.39) | 990215 (755978-1286023) | 161.38 (123.22-209.54) | -1.35 (-2.36--0.34) |
| North Africa and Middle East | 101507 (78048-129481) | 19.38 (14.9-24.71) | 62330 (44752-83225) | 10.87 (7.8-14.51) | -2.68 (-3.03--2.33) |
| North America | 4356 (3589-5136) | 1.98 (1.63-2.33) | 1591 (1180-2056) | 0.81 (0.6-1.05) | -3.68 (-4.34--3.01) |
| Northern Africa | 25650 (17305-35924) | 13.81 (9.32-19.34) | 15997 (10808-22674) | 7.37 (4.98-10.45) | -2.51 (-2.91--2.1) |
| Oceania | 3785 (2223-6341) | 35.14 (20.64-58.86) | 7177 (3911-12010) | 34.96 (19.06-58.51) | -0.2 (-0.81-0.41) |
| Region of the Americas | 337206 (293349-384139) | 44.02 (38.29-50.15) | 119417 (90197-153149) | 17.52 (13.23-22.47) | -3.64 (-3.93--3.34) |
| South-East Asia Region | 2669054 (2244398-3191975) | 147.3 (123.84-176.05) | 1199716 (929418-1504303) | 78.15 (60.54-97.99) | -3.13 (-3.39--2.86) |
| South Asia | 2483219 (2078503-3011236) | 151.48 (126.9-183.48) | 1321612 (1043227-1655988) | 87.36 (68.96-109.46) | -2.86 (-3.17--2.55) |
| South Asia - WB | 2500889 (2092991-3027843) | 149.04 (124.84-180.17) | 1341228 (1059395-1675358) | 84.59 (66.81-105.65) | -2.87 (-3.18--2.56) |
| Southeast Asia | 718620 (551009-943308) | 121.42 (93.1-159.39) | 270803 (207428-360576) | 50.16 (38.41-66.79) | -3.65 (-3.89--3.42) |
| Southern Africa | 289369 (227528-359488) | 167.63 (131.85-208.27) | 281018 (206508-380599) | 114.58 (84.2-155.19) | -1.62 (-2.29--0.95) |
| Southern Latin America | 8419 (4825-12411) | 16.59 (9.5-24.46) | 1942 (930-3086) | 5.2 (2.5-8.27) | -5.13 (-5.52--4.74) |
| Southern Sub-Saharan Africa | 53899 (39891-68444) | 69.51 (51.45-88.26) | 48276 (35955-67185) | 61.78 (46.02-85.98) | -0.6 (-0.93--0.27) |
| Sub-Saharan Africa - WB | 2555569 (2164291-2965735) | 233.2 (197.48-270.51) | 3053186 (2476785-3728702) | 164.94 (133.81-201.42) | -1.45 (-2.33--0.57) |
| Tropical Latin America | 110034 (91936-131978) | 68.33 (57.08-81.95) | 16896 (12185-22239) | 10.19 (7.35-13.42) | -6.43 (-6.85--6.01) |
| Western Africa | 1121460 (898244-1350408) | 292.25 (233.96-352.17) | 1498613 (1233356-1819812) | 199.15 (163.93-241.8) | -1.5 (-2.45--0.54) |
| Western Europe | 5256 (4095-6712) | 2.36 (1.83-3.01) | 1540 (1028-2178) | 0.78 (0.52-1.1) | -3.59 (-4.45--2.71) |
| Western Pacific Region | 429302 (351120-525705) | 29.15 (23.84-35.7) | 145505 (116995-177562) | 16.88 (13.57-20.6) | -3.11 (-3.73--2.48) |
| Western Sub-Saharan Africa | 1226283 (995498-1464765) | 287.76 (233.62-343.58) | 1666878 (1367418-2016820) | 196.06 (160.9-237.16) | -1.51 (-2.46--0.54) |
| **country_eapc** |  |  |  |  |  |
| Afghanistan | 7082 (3773-11709) | 33.78 (17.96-55.92) | 17662 (9655-29780) | 30.43 (16.64-51.3) | -0.88 (-1.91-0.16) |
| Albania | 18 (9-31) | 0.45 (0.24-0.8) | 5 (2-11) | 0.38 (0.13-0.8) | -2.75 (-3.71--1.78) |
| Algeria | 1478 (518-3015) | 4 (1.4-8.16) | 977 (293-2685) | 2.23 (0.67-6.14) | -2.42 (-2.68--2.17) |
| American Samoa | 1 (0-4) | 1.7 (0.1-4.34) | 0 (0-1) | 0.61 (0.02-1.8) | -5.85 (-6.5--5.19) |
| Andorra | 0 (0-1) | 1.25 (0.33-2.89) | 0 (0-0) | 0.16 (0-0.51) | -6.02 (-7.32--4.7) |
| Angola | 32316 (18222-52477) | 135.4 (76.38-219.81) | 23701 (12942-40601) | 40.92 (22.35-70.07) | -4.51 (-5.53--3.47) |
| Antigua and Barbuda | 4 (1-8) | 6.82 (1.96-12.96) | 4 (1-7) | 7.67 (2.29-14.32) | -0.54 (-1.02--0.06) |
| Argentina | 6486 (3173-10404) | 19.43 (9.5-31.17) | 1442 (509-2512) | 5.58 (1.96-9.73) | -5.51 (-5.86--5.15) |
| Armenia | 2121 (1149-3437) | 58.4 (31.64-94.65) | 607 (333-960) | 36.49 (20.01-57.65) | -2.8 (-3.38--2.22) |
| Australia | 154 (6-365) | 1.25 (0.05-2.95) | 70 (5-155) | 0.49 (0.03-1.09) | -3.65 (-4.57--2.71) |
| Austria | 52 (21-86) | 1.16 (0.47-1.93) | 15 (2-33) | 0.37 (0.05-0.81) | -3.67 (-4.65--2.68) |
| Azerbaijan | 1409 (657-2662) | 15.86 (7.4-29.96) | 674 (267-1410) | 10.45 (4.14-21.87) | -2.6 (-2.99--2.21) |
| Bahamas | 50 (22-89) | 18.93 (8.37-33.5) | 29 (12-53) | 15.26 (6.22-27.5) | -1.65 (-2.24--1.06) |
| Bahrain | 63 (36-100) | 9.9 (5.65-15.67) | 23 (13-39) | 2.68 (1.53-4.59) | -4.87 (-5.28--4.46) |
| Bangladesh | 642106 (392843-991158) | 317.25 (194.44-487.87) | 181733 (99767-294868) | 135.4 (74.33-219.69) | -3.87 (-4.33--3.41) |
| Barbados | 41 (20-67) | 20.58 (9.82-33.44) | 34 (15-64) | 27.18 (11.71-50.46) | 0.41 (-0.38-1.21) |
| Belarus | 751 (352-1375) | 10.95 (5.13-20.06) | 136 (35-271) | 3.38 (0.86-6.76) | -3.77 (-4.71--2.82) |
| Belgium | 144 (54-250) | 2.39 (0.89-4.14) | 46 (8-100) | 0.84 (0.14-1.82) | -4 (-4.81--3.19) |
| Belize | 119 (66-185) | 39.35 (21.98-61.11) | 98 (48-155) | 26.56 (13.13-42.03) | -1.88 (-2.33--1.42) |
| Benin | 34139 (20028-51478) | 300.78 (176.62-453.15) | 48642 (30225-76649) | 194.23 (120.77-305.96) | -1.58 (-2.58--0.56) |
| Bermuda | 0 (0-1) | 1.1 (0.07-2.57) | 0 (0-0) | 0.33 (0-0.89) | -4.74 (-5.59--3.89) |
| Bhutan | 1063 (638-1722) | 100.26 (60.26-162.12) | 123 (60-224) | 20.52 (9.94-37.43) | -6.29 (-6.65--5.93) |
| Bolivia (Plurinational State of) | 13495 (7917-21515) | 123.68 (72.54-196.99) | 4675 (2231-8264) | 39.9 (19.04-70.54) | -4.58 (-4.96--4.19) |
| Bosnia and Herzegovina | 213 (59-504) | 6.54 (1.83-15.51) | 30 (11-66) | 2.27 (0.81-4.89) | -4.43 (-5.42--3.43) |
| Botswana | 2009 (1232-3114) | 90.46 (55.5-140.16) | 1018 (487-1795) | 43.75 (20.91-77.13) | -2.77 (-3.19--2.36) |
| Brazil | 103994 (86323-125481) | 67.17 (55.75-81.04) | 16157 (11642-21500) | 10.13 (7.3-13.48) | -6.34 (-6.76--5.91) |
| Brunei Darussalam | 11 (2-24) | 3.3 (0.48-7.22) | 10 (2-23) | 3.27 (0.55-7.85) | -1.07 (-1.54--0.59) |
| Bulgaria | 26 (13-41) | 0.54 (0.28-0.86) | 24 (8-41) | 0.84 (0.29-1.45) | 0.81 (-1.02-2.67) |
| Burkina Faso | 69101 (40977-109512) | 312.78 (185.43-494.7) | 103187 (62089-161611) | 227 (136.77-355.35) | -1.09 (-2.14--0.03) |
| Burundi | 31521 (19490-48306) | 253.71 (156.89-388.4) | 41525 (25025-67018) | 185.43 (111.78-299.24) | -1.18 (-2.12--0.24) |
| Cabo Verde | 575 (308-930) | 95.48 (51.09-154.23) | 202 (112-352) | 49.45 (27.55-86.3) | -3.44 (-3.87--3.02) |
| Cambodia | 31220 (17186-49286) | 152.5 (84.04-240.8) | 12088 (6791-19422) | 70.6 (39.66-113.44) | -3.99 (-4.45--3.54) |
| Cameroon | 48151 (29664-73928) | 211.25 (130.13-324.08) | 66376 (39555-103627) | 133.57 (79.6-208.51) | -1.84 (-2.73--0.95) |
| Canada | 158 (27-327) | 0.81 (0.14-1.67) | 82 (0-189) | 0.46 (0-1.06) | -2.32 (-3.12--1.51) |
| Central African Republic | 7067 (4247-12252) | 117.57 (70.84-203.39) | 10787 (5928-18680) | 119.16 (65.48-206.32) | -0.17 (-1.11-0.79) |
| Chad | 43937 (25889-66001) | 288.3 (169.79-432.22) | 92573 (54275-147736) | 224.36 (131.57-357.63) | -0.92 (-2.12-0.29) |
| Chile | 1582 (906-2398) | 10.77 (6.17-16.33) | 436 (189-704) | 4.46 (1.93-7.2) | -3.58 (-4.23--2.92) |
| China | 121478 (92924-158193) | 10.98 (8.4-14.3) | 11128 (7969-14462) | 2.09 (1.5-2.72) | -6.19 (-6.94--5.43) |
| Colombia | 8941 (5157-13307) | 20.22 (11.66-30.09) | 3744 (1447-6841) | 11.54 (4.45-21.09) | -2.33 (-3.02--1.65) |
| Comoros | 2611 (1542-3996) | 277.57 (164.18-424.4) | 1458 (862-2327) | 179.03 (105.93-285.6) | -2.36 (-2.95--1.75) |
| Congo | 3111 (1434-5665) | 71.23 (32.84-129.6) | 3423 (1553-5964) | 56.03 (25.42-97.62) | -1.3 (-2.03--0.56) |
| Cook Islands | 1 (0-1) | 2.89 (0.44-7.03) | 0 (0-0) | 0.95 (0.03-2.72) | -6.15 (-6.71--5.58) |
| Costa Rica | 335 (181-531) | 8.55 (4.62-13.54) | 75 (19-137) | 2.87 (0.73-5.22) | -5.13 (-5.55--4.7) |
| Croatia | 35 (15-55) | 1.29 (0.57-2.07) | 33 (10-65) | 1.95 (0.56-3.84) | -0.52 (-2.19-1.17) |
| Cuba | 770 (294-1330) | 8.97 (3.41-15.5) | 176 (54-316) | 3.65 (1.11-6.53) | -3.58 (-4.37--2.79) |
| Cyprus | 24 (8-50) | 3.59 (1.26-7.49) | 6 (2-12) | 0.8 (0.26-1.64) | -5.97 (-6.63--5.32) |
| Czechia | 53 (26-86) | 0.86 (0.42-1.39) | 30 (8-58) | 0.58 (0.15-1.14) | -0.85 (-2.22-0.54) |
| Democratic People's Republic of Korea | 2912 (1473-5127) | 11.28 (5.71-19.86) | 921 (389-1896) | 6.5 (2.75-13.4) | -2.9 (-3.35--2.45) |
| Democratic Republic of the Congo | 57733 (31039-97271) | 67.35 (36.24-113.34) | 82217 (36185-164639) | 60.17 (26.48-120.48) | -0.13 (-1.11-0.86) |
| Denmark | 14 (5-25) | 0.44 (0.15-0.81) | 6 (0-13) | 0.18 (0.01-0.43) | -3.29 (-4.05--2.52) |
| Djibouti | 1282 (757-2172) | 171.96 (101.59-291.04) | 1408 (678-2422) | 97.2 (46.82-167.27) | -2.15 (-2.73--1.57) |
| Dominica | 43 (22-73) | 47.58 (24.51-81.42) | 18 (8-34) | 56.68 (24.34-109.25) | -0.37 (-0.99-0.26) |
| Dominican Republic | 13624 (8175-21294) | 126.68 (76.02-197.98) | 7084 (3019-12248) | 69.09 (29.44-119.47) | -2.3 (-2.64--1.97) |
| Ecuador | 7687 (4563-11307) | 53.15 (31.56-78.18) | 1486 (578-2804) | 9.62 (3.75-18.15) | -7.33 (-8.01--6.65) |
| Egypt | 7571 (3332-15556) | 8.41 (3.7-17.27) | 4303 (2129-7649) | 3.46 (1.71-6.15) | -2.8 (-3.4--2.2) |
| El Salvador | 5909 (3725-8614) | 70.72 (44.59-103.1) | 558 (238-1079) | 9.96 (4.23-19.26) | -7.13 (-7.49--6.78) |
| Equatorial Guinea | 956 (486-1820) | 97.62 (49.74-185.58) | 640 (306-1127) | 35.62 (17-62.67) | -5.08 (-5.96--4.19) |
| Eritrea | 14290 (8137-23603) | 201.96 (115.04-333.07) | 13334 (7544-20941) | 142.48 (80.6-223.76) | -1.54 (-2.27--0.81) |
| Estonia | 66 (22-113) | 6.32 (2.11-10.93) | 6 (0-15) | 1 (0.05-2.32) | -7.69 (-8.66--6.72) |
| Eswatini | 1200 (672-1990) | 77.57 (43.47-128.69) | 670 (309-1162) | 48.25 (22.29-83.77) | -1.88 (-2.5--1.26) |
| Ethiopia | 439664 (323264-583064) | 379.96 (279.87-503.66) | 340310 (226672-466941) | 204.14 (135.95-280.12) | -2.61 (-3.48--1.74) |
| Fiji | 261 (147-422) | 28.88 (16.26-46.56) | 97 (34-180) | 11.11 (3.87-20.76) | -3.74 (-4.15--3.32) |
| Finland | 16 (1-38) | 0.5 (0.03-1.22) | 6 (0-15) | 0.24 (0-0.63) | -3.14 (-4.01--2.26) |
| France | 1087 (302-2042) | 2.94 (0.82-5.54) | 336 (32-792) | 1.01 (0.1-2.38) | -3.24 (-3.97--2.51) |
| Gabon | 680 (293-1284) | 39.91 (17.27-75.33) | 653 (301-1158) | 31.6 (14.58-56.07) | -0.67 (-1.26--0.07) |
| Gambia | 6674 (4194-10308) | 309.61 (194.68-477.52) | 6823 (4055-10512) | 184.25 (109.52-283.87) | -2.24 (-3.13--1.34) |
| Georgia | 291 (124-493) | 7.01 (2.98-11.88) | 271 (108-464) | 12.58 (4.99-21.5) | 2.14 (0.86-3.43) |
| Germany | 914 (400-1497) | 2.15 (0.94-3.53) | 247 (40-518) | 0.65 (0.11-1.37) | -4.04 (-5.1--2.96) |
| Ghana | 94806 (60302-142621) | 328.86 (209.41-494.03) | 86575 (47749-142968) | 186.47 (102.88-307.92) | -1.96 (-2.63--1.3) |
| Greece | 73 (23-134) | 1.45 (0.46-2.64) | 25 (8-48) | 0.63 (0.2-1.2) | -2.47 (-3.58--1.34) |
| Greenland | 0 (0-1) | 0.36 (0.01-1.16) | 0 (0-0) | 0.19 (0-0.59) | -2.51 (-3.37--1.65) |
| Grenada | 30 (16-48) | 25.71 (13.36-41.77) | 13 (5-24) | 20.73 (7.49-36.57) | -1.25 (-1.6--0.89) |
| Guam | 1 (0-2) | 0.36 (0.05-0.85) | 3 (0-7) | 2.48 (0.33-5.89) | 6.57 (5.13-8.02) |
| Guatemala | 15420 (10108-22407) | 93.01 (60.97-135.16) | 6137 (3657-9307) | 42.95 (25.61-65.13) | -3.83 (-4.38--3.27) |
| Guinea | 44130 (27580-69374) | 318.68 (199-501.31) | 46065 (28544-72411) | 193.69 (120.05-304.31) | -1.88 (-2.83--0.92) |
| Guinea-Bissau | 8847 (5379-13135) | 404.62 (245.98-600.43) | 7240 (4170-12113) | 210.89 (121.52-352.63) | -2.46 (-3.37--1.54) |
| Guyana | 712 (391-1173) | 57 (31.3-93.81) | 366 (162-665) | 50.99 (22.59-92.76) | -1.69 (-2.19--1.19) |
| Haiti | 22786 (12742-36298) | 189 (105.71-301.28) | 30750 (17214-49230) | 187.84 (105.15-300.5) | -0.6 (-1.18--0.02) |
| Honduras | 9078 (5307-14057) | 105.73 (61.81-163.71) | 5845 (2967-9704) | 54.66 (27.74-90.73) | -2.88 (-3.37--2.4) |
| Hungary | 77 (39-125) | 1.27 (0.65-2.06) | 26 (7-51) | 0.61 (0.16-1.19) | -2.73 (-4.02--1.42) |
| Iceland | 1 (0-3) | 0.63 (0.05-1.48) | 0 (0-1) | 0.16 (0-0.41) | -4.62 (-5.1--4.13) |
| India | 1558124 (1285572-1913466) | 130.06 (107.43-159.64) | 853693 (612848-1134005) | 81.36 (58.41-108.07) | -2.71 (-2.99--2.42) |
| Indonesia | 241430 (155216-371460) | 107.48 (69.11-165.34) | 72388 (44725-127353) | 34.15 (21.09-60.08) | -4.51 (-4.78--4.23) |
| Iran (Islamic Republic of) | 5389 (3940-7113) | 7.26 (5.31-9.58) | 1166 (629-1703) | 2.34 (1.26-3.42) | -2.54 (-3.1--1.97) |
| Iraq | 21929 (12558-33740) | 66.24 (37.93-101.92) | 13747 (6953-23429) | 34.47 (17.43-58.77) | -3.17 (-3.73--2.62) |
| Ireland | 17 (4-34) | 0.66 (0.16-1.31) | 8 (0-21) | 0.28 (0-0.76) | -2.98 (-3.53--2.43) |
| Israel | 119 (54-197) | 2.35 (1.07-3.89) | 75 (25-137) | 0.84 (0.28-1.54) | -3.32 (-3.56--3.08) |
| Italy | 859 (689-1043) | 3.18 (2.55-3.86) | 294 (203-407) | 1.51 (1.04-2.09) | -2.17 (-3.34--0.98) |
| Jamaica | 1152 (652-1710) | 41.62 (23.55-61.8) | 460 (210-831) | 28.99 (13.21-52.34) | -1.48 (-2.1--0.86) |
| Japan | 521 (421-625) | 0.87 (0.7-1.04) | 126 (99-156) | 0.3 (0.24-0.38) | -4.95 (-6.14--3.74) |
| Jordan | 1724 (838-2779) | 27 (13.11-43.51) | 1737 (847-2989) | 16.74 (8.16-28.81) | -2.45 (-2.99--1.91) |
| Kazakhstan | 1213 (544-2220) | 6.82 (3.06-12.48) | 746 (291-1375) | 3.78 (1.48-6.98) | -1.75 (-2.05--1.44) |
| Kenya | 79144 (57305-105486) | 167.93 (121.61-223.77) | 73321 (55012-96201) | 129.4 (97.09-169.78) | -1.31 (-2.04--0.59) |
| Kiribati | 39 (23-60) | 30.28 (17.59-46.26) | 20 (11-33) | 14.57 (7.64-23.17) | -3.09 (-3.6--2.58) |
| Kuwait | 156 (85-233) | 9.66 (5.28-14.42) | 113 (61-175) | 4.72 (2.54-7.28) | -2.72 (-3.47--1.97) |
| Kyrgyzstan | 322 (173-568) | 5.08 (2.72-8.95) | 779 (409-1169) | 10.34 (5.43-15.51) | 2.3 (1.92-2.67) |
| Lao People's Democratic Republic | 14444 (7819-24361) | 174.37 (94.39-293.66) | 6849 (3775-11116) | 81.32 (44.82-131.97) | -3.63 (-4.25--3.01) |
| Latvia | 108 (58-168) | 6.11 (3.26-9.49) | 13 (3-25) | 1.57 (0.34-3.05) | -5.48 (-6.49--4.45) |
| Lebanon | 240 (78-513) | 5.87 (1.9-12.55) | 117 (47-232) | 3.06 (1.24-6.09) | -3.09 (-3.39--2.79) |
| Lesotho | 2911 (1514-5170) | 113.77 (59.3-201.89) | 2167 (1218-3413) | 107.16 (60.23-168.91) | -0.66 (-1.09--0.22) |
| Liberia | 17217 (10052-27753) | 321.39 (187.63-518.12) | 13745 (7804-21738) | 176.38 (100.18-278.83) | -2.58 (-3.45--1.69) |
| Libya | 293 (136-523) | 4.68 (2.17-8.37) | 130 (42-407) | 3.37 (1.1-10.54) | -2.64 (-3.04--2.25) |
| Lithuania | 122 (58-193) | 4.49 (2.14-7.1) | 15 (2-34) | 1.34 (0.19-2.99) | -5.1 (-6.04--4.16) |
| Luxembourg | 2 (1-4) | 0.92 (0.26-1.64) | 1 (0-2) | 0.21 (0.01-0.46) | -5.04 (-5.81--4.27) |
| Madagascar | 43401 (27780-65202) | 174.13 (111.54-261.39) | 61941 (37814-98021) | 153 (93.4-242.06) | -0.68 (-1.55-0.2) |
| Malawi | 58606 (35603-87462) | 245.53 (149.35-366.06) | 44337 (28492-66110) | 162.58 (104.48-242.4) | -2.2 (-3.16--1.22) |
| Malaysia | 6329 (3040-10860) | 26.45 (12.7-45.4) | 1577 (568-2898) | 6.85 (2.47-12.6) | -5.41 (-5.87--4.96) |
| Maldives | 343 (175-551) | 80.76 (41.11-129.51) | 21 (6-42) | 7.15 (2.08-14.42) | -10.22 (-10.64--9.81) |
| Mali | 92084 (55606-146995) | 433.31 (262.61-689.64) | 102940 (61516-158814) | 202.19 (120.86-311.68) | -2.72 (-3.83--1.6) |
| Malta | 2 (1-4) | 0.79 (0.3-1.38) | 1 (0-2) | 0.38 (0.05-0.79) | -2.61 (-3.5--1.71) |
| Marshall Islands | 10 (5-18) | 13.48 (6.57-24.61) | 5 (2-10) | 9.57 (4.1-17.65) | -1.75 (-2.23--1.27) |
| Mauritania | 12336 (7684-18280) | 298.43 (186.03-441.56) | 9163 (5096-14703) | 141.23 (78.55-226.62) | -2.78 (-3.58--1.97) |
| Mauritius | 97 (22-177) | 8.72 (1.99-15.89) | 35 (9-70) | 5.68 (1.5-11.36) | -2.29 (-2.99--1.58) |
| Mexico | 42852 (35586-49712) | 35.19 (29.22-40.82) | 16693 (12544-21120) | 18.37 (13.8-23.23) | -3.56 (-3.93--3.2) |
| Micronesia (Federated States of) | 38 (19-66) | 25.28 (12.67-43.49) | 8 (3-15) | 8.51 (3.68-16.99) | -4.69 (-4.99--4.39) |
| Monaco | 0 (0-0) | 0.8 (0.07-2.02) | 0 (0-0) | 0.51 (0.03-1.25) | -2.11 (-3.35--0.86) |
| Mongolia | 562 (265-1066) | 16.23 (7.64-30.79) | 408 (185-723) | 11.09 (5.02-19.67) | -1.69 (-1.99--1.39) |
| Montenegro | 39 (19-67) | 8.07 (3.88-14.04) | 6 (2-13) | 1.75 (0.57-3.78) | -6.11 (-6.84--5.37) |
| Morocco | 3432 (1563-5890) | 9.1 (4.14-15.62) | 1232 (514-2173) | 4.01 (1.68-7.07) | -3.13 (-3.61--2.64) |
| Mozambique | 110866 (66679-160789) | 381.2 (229.52-553.74) | 121423 (70471-192310) | 229.3 (133.04-363.25) | -1.97 (-2.94--1) |
| Myanmar | 129629 (76643-197739) | 242.78 (143.57-370.05) | 64814 (36795-108442) | 126.01 (71.54-210.83) | -2.84 (-3.03--2.66) |
| Namibia | 2315 (1396-3786) | 94.33 (56.91-154.32) | 1349 (650-2388) | 48.98 (23.6-86.7) | -2.32 (-2.83--1.81) |
| Nauru | 1 (0-1) | 3.12 (0.31-8.21) | 0 (0-1) | 2.21 (0.04-5.88) | -2.45 (-3.33--1.55) |
| Nepal | 48517 (27994-76353) | 129.53 (74.98-203.29) | 19532 (10677-32685) | 63.43 (34.67-106.13) | -3.37 (-3.84--2.89) |
| Netherlands | 369 (134-634) | 3.88 (1.4-6.66) | 148 (16-338) | 1.72 (0.19-3.94) | -2.82 (-3.6--2.03) |
| New Zealand | 14 (3-29) | 0.48 (0.12-0.99) | 16 (3-35) | 0.54 (0.09-1.19) | 1.8 (0.8-2.82) |
| Nicaragua | 8837 (5831-12740) | 129.46 (85.44-186.6) | 2768 (1459-4433) | 44.89 (23.66-71.89) | -4.19 (-4.57--3.81) |
| Niger | 71537 (43747-112665) | 336.69 (206.36-529.74) | 122590 (66877-208802) | 216.61 (118.19-368.89) | -2.05 (-3.26--0.82) |
| Nigeria | 497371 (384880-632436) | 255.59 (197.76-325.09) | 789303 (594684-1020817) | 200.04 (150.72-258.73) | -1.04 (-2.01--0.06) |
| Niue | 0 (0-0) | 6.6 (2.38-12.07) | 0 (0-0) | 5.76 (0.21-14.99) | -4.14 (-5--3.27) |
| North Macedonia | 15 (5-28) | 0.9 (0.31-1.72) | 8 (2-17) | 0.9 (0.27-1.89) | -0.13 (-1.22-0.97) |
| Northern Mariana Islands | 1 (0-1) | 1.02 (0.12-2.53) | 0 (0-0) | 0.41 (0.04-0.99) | -3.78 (-4.4--3.14) |
| Norway | 18 (12-26) | 0.63 (0.42-0.89) | 3 (1-5) | 0.11 (0.05-0.2) | -4.9 (-6.34--3.45) |
| Oman | 389 (185-675) | 11.28 (5.35-19.56) | 200 (100-348) | 5.23 (2.63-9.1) | -2.78 (-3.26--2.3) |
| Pakistan | 233409 (156834-343528) | 116.5 (78.34-171.15) | 266531 (183473-390314) | 89.54 (61.65-131.02) | -1.63 (-2.22--1.03) |
| Palau | 0 (0-0) | 1.2 (0.05-2.95) | 0 (0-0) | 0.77 (0.05-1.99) | -2.15 (-2.7--1.6) |
| Palestine | 654 (306-1095) | 15.46 (7.25-25.89) | 576 (279-1004) | 9.94 (4.81-17.34) | -2.21 (-2.94--1.47) |
| Panama | 969 (499-1541) | 34.14 (17.58-54.29) | 316 (86-626) | 9.28 (2.52-18.44) | -4.77 (-5.01--4.52) |
| Papua New Guinea | 2823 (1487-5228) | 39.95 (21.05-73.96) | 6360 (3264-11026) | 38.84 (19.93-67.32) | -0.25 (-0.93-0.44) |
| Paraguay | 6040 (3662-9131) | 97.27 (58.97-147.04) | 739 (282-1379) | 11.9 (4.54-22.21) | -8.34 (-8.87--7.81) |
| Peru | 51310 (32026-77372) | 166.36 (103.88-250.91) | 13680 (6573-24050) | 42.25 (20.28-74.25) | -3.99 (-4.37--3.6) |
| Philippines | 147298 (117737-180544) | 151.91 (121.43-186.2) | 86755 (65752-110262) | 79.45 (60.22-100.98) | -2.57 (-2.99--2.13) |
| Poland | 2906 (2251-3846) | 11.04 (8.55-14.61) | 124 (88-171) | 0.75 (0.54-1.04) | -10.35 (-11.39--9.31) |
| Portugal | 214 (53-402) | 3.87 (0.95-7.25) | 28 (3-68) | 0.7 (0.08-1.71) | -5.35 (-6.37--4.32) |
| Puerto Rico | 100 (15-217) | 3.17 (0.48-6.88) | 26 (0-60) | 2.95 (0-6.81) | -1.72 (-2.83--0.59) |
| Qatar | 16 (9-27) | 2.91 (1.56-4.93) | 17 (9-28) | 0.92 (0.47-1.5) | -4.82 (-5.21--4.43) |
| Republic of C么te d'Ivoire | 79188 (50261-120981) | 299.91 (190.49-457.89) | 80507 (45279-130991) | 177.73 (99.99-289.22) | -2.1 (-2.95--1.24) |
| Republic of Korea | 1855 (779-3345) | 5.73 (2.41-10.33) | 219 (81-404) | 1.7 (0.62-3.13) | -5.61 (-6.67--4.53) |
| Republic of Moldova | 1759 (1057-2569) | 46.12 (27.71-67.39) | 269 (108-502) | 19.75 (7.91-36.91) | -4.37 (-5.34--3.4) |
| Romania | 288 (155-456) | 1.98 (1.06-3.12) | 27 (11-47) | 0.32 (0.12-0.55) | -7.01 (-8.13--5.88) |
| Russian Federation | 7081 (6083-8032) | 7.42 (6.37-8.42) | 2436 (1948-2953) | 3.73 (2.98-4.52) | -3.11 (-4.11--2.11) |
| Rwanda | 44111 (28036-64309) | 290.65 (184.83-423.77) | 22353 (12859-34281) | 126.23 (72.62-193.6) | -3.28 (-4.06--2.49) |
| Saint Kitts and Nevis | 1 (0-3) | 3.14 (1-5.93) | 1 (0-2) | 2.46 (0.17-5.68) | -1.91 (-2.49--1.32) |
| Saint Lucia | 42 (25-68) | 24.69 (14.58-39.79) | 18 (8-33) | 22.49 (9.34-40.91) | -1.36 (-2--0.71) |
| Saint Vincent and the Grenadines | 47 (26-72) | 38.05 (21.19-58.14) | 17 (7-30) | 27.63 (11.35-47.52) | -1.68 (-2.14--1.23) |
| Samoa | 32 (15-61) | 12.41 (5.91-23.39) | 20 (9-37) | 6.69 (3.01-12.41) | -1.95 (-2.46--1.44) |
| San Marino | 0 (0-1) | 3.06 (0.63-6.73) | 0 (0-0) | 0.63 (0.04-2.07) | -5.1 (-6.1--4.08) |
| Sao Tome and Principe | 358 (212-574) | 161.78 (95.63-259.28) | 133 (63-240) | 56.22 (26.51-101.11) | -4.18 (-4.92--3.43) |
| Saudi Arabia | 7874 (3723-14032) | 32.07 (15.17-57.15) | 1788 (872-3078) | 7.99 (3.89-13.76) | -6.23 (-6.74--5.71) |
| Senegal | 46406 (28470-68019) | 281.32 (172.82-412.66) | 37743 (22637-58879) | 163.77 (98.24-255.47) | -2.03 (-2.85--1.2) |
| Serbia | 568 (270-1025) | 8.58 (4.08-15.49) | 51 (21-84) | 1.58 (0.65-2.6) | -7.79 (-8.88--6.68) |
| Seychelles | 8 (2-19) | 10.54 (1.9-23.82) | 6 (1-14) | 8.03 (1.19-18.21) | -1.78 (-2.12--1.45) |
| Sierra Leone | 38870 (22345-61565) | 402.16 (231.09-635.82) | 35253 (21921-56022) | 244.07 (151.91-388.02) | -2.01 (-2.95--1.06) |
| Singapore | 24 (13-38) | 1.01 (0.53-1.55) | 8 (3-14) | 0.3 (0.1-0.52) | -4.61 (-5.49--3.72) |
| Slovakia | 22 (10-37) | 0.58 (0.26-0.98) | 7 (2-14) | 0.25 (0.08-0.51) | -3.14 (-4.11--2.17) |
| Slovenia | 2 (0-5) | 0.19 (0-0.5) | 2 (0-5) | 0.21 (0.02-0.56) | 0.07 (-1.44-1.59) |
| Solomon Islands | 239 (129-406) | 36.57 (19.78-62.02) | 253 (123-443) | 25.81 (12.53-45.27) | -1.64 (-2.38--0.9) |
| Somalia | 48304 (25215-76307) | 262.07 (136.66-413.84) | 105023 (49234-179120) | 228.55 (107.26-389.67) | -0.45 (-1.56-0.68) |
| South Africa | 27498 (19490-36581) | 54.78 (38.84-72.88) | 19807 (13869-27305) | 41.91 (29.34-57.77) | -1.6 (-1.83--1.37) |
| South Sudan | 30173 (16570-49741) | 247.18 (135.72-406.91) | 39753 (19515-77237) | 217.82 (107.05-422.56) | -0.34 (-1.31-0.64) |
| Spain | 682 (298-1109) | 3.55 (1.55-5.77) | 157 (24-327) | 0.97 (0.14-2.02) | -3.92 (-5--2.83) |
| Sri Lanka | 10245 (6933-14457) | 59.31 (40.14-83.69) | 1933 (950-3226) | 13.29 (6.53-22.19) | -5.58 (-6.03--5.13) |
| Sudan | 9002 (4742-14993) | 21.63 (11.42-35.95) | 6531 (3210-11165) | 11.85 (5.83-20.26) | -2.59 (-3.37--1.81) |
| Suriname | 342 (175-574) | 78 (39.91-130.76) | 233 (108-407) | 54.63 (25.36-95.24) | -1.84 (-2.05--1.62) |
| Sweden | 79 (25-149) | 1.31 (0.41-2.49) | 19 (2-42) | 0.34 (0.04-0.77) | -4.63 (-5.51--3.74) |
| Switzerland | 78 (24-138) | 1.92 (0.6-3.4) | 34 (2-78) | 0.8 (0.04-1.83) | -3.36 (-4.28--2.43) |
| Syrian Arab Republic | 2496 (1108-4730) | 11.33 (5.03-21.46) | 343 (139-777) | 3.62 (1.46-8.19) | -4.28 (-4.85--3.7) |
| Taiwan (Province of China) | 35 (15-60) | 0.22 (0.1-0.38) | 145 (44-276) | 1.91 (0.58-3.63) | 7.46 (6.18-8.76) |
| Tajikistan | 1823 (815-3427) | 18.24 (8.16-34.27) | 1829 (829-3355) | 13.45 (6.1-24.67) | -1.49 (-2.05--0.91) |
| Thailand | 31831 (16765-54129) | 63.79 (33.6-108.47) | 2915 (1477-4646) | 11.25 (5.7-17.93) | -6.74 (-7.48--5.98) |
| Timor-Leste | 2854 (1635-4529) | 171.57 (98.3-272.03) | 1644 (932-2589) | 83.94 (47.55-132.18) | -3.68 (-4.41--2.95) |
| Togo | 20515 (12910-31860) | 267.78 (168.62-415.48) | 17797 (10342-29150) | 153.02 (88.94-250.57) | -2.25 (-3.06--1.43) |
| Tokelau | 0 (0-0) | 3.58 (0.19-9.86) | 0 (0-0) | 12.41 (0-38.43) | -1.4 (-2.59--0.19) |
| Tonga | 24 (10-43) | 15.24 (6.32-27.58) | 10 (4-19) | 7.13 (2.51-13.55) | -2.64 (-3.18--2.1) |
| Trinidad and Tobago | 330 (126-617) | 28.71 (10.93-53.7) | 192 (73-366) | 26.81 (10.19-51.2) | -0.8 (-1.38--0.23) |
| Tunisia | 540 (245-1037) | 5.13 (2.32-9.85) | 192 (72-450) | 2.38 (0.89-5.59) | -2.93 (-3.22--2.63) |
| Turkey | 23355 (11974-38404) | 32.95 (16.9-54.15) | 4463 (1798-8177) | 9.17 (3.69-16.81) | -5.06 (-5.36--4.75) |
| Turkmenistan | 346 (123-654) | 5.75 (2.05-10.87) | 331 (133-580) | 6.25 (2.51-10.95) | 0.43 (-0.04-0.91) |
| Tuvalu | 2 (1-5) | 13.72 (5.61-28.9) | 0 (0-1) | 2.28 (0.33-5.65) | -6.93 (-7.28--6.59) |
| Uganda | 94308 (61566-141332) | 214.32 (140.04-321.02) | 119666 (76195-190107) | 158.31 (100.82-251.48) | -1.4 (-2.48--0.31) |
| Ukraine | 2490 (1148-4112) | 7.71 (3.55-12.73) | 588 (120-1152) | 4.44 (0.9-8.71) | -2.44 (-3.54--1.33) |
| United Arab Emirates | 42 (14-105) | 1.8 (0.61-4.5) | 34 (15-72) | 0.92 (0.42-1.95) | -2.26 (-3.05--1.46) |
| United Kingdom | 486 (412-571) | 1.27 (1.07-1.49) | 86 (67-110) | 0.26 (0.2-0.33) | -6.3 (-7.05--5.53) |
| United Republic of Tanzania | 133775 (82521-204097) | 232.82 (143.73-354.96) | 182333 (105117-293007) | 201.97 (116.46-324.49) | -0.5 (-1.42-0.44) |
| United States of America | 4198 (3469-4963) | 2.09 (1.73-2.48) | 1509 (1112-1946) | 0.85 (0.63-1.09) | -3.73 (-4.38--3.08) |
| United States Virgin Islands | 3 (1-7) | 2.71 (0.52-6.12) | 1 (0-1) | 1.55 (0.13-3.81) | -2.7 (-3.3--2.09) |
| Uruguay | 351 (132-600) | 13.06 (4.89-22.3) | 63 (14-133) | 3.69 (0.83-7.78) | -5.44 (-5.99--4.9) |
| Uzbekistan | 2977 (1693-5293) | 8.73 (4.97-15.53) | 6432 (3601-9535) | 16.78 (9.39-24.88) | 2.28 (1.87-2.7) |
| Vanuatu | 70 (37-112) | 24.14 (12.63-38.38) | 77 (42-134) | 18.39 (9.91-31.94) | -1.57 (-2.29--0.85) |
| Venezuela (Bolivarian Republic of) | 9469 (4194-15733) | 35.85 (15.87-59.57) | 3517 (1140-6608) | 16.19 (5.25-30.41) | -2.77 (-3.11--2.43) |
| Viet Nam | 101852 (61880-169405) | 109.69 (66.65-182.45) | 19400 (9820-31806) | 25.98 (13.16-42.61) | -5.76 (-5.98--5.54) |
| Yemen | 7728 (4514-12351) | 24.66 (14.42-39.38) | 6922 (3558-11616) | 14.86 (7.64-24.94) | -2.25 (-3.15--1.34) |
| Zambia | 33683 (20148-49329) | 184.58 (110.51-270.08) | 43281 (24314-68908) | 149.45 (83.97-237.95) | -1.08 (-2.06--0.08) |
| Zimbabwe | 17967 (10471-27465) | 96.77 (56.4-147.88) | 23265 (14159-35739) | 103.96 (63.28-159.61) | 0.91 (0.18-1.65) |
| **Household air pollution from solid fuels** |  |  |  |  |  |
| **region_eapc** | |  |  |  |  |
| Advanced Health System | 9195 (1999-24259) | 1.01 (0.22-2.67) | 174 (9-1073) | 0.02 (0-0.15) | -12.9 (-13.74--12.05) |
| Africa | 2223406 (1870335-2604883) | 173.94 (146.34-203.64) | 2513171 (1960934-3158365) | 121.83 (95.06-153.09) | -1.66 (-2.46--0.85) |
| African Region | 2164581 (1832316-2540861) | 201.78 (170.83-236.67) | 2409204 (1884980-3016440) | 134.31 (105.1-168.16) | -1.79 (-2.65--0.93) |
| America | 199098 (141425-265762) | 25.99 (18.46-34.69) | 51885 (31486-81490) | 7.61 (4.62-11.96) | -4.78 (-5.06--4.51) |
| Andean Latin America | 36426 (18258-60067) | 64.8 (32.48-106.83) | 3981 (725-12095) | 6.69 (1.22-20.32) | -7.15 (-7.68--6.61) |
| Asia | 2875672 (2387249-3443277) | 75.48 (62.66-90.35) | 1115907 (815252-1472607) | 36.53 (26.69-48.21) | -3.54 (-3.83--3.25) |
| Australasia | 3 (0-30) | 0.02 (0-0.2) | 0 (0-0) | 0 (0-0) | -12.57 (-13.36--11.79) |
| Basic Health System | 717790 (554021-904419) | 27.97 (21.59-35.24) | 123212 (69318-203598) | 6.54 (3.68-10.81) | -5.72 (-6.15--5.28) |
| Caribbean | 34445 (22150-51281) | 79.83 (51.33-118.84) | 30328 (17197-47940) | 79.51 (45.09-125.69) | -0.64 (-0.84--0.44) |
| Central Africa | 170744 (127310-226338) | 114.26 (85.22-151.24) | 259780 (173382-371408) | 96.48 (64.39-137.93) | -0.77 (-1.72-0.19) |
| Central Asia | 6081 (3073-9765) | 6.45 (3.26-10.35) | 3203 (1385-6744) | 3.26 (1.41-6.85) | -3.27 (-3.77--2.76) |
| Central Europe | 1528 (444-3051) | 1.85 (0.54-3.69) | 27 (2-145) | 0.05 (0-0.29) | -13.29 (-14.33--12.23) |
| Central Latin America | 47429 (28757-73456) | 19.8 (12.01-30.67) | 14235 (7229-25557) | 7.58 (3.85-13.61) | -4.38 (-4.7--4.06) |
| Central Sub-Saharan Africa | 92104 (59472-141941) | 75.1 (48.56-115.61) | 101866 (50394-185358) | 47.69 (23.6-86.77) | -1.61 (-2.57--0.64) |
| Commonwealth High Income | 12 (0-90) | 0.01 (0-0.11) | 0 (0-3) | 0 (0-0) | -12.1 (-12.84--11.36) |
| Commonwealth Low Income | 1113551 (849775-1457020) | 259.71 (198.23-339.05) | 705244 (560139-908207) | 150.33 (119.4-193.59) | -2.41 (-3.09--1.71) |
| Commonwealth Middle Income | 2074198 (1736002-2489761) | 116.61 (97.63-139.92) | 1448982 (1044451-1945650) | 73.07 (52.67-98.1) | -2.49 (-2.85--2.12) |
| East Asia | 104346 (75853-138892) | 9.09 (6.61-12.1) | 3250 (1237-7537) | 0.59 (0.22-1.36) | -10.05 (-10.78--9.31) |
| East Asia & Pacific - WB | 699695 (534459-899531) | 37.94 (28.98-48.78) | 175519 (113781-253940) | 14.94 (9.68-21.61) | -4.29 (-4.86--3.72) |
| Eastern Africa | 883444 (712948-1094611) | 228.45 (184.62-282.78) | 889279 (661447-1138603) | 153.57 (114.22-196.62) | -1.78 (-2.65--0.9) |
| Eastern Europe | 2026 (881-4244) | 1.41 (0.61-2.95) | 218 (47-732) | 0.25 (0.05-0.85) | -7.72 (-8.86--6.55) |
| Eastern Mediterranean Region | 265699 (183936-379745) | 41.82 (28.95-59.72) | 294752 (203124-410396) | 35.71 (24.61-49.71) | -1.54 (-2.03--1.05) |
| Eastern Sub-Saharan Africa | 1098554 (921383-1320205) | 256.82 (215.48-308.37) | 1119868 (838154-1420237) | 170.86 (127.88-216.68) | -1.8 (-2.7--0.89) |
| Europe | 11159 (3322-26295) | 2.1 (0.63-4.95) | 412 (73-1498) | 0.11 (0.02-0.39) | -10.82 (-11.77--9.86) |
| Europe & Central Asia - WB | 14950 (5596-31030) | 2.48 (0.93-5.15) | 3355 (1458-7573) | 0.72 (0.31-1.62) | -5.23 (-5.96--4.49) |
| European Region | 14956 (5597-31047) | 2.45 (0.92-5.09) | 3356 (1458-7578) | 0.7 (0.31-1.59) | -5.24 (-5.97--4.51) |
| High-income Asia Pacific | 95 (5-421) | 0.1 (0.01-0.44) | 0 (0-1) | 0 (0-0) | -18.18 (-19.44--16.9) |
| High-income North America | 4 (0-34) | 0 (0-0.02) | 1 (0-5) | 0 (0-0) | -6.22 (-6.85--5.59) |
| Latin America & Caribbean - WB | 199095 (141425-265715) | 36.26 (25.75-48.39) | 51884 (31486-81490) | 10.65 (6.46-16.73) | -4.84 (-5.05--4.62) |
| Limited Health System | 3952000 (3384283-4672938) | 151.8 (130.05-179.49) | 2677717 (2084683-3369643) | 89.75 (69.88-112.95) | -2.53 (-2.98--2.07) |
| Middle East & North Africa - WB | 16422 (8998-28237) | 4.15 (2.27-7.13) | 4797 (2429-8380) | 1.14 (0.58-1.99) | -5.97 (-6.36--5.57) |
| Minimal Health System | 630350 (498877-783357) | 204.14 (161.62-253.41) | 880272 (659090-1160197) | 143.47 (107.41-189.09) | -1.41 (-2.41--0.39) |
| North Africa and Middle East | 34993 (20755-57002) | 6.68 (3.96-10.88) | 23279 (14743-36313) | 4.06 (2.57-6.33) | -3.1 (-3.46--2.74) |
| North America | 4 (0-34) | 0 (0-0.02) | 1 (0-5) | 0 (0-0) | -6.23 (-6.86--5.59) |
| Northern Africa | 13603 (8483-20273) | 7.32 (4.57-10.91) | 5341 (2597-8986) | 2.46 (1.2-4.15) | -4.3 (-4.67--3.92) |
| Oceania | 3516 (2029-5866) | 32.64 (18.84-54.45) | 6484 (3418-10919) | 31.59 (16.65-53.19) | -0.28 (-0.89-0.34) |
| Region of the Americas | 199098 (141425-265762) | 25.99 (18.46-34.69) | 51885 (31486-81490) | 7.61 (4.62-11.96) | -4.78 (-5.06--4.51) |
| South-East Asia Region | 2304041 (1884202-2818296) | 127.16 (103.99-155.39) | 828862 (603085-1111959) | 53.99 (39.29-72.44) | -3.86 (-4.15--3.57) |
| South Asia | 2145764 (1764443-2666835) | 130.89 (107.68-162.65) | 917124 (661754-1210250) | 60.63 (43.75-80) | -3.61 (-3.93--3.28) |
| South Asia - WB | 2161247 (1782141-2683703) | 128.8 (106.2-159.91) | 933382 (676781-1231340) | 58.86 (42.68-77.65) | -3.62 (-3.94--3.29) |
| Southeast Asia | 601678 (451464-791392) | 101.66 (76.28-133.72) | 167006 (107379-243125) | 30.93 (19.89-45.03) | -4.52 (-4.81--4.23) |
| Southern Africa | 258621 (198194-326205) | 149.81 (114.82-188.99) | 235018 (169517-327014) | 95.83 (69.13-133.33) | -1.9 (-2.57--1.22) |
| Southern Latin America | 2096 (552-5365) | 4.13 (1.09-10.57) | 51 (0-444) | 0.14 (0-1.19) | -12.13 (-12.56--11.7) |
| Southern Sub-Saharan Africa | 37706 (25133-50594) | 48.63 (32.41-65.25) | 28115 (17865-43073) | 35.98 (22.86-55.13) | -1.22 (-1.55--0.89) |
| Sub-Saharan Africa - WB | 2217917 (1865151-2598901) | 202.38 (170.23-236.96) | 2512437 (1960375-3156813) | 135.73 (105.9-170.51) | -1.76 (-2.64--0.88) |
| Tropical Latin America | 79865 (56544-103585) | 49.59 (35.11-64.32) | 4316 (1572-8485) | 2.6 (0.95-5.12) | -9.58 (-10.07--9.08) |
| Western Africa | 896993 (698367-1097849) | 233.75 (181.95-285.97) | 1123753 (847999-1449770) | 149.33 (112.69-192.65) | -1.96 (-2.91--1) |
| Western Europe | 54 (1-379) | 0.02 (0-0.17) | 1 (0-11) | 0 (0-0.01) | -11.34 (-12.29--10.38) |
| Western Pacific Region | 360952 (286964-455882) | 24.51 (19.49-30.96) | 93315 (65257-126997) | 10.83 (7.57-14.73) | -4.05 (-4.69--3.41) |
| Western Sub-Saharan Africa | 983743 (767792-1197087) | 230.84 (180.16-281.04) | 1260584 (961080-1611591) | 148.27 (113.04-189.54) | -1.93 (-2.89--0.97) |
| **country_eapc** | |  |  |  |  |
| Afghanistan | 6176 (3316-10010) | 29.46 (15.79-47.83) | 15442 (8188-25678) | 26.6 (14.11-44.24) | -0.94 (-1.97-0.11) |
| Albania | 15 (7-26) | 0.37 (0.17-0.66) | 1 (0-5) | 0.1 (0.01-0.35) | -6.88 (-8.07--5.67) |
| Algeria | 123 (9-521) | 0.33 (0.03-1.41) | 2 (0-12) | 0.01 (0-0.03) | -15.02 (-15.59--14.45) |
| American Samoa | 0 (0-1) | 0.24 (0-1.42) | 0 (0-0) | 0.12 (0-0.65) | -4.88 (-5.53--4.23) |
| Andorra | 0 (0-0) | 0 (0-0) | 0 (0-0) | 0 (0-0) | -9.14 (-10.49--7.78) |
| Angola | 28439 (16047-46587) | 119.16 (67.31-195.19) | 12843 (4192-26104) | 22.17 (7.24-45.05) | -6.31 (-7.37--5.24) |
| Antigua and Barbuda | 0 (0-1) | 0.35 (0.01-1.98) | 0 (0-0) | 0.04 (0-0.29) | -7.66 (-8.14--7.17) |
| Argentina | 1050 (95-3766) | 3.14 (0.28-11.28) | 31 (0-260) | 0.12 (0-1.01) | -11.84 (-12.28--11.41) |
| Armenia | 937 (328-1927) | 25.8 (9.04-53.06) | 31 (3-112) | 1.86 (0.17-6.76) | -11.61 (-12.78--10.41) |
| Australia | 3 (0-29) | 0.02 (0-0.23) | 0 (0-0) | 0 (0-0) | -13.98 (-14.9--13.04) |
| Austria | 0 (0-1) | 0 (0-0.02) | 0 (0-0) | 0 (0-0) | -8.96 (-10--7.92) |
| Azerbaijan | 720 (175-1621) | 8.1 (1.97-18.25) | 32 (0-169) | 0.49 (0.01-2.62) | -12.46 (-13.82--11.08) |
| Bahamas | 1 (0-5) | 0.2 (0-1.92) | 0 (0-1) | 0.05 (0-0.49) | -5.73 (-6.33--5.13) |
| Bahrain | 0 (0-3) | 0.05 (0-0.45) | 0 (0-0) | 0 (0-0) | -14.9 (-15.25--14.55) |
| Bangladesh | 587040 (356024-898556) | 290.04 (175.9-442.52) | 155005 (84937-250181) | 115.48 (63.28-186.39) | -4.09 (-4.56--3.63) |
| Barbados | 0 (0-1) | 0.07 (0-0.39) | 0 (0-0) | 0.03 (0-0.16) | -3.41 (-4.22--2.61) |
| Belarus | 34 (2-148) | 0.5 (0.03-2.16) | 1 (0-10) | 0.02 (0-0.24) | -11.59 (-12.89--10.27) |
| Belgium | 0 (0-3) | 0.01 (0-0.04) | 0 (0-0) | 0 (0-0) | -10.29 (-11.15--9.43) |
| Belize | 67 (23-132) | 22.01 (7.49-43.63) | 24 (4-63) | 6.39 (0.99-17.04) | -4.49 (-4.99--3.98) |
| Benin | 28992 (17076-43316) | 255.43 (150.59-381.6) | 41346 (26111-66122) | 165.1 (104.27-263.91) | -1.7 (-2.7--0.69) |
| Bermuda | 0 (0-0) | 0.02 (0-0.16) | 0 (0-0) | 0 (0-0) | -11.91 (-12.8--11.02) |
| Bhutan | 943 (574-1554) | 88.95 (54.06-146.13) | 34 (7-93) | 5.63 (1.13-15.58) | -10.17 (-10.73--9.6) |
| Bolivia (Plurinational State of) | 8081 (4028-13851) | 74.06 (36.95-126.8) | 1847 (514-3978) | 15.76 (4.38-33.96) | -5.56 (-5.99--5.13) |
| Bosnia and Herzegovina | 185 (52-441) | 5.68 (1.59-13.55) | 8 (1-27) | 0.61 (0.06-2.02) | -8.79 (-9.77--7.8) |
| Botswana | 1565 (902-2542) | 70.49 (40.62-114.44) | 186 (6-732) | 7.97 (0.26-31.46) | -7.73 (-8.16--7.3) |
| Brazil | 74887 (52746-97258) | 48.37 (34.07-62.82) | 3956 (1444-7828) | 2.48 (0.91-4.91) | -9.56 (-10.05--9.08) |
| Brunei Darussalam | 0 (0-0) | 0.01 (0-0.03) | 0 (0-0) | 0 (0-0.01) | -5.2 (-5.77--4.63) |
| Bulgaria | 5 (1-16) | 0.11 (0.01-0.34) | 2 (0-10) | 0.06 (0-0.37) | -3.75 (-5.95--1.5) |
| Burkina Faso | 60361 (35035-96945) | 273.22 (158.72-437.8) | 88728 (52451-139661) | 195.2 (115.47-307.04) | -1.25 (-2.3--0.19) |
| Burundi | 29366 (18230-45773) | 236.36 (146.79-368.21) | 39172 (23232-63522) | 174.92 (103.76-283.56) | -1.14 (-2.07--0.2) |
| Cabo Verde | 481 (252-768) | 79.85 (41.8-127.37) | 62 (17-141) | 15.09 (4.13-34.5) | -7.05 (-7.52--6.57) |
| Cambodia | 28857 (15577-45931) | 140.96 (76.07-224.43) | 10464 (5987-17296) | 61.11 (34.96-101.01) | -4.2 (-4.65--3.75) |
| Cameroon | 38988 (23133-60815) | 171.05 (101.57-266.6) | 52938 (29092-84217) | 106.53 (58.55-169.42) | -2.03 (-2.92--1.13) |
| Canada | 1 (0-5) | 0 (0-0.03) | 0 (0-0) | 0 (0-0) | -12.1 (-13.12--11.06) |
| Central African Republic | 6528 (3882-11350) | 108.6 (64.61-188.42) | 9993 (5494-17334) | 110.4 (60.73-191.56) | -0.17 (-1.12-0.78) |
| Chad | 38397 (22549-57875) | 251.95 (148.09-379.02) | 78541 (45488-127987) | 190.35 (110.36-310.08) | -1.12 (-2.32-0.09) |
| Chile | 961 (377-1678) | 6.55 (2.56-11.43) | 18 (0-173) | 0.18 (0-1.77) | -12.49 (-13.04--11.94) |
| China | 101855 (73957-135915) | 9.2 (6.68-12.28) | 2420 (614-6549) | 0.45 (0.12-1.23) | -10.7 (-11.46--9.93) |
| Colombia | 4133 (1279-7796) | 9.35 (2.89-17.63) | 419 (20-1845) | 1.29 (0.06-5.68) | -6.79 (-7.77--5.8) |
| Comoros | 2498 (1475-3803) | 265.52 (157.12-403.87) | 1388 (811-2223) | 170.51 (99.6-272.89) | -2.39 (-2.98--1.78) |
| Congo | 2660 (1209-4909) | 60.89 (27.7-112.33) | 2261 (955-4340) | 37.02 (15.63-71.05) | -2.29 (-3.05--1.52) |
| Cook Islands | 0 (0-1) | 0.78 (0.02-3.5) | 0 (0-0) | 0.03 (0-0.34) | -12.48 (-12.98--11.98) |
| Costa Rica | 152 (41-302) | 3.88 (1.03-7.7) | 5 (0-29) | 0.2 (0-1.11) | -11.14 (-11.55--10.73) |
| Croatia | 4 (0-19) | 0.14 (0-0.7) | 1 (0-5) | 0.04 (0-0.31) | -8.04 (-9.74--6.3) |
| Cuba | 139 (18-424) | 1.62 (0.21-4.95) | 9 (1-36) | 0.18 (0.02-0.74) | -8.77 (-9.65--7.89) |
| Cyprus | 0 (0-1) | 0.03 (0-0.2) | 0 (0-0) | 0 (0-0) | -14.87 (-15.78--13.94) |
| Czechia | 1 (0-5) | 0.01 (0-0.09) | 0 (0-1) | 0 (0-0.02) | -6.51 (-7.88--5.13) |
| Democratic People's Republic of Korea | 2484 (1222-4391) | 9.62 (4.73-17.01) | 829 (346-1763) | 5.85 (2.44-12.46) | -2.79 (-3.24--2.34) |
| Democratic Republic of the Congo | 53478 (28792-90444) | 62.38 (33.63-105.51) | 76712 (33605-154169) | 56.14 (24.59-112.82) | -0.13 (-1.11-0.87) |
| Denmark | 0 (0-0) | 0 (0-0) | 0 (0-0) | 0 (0-0) | -8.52 (-9.44--7.6) |
| Djibouti | 890 (442-1489) | 119.3 (59.37-199.6) | 626 (242-1278) | 43.24 (16.7-88.25) | -3.82 (-4.38--3.26) |
| Dominica | 28 (10-50) | 30.7 (11.58-56.27) | 2 (0-8) | 7.42 (0.53-26.74) | -5.87 (-6.44--5.29) |
| Dominican Republic | 10255 (5341-17130) | 95.35 (49.67-159.19) | 533 (14-3159) | 5.2 (0.13-30.81) | -10.04 (-10.39--9.69) |
| Ecuador | 2804 (779-5929) | 19.39 (5.39-40.99) | 153 (10-647) | 0.99 (0.07-4.19) | -11.2 (-11.86--10.53) |
| Egypt | 2296 (701-5859) | 2.55 (0.78-6.5) | 8 (0-37) | 0.01 (0-0.03) | -19.66 (-20.25--19.07) |
| El Salvador | 4661 (2886-7084) | 55.79 (34.54-84.79) | 187 (42-503) | 3.33 (0.75-8.97) | -9.88 (-10.32--9.43) |
| Equatorial Guinea | 855 (430-1639) | 87.25 (43.83-167.08) | 18 (0-178) | 1.01 (0-9.89) | -19.29 (-20.87--17.68) |
| Eritrea | 12806 (7338-21378) | 180.99 (103.77-301.83) | 11534 (6360-17815) | 123.24 (67.95-190.36) | -1.63 (-2.36--0.9) |
| Estonia | 18 (2-55) | 1.72 (0.16-5.35) | 0 (0-2) | 0.03 (0-0.28) | -15.11 (-16.18--14.02) |
| Eswatini | 972 (540-1722) | 62.83 (34.88-111.32) | 365 (102-790) | 26.33 (7.36-56.92) | -3.02 (-3.64--2.4) |
| Ethiopia | 417605 (308261-554017) | 360.9 (266.79-477.7) | 315451 (211181-438363) | 189.23 (126.67-262.98) | -2.73 (-3.6--1.86) |
| Fiji | 226 (120-374) | 25 (13.2-41.31) | 46 (8-110) | 5.33 (0.92-12.66) | -5.49 (-5.96--5.02) |
| Finland | 0 (0-0) | 0 (0-0.01) | 0 (0-0) | 0 (0-0) | -9.52 (-10.44--8.59) |
| France | 3 (0-28) | 0.01 (0-0.07) | 0 (0-1) | 0 (0-0) | -9.07 (-9.93--8.2) |
| Gabon | 144 (6-579) | 8.45 (0.38-33.97) | 38 (1-219) | 1.86 (0.03-10.58) | -5.42 (-6.02--4.82) |
| Gambia | 5527 (3334-8387) | 256.43 (154.81-388.72) | 5730 (3332-8848) | 154.73 (89.99-238.89) | -2.23 (-3.12--1.33) |
| Georgia | 133 (24-314) | 3.21 (0.57-7.57) | 78 (12-235) | 3.62 (0.55-10.87) | -0.67 (-2.51-1.2) |
| Germany | 1 (0-4) | 0 (0-0.01) | 0 (0-0) | 0 (0-0) | -8.08 (-9.13--7.01) |
| Ghana | 77015 (47612-118521) | 267.15 (165.43-410.75) | 57593 (29407-98643) | 124.04 (63.34-212.44) | -2.8 (-3.47--2.12) |
| Greece | 1 (0-12) | 0.03 (0-0.23) | 0 (0-1) | 0 (0-0.02) | -9.87 (-11.37--8.34) |
| Greenland | 0 (0-0) | 0 (0-0) | 0 (0-0) | 0 (0-0) | -9.45 (-10.36--8.53) |
| Grenada | 16 (5-31) | 13.75 (4.22-26.71) | 0 (0-2) | 0.46 (0.01-2.72) | -11.84 (-12.22--11.46) |
| Guam | 0 (0-0) | 0 (0-0.04) | 0 (0-0) | 0.02 (0-0.16) | 4.65 (3.03-6.3) |
| Guatemala | 11190 (6677-17187) | 67.49 (40.29-103.64) | 3901 (1737-6585) | 27.3 (12.16-46.09) | -4.22 (-4.77--3.66) |
| Guinea | 37889 (23457-60273) | 273.61 (169.59-434.71) | 39907 (24379-65128) | 167.79 (102.56-273.69) | -1.91 (-2.86--0.95) |
| Guinea-Bissau | 7534 (4539-11225) | 344.59 (208.02-511.4) | 6245 (3530-10340) | 181.91 (102.84-301.18) | -2.43 (-3.35--1.51) |
| Guyana | 461 (195-814) | 36.87 (15.64-65.14) | 55 (9-166) | 7.73 (1.22-23.19) | -6.39 (-7.08--5.69) |
| Haiti | 21282 (11371-34028) | 176.53 (94.45-282.13) | 28544 (16005-45918) | 174.36 (97.81-280.29) | -0.63 (-1.21--0.05) |
| Honduras | 7588 (4298-11936) | 88.37 (50.07-139) | 4609 (2339-7930) | 43.1 (21.87-74.12) | -3.1 (-3.58--2.61) |
| Hungary | 13 (1-53) | 0.22 (0.01-0.87) | 1 (0-12) | 0.03 (0-0.28) | -8.09 (-9.37--6.8) |
| Iceland | 0 (0-0) | 0 (0-0.01) | 0 (0-0) | 0 (0-0) | -9.98 (-10.62--9.35) |
| India | 1325778 (1068021-1664608) | 110.67 (89.18-138.89) | 575751 (391046-807755) | 54.87 (37.27-76.98) | -3.53 (-3.83--3.23) |
| Indonesia | 196011 (123059-302954) | 87.26 (54.78-134.87) | 33079 (14879-65691) | 15.6 (7.02-30.99) | -6.08 (-6.53--5.62) |
| Iran (Islamic Republic of) | 911 (389-1793) | 1.23 (0.52-2.42) | 6 (0-35) | 0.01 (0-0.07) | -14.73 (-15.09--14.37) |
| Iraq | 3031 (373-10346) | 9.16 (1.13-31.22) | 91 (1-668) | 0.23 (0-1.68) | -14.95 (-16.34--13.53) |
| Ireland | 0 (0-2) | 0.01 (0-0.06) | 0 (0-0) | 0 (0-0) | -14.34 (-15.22--13.44) |
| Israel | 0 (0-3) | 0.01 (0-0.06) | 0 (0-0) | 0 (0-0) | -9.71 (-9.95--9.47) |
| Italy | 8 (0-61) | 0.03 (0-0.23) | 0 (0-3) | 0 (0-0.02) | -8.4 (-9.73--7.04) |
| Jamaica | 862 (430-1372) | 31.15 (15.54-49.59) | 119 (18-309) | 7.52 (1.12-19.48) | -4.75 (-5.5--4) |
| Japan | 5 (0-42) | 0.01 (0-0.07) | 0 (0-1) | 0 (0-0) | -12.03 (-13.15--10.89) |
| Jordan | 37 (7-112) | 0.57 (0.1-1.75) | 2 (0-7) | 0.01 (0-0.06) | -13.26 (-13.79--12.73) |
| Kazakhstan | 344 (32-1034) | 1.93 (0.18-5.81) | 15 (0-159) | 0.08 (0-0.81) | -12.42 (-13.7--11.11) |
| Kenya | 74032 (53432-97907) | 157.09 (113.4-207.68) | 66264 (48909-88131) | 116.95 (86.32-155.54) | -1.48 (-2.2--0.76) |
| Kiribati | 38 (22-58) | 29.28 (17.1-44.96) | 19 (10-31) | 13.65 (7-21.98) | -3.18 (-3.69--2.67) |
| Kuwait | 0 (0-1) | 0.01 (0-0.06) | 0 (0-0) | 0 (0-0) | -11.97 (-12.92--11) |
| Kyrgyzstan | 237 (108-456) | 3.74 (1.71-7.19) | 495 (245-795) | 6.57 (3.26-10.55) | 1.7 (1.27-2.14) |
| Lao People's Democratic Republic | 13305 (7140-22817) | 160.62 (86.36-275.13) | 5293 (2550-9116) | 62.85 (30.27-108.22) | -4.15 (-4.79--3.51) |
| Latvia | 22 (2-75) | 1.25 (0.1-4.23) | 1 (0-5) | 0.07 (0-0.6) | -12.2 (-13.56--10.82) |
| Lebanon | 32 (3-124) | 0.79 (0.08-3.03) | 1 (0-4) | 0.02 (0-0.1) | -14.41 (-14.8--14.02) |
| Lesotho | 2641 (1379-4680) | 103.24 (53.99-182.91) | 1884 (1054-3013) | 93.16 (52.07-148.86) | -0.82 (-1.25--0.38) |
| Liberia | 15365 (8883-24416) | 286.83 (165.72-455.96) | 12383 (7115-19437) | 158.9 (91.31-249.37) | -2.68 (-3.55--1.79) |
| Libya | 3 (0-27) | 0.05 (0-0.44) | 0 (0-3) | 0.01 (0-0.08) | -6.66 (-8.01--5.3) |
| Lithuania | 14 (1-61) | 0.5 (0.04-2.26) | 0 (0-1) | 0.01 (0-0.13) | -13.85 (-14.8--12.88) |
| Luxembourg | 0 (0-0) | 0 (0-0.01) | 0 (0-0) | 0 (0-0) | -11.26 (-12.06--10.46) |
| Madagascar | 42239 (27165-63593) | 169.47 (109.1-254.85) | 59662 (36241-93757) | 147.37 (89.51-231.53) | -0.74 (-1.61-0.13) |
| Malawi | 55582 (33315-83043) | 232.86 (139.72-347.9) | 41859 (27230-61717) | 153.5 (99.86-226.29) | -2.24 (-3.2--1.27) |
| Malaysia | 569 (62-2284) | 2.38 (0.26-9.54) | 5 (0-44) | 0.02 (0-0.19) | -14.97 (-15.6--14.34) |
| Maldives | 279 (139-490) | 65.72 (32.68-115.11) | 2 (0-11) | 0.72 (0.01-3.78) | -16.74 (-17.26--16.21) |
| Mali | 78891 (48639-128271) | 371.23 (229.58-602.38) | 85821 (51327-133196) | 168.57 (100.86-261.4) | -2.91 (-4.02--1.8) |
| Malta | 0 (0-0) | 0.02 (0-0.14) | 0 (0-0) | 0 (0-0) | -14.32 (-15.24--13.39) |
| Marshall Islands | 9 (4-15) | 11.92 (5.72-21.53) | 4 (2-8) | 7.97 (3.28-14.11) | -1.85 (-2.32--1.37) |
| Mauritania | 9003 (5412-13736) | 217.8 (131.03-332.14) | 5233 (2561-8882) | 80.66 (39.47-136.9) | -3.61 (-4.41--2.8) |
| Mauritius | 35 (5-88) | 3.17 (0.47-7.88) | 0 (0-4) | 0.08 (0-0.64) | -12.01 (-12.66--11.36) |
| Mexico | 11324 (2937-25619) | 9.3 (2.41-21.04) | 2856 (514-8134) | 3.14 (0.57-8.95) | -5.21 (-5.62--4.8) |
| Micronesia (Federated States of) | 36 (18-62) | 23.49 (11.59-41.06) | 6 (3-13) | 7.04 (2.87-14.72) | -5.07 (-5.37--4.76) |
| Monaco | 0 (0-0) | 0 (0-0) | 0 (0-0) | 0 (0-0) | -3.06 (-5.12--0.95) |
| Mongolia | 494 (223-926) | 14.27 (6.45-26.74) | 119 (11-365) | 3.24 (0.3-9.93) | -5.59 (-6.14--5.03) |
| Montenegro | 11 (1-33) | 2.25 (0.2-6.91) | 1 (0-5) | 0.29 (0.01-1.49) | -9.16 (-10.19--8.11) |
| Morocco | 1998 (904-3779) | 5.3 (2.4-10.02) | 97 (13-312) | 0.31 (0.04-1.02) | -9.85 (-10.57--9.12) |
| Mozambique | 106184 (64147-154591) | 365.11 (220.46-532.4) | 114977 (66452-181865) | 217.12 (125.48-343.29) | -2.03 (-2.99--1.05) |
| Myanmar | 117210 (69594-181459) | 219.52 (130.36-339.49) | 46797 (23714-79415) | 90.98 (46.11-154.4) | -3.53 (-3.77--3.29) |
| Namibia | 1848 (992-3033) | 75.29 (40.46-123.6) | 592 (99-1492) | 21.49 (3.58-54.15) | -4.52 (-5.07--3.96) |
| Nauru | 0 (0-1) | 0.54 (0-3.13) | 0 (0-0) | 0.45 (0.01-1.93) | -2.66 (-4.53--0.75) |
| Nepal | 42237 (24591-66780) | 112.76 (65.7-177.71) | 15054 (8297-25933) | 48.88 (26.94-84.21) | -3.75 (-4.23--3.28) |
| Netherlands | 1 (0-5) | 0.01 (0-0.05) | 0 (0-0) | 0 (0-0) | -10.79 (-11.62--9.95) |
| New Zealand | 0 (0-2) | 0.01 (0-0.08) | 0 (0-0) | 0 (0-0.01) | -7.52 (-8.28--6.76) |
| Nicaragua | 7608 (4911-10952) | 111.46 (71.95-160.42) | 2175 (1074-3557) | 35.28 (17.42-57.67) | -4.6 (-4.98--4.22) |
| Niger | 59837 (35994-91712) | 281.62 (169.67-431.39) | 105233 (57824-181864) | 185.94 (102.19-321.23) | -2.07 (-3.28--0.84) |
| Nigeria | 375576 (274125-496769) | 193 (140.91-255.51) | 540148 (351048-762926) | 136.9 (89.01-193.31) | -1.76 (-2.73--0.77) |
| Niue | 0 (0-0) | 4.84 (1.61-9.55) | 0 (0-0) | 1.01 (0.01-4.73) | -9.42 (-10.36--8.47) |
| North Macedonia | 6 (1-14) | 0.35 (0.05-0.86) | 1 (0-5) | 0.12 (0-0.57) | -4.34 (-5.67--3) |
| Northern Mariana Islands | 0 (0-0) | 0.01 (0-0.15) | 0 (0-0) | 0.01 (0-0.11) | -0.37 (-1.28-0.54) |
| Norway | 0 (0-0) | 0 (0-0) | 0 (0-0) | 0 (0-0) | -11.54 (-12.89--10.18) |
| Oman | 10 (0-95) | 0.29 (0-2.75) | 0 (0-0) | 0 (0-0.01) | -16.63 (-17.27--15.97) |
| Pakistan | 189765 (122495-285072) | 94.71 (61.17-142.21) | 171280 (97257-270282) | 57.54 (32.67-90.75) | -2.47 (-3.07--1.86) |
| Palau | 0 (0-0) | 0.01 (0-0.05) | 0 (0-0) | 0 (0-0.01) | -5.88 (-6.45--5.32) |
| Palestine | 271 (110-518) | 6.41 (2.6-12.25) | 41 (11-102) | 0.71 (0.2-1.76) | -7.43 (-8.12--6.74) |
| Panama | 523 (184-1011) | 18.4 (6.49-35.59) | 14 (0-135) | 0.42 (0-3.96) | -12.86 (-13.54--12.17) |
| Papua New Guinea | 2631 (1327-4702) | 37.24 (18.78-66.54) | 5780 (2859-9935) | 35.3 (17.46-60.67) | -0.31 (-1-0.37) |
| Paraguay | 4979 (2724-7606) | 80.17 (43.86-122.48) | 360 (60-898) | 5.8 (0.97-14.47) | -9.9 (-10.56--9.23) |
| Peru | 25541 (11478-45639) | 82.81 (37.22-147.99) | 1981 (128-7964) | 6.12 (0.39-24.62) | -7.56 (-8.26--6.85) |
| Philippines | 115710 (89567-148378) | 119.34 (92.38-153.03) | 56745 (37401-81905) | 51.96 (34.25-75) | -3 (-3.45--2.55) |
| Poland | 960 (236-2003) | 3.65 (0.9-7.61) | 4 (0-33) | 0.02 (0-0.2) | -17.98 (-19.04--16.91) |
| Portugal | 14 (0-71) | 0.26 (0.01-1.29) | 0 (0-1) | 0 (0-0.03) | -14.25 (-15.28--13.21) |
| Puerto Rico | 0 (0-3) | 0.01 (0-0.09) | 0 (0-0) | 0 (0-0.01) | -8.04 (-8.95--7.11) |
| Qatar | 0 (0-0) | 0 (0-0) | 0 (0-0) | 0 (0-0) | -13.9 (-14.6--13.2) |
| Republic of C么te d'Ivoire | 62385 (37567-97176) | 236.27 (142.38-367.19) | 61600 (33513-101906) | 135.99 (74.03-224.9) | -2.35 (-3.21--1.49) |
| Republic of Korea | 90 (5-369) | 0.28 (0.02-1.14) | 0 (0-0) | 0 (0-0) | -21.01 (-22.21--19.79) |
| Republic of Moldova | 1315 (729-2019) | 34.48 (19.09-52.93) | 117 (33-259) | 8.59 (2.43-19.03) | -6.65 (-7.59--5.69) |
| Romania | 112 (21-272) | 0.77 (0.14-1.87) | 1 (0-11) | 0.01 (0-0.13) | -14.81 (-16.22--13.37) |
| Russian Federation | 376 (55-1631) | 0.39 (0.06-1.71) | 42 (0-313) | 0.06 (0-0.48) | -8.92 (-10.64--7.17) |
| Rwanda | 40940 (25961-59943) | 269.76 (171.14-394.57) | 20955 (11961-32347) | 118.34 (67.55-182.67) | -3.28 (-4.06--2.49) |
| Saint Kitts and Nevis | 0 (0-1) | 0.69 (0.05-2.46) | 0 (0-0) | 0.03 (0-0.28) | -10.9 (-11.49--10.31) |
| Saint Lucia | 20 (5-41) | 11.59 (3.06-24.11) | 1 (0-4) | 1.07 (0.04-5.27) | -8.95 (-9.61--8.29) |
| Saint Vincent and the Grenadines | 25 (8-47) | 20.21 (6.16-37.72) | 1 (0-4) | 1.43 (0.08-6.44) | -9.6 (-9.97--9.22) |
| Samoa | 30 (14-57) | 11.4 (5.27-21.79) | 17 (7-30) | 5.56 (2.42-10.14) | -2.29 (-2.8--1.78) |
| San Marino | 0 (0-0) | 0 (0-0.01) | 0 (0-0) | 0 (0-0) | -7.39 (-8.88--5.88) |
| Sao Tome and Principe | 329 (193-523) | 148.38 (87.09-235.9) | 105 (49-195) | 44.27 (20.5-82.37) | -4.78 (-5.55--4.01) |
| Saudi Arabia | 73 (0-638) | 0.3 (0-2.6) | 0 (0-2) | 0 (0-0.01) | -19.53 (-20.2--18.85) |
| Senegal | 35388 (22064-52767) | 214.53 (133.89-319.23) | 32221 (18683-50620) | 139.81 (81.07-219.63) | -1.6 (-2.43--0.76) |
| Serbia | 192 (29-496) | 2.91 (0.44-7.49) | 7 (0-31) | 0.21 (0.01-0.97) | -12.17 (-13.54--10.77) |
| Seychelles | 1 (0-5) | 1.38 (0.04-6.82) | 0 (0-0) | 0.03 (0-0.33) | -12.34 (-12.73--11.95) |
| Sierra Leone | 34383 (19859-55130) | 355.74 (205.32-571.42) | 31512 (19321-50042) | 218.17 (133.83-346.41) | -2.06 (-3.01--1.1) |
| Singapore | 1 (0-5) | 0.02 (0-0.19) | 0 (0-0) | 0 (0-0) | -18.74 (-19.59--17.88) |
| Slovakia | 0 (0-3) | 0.01 (0-0.08) | 0 (0-0) | 0 (0-0) | -11.71 (-12.82--10.57) |
| Slovenia | 0 (0-1) | 0.01 (0-0.1) | 0 (0-0) | 0 (0-0.04) | -5.8 (-7.16--4.42) |
| Solomon Islands | 230 (126-386) | 35.17 (19.19-59.01) | 240 (116-411) | 24.52 (11.83-42.04) | -1.68 (-2.41--0.93) |
| Somalia | 45978 (24016-73045) | 249.45 (130.27-396.13) | 99616 (46396-169033) | 216.79 (101.09-367.72) | -0.49 (-1.61-0.63) |
| South Africa | 14704 (8122-22815) | 29.3 (16.18-45.46) | 3815 (955-8847) | 8.07 (2.02-18.72) | -5.47 (-5.85--5.1) |
| South Sudan | 23550 (12370-38328) | 192.92 (101.51-313.77) | 35491 (17763-67332) | 194.47 (97.35-368.77) | -0.06 (-1.03-0.91) |
| Spain | 25 (0-172) | 0.13 (0-0.9) | 0 (0-4) | 0 (0-0.03) | -12.49 (-13.54--11.42) |
| Sri Lanka | 9027 (5999-13076) | 52.26 (34.72-75.7) | 814 (125-1889) | 5.59 (0.86-12.98) | -7.76 (-8.4--7.12) |
| Sudan | 7481 (3875-12749) | 17.97 (9.3-30.52) | 3619 (1586-6894) | 6.57 (2.88-12.51) | -4.12 (-4.91--3.33) |
| Suriname | 119 (19-310) | 27.11 (4.41-70.62) | 13 (0-67) | 3.05 (0.08-15.76) | -8.63 (-9.13--8.12) |
| Sweden | 0 (0-1) | 0 (0-0.02) | 0 (0-0) | 0 (0-0) | -10.57 (-11.46--9.67) |
| Switzerland | 0 (0-0) | 0 (0-0) | 0 (0-0) | 0 (0-0) | -6.81 (-7.82--5.79) |
| Syrian Arab Republic | 499 (164-1112) | 2.27 (0.74-5.05) | 2 (0-7) | 0.02 (0-0.07) | -15.82 (-16.26--15.39) |
| Taiwan (Province of China) | 8 (0-29) | 0.05 (0-0.18) | 1 (0-7) | 0.01 (0-0.1) | -4.31 (-5.49--3.12) |
| Tajikistan | 1430 (612-2842) | 14.31 (6.12-28.43) | 1214 (500-2300) | 8.92 (3.68-16.91) | -2.37 (-2.98--1.75) |
| Thailand | 20392 (9336-37079) | 40.86 (18.7-74.31) | 203 (4-1300) | 0.78 (0.01-5.02) | -13.39 (-14.17--12.61) |
| Timor-Leste | 2640 (1537-4159) | 158.71 (92.48-249.97) | 1295 (649-2155) | 66.13 (33.13-110.03) | -4.43 (-5.18--3.67) |
| Togo | 17369 (10726-27126) | 226.7 (140.15-353.38) | 15226 (8826-24385) | 130.91 (75.91-209.59) | -2.31 (-3.12--1.49) |
| Tokelau | 0 (0-0) | 0.34 (0.01-1.33) | 0 (0-0) | 0.1 (0-0.51) | -9.13 (-10.22--8.02) |
| Tonga | 22 (9-41) | 13.93 (5.75-25.93) | 8 (3-16) | 5.39 (1.85-11.44) | -3.19 (-3.74--2.64) |
| Trinidad and Tobago | 4 (0-30) | 0.39 (0.01-2.59) | 0 (0-1) | 0.01 (0-0.07) | -15.84 (-16.71--14.96) |
| Tunisia | 180 (59-394) | 1.71 (0.56-3.74) | 1 (0-4) | 0.01 (0-0.05) | -17.55 (-17.87--17.23) |
| Turkey | 5784 (759-16721) | 8.16 (1.07-23.6) | 26 (0-229) | 0.05 (0-0.47) | -16.77 (-17.39--16.16) |
| Turkmenistan | 8 (1-35) | 0.14 (0.02-0.58) | 0 (0-3) | 0.01 (0-0.06) | -11.53 (-12.9--10.15) |
| Tuvalu | 2 (1-5) | 13.41 (5.44-28.43) | 0 (0-1) | 1.91 (0.29-5) | -7.4 (-7.74--7.06) |
| Uganda | 87932 (57178-132224) | 199.83 (129.94-300.25) | 107689 (68740-171394) | 142.46 (90.94-226.71) | -1.55 (-2.63--0.46) |
| Ukraine | 247 (22-916) | 0.77 (0.07-2.84) | 57 (5-239) | 0.43 (0.04-1.8) | -3.97 (-5.38--2.53) |
| United Arab Emirates | 0 (0-0) | 0 (0-0) | 0 (0-0) | 0 (0-0) | -9.24 (-10.15--8.33) |
| United Kingdom | 0 (0-4) | 0 (0-0.01) | 0 (0-0) | 0 (0-0) | -11.15 (-11.96--10.33) |
| United Republic of Tanzania | 127457 (78994-194664) | 221.82 (137.59-338.79) | 166983 (96040-268033) | 184.97 (106.42-296.88) | -0.69 (-1.61-0.24) |
| United States of America | 3 (0-28) | 0 (0-0.01) | 1 (0-5) | 0 (0-0) | -5.57 (-6.19--4.95) |
| United States Virgin Islands | 0 (0-1) | 0.05 (0-0.49) | 0 (0-0) | 0 (0-0.01) | -10 (-11.28--8.69) |
| Uruguay | 85 (11-255) | 3.17 (0.43-9.47) | 1 (0-11) | 0.08 (0-0.66) | -12.22 (-12.86--11.58) |
| Uzbekistan | 1777 (845-3364) | 5.21 (2.48-9.87) | 1219 (223-3615) | 3.18 (0.58-9.43) | -1.93 (-2.55--1.31) |
| Vanuatu | 68 (35-108) | 23.22 (11.92-37.08) | 72 (40-125) | 17.22 (9.42-29.71) | -1.69 (-2.41--0.96) |
| Venezuela (Bolivarian Republic of) | 251 (9-1299) | 0.95 (0.03-4.92) | 68 (2-434) | 0.31 (0.01-2) | -4.81 (-5.27--4.34) |
| Viet Nam | 96772 (58909-159678) | 104.22 (63.45-171.96) | 12075 (5147-22037) | 16.17 (6.89-29.52) | -7.1 (-7.37--6.83) |
| Yemen | 6068 (3465-9972) | 19.36 (11.05-31.86) | 3919 (1930-6914) | 8.42 (4.14-14.84) | -3.5 (-4.39--2.6) |
| Zambia | 30710 (18074-45028) | 168.28 (99-246.45) | 37224 (20648-59694) | 128.54 (71.31-206.12) | -1.26 (-2.26--0.26) |
| Zimbabwe | 15976 (9343-24391) | 86.04 (50.34-131.38) | 21273 (12613-33214) | 95.06 (56.36-148.39) | 1.06 (0.32-1.81) |
| **Ambient particulate matter pollution** |  |  |  |  |  |
| **region_eapc** |  |  |  |  |  |
| Advanced Health System | 51487 (35246-69334) | 5.66 (3.87-7.62) | 14241 (10712-19122) | 2.02 (1.52-2.72) | -3.89 (-4.64--3.12) |
| Africa | 346503 (212525-509740) | 27.11 (16.63-39.9) | 547783 (301853-879191) | 26.56 (14.63-42.62) | 0.32 (-0.51-1.17) |
| African Region | 334909 (203387-492771) | 31.22 (18.96-45.93) | 532931 (290099-860069) | 29.71 (16.17-47.94) | 0.27 (-0.62-1.17) |
| America | 137977 (78068-203099) | 18.01 (10.19-26.51) | 67477 (41089-92367) | 9.9 (6.03-13.55) | -2.55 (-2.89--2.2) |
| Andean Latin America | 36033 (16518-63484) | 64.1 (29.39-112.88) | 15842 (7533-26493) | 26.61 (12.64-44.54) | -3.07 (-3.4--2.74) |
| Asia | 518432 (330461-797240) | 13.61 (8.67-20.93) | 550311 (310636-857586) | 18.02 (10.17-28.07) | -0.06 (-0.36-0.24) |
| Australasia | 165 (19-374) | 1.08 (0.12-2.45) | 86 (19-178) | 0.5 (0.11-1.03) | -2.8 (-3.63--1.96) |
| Basic Health System | 294999 (168267-433967) | 11.49 (6.56-16.91) | 198618 (120754-271323) | 10.54 (6.41-14.4) | -1.32 (-1.7--0.95) |
| Caribbean | 7148 (2794-14502) | 16.57 (6.47-33.61) | 10570 (5137-16772) | 27.71 (13.47-43.97) | 1.33 (1.11-1.56) |
| Central Africa | 22746 (12896-37322) | 15.22 (8.63-24.96) | 38515 (21991-65480) | 14.3 (8.17-24.31) | 0.04 (-0.92-1.01) |
| Central Asia | 4977 (2190-8612) | 5.28 (2.32-9.13) | 8869 (5469-12990) | 9.01 (5.56-13.21) | 2.66 (2.17-3.17) |
| Central Europe | 2799 (1164-4245) | 3.38 (1.41-5.13) | 351 (226-479) | 0.7 (0.45-0.95) | -6.22 (-7.26--5.18) |
| Central Latin America | 54343 (30440-75418) | 22.69 (12.71-31.49) | 25400 (15400-35790) | 13.52 (8.2-19.05) | -2.65 (-3.01--2.3) |
| Central Sub-Saharan Africa | 9754 (4740-18353) | 7.95 (3.87-14.97) | 19538 (9274-33513) | 9.15 (4.34-15.69) | 0.91 (-0.07-1.9) |
| Commonwealth High Income | 1297 (983-1710) | 1.65 (1.25-2.17) | 544 (356-794) | 0.74 (0.48-1.08) | -3.11 (-3.81--2.41) |
| Commonwealth Low Income | 88274 (51988-144032) | 20.59 (12.12-33.58) | 75154 (43344-121427) | 16.02 (9.24-25.88) | -1.05 (-1.77--0.32) |
| Commonwealth Middle Income | 449608 (280469-705688) | 25.28 (15.76-39.68) | 692662 (375068-1117658) | 34.93 (18.91-56.36) | 0.83 (0.39-1.26) |
| East Asia | 20070 (9382-36824) | 1.75 (0.82-3.21) | 8938 (5106-12972) | 1.61 (0.92-2.34) | -0.39 (-1.23-0.46) |
| East Asia & Pacific - WB | 138158 (71620-232306) | 7.49 (3.88-12.6) | 112701 (50311-187154) | 9.59 (4.28-15.92) | -0.62 (-1.17--0.08) |
| Eastern Africa | 56699 (37503-86225) | 14.66 (9.69-22.3) | 78182 (48501-123680) | 13.5 (8.38-21.36) | 0.03 (-0.87-0.95) |
| Eastern Europe | 10345 (7609-12868) | 7.19 (5.29-8.94) | 3244 (2448-4102) | 3.75 (2.83-4.74) | -2.68 (-3.65--1.69) |
| Eastern Mediterranean Region | 93885 (58957-139267) | 14.78 (9.28-21.93) | 134992 (82074-224112) | 16.35 (9.94-27.15) | -0.24 (-0.74-0.25) |
| Eastern Sub-Saharan Africa | 67993 (45269-103058) | 15.89 (10.58-24.08) | 92620 (56538-148092) | 14.13 (8.63-22.59) | -0.16 (-1.09-0.77) |
| Europe | 37757 (22250-53513) | 7.11 (4.19-10.08) | 10899 (7733-15075) | 2.84 (2.01-3.93) | -3.32 (-4.14--2.51) |
| Europe & Central Asia - WB | 40627 (23415-57607) | 6.74 (3.88-9.56) | 18061 (12463-23768) | 3.86 (2.67-5.09) | -2.15 (-2.88--1.42) |
| European Region | 40756 (23507-57767) | 6.68 (3.85-9.47) | 18143 (12522-23896) | 3.8 (2.62-5.01) | -2.17 (-2.88--1.45) |
| High-income Asia Pacific | 2316 (1289-3737) | 2.43 (1.35-3.92) | 362 (227-553) | 0.63 (0.4-0.96) | -5.54 (-6.64--4.42) |
| High-income North America | 4351 (3573-5136) | 1.98 (1.62-2.33) | 1590 (1180-2055) | 0.81 (0.6-1.05) | -3.68 (-4.34--3.01) |
| Latin America & Caribbean - WB | 133728 (73855-199089) | 24.35 (13.45-36.25) | 65914 (39693-90784) | 13.53 (8.15-18.64) | -2.57 (-2.87--2.28) |
| Limited Health System | 615020 (401553-931158) | 23.62 (15.41-35.78) | 853762 (467252-1367304) | 28.62 (15.66-45.83) | 0.41 (-0.11-0.93) |
| Middle East & North Africa - WB | 46978 (31757-65373) | 11.86 (8.02-16.51) | 30300 (20347-41845) | 7.18 (4.82-9.92) | -2.14 (-2.51--1.76) |
| Minimal Health System | 79268 (49724-115802) | 25.67 (16.11-37.52) | 109880 (71353-170549) | 17.91 (11.63-27.79) | -0.92 (-1.94-0.11) |
| North Africa and Middle East | 66477 (40911-93231) | 12.69 (7.81-17.8) | 39042 (27056-52966) | 6.81 (4.72-9.23) | -2.47 (-2.83--2.1) |
| North America | 4351 (3574-5136) | 1.98 (1.62-2.33) | 1590 (1180-2055) | 0.81 (0.6-1.05) | -3.68 (-4.34--3.01) |
| Northern Africa | 12043 (6344-20336) | 6.49 (3.42-10.96) | 10656 (6844-15551) | 4.91 (3.15-7.17) | -1.26 (-1.72--0.8) |
| Oceania | 268 (62-761) | 2.49 (0.58-7.07) | 690 (173-1770) | 3.36 (0.84-8.62) | 0.62 (-0.01-1.25) |
| Region of the Americas | 137977 (78068-203099) | 18.01 (10.19-26.51) | 67477 (41089-92367) | 9.9 (6.03-13.55) | -2.55 (-2.89--2.2) |
| South-East Asia Region | 364812 (221733-585895) | 20.13 (12.24-32.31) | 370641 (206693-568230) | 24.14 (13.46-37.02) | -0.36 (-0.73-0.01) |
| South Asia | 337340 (187995-560851) | 20.58 (11.47-34.18) | 404296 (225522-633230) | 26.73 (14.91-41.86) | 0.01 (-0.42-0.43) |
| South Asia - WB | 339523 (189668-564003) | 20.23 (11.31-33.58) | 407649 (227435-637744) | 25.71 (14.34-40.22) | -0.03 (-0.45-0.4) |
| Southeast Asia | 116807 (53892-211190) | 19.74 (9.11-35.68) | 103690 (45113-177121) | 19.21 (8.36-32.8) | -1.16 (-1.38--0.94) |
| Southern Africa | 30737 (18408-46189) | 17.81 (10.67-26.76) | 45943 (25711-69367) | 18.73 (10.48-28.29) | 0.24 (-0.45-0.93) |
| Southern Latin America | 6317 (2757-10435) | 12.45 (5.43-20.57) | 1890 (901-3038) | 5.06 (2.41-8.14) | -4.47 (-4.87--4.07) |
| Southern Sub-Saharan Africa | 16182 (8124-26732) | 20.87 (10.47-34.49) | 20147 (11867-28973) | 25.78 (15.19-37.08) | 0.48 (0.14-0.83) |
| Sub-Saharan Africa - WB | 337399 (204834-495142) | 30.79 (18.69-45.17) | 540277 (297344-870395) | 29.19 (16.06-47.02) | 0.26 (-0.65-1.19) |
| Tropical Latin America | 30130 (12691-50721) | 18.71 (7.88-31.49) | 12569 (7217-17964) | 7.58 (4.36-10.84) | -3.37 (-3.9--2.83) |
| Western Africa | 224278 (129046-344747) | 58.45 (33.65-89.89) | 374488 (187960-643552) | 49.76 (24.98-85.52) | 0.07 (-0.93-1.08) |
| Western Europe | 5201 (3987-6655) | 2.33 (1.79-2.99) | 1539 (1027-2178) | 0.78 (0.52-1.1) | -3.56 (-4.43--2.69) |
| Western Pacific Region | 68302 (40693-106772) | 4.64 (2.76-7.25) | 52140 (26772-80601) | 6.05 (3.11-9.35) | -0.32 (-0.96-0.32) |
| Western Sub-Saharan Africa | 242329 (140866-371055) | 56.87 (33.05-87.14) | 405888 (206528-688480) | 47.74 (24.3-80.97) | 0 (-1-1.01) |
| **country_eapc** |  |  |  |  |  |
| location_name | Num_1990 | ASR_1990 | Num_2021 | ASR_2021 | EAPC_CI |
| Afghanistan | 903 (342-1975) | 4.31 (1.63-9.43) | 2217 (1013-4261) | 3.82 (1.74-7.34) | -0.44 (-1.52-0.65) |
| Albania | 3 (1-7) | 0.08 (0.02-0.18) | 4 (1-8) | 0.28 (0.07-0.61) | 3.53 (2.6-4.47) |
| Algeria | 1354 (492-2874) | 3.67 (1.33-7.78) | 975 (292-2695) | 2.23 (0.67-6.17) | -2.12 (-2.38--1.86) |
| American Samoa | 1 (0-3) | 1.45 (0.07-4.06) | 0 (0-0) | 0.49 (0.02-1.53) | -6.05 (-6.71--5.39) |
| Andorra | 0 (0-1) | 1.25 (0.33-2.89) | 0 (0-0) | 0.16 (0-0.51) | -6.02 (-7.32--4.7) |
| Angola | 3876 (1578-7968) | 16.24 (6.63-33.35) | 10840 (3648-21415) | 18.72 (6.3-36.96) | 1.07 (-0.02-2.17) |
| Antigua and Barbuda | 4 (1-7) | 6.47 (1.9-12.31) | 4 (1-7) | 7.63 (2.28-14.28) | -0.42 (-0.9-0.06) |
| Argentina | 5431 (2387-9420) | 16.27 (7.15-28.23) | 1411 (477-2457) | 5.46 (1.84-9.51) | -5.09 (-5.46--4.73) |
| Armenia | 1183 (441-2281) | 32.57 (12.13-62.8) | 576 (313-924) | 34.63 (18.77-55.51) | 0.35 (-0.31-1.02) |
| Australia | 151 (5-357) | 1.22 (0.04-2.89) | 70 (5-154) | 0.49 (0.03-1.08) | -3.59 (-4.51--2.66) |
| Austria | 52 (21-86) | 1.16 (0.47-1.93) | 15 (2-33) | 0.37 (0.05-0.81) | -3.67 (-4.65--2.67) |
| Azerbaijan | 688 (145-1645) | 7.75 (1.63-18.52) | 641 (244-1390) | 9.95 (3.79-21.57) | 1.61 (0.88-2.34) |
| Bahamas | 50 (22-88) | 18.72 (8.34-33.07) | 29 (12-53) | 15.2 (6.17-27.49) | -1.63 (-2.21--1.04) |
| Bahrain | 63 (36-100) | 9.85 (5.64-15.67) | 23 (13-39) | 2.68 (1.53-4.59) | -4.86 (-5.27--4.45) |
| Bangladesh | 55115 (24831-105606) | 27.23 (12.26-52.07) | 26723 (10965-53490) | 19.91 (8.17-39.85) | -2.11 (-2.68--1.54) |
| Barbados | 41 (20-67) | 20.51 (9.81-33.47) | 34 (15-64) | 27.15 (11.7-50.4) | 0.42 (-0.37-1.22) |
| Belarus | 717 (330-1300) | 10.45 (4.83-18.96) | 135 (35-269) | 3.36 (0.86-6.73) | -3.56 (-4.5--2.6) |
| Belgium | 144 (54-250) | 2.38 (0.89-4.13) | 46 (8-100) | 0.84 (0.14-1.82) | -3.99 (-4.8--3.18) |
| Belize | 52 (17-103) | 17.33 (5.7-34.12) | 74 (33-133) | 20.16 (8.83-36.17) | -0.31 (-0.78-0.17) |
| Benin | 5145 (2469-8864) | 45.33 (21.77-78.03) | 7300 (3384-14474) | 29.15 (13.51-57.77) | -0.95 (-1.99-0.11) |
| Bermuda | 0 (0-1) | 1.09 (0.07-2.49) | 0 (0-0) | 0.33 (0-0.88) | -4.7 (-5.55--3.84) |
| Bhutan | 120 (50-253) | 11.29 (4.73-23.79) | 89 (32-164) | 14.88 (5.36-27.45) | 0.58 (0.13-1.03) |
| Bolivia (Plurinational State of) | 5409 (2270-10542) | 49.57 (20.78-96.55) | 2824 (1117-5115) | 24.1 (9.53-43.64) | -3.62 (-4.05--3.18) |
| Bosnia and Herzegovina | 28 (7-78) | 0.86 (0.22-2.39) | 22 (5-57) | 1.66 (0.38-4.26) | 2.2 (0.99-3.42) |
| Botswana | 443 (132-1004) | 19.95 (5.94-45.21) | 831 (254-1579) | 35.73 (10.93-67.87) | 1.33 (0.75-1.91) |
| Brazil | 29073 (12410-48578) | 18.78 (8.01-31.37) | 12191 (7143-17478) | 7.64 (4.48-10.96) | -3.32 (-3.87--2.77) |
| Brunei Darussalam | 11 (2-24) | 3.29 (0.47-7.22) | 10 (2-23) | 3.26 (0.55-7.85) | -1.06 (-1.53--0.58) |
| Bulgaria | 21 (8-37) | 0.43 (0.16-0.77) | 22 (7-39) | 0.78 (0.24-1.39) | 1.64 (-0.13-3.43) |
| Burkina Faso | 8730 (4227-15829) | 39.51 (19.17-71.6) | 14439 (7112-26989) | 31.76 (15.64-59.36) | -0.12 (-1.2-0.96) |
| Burundi | 2155 (908-4442) | 17.35 (7.32-35.77) | 2351 (1053-4650) | 10.5 (4.7-20.76) | -1.82 (-2.74--0.89) |
| Cabo Verde | 94 (44-169) | 15.62 (7.25-28.05) | 140 (64-263) | 34.32 (15.59-64.41) | 1.8 (1.26-2.34) |
| Cambodia | 2360 (818-5266) | 11.53 (4-25.72) | 1620 (608-3426) | 9.46 (3.55-20.01) | -2.08 (-2.58--1.58) |
| Cameroon | 9149 (3672-17299) | 40.14 (16.11-75.81) | 13428 (5814-27573) | 27.02 (11.7-55.48) | -0.99 (-1.91--0.06) |
| Canada | 157 (27-328) | 0.8 (0.14-1.68) | 82 (0-189) | 0.46 (0-1.06) | -2.3 (-3.1--1.5) |
| Central African Republic | 538 (244-1006) | 8.95 (4.07-16.73) | 793 (353-1497) | 8.76 (3.9-16.54) | -0.09 (-1.04-0.86) |
| Chad | 5533 (2679-10000) | 36.31 (17.6-65.67) | 14010 (6483-27377) | 33.96 (15.72-66.35) | 0.28 (-0.95-1.51) |
| Chile | 620 (172-1342) | 4.22 (1.17-9.14) | 418 (167-695) | 4.28 (1.7-7.11) | -1.05 (-1.61--0.49) |
| China | 19615 (9100-35767) | 1.77 (0.82-3.23) | 8702 (4897-12624) | 1.63 (0.92-2.37) | -0.4 (-1.24-0.46) |
| Colombia | 4804 (1535-9131) | 10.87 (3.47-20.65) | 3323 (1161-6421) | 10.24 (3.57-19.81) | -0.76 (-1.43--0.1) |
| Comoros | 113 (46-220) | 12.02 (4.89-23.45) | 69 (30-137) | 8.51 (3.69-16.88) | -1.73 (-2.36--1.1) |
| Congo | 451 (138-1092) | 10.32 (3.15-25.02) | 1159 (346-2484) | 18.97 (5.67-40.66) | 2.21 (1.48-2.94) |
| Cook Islands | 0 (0-1) | 2.1 (0.2-5.53) | 0 (0-0) | 0.92 (0.02-2.73) | -5.46 (-6.03--4.87) |
| Costa Rica | 183 (53-360) | 4.67 (1.35-9.2) | 70 (17-132) | 2.67 (0.66-5.03) | -3.6 (-4.09--3.1) |
| Croatia | 31 (12-51) | 1.15 (0.44-1.91) | 32 (9-65) | 1.91 (0.56-3.81) | -0.03 (-1.71-1.69) |
| Cuba | 630 (211-1134) | 7.34 (2.46-13.21) | 168 (53-302) | 3.47 (1.09-6.25) | -2.86 (-3.65--2.05) |
| Cyprus | 24 (8-49) | 3.56 (1.24-7.48) | 6 (2-12) | 0.8 (0.26-1.64) | -5.96 (-6.61--5.3) |
| Czechia | 53 (26-86) | 0.85 (0.42-1.39) | 30 (8-57) | 0.58 (0.15-1.11) | -0.81 (-2.18-0.58) |
| Democratic People's Republic of Korea | 428 (140-948) | 1.66 (0.54-3.67) | 92 (38-192) | 0.65 (0.27-1.35) | -3.68 (-4.16--3.21) |
| Democratic Republic of the Congo | 4253 (1790-8742) | 4.96 (2.09-10.19) | 5510 (2250-11986) | 4.03 (1.65-8.77) | -0.17 (-1.15-0.81) |
| Denmark | 14 (5-25) | 0.44 (0.15-0.81) | 6 (0-13) | 0.18 (0.01-0.43) | -3.29 (-4.05--2.52) |
| Djibouti | 392 (167-753) | 52.58 (22.49-101) | 780 (337-1426) | 53.89 (23.24-98.46) | 0.08 (-0.59-0.75) |
| Dominica | 15 (3-40) | 16.83 (3.33-44.21) | 15 (6-31) | 49.19 (17.96-99.65) | 2.3 (1.7-2.9) |
| Dominican Republic | 3361 (847-8284) | 31.25 (7.87-77.04) | 6548 (2539-11338) | 63.86 (24.77-110.58) | 1.97 (1.59-2.35) |
| Ecuador | 4877 (2248-8243) | 33.73 (15.55-56.99) | 1332 (472-2470) | 8.63 (3.06-16.01) | -6.32 (-7.04--5.6) |
| Egypt | 5274 (1940-11436) | 5.86 (2.15-12.7) | 4295 (2127-7609) | 3.45 (1.71-6.12) | -1.79 (-2.44--1.13) |
| El Salvador | 1247 (472-2406) | 14.93 (5.65-28.8) | 371 (122-724) | 6.62 (2.17-12.92) | -3.46 (-3.88--3.04) |
| Equatorial Guinea | 101 (39-227) | 10.35 (4.02-23.14) | 622 (271-1100) | 34.59 (15.09-61.19) | 3.8 (2.56-5.05) |
| Eritrea | 1481 (698-2848) | 20.93 (9.86-40.24) | 1799 (830-3558) | 19.23 (8.87-38.02) | -0.89 (-1.69--0.08) |
| Estonia | 48 (11-93) | 4.59 (1.06-8.93) | 6 (0-14) | 0.96 (0.05-2.27) | -6.52 (-7.47--5.56) |
| Eswatini | 227 (82-508) | 14.69 (5.27-32.83) | 304 (80-683) | 21.9 (5.75-49.18) | 0.52 (-0.12-1.16) |
| Ethiopia | 22050 (14035-33917) | 19.05 (12.13-29.3) | 24847 (14137-41626) | 14.9 (8.48-24.97) | -0.63 (-1.52-0.27) |
| Fiji | 35 (7-102) | 3.87 (0.77-11.24) | 50 (10-115) | 5.76 (1.13-13.19) | 0.42 (-0.15-0.98) |
| Finland | 16 (1-38) | 0.5 (0.03-1.22) | 6 (0-15) | 0.24 (0-0.62) | -3.13 (-4.01--2.25) |
| France | 1083 (302-2024) | 2.93 (0.82-5.49) | 336 (32-788) | 1.01 (0.1-2.37) | -3.24 (-3.96--2.5) |
| Gabon | 536 (128-1139) | 31.43 (7.52-66.75) | 614 (278-1126) | 29.72 (13.43-54.52) | -0.01 (-0.61-0.59) |
| Gambia | 1146 (512-2154) | 53.17 (23.8-99.93) | 1091 (504-2170) | 29.45 (13.62-58.6) | -2.32 (-3.21--1.42) |
| Georgia | 157 (33-364) | 3.79 (0.79-8.77) | 193 (56-379) | 8.95 (2.58-17.6) | 5.79 (4.51-7.08) |
| Germany | 913 (400-1496) | 2.15 (0.94-3.53) | 247 (40-518) | 0.65 (0.11-1.37) | -4.03 (-5.1--2.96) |
| Ghana | 17786 (8854-32360) | 61.7 (30.75-112.18) | 28966 (10600-60580) | 62.39 (22.84-130.47) | 0.54 (-0.21-1.3) |
| Greece | 72 (23-132) | 1.42 (0.46-2.62) | 25 (8-48) | 0.63 (0.2-1.19) | -2.42 (-3.53--1.3) |
| Greenland | 0 (0-1) | 0.36 (0.01-1.16) | 0 (0-0) | 0.19 (0-0.59) | -2.51 (-3.37--1.64) |
| Grenada | 14 (4-29) | 11.94 (3.42-25.28) | 13 (5-23) | 20.26 (7.28-35.82) | 0.78 (0.38-1.19) |
| Guam | 1 (0-2) | 0.36 (0.04-0.85) | 3 (0-7) | 2.46 (0.31-5.87) | 6.58 (5.15-8.04) |
| Guatemala | 4226 (1633-8526) | 25.49 (9.85-51.41) | 2234 (626-4699) | 15.64 (4.38-32.86) | -3.01 (-3.59--2.42) |
| Guinea | 6231 (2997-11288) | 45 (21.65-81.37) | 6152 (2881-11864) | 25.87 (12.11-49.85) | -1.73 (-2.7--0.75) |
| Guinea-Bissau | 1311 (613-2444) | 59.96 (28.09-111.61) | 995 (487-1810) | 28.99 (14.2-52.77) | -2.61 (-3.52--1.69) |
| Guyana | 251 (85-569) | 20.1 (6.82-45.5) | 310 (125-578) | 43.22 (17.36-80.52) | 1.24 (0.69-1.8) |
| Haiti | 1501 (388-3762) | 12.45 (3.22-31.21) | 2203 (767-4865) | 13.46 (4.69-29.72) | -0.15 (-0.74-0.45) |
| Honduras | 1488 (601-2984) | 17.33 (7-34.75) | 1235 (413-2734) | 11.55 (3.86-25.57) | -1.98 (-2.48--1.47) |
| Hungary | 63 (18-115) | 1.05 (0.29-1.9) | 25 (6-49) | 0.58 (0.14-1.15) | -2.14 (-3.45--0.8) |
| Iceland | 1 (0-3) | 0.63 (0.05-1.47) | 0 (0-1) | 0.16 (0-0.41) | -4.61 (-5.09--4.13) |
| India | 232191 (128007-383651) | 19.38 (10.68-32.04) | 277805 (161405-425825) | 26.48 (15.38-40.58) | 0.13 (-0.27-0.54) |
| Indonesia | 45352 (18025-91967) | 20.19 (8.02-40.94) | 39268 (14648-78836) | 18.52 (6.91-37.19) | -1.53 (-1.76--1.29) |
| Iran (Islamic Republic of) | 4476 (3064-6153) | 6.03 (4.13-8.29) | 1160 (626-1687) | 2.33 (1.26-3.38) | -2.03 (-2.6--1.44) |
| Iraq | 18890 (9171-30002) | 57.06 (27.7-90.56) | 13654 (6938-23102) | 34.23 (17.4-57.9) | -2.18 (-2.74--1.61) |
| Ireland | 17 (4-34) | 0.66 (0.16-1.31) | 8 (0-20) | 0.28 (0-0.75) | -2.96 (-3.51--2.4) |
| Israel | 119 (54-197) | 2.34 (1.07-3.89) | 75 (25-137) | 0.84 (0.28-1.54) | -3.31 (-3.56--3.07) |
| Italy | 851 (673-1034) | 3.15 (2.49-3.83) | 294 (203-406) | 1.51 (1.04-2.08) | -2.15 (-3.32--0.96) |
| Jamaica | 290 (74-679) | 10.46 (2.68-24.55) | 341 (120-669) | 21.46 (7.54-42.18) | 1.53 (1.04-2.02) |
| Japan | 515 (411-624) | 0.86 (0.68-1.04) | 126 (99-156) | 0.3 (0.24-0.38) | -4.93 (-6.12--3.72) |
| Jordan | 1687 (827-2735) | 26.42 (12.96-42.82) | 1736 (846-2985) | 16.72 (8.15-28.78) | -2.39 (-2.93--1.84) |
| Kazakhstan | 869 (232-1788) | 4.88 (1.3-10.05) | 730 (281-1327) | 3.7 (1.42-6.73) | 0.08 (-0.38-0.54) |
| Kenya | 5107 (2872-8623) | 10.84 (6.1-18.3) | 7051 (3784-12785) | 12.44 (6.68-22.56) | 0.52 (-0.25-1.3) |
| Kiribati | 1 (0-4) | 1 (0.24-2.91) | 1 (0-3) | 0.91 (0.3-2.26) | -1.14 (-1.64--0.64) |
| Kuwait | 156 (85-234) | 9.65 (5.28-14.45) | 113 (61-175) | 4.72 (2.54-7.28) | -2.72 (-3.47--1.97) |
| Kyrgyzstan | 85 (27-200) | 1.34 (0.42-3.16) | 284 (116-522) | 3.77 (1.54-6.93) | 3.94 (3.04-4.84) |
| Lao People's Democratic Republic | 1137 (347-2510) | 13.73 (4.19-30.31) | 1554 (420-3621) | 18.44 (4.99-42.99) | -0.16 (-0.77-0.46) |
| Latvia | 86 (32-151) | 4.86 (1.81-8.53) | 12 (3-24) | 1.5 (0.33-3.01) | -4.2 (-5.21--3.18) |
| Lebanon | 208 (65-453) | 5.07 (1.6-11.07) | 116 (47-232) | 3.05 (1.24-6.09) | -2.64 (-2.94--2.34) |
| Lesotho | 269 (128-522) | 10.52 (5.02-20.38) | 283 (126-542) | 13.99 (6.24-26.78) | 0.62 (0.14-1.12) |
| Liberia | 1851 (908-3501) | 34.55 (16.94-65.37) | 1361 (696-2456) | 17.47 (8.93-31.51) | -1.64 (-2.58--0.69) |
| Libya | 290 (134-522) | 4.63 (2.14-8.34) | 130 (42-404) | 3.36 (1.1-10.47) | -2.62 (-3.02--2.23) |
| Lithuania | 108 (49-178) | 3.98 (1.82-6.53) | 15 (2-34) | 1.32 (0.2-2.98) | -4.57 (-5.51--3.61) |
| Luxembourg | 2 (1-4) | 0.92 (0.26-1.64) | 1 (0-2) | 0.21 (0.01-0.46) | -5.04 (-5.8--4.27) |
| Madagascar | 1157 (590-1984) | 4.64 (2.37-7.96) | 2281 (1259-4058) | 5.63 (3.11-10.03) | 1.41 (0.46-2.36) |
| Malawi | 3024 (1624-5283) | 12.67 (6.82-22.15) | 2469 (1270-4405) | 9.05 (4.66-16.15) | -1.44 (-2.43--0.45) |
| Malaysia | 5757 (2603-9844) | 24.06 (10.87-41.17) | 1572 (568-2873) | 6.83 (2.47-12.48) | -5.2 (-5.63--4.76) |
| Maldives | 64 (17-154) | 15.01 (3.91-36.28) | 19 (5-40) | 6.42 (1.79-13.67) | -5.12 (-5.6--4.63) |
| Mali | 13178 (6194-23573) | 62.01 (29.21-110.71) | 17106 (8186-32252) | 33.6 (16.09-63.36) | -1.69 (-2.83--0.55) |
| Malta | 2 (1-4) | 0.76 (0.29-1.33) | 1 (0-2) | 0.38 (0.05-0.79) | -2.54 (-3.43--1.63) |
| Marshall Islands | 1 (0-3) | 1.56 (0.38-4.16) | 1 (0-2) | 1.6 (0.46-4.06) | -1.16 (-1.72--0.6) |
| Mauritania | 3331 (1543-6009) | 80.59 (37.33-145.2) | 3930 (1831-6969) | 60.57 (28.23-107.42) | -1.23 (-2.04--0.41) |
| Mauritius | 62 (14-123) | 5.55 (1.25-11.06) | 34 (9-68) | 5.6 (1.47-11.16) | -1.23 (-1.96--0.49) |
| Mexico | 31504 (16517-43024) | 25.87 (13.56-35.33) | 13826 (8193-18809) | 15.22 (9.02-20.7) | -3.11 (-3.47--2.75) |
| Micronesia (Federated States of) | 3 (1-7) | 1.79 (0.4-4.73) | 1 (0-3) | 1.46 (0.38-3.72) | -1.81 (-2.14--1.49) |
| Monaco | 0 (0-0) | 0.8 (0.07-2.02) | 0 (0-0) | 0.51 (0.03-1.25) | -2.11 (-3.35--0.86) |
| Mongolia | 68 (18-187) | 1.96 (0.52-5.4) | 288 (81-564) | 7.84 (2.21-15.36) | 5.99 (5.24-6.74) |
| Montenegro | 28 (6-57) | 5.82 (1.35-11.92) | 5 (1-11) | 1.46 (0.31-3.39) | -4.83 (-5.49--4.17) |
| Morocco | 1435 (483-2865) | 3.8 (1.28-7.6) | 1135 (457-2066) | 3.7 (1.49-6.73) | -0.51 (-1.09-0.08) |
| Mozambique | 4685 (2605-7577) | 16.11 (8.96-26.02) | 6441 (3184-11496) | 12.16 (6.01-21.71) | -0.88 (-1.86-0.11) |
| Myanmar | 12403 (4928-26205) | 23.23 (9.23-49.11) | 17997 (5526-38830) | 34.99 (10.74-75.49) | 0.84 (0.56-1.12) |
| Namibia | 467 (139-1118) | 19.02 (5.66-45.55) | 756 (169-1626) | 27.45 (6.15-59.04) | 1.81 (1.2-2.42) |
| Nauru | 0 (0-1) | 2.58 (0.21-6.97) | 0 (0-1) | 1.76 (0.04-4.77) | -2.39 (-2.98--1.79) |
| Nepal | 6280 (2562-12944) | 16.77 (6.86-34.59) | 4473 (2043-8090) | 14.53 (6.63-26.27) | -1.56 (-2.12--1) |
| Netherlands | 369 (134-633) | 3.87 (1.4-6.65) | 148 (16-338) | 1.72 (0.19-3.94) | -2.82 (-3.6--2.03) |
| New Zealand | 14 (3-29) | 0.47 (0.1-0.99) | 16 (3-35) | 0.54 (0.09-1.18) | 1.88 (0.87-2.9) |
| Nicaragua | 1228 (470-2599) | 17.99 (6.89-38.08) | 592 (197-1314) | 9.6 (3.19-21.31) | -2.15 (-2.56--1.75) |
| Niger | 11698 (5637-22319) | 55.06 (26.52-104.89) | 17354 (7404-33997) | 30.66 (13.09-60.05) | -1.96 (-3.18--0.73) |
| Nigeria | 121677 (60798-202493) | 62.53 (31.23-104) | 248875 (105449-445478) | 63.07 (26.73-112.87) | 0.82 (-0.21-1.86) |
| Niue | 0 (0-0) | 1.76 (0.33-4.58) | 0 (0-0) | 4.75 (0.15-12.46) | 0.06 (-0.69-0.81) |
| North Macedonia | 9 (2-20) | 0.55 (0.13-1.21) | 7 (2-15) | 0.78 (0.21-1.65) | 1.51 (0.45-2.57) |
| Northern Mariana Islands | 1 (0-1) | 1 (0.11-2.52) | 0 (0-0) | 0.4 (0.04-1) | -3.87 (-4.49--3.23) |
| Norway | 18 (12-26) | 0.63 (0.42-0.89) | 3 (1-5) | 0.11 (0.05-0.2) | -4.9 (-6.34--3.44) |
| Oman | 379 (178-661) | 10.98 (5.14-19.14) | 200 (100-348) | 5.23 (2.63-9.1) | -2.71 (-3.19--2.24) |
| Pakistan | 43634 (19371-78870) | 21.78 (9.67-39.37) | 95205 (43222-177077) | 31.98 (14.53-59.49) | 0.69 (0.05-1.33) |
| Palau | 0 (0-0) | 1.19 (0.05-2.94) | 0 (0-0) | 0.77 (0.05-1.96) | -2.14 (-2.69--1.58) |
| Palestine | 383 (170-673) | 9.05 (4.02-15.92) | 534 (261-920) | 9.23 (4.51-15.89) | -1 (-1.78--0.22) |
| Panama | 446 (112-846) | 15.71 (3.95-29.78) | 301 (76-603) | 8.86 (2.25-17.72) | -2.24 (-2.54--1.93) |
| Papua New Guinea | 191 (32-592) | 2.7 (0.46-8.38) | 578 (130-1553) | 3.53 (0.79-9.48) | 0.56 (-0.13-1.25) |
| Paraguay | 1058 (213-2858) | 17.03 (3.44-46.03) | 378 (69-920) | 6.09 (1.11-14.81) | -4.95 (-5.34--4.56) |
| Peru | 25747 (10256-48607) | 83.48 (33.28-157.64) | 11686 (4596-20571) | 36.09 (14.19-63.57) | -2.34 (-2.69--1.99) |
| Philippines | 31558 (14986-55044) | 32.55 (15.46-56.77) | 29979 (13968-48645) | 27.45 (12.79-44.55) | -1.43 (-1.87--0.99) |
| Poland | 1944 (823-3016) | 7.38 (3.13-11.46) | 120 (82-170) | 0.73 (0.5-1.03) | -9.16 (-10.24--8.07) |
| Portugal | 200 (50-385) | 3.61 (0.89-6.95) | 28 (3-67) | 0.7 (0.08-1.7) | -5.21 (-6.23--4.17) |
| Puerto Rico | 100 (15-217) | 3.16 (0.48-6.88) | 26 (0-60) | 2.95 (0-6.81) | -1.71 (-2.83--0.58) |
| Qatar | 16 (9-27) | 2.91 (1.56-4.93) | 17 (9-28) | 0.92 (0.47-1.5) | -4.82 (-5.21--4.43) |
| Republic of C么te d'Ivoire | 16800 (7618-30206) | 63.63 (28.82-114.21) | 18879 (8091-39040) | 41.68 (17.86-86.13) | -1.16 (-2.05--0.26) |
| Republic of Korea | 1765 (750-3143) | 5.45 (2.32-9.71) | 219 (81-404) | 1.7 (0.62-3.13) | -5.52 (-6.58--4.44) |
| Republic of Moldova | 444 (130-980) | 11.63 (3.41-25.7) | 152 (47-309) | 11.16 (3.43-22.7) | 0.09 (-1.14-1.33) |
| Romania | 176 (50-334) | 1.21 (0.34-2.29) | 26 (10-47) | 0.3 (0.11-0.54) | -5.04 (-6.16--3.9) |
| Russian Federation | 6702 (5433-7789) | 7.02 (5.69-8.16) | 2393 (1859-2941) | 3.66 (2.84-4.5) | -2.82 (-3.8--1.83) |
| Rwanda | 3166 (1428-6071) | 20.86 (9.42-39.95) | 1395 (509-2813) | 7.88 (2.88-15.89) | -3.35 (-4.13--2.56) |
| Saint Kitts and Nevis | 1 (0-2) | 2.44 (0.54-5.05) | 1 (0-2) | 2.43 (0.16-5.57) | -1.3 (-1.87--0.73) |
| Saint Lucia | 22 (7-43) | 13.08 (3.95-24.81) | 17 (7-32) | 21.41 (8.56-38.98) | 0.34 (-0.33-1.02) |
| Saint Vincent and the Grenadines | 22 (7-45) | 17.82 (5.61-36.02) | 16 (7-29) | 26.19 (10.54-45.67) | 0.39 (-0.19-0.97) |
| Samoa | 3 (0-8) | 1 (0.19-3.24) | 3 (1-9) | 1.12 (0.2-3.02) | 0.68 (0.15-1.22) |
| San Marino | 0 (0-1) | 3.05 (0.63-6.72) | 0 (0-0) | 0.63 (0.04-2.07) | -5.1 (-6.1--4.08) |
| Sao Tome and Principe | 30 (15-53) | 13.39 (6.6-24.08) | 28 (10-61) | 11.95 (4.35-25.76) | -0.1 (-0.83-0.63) |
| Saudi Arabia | 7798 (3709-13820) | 31.76 (15.11-56.29) | 1787 (872-3077) | 7.99 (3.89-13.76) | -6.2 (-6.71--5.69) |
| Senegal | 11007 (5089-19679) | 66.73 (30.86-119.41) | 5518 (2247-11391) | 23.94 (9.75-49.42) | -4.04 (-4.99--3.09) |
| Serbia | 375 (83-762) | 5.67 (1.25-11.52) | 44 (16-79) | 1.37 (0.51-2.45) | -5.99 (-7--4.97) |
| Seychelles | 7 (1-17) | 9.15 (1.58-21.35) | 6 (1-14) | 8 (1.19-18.18) | -1.47 (-1.79--1.15) |
| Sierra Leone | 4481 (2155-8585) | 46.36 (22.25-88.74) | 3737 (1831-6911) | 25.88 (12.66-47.85) | -1.63 (-2.59--0.67) |
| Singapore | 24 (12-37) | 0.98 (0.5-1.53) | 8 (3-14) | 0.3 (0.1-0.52) | -4.56 (-5.43--3.67) |
| Slovakia | 22 (10-37) | 0.57 (0.25-0.96) | 7 (2-14) | 0.25 (0.08-0.51) | -3.06 (-4.03--2.09) |
| Slovenia | 2 (0-5) | 0.18 (0-0.47) | 2 (0-5) | 0.21 (0.02-0.55) | 0.3 (-1.21-1.84) |
| Solomon Islands | 9 (2-27) | 1.39 (0.31-4.16) | 13 (3-33) | 1.29 (0.35-3.4) | -0.86 (-1.6--0.12) |
| Somalia | 2323 (1017-4060) | 12.6 (5.52-22) | 5412 (2343-9943) | 11.78 (5.1-21.65) | 0.63 (-0.54-1.82) |
| South Africa | 12785 (6403-20757) | 25.47 (12.75-41.34) | 15981 (9776-23193) | 33.81 (20.68-49.07) | 0.54 (0.28-0.8) |
| South Sudan | 6623 (1856-15388) | 54.26 (15.21-126.2) | 4262 (1617-9992) | 23.35 (8.87-54.73) | -1.87 (-2.98--0.76) |
| Spain | 657 (287-1074) | 3.42 (1.5-5.58) | 156 (23-324) | 0.97 (0.14-2) | -3.84 (-4.92--2.74) |
| Sri Lanka | 1216 (407-2719) | 7.04 (2.36-15.74) | 1118 (202-2336) | 7.69 (1.38-16.08) | -0.27 (-0.63-0.1) |
| Sudan | 1521 (656-2864) | 3.65 (1.58-6.89) | 2909 (1265-5661) | 5.28 (2.29-10.27) | 1.54 (0.71-2.38) |
| Suriname | 223 (61-426) | 50.85 (13.81-96.99) | 220 (97-392) | 51.56 (22.64-91.7) | -0.35 (-0.57--0.13) |
| Sweden | 78 (25-148) | 1.31 (0.41-2.48) | 19 (2-42) | 0.34 (0.04-0.77) | -4.63 (-5.51--3.74) |
| Switzerland | 78 (24-138) | 1.92 (0.6-3.4) | 34 (2-78) | 0.8 (0.04-1.83) | -3.36 (-4.28--2.43) |
| Syrian Arab Republic | 1995 (877-3895) | 9.06 (3.98-17.67) | 341 (138-768) | 3.6 (1.45-8.1) | -3.68 (-4.26--3.1) |
| Taiwan (Province of China) | 28 (8-51) | 0.18 (0.05-0.33) | 144 (44-274) | 1.89 (0.58-3.61) | 8.02 (6.72-9.34) |
| Tajikistan | 392 (95-927) | 3.92 (0.95-9.27) | 615 (247-1243) | 4.52 (1.81-9.13) | 1.63 (0.76-2.51) |
| Thailand | 11431 (3445-24994) | 22.91 (6.9-50.09) | 2709 (1160-4451) | 10.46 (4.48-17.18) | -4.19 (-5.02--3.35) |
| Timor-Leste | 214 (60-485) | 12.84 (3.63-29.12) | 348 (85-876) | 17.79 (4.34-44.74) | 0.82 (0.04-1.6) |
| Togo | 3143 (1571-5698) | 41.03 (20.5-74.32) | 2574 (1181-4878) | 22.13 (10.16-41.93) | -1.93 (-2.79--1.07) |
| Tokelau | 0 (0-0) | 3.24 (0.17-8.84) | 0 (0-0) | 12.31 (0-38.26) | -1.13 (-2.32-0.07) |
| Tonga | 2 (0-6) | 1.3 (0.25-3.71) | 2 (1-6) | 1.73 (0.35-4.42) | 0.44 (-0.15-1.05) |
| Trinidad and Tobago | 325 (125-605) | 28.31 (10.88-52.66) | 192 (73-366) | 26.8 (10.19-51.21) | -0.75 (-1.33--0.17) |
| Tunisia | 360 (144-698) | 3.42 (1.37-6.63) | 191 (72-449) | 2.37 (0.89-5.57) | -1.84 (-2.23--1.45) |
| Turkey | 17551 (6851-31249) | 24.76 (9.67-44.07) | 4436 (1794-8203) | 9.12 (3.69-16.87) | -4.21 (-4.53--3.9) |
| Turkmenistan | 337 (120-636) | 5.61 (1.99-10.57) | 331 (132-580) | 6.25 (2.5-10.94) | 0.56 (0.08-1.04) |
| Tuvalu | 0 (0-0) | 0.31 (0.07-0.94) | 0 (0-0) | 0.37 (0.04-1.16) | -0.72 (-1.15--0.29) |
| Uganda | 6382 (3565-11290) | 14.5 (8.11-25.64) | 11986 (5849-22085) | 15.86 (7.74-29.21) | 0.15 (-0.96-1.27) |
| Ukraine | 2241 (922-3809) | 6.94 (2.86-11.8) | 531 (108-1028) | 4.01 (0.81-7.76) | -2.17 (-3.28--1.04) |
| United Arab Emirates | 42 (14-105) | 1.8 (0.61-4.5) | 34 (15-72) | 0.92 (0.42-1.95) | -2.26 (-3.05--1.46) |
| United Kingdom | 486 (412-570) | 1.27 (1.07-1.49) | 86 (67-110) | 0.26 (0.2-0.33) | -6.29 (-7.05--5.53) |
| United Republic of Tanzania | 6314 (3302-11316) | 10.99 (5.76-19.67) | 15351 (6795-30589) | 17 (7.53-33.88) | 2.32 (1.34-3.3) |
| United States of America | 4194 (3466-4950) | 2.09 (1.73-2.47) | 1508 (1113-1946) | 0.85 (0.63-1.09) | -3.73 (-4.38--3.08) |
| United States Virgin Islands | 3 (1-7) | 2.66 (0.52-6) | 1 (0-1) | 1.55 (0.13-3.81) | -2.66 (-3.27--2.06) |
| Uruguay | 266 (85-503) | 9.89 (3.15-18.7) | 62 (13-131) | 3.6 (0.77-7.65) | -4.81 (-5.37--4.25) |
| Uzbekistan | 1198 (438-2563) | 3.51 (1.29-7.52) | 5209 (2632-8508) | 13.59 (6.87-22.21) | 5.22 (4.63-5.82) |
| Vanuatu | 3 (1-7) | 0.91 (0.19-2.55) | 5 (1-13) | 1.17 (0.3-3.08) | 0.92 (0.18-1.66) |
| Venezuela (Bolivarian Republic of) | 9216 (3939-15474) | 34.89 (14.91-58.59) | 3449 (1079-6457) | 15.87 (4.97-29.72) | -2.73 (-3.07--2.38) |
| Viet Nam | 5077 (1862-10469) | 5.47 (2.01-11.27) | 7322 (2466-15176) | 9.81 (3.3-20.34) | 1.79 (1.46-2.12) |
| Yemen | 1660 (705-2936) | 5.3 (2.25-9.38) | 3003 (1341-5539) | 6.45 (2.88-11.89) | 0.39 (-0.57-1.35) |
| Zambia | 2971 (1349-5198) | 16.28 (7.39-28.49) | 6045 (2420-12989) | 20.87 (8.36-44.85) | 0.45 (-0.64-1.55) |
| Zimbabwe | 1991 (796-3997) | 10.72 (4.29-21.53) | 1991 (963-3808) | 8.9 (4.3-17.01) | -0.36 (-1.05-0.34) |

Abbreviations: UI: uncertainty intervals; CI: confidence interval; EAPC: estimated annual percentage change.

Table S3: Decomposition analysis of change in Deaths and DALYs.

|  | Deaths | | | | DALYs | | | |
| --- | --- | --- | --- | --- | --- | --- | --- | --- |
| Location | overll difference | Aging | Population | Epidemiological change | overll difference | Aging | Population | Epidemiological change |
| **Air pollution** |  |  |  |  |  |  |  |  |
| Global | 40294.84 | 0 | 250628.85 (621.99%) | -210334.007  (-521.99%) | 3623017.97 | 0 | 22555688.928 (622.57%) | -18932670.959 (-522.57%) |
| High-middle SDI | -9927.92 | 0 | 2296.3  (-23.13%) | -12224.217 (123.13%) | -894161.9 | 0 | 206809.032  (-23.13%) | -1100970.929 (123.13%) |
| Low-middle SDI | -31453.47 | 0 | 104196.994  (-331.27%) | -135650.465  (431.27%) | -2831028.95 | 0 | 9377366.983  (-331.24%) | -12208395.935 (431.24%) |
| High SDI | -2436.23 | 0 | 580.898  (-23.84%) | -3017.129  (123.84%) | -219343.9 | 0 | 52325.856  (-23.86%) | -271669.752 (123.86%) |
| Low SDI | 63061.15 | 0 | 147854.456 (234.46%) | -84793.307  (-134.46%) | 5674309.4 | 0 | 13305678.995 (234.49%) | -7631369.59  (-134.49%) |
| Middle SDI | -28876.51 | 0 | 29511.363  (-102.2%) | -58387.876  (202.2%) | -2599243.6 | 0 | 2656192.248  (-102.19%) | -5255435.846 (202.19%) |
| **Particulate matter pollution** |  |  |  |  |  |  |  |  |
| Global | 40294.84 | 0 | 250628.85 (621.99%) | -210334.007  (-521.99%) | 3623017.97 | 0 | 22555688.928 (622.57%) | -18932670.959 (-522.57%) |
| High-middle SDI | -9927.92 | 0 | 2296.3  (-23.13%) | -12224.217 (123.13%) | -894161.9 | 0 | 206809.032  (-23.13%) | -1100970.929 (123.13%) |
| Low-middle SDI | -31453.47 | 0 | 104196.994  (-331.27%) | -135650.465 (431.27%) | -2831028.95 | 0 | 9377366.983  (-331.24%) | -12208395.935 (431.24%) |
| High SDI | -2436.23 | 0 | 580.898  (-23.84%) | -3017.129  (123.84%) | -219343.9 | 0 | 52325.856  (-23.86%) | -271669.752 (123.86%) |
| Low SDI | 63061.15 | 0 | 147854.456 (234.46%) | -84793.307  (-134.46%) | 5674309.4 | 0 | 13305678.995 (234.49%) | -7631369.59  (-134.49%) |
| Middle SDI | -28876.51 | 0 | 29511.363  (-102.2%) | -58387.876  (202.2%) | -2599243.6 | 0 | 2656192.248  (-102.19%) | -5255435.846 (202.19%) |
| **Household air pollution from solid fuels** |  |  |  |  |  |  |  |  |
| Global | -17034.57 | 0 | 201312.95  (-1181.79%) | -218347.515 (1281.79%) | -1535914.84 | 0 | 18117126.347  (-1179.57%) | -19653041.188 (1279.57%) |
| High-middle SDI | -6413.91 | 0 | 762.243  (-11.88%) | -7176.15  (111.88%) | -577713.36 | 0 | 68656.342  (-11.88%) | -646369.699 (111.88%) |
| Low-middle SDI | -53364.36 | 0 | 85194.031  (-159.65%) | -138558.395 (259.65%) | -4802958.92 | 0 | 7667101.953  (-159.63%) | -12470060.875 (259.63%) |
| High SDI | -326.65 | 0 | 40.314  (-12.34%) | -366.964  (112.34%) | -29415.82 | 0 | 3630.417  (-12.34%) | -33046.239 (112.34%) |
| Low SDI | 48734.56 | 0 | 128515.272 (263.7%) | -79780.715  (-163.7%) | 4385123.02 | 0 | 11565327.136 (263.74%) | -7180204.119  (-163.74%) |
| Middle SDI | -43552.14 | 0 | 17365.617  (-39.87%) | -60917.76  (139.87%) | -3920051.78 | 0 | 1562974.28  (-39.87%) | -5483026.056 (139.87%) |
| **Ambient particulate matter pollution** |  |  |  |  |  |  |  |  |
| Global | 57282.97 | 0 | 49281.578 (86.03%) | 8001.394  (13.97%) | 5154754.26 | 0 | 4435473.569 (86.05%) | 719280.687 (13.95%) |
| High-middle SDI | -3509.64 | 0 | 1533.166  (-43.68%) | -5042.801  (143.68%) | -316054.81 | 0 | 138072.404  (-43.69%) | -454127.218 (143.69%) |
| Low-middle SDI | 21892.84 | 0 | 18992.228 (86.75%) | 2900.609  (13.25%) | 1970305.17 | 0 | 1709298.797 (86.75%) | 261006.37 (13.25%) |
| High SDI | -2108.78 | 0 | 540.438  (-25.63%) | -2649.213  (125.63%) | -189855.55 | 0 | 48682.274  (-25.64%) | -238537.822 (125.64%) |
| Low SDI | 14311.62 | 0 | 19324.376 (135.03%) | -5012.76  (-35.03%) | 1287838.69 | 0 | 1739019.247 (135.03%) | -451180.557  (-35.03%) |
| Middle SDI | 14670.73 | 0 | 12136.059 (82.72%) | 2534.676  (17.28%) | 1320367.59 | 0 | 1092346.126 (82.73%) | 228021.465 (17.27%) |

Table S4: Projection analysis of deaths and DALYs for neonatal sepsis and other neonatal infections attributable to air pollution from 2022 to 2050.

| **Air pollution** | | | | | |
| --- | --- | --- | --- | --- | --- |
| year | sex | Age-standardized deaths rate | Numer of deaths cases | Age-standardized DALYs rate | Numer of DALYs cases |
| 2022 | male | 0.995819524 | 31386.58725 | 89.61873025 | 2824625.035 |
| 2023 | male | 0.996689679 | 30939.27567 | 89.69798186 | 2784389.981 |
| 2024 | male | 0.997559834 | 30491.96408 | 89.77723347 | 2744154.928 |
| 2025 | male | 0.998429989 | 30044.6525 | 89.85648509 | 2703919.874 |
| 2026 | male | 0.999300144 | 29597.34091 | 89.9357367 | 2663684.821 |
| 2027 | male | 1.000170299 | 29150.02933 | 90.01498831 | 2623449.767 |
| 2028 | male | 1.001040454 | 28702.71775 | 90.09423992 | 2583214.714 |
| 2029 | male | 1.001910609 | 28255.40616 | 90.17349153 | 2542979.66 |
| 2030 | male | 1.002780764 | 27808.09458 | 90.25274314 | 2502744.607 |
| 2031 | male | 1.00365092 | 27360.78299 | 90.33199475 | 2462509.554 |
| 2032 | male | 1.004521075 | 26913.47141 | 90.41124636 | 2422274.5 |
| 2033 | male | 1.00539123 | 26466.15982 | 90.49049797 | 2382039.447 |
| 2034 | male | 1.006261385 | 26018.84824 | 90.56974958 | 2341804.393 |
| 2035 | male | 1.00713154 | 25571.53666 | 90.64900119 | 2301569.34 |
| 2036 | male | 1.008001695 | 25124.22507 | 90.72825281 | 2261334.286 |
| 2037 | male | 1.00887185 | 24676.91349 | 90.80750442 | 2221099.233 |
| 2038 | male | 1.009742005 | 24229.6019 | 90.88675603 | 2180864.179 |
| 2039 | male | 1.01061216 | 23782.29032 | 90.96600764 | 2140629.126 |
| 2040 | male | 1.011482315 | 23334.97873 | 91.04525925 | 2100394.073 |
| 2041 | male | 1.012352471 | 22887.66715 | 91.12451086 | 2060159.019 |
| 2042 | male | 1.013222626 | 22440.35557 | 91.20376247 | 2019923.966 |
| 2043 | male | 1.014092781 | 21993.04398 | 91.28301408 | 1979688.912 |
| 2044 | male | 1.014962936 | 21545.7324 | 91.36226569 | 1939453.859 |
| 2045 | male | 1.015833091 | 21098.42081 | 91.4415173 | 1899218.805 |
| 2046 | male | 1.016703246 | 20651.10923 | 91.52076891 | 1858983.752 |
| 2047 | male | 1.017573401 | 20203.79765 | 91.60002053 | 1818748.698 |
| 2048 | male | 1.018443556 | 19756.48606 | 91.67927214 | 1778513.645 |
| 2049 | male | 1.019313711 | 19309.17448 | 91.75852375 | 1738278.592 |
| 2050 | male | 1.020183867 | 18861.86289 | 91.83777536 | 1698043.538 |
| 2022 | female | 0.734320057 | 21097.33593 | 66.08304084 | 1898593.58 |
| 2023 | female | 0.72619411 | 20571.51378 | 65.35172673 | 1851288.071 |
| 2024 | female | 0.718068162 | 19621.81614 | 64.62041262 | 1765820.263 |
| 2025 | female | 0.709942215 | 18988.09004 | 63.88909851 | 1708803.082 |
| 2026 | female | 0.701816267 | 18118.82776 | 63.1577844 | 1630575.487 |
| 2027 | female | 0.69369032 | 17425.14235 | 62.42647029 | 1568160.606 |
| 2028 | female | 0.685564373 | 16600.57582 | 61.69515618 | 1493957.085 |
| 2029 | female | 0.677438425 | 15873.57264 | 60.96384206 | 1428542.191 |
| 2030 | female | 0.669312478 | 15073.84235 | 60.23252795 | 1356575.23 |
| 2031 | female | 0.66118653 | 14328.32537 | 59.50121384 | 1289492.943 |
| 2032 | female | 0.653060583 | 13542.3959 | 58.76989973 | 1218769.051 |
| 2033 | female | 0.644934636 | 12786.59131 | 58.03858562 | 1150760.036 |
| 2034 | female | 0.636808688 | 12008.33059 | 57.30727151 | 1080727.035 |
| 2035 | female | 0.628682741 | 11246.80945 | 56.5759574 | 1012202.949 |
| 2036 | female | 0.620556793 | 10472.81004 | 55.84464329 | 942553.9419 |
| 2037 | female | 0.612430846 | 9708.112366 | 55.11332918 | 873743.5822 |
| 2038 | female | 0.604304899 | 8936.480855 | 54.38201507 | 804307.9969 |
| 2039 | female | 0.596178951 | 8170.018069 | 53.65070096 | 735338.5276 |
| 2040 | female | 0.588053004 | 7399.702335 | 52.91938685 | 666021.5612 |
| 2041 | female | 0.579927057 | 6632.258723 | 52.18807274 | 596963.6596 |
| 2042 | female | 0.571801109 | 5862.674131 | 51.45675862 | 527712.6209 |
| 2043 | female | 0.563675162 | 5094.6855 | 50.72544451 | 458605.5691 |
| 2044 | female | 0.555549214 | 4325.507185 | 49.9941304 | 389391.1727 |
| 2045 | female | 0.547423267 | 3557.215701 | 49.26281629 | 320256.8035 |
| 2046 | female | 0.53929732 | 2788.263142 | 48.53150218 | 251062.7726 |
| 2047 | female | 0.531171372 | 2019.803372 | 47.80018807 | 181913.2205 |
| 2048 | female | 0.523045425 | 1250.97626 | 47.06887396 | 112730.5088 |
| 2049 | female | 0.514919477 | 482.4229769 | 46.33755985 | 43572.5181 |
| 2050 | female | 0.50679353 | -286.3344278 | 45.60624574 | -25603.90254 |

Table S5: Projection analysis of deaths and DALYs for neonatal sepsis and other neonatal infections attributable to particulate matter pollution from 2022 to 2050.

| **Particulate matter pollution** | | | | | |
| --- | --- | --- | --- | --- | --- |
| year | sex | Age-standardized deaths rate | Numer of deaths cases | Age-standardized DALYs rate | Numer of DALYs cases |
| 2022 | male | 0.9958195 | 31386.587 | 89.61873 | 2824625 |
| 2023 | male | 0.9966897 | 30939.276 | 89.697982 | 2784390 |
| 2024 | male | 0.9975598 | 30491.964 | 89.777233 | 2744154.9 |
| 2025 | male | 0.99843 | 30044.652 | 89.856485 | 2703919.9 |
| 2026 | male | 0.9993001 | 29597.341 | 89.935737 | 2663684.8 |
| 2027 | male | 1.0001703 | 29150.029 | 90.014988 | 2623449.8 |
| 2028 | male | 1.0010405 | 28702.718 | 90.09424 | 2583214.7 |
| 2029 | male | 1.0019106 | 28255.406 | 90.173492 | 2542979.7 |
| 2030 | male | 1.0027808 | 27808.095 | 90.252743 | 2502744.6 |
| 2031 | male | 1.0036509 | 27360.783 | 90.331995 | 2462509.6 |
| 2032 | male | 1.0045211 | 26913.471 | 90.411246 | 2422274.5 |
| 2033 | male | 1.0053912 | 26466.16 | 90.490498 | 2382039.4 |
| 2034 | male | 1.0062614 | 26018.848 | 90.56975 | 2341804.4 |
| 2035 | male | 1.0071315 | 25571.537 | 90.649001 | 2301569.3 |
| 2036 | male | 1.0080017 | 25124.225 | 90.728253 | 2261334.3 |
| 2037 | male | 1.0088719 | 24676.913 | 90.807504 | 2221099.2 |
| 2038 | male | 1.009742 | 24229.602 | 90.886756 | 2180864.2 |
| 2039 | male | 1.0106122 | 23782.29 | 90.966008 | 2140629.1 |
| 2040 | male | 1.0114823 | 23334.979 | 91.045259 | 2100394.1 |
| 2041 | male | 1.0123525 | 22887.667 | 91.124511 | 2060159 |
| 2042 | male | 1.0132226 | 22440.356 | 91.203762 | 2019924 |
| 2043 | male | 1.0140928 | 21993.044 | 91.283014 | 1979688.9 |
| 2044 | male | 1.0149629 | 21545.732 | 91.362266 | 1939453.9 |
| 2045 | male | 1.0158331 | 21098.421 | 91.441517 | 1899218.8 |
| 2046 | male | 1.0167032 | 20651.109 | 91.520769 | 1858983.8 |
| 2047 | male | 1.0175734 | 20203.798 | 91.600021 | 1818748.7 |
| 2048 | male | 1.0184436 | 19756.486 | 91.679272 | 1778513.6 |
| 2049 | male | 1.0193137 | 19309.174 | 91.758524 | 1738278.6 |
| 2050 | male | 1.0201839 | 18861.863 | 91.837775 | 1698043.5 |
| 2022 | female | 0.7343201 | 21097.336 | 66.083041 | 1898593.6 |
| 2023 | female | 0.7261941 | 20571.514 | 65.351727 | 1851288.1 |
| 2024 | female | 0.7180682 | 19621.816 | 64.620413 | 1765820.3 |
| 2025 | female | 0.7099422 | 18988.09 | 63.889099 | 1708803.1 |
| 2026 | female | 0.7018163 | 18118.828 | 63.157784 | 1630575.5 |
| 2027 | female | 0.6936903 | 17425.142 | 62.42647 | 1568160.6 |
| 2028 | female | 0.6855644 | 16600.576 | 61.695156 | 1493957.1 |
| 2029 | female | 0.6774384 | 15873.573 | 60.963842 | 1428542.2 |
| 2030 | female | 0.6693125 | 15073.842 | 60.232528 | 1356575.2 |
| 2031 | female | 0.6611865 | 14328.325 | 59.501214 | 1289492.9 |
| 2032 | female | 0.6530606 | 13542.396 | 58.7699 | 1218769.1 |
| 2033 | female | 0.6449346 | 12786.591 | 58.038586 | 1150760 |
| 2034 | female | 0.6368087 | 12008.331 | 57.307272 | 1080727 |
| 2035 | female | 0.6286827 | 11246.809 | 56.575957 | 1012202.9 |
| 2036 | female | 0.6205568 | 10472.81 | 55.844643 | 942553.94 |
| 2037 | female | 0.6124308 | 9708.1124 | 55.113329 | 873743.58 |
| 2038 | female | 0.6043049 | 8936.4809 | 54.382015 | 804308 |
| 2039 | female | 0.596179 | 8170.0181 | 53.650701 | 735338.53 |
| 2040 | female | 0.588053 | 7399.7023 | 52.919387 | 666021.56 |
| 2041 | female | 0.5799271 | 6632.2587 | 52.188073 | 596963.66 |
| 2042 | female | 0.5718011 | 5862.6741 | 51.456759 | 527712.62 |
| 2043 | female | 0.5636752 | 5094.6855 | 50.725445 | 458605.57 |
| 2044 | female | 0.5555492 | 4325.5072 | 49.99413 | 389391.17 |
| 2045 | female | 0.5474233 | 3557.2157 | 49.262816 | 320256.8 |
| 2046 | female | 0.5392973 | 2788.2631 | 48.531502 | 251062.77 |
| 2047 | female | 0.5311714 | 2019.8034 | 47.800188 | 181913.22 |
| 2048 | female | 0.5230454 | 1250.9763 | 47.068874 | 112730.51 |
| 2049 | female | 0.5149195 | 482.42298 | 46.33756 | 43572.518 |
| 2050 | female | 0.5067935 | -286.3344 | 45.606246 | -25603.9 |

Table S6: Projection analysis of deaths and DALYs for neonatal sepsis and other neonatal infections attributable to household air pollution from 2022 to 2050.

| **Household air pollution from solid fuels** | | | | | |
| --- | --- | --- | --- | --- | --- |
| year | sex | Age-standardized deaths rate | Numer of deaths cases | Age-standardized DALYs rate | Numer of DALYs cases |
| 2022 | male | 0.7595772 | 23929.9773 | 68.356273 | 2153514.4 |
| 2023 | male | 0.7625783 | 23652.93222 | 68.626817 | 2128592.5 |
| 2024 | male | 0.7655794 | 23375.88713 | 68.897362 | 2103670.5 |
| 2025 | male | 0.7685805 | 23098.84204 | 69.167907 | 2078748.6 |
| 2026 | male | 0.7715816 | 22821.79695 | 69.438452 | 2053826.7 |
| 2027 | male | 0.7745827 | 22544.75187 | 69.708997 | 2028904.7 |
| 2028 | male | 0.7775838 | 22267.70678 | 69.979541 | 2003982.8 |
| 2029 | male | 0.7805849 | 21990.66169 | 70.250086 | 1979060.9 |
| 2030 | male | 0.783586 | 21713.6166 | 70.520631 | 1954138.9 |
| 2031 | male | 0.7865871 | 21436.57152 | 70.791176 | 1929217 |
| 2032 | male | 0.7895882 | 21159.52643 | 71.061721 | 1904295.1 |
| 2033 | male | 0.7925893 | 20882.48134 | 71.332265 | 1879373.1 |
| 2034 | male | 0.7955904 | 20605.43625 | 71.60281 | 1854451.2 |
| 2035 | male | 0.7985915 | 20328.39117 | 71.873355 | 1829529.3 |
| 2036 | male | 0.8015926 | 20051.34608 | 72.1439 | 1804607.3 |
| 2037 | male | 0.8045937 | 19774.30099 | 72.414445 | 1779685.4 |
| 2038 | male | 0.8075948 | 19497.2559 | 72.684989 | 1754763.5 |
| 2039 | male | 0.8105959 | 19220.21081 | 72.955534 | 1729841.5 |
| 2040 | male | 0.813597 | 18943.16573 | 73.226079 | 1704919.6 |
| 2041 | male | 0.8165981 | 18666.12064 | 73.496624 | 1679997.7 |
| 2042 | male | 0.8195992 | 18389.07555 | 73.767169 | 1655075.7 |
| 2043 | male | 0.8226003 | 18112.03046 | 74.037714 | 1630153.8 |
| 2044 | male | 0.8256014 | 17834.98538 | 74.308258 | 1605231.9 |
| 2045 | male | 0.8286025 | 17557.94029 | 74.578803 | 1580309.9 |
| 2046 | male | 0.8316036 | 17280.8952 | 74.849348 | 1555388 |
| 2047 | male | 0.8346047 | 17003.85011 | 75.119893 | 1530466.1 |
| 2048 | male | 0.8376058 | 16726.80503 | 75.390438 | 1505544.1 |
| 2049 | male | 0.8406069 | 16449.75994 | 75.660982 | 1480622.2 |
| 2050 | male | 0.843608 | 16172.71485 | 75.931527 | 1455700.3 |
| 2022 | female | 0.5535581 | 15992.45217 | 49.815104 | 1439166.2 |
| 2023 | female | 0.5494665 | 15605.76745 | 49.447109 | 1404378.6 |
| 2024 | female | 0.5446343 | 14964.20515 | 49.012439 | 1346641.6 |
| 2025 | female | 0.5400685 | 14508.024 | 48.601759 | 1305599.4 |
| 2026 | female | 0.5354069 | 13917.00883 | 48.182448 | 1252412.4 |
| 2027 | female | 0.5307797 | 13424.06302 | 47.766242 | 1208060.2 |
| 2028 | female | 0.5261402 | 12859.78803 | 47.348919 | 1157281.1 |
| 2029 | female | 0.5215051 | 12347.39318 | 46.931998 | 1111177.3 |
| 2030 | female | 0.5168684 | 11797.26414 | 46.514932 | 1061672.4 |
| 2031 | female | 0.5122322 | 11274.58047 | 46.097918 | 1014641.6 |
| 2032 | female | 0.5075959 | 10731.93484 | 45.680886 | 965811.05 |
| 2033 | female | 0.5029596 | 10203.80822 | 45.26386 | 918289.76 |
| 2034 | female | 0.4983234 | 9665.121432 | 44.846832 | 869816.02 |
| 2035 | female | 0.4936871 | 9134.115414 | 44.429805 | 822035.14 |
| 2036 | female | 0.4890508 | 8597.522911 | 44.012777 | 773750.24 |
| 2037 | female | 0.4844145 | 8064.993648 | 43.59575 | 725831.99 |
| 2038 | female | 0.4797782 | 7529.509052 | 43.178722 | 677647.02 |
| 2039 | female | 0.475142 | 6996.173971 | 42.761695 | 629656.08 |
| 2040 | female | 0.4705057 | 6461.275475 | 42.344667 | 581523.99 |
| 2041 | female | 0.4658694 | 5927.514104 | 41.92764 | 533494.58 |
| 2042 | female | 0.4612331 | 5392.925662 | 41.510612 | 485390.47 |
| 2043 | female | 0.4565968 | 4858.938777 | 41.093584 | 437340.7 |
| 2044 | female | 0.4519606 | 4324.51436 | 40.676557 | 389251.41 |
| 2045 | female | 0.4473243 | 3790.408175 | 40.259529 | 341190.87 |
| 2046 | female | 0.442688 | 3256.070529 | 39.842502 | 293109.41 |
| 2047 | female | 0.4380517 | 2721.901232 | 39.425474 | 245043.17 |
| 2048 | female | 0.4334155 | 2187.609489 | 39.008447 | 196965.85 |
| 2049 | female | 0.4287792 | 1653.406805 | 38.591419 | 148896.59 |
| 2050 | female | 0.4241429 | 1119.139346 | 38.174392 | 100821.48 |

Table S7: Projection analysis of deaths and DALYs for neonatal sepsis and other neonatal infections attributable to ambient particulate matter pollution from 2022 to 2050.

| **Ambient particulate matter pollution** | | | | | |
| --- | --- | --- | --- | --- | --- |
| year | sex | Age-standardized deaths rate | Numer of deaths cases | Age-standardized DALYs rate | Numer of DALYs cases |
| 2022 | male | 0.237123 | 7567.8438 | 21.341986 | 681137.44 |
| 2023 | male | 0.2368771 | 7620.783 | 21.320672 | 685933.48 |
| 2024 | male | 0.2373197 | 7740.3296 | 21.361095 | 696719.07 |
| 2025 | male | 0.2381204 | 7875.9944 | 21.433391 | 708943.65 |
| 2026 | male | 0.2389168 | 7983.8626 | 21.504959 | 718653.05 |
| 2027 | male | 0.239444 | 8038.1558 | 21.552083 | 723529.62 |
| 2028 | male | 0.2395972 | 8034.8063 | 21.565497 | 723212.36 |
| 2029 | male | 0.2394243 | 7987.7795 | 21.549652 | 718963.93 |
| 2030 | male | 0.2390696 | 7920.869 | 21.517595 | 712930.41 |
| 2031 | male | 0.2386997 | 7858.4191 | 21.484333 | 707305.22 |
| 2032 | male | 0.2384422 | 7817.9716 | 21.461297 | 703667.06 |
| 2033 | male | 0.2383534 | 7806.5871 | 21.453474 | 702649.37 |
| 2034 | male | 0.2384182 | 7821.1066 | 21.459445 | 703964.86 |
| 2035 | male | 0.2385743 | 7851.3894 | 21.473561 | 706697.97 |
| 2036 | male | 0.2387453 | 7884.8955 | 21.488947 | 709718.1 |
| 2037 | male | 0.2388701 | 7910.9494 | 21.500126 | 712063.76 |
| 2038 | male | 0.2389193 | 7923.491 | 21.504478 | 713190.08 |
| 2039 | male | 0.2388965 | 7921.8362 | 21.502359 | 713036.88 |
| 2040 | male | 0.2388284 | 7909.6711 | 21.496191 | 711937.5 |
| 2041 | male | 0.2387497 | 7892.9732 | 21.489108 | 710431.3 |
| 2042 | male | 0.2386896 | 7877.7212 | 21.483718 | 709057.11 |
| 2043 | male | 0.2386632 | 7868.1191 | 21.481374 | 708193.34 |
| 2044 | male | 0.2386703 | 7865.7471 | 21.482052 | 707981.68 |
| 2045 | male | 0.2386998 | 7869.6826 | 21.484724 | 708338.28 |
| 2046 | male | 0.2387358 | 7877.3376 | 21.487969 | 709029.41 |
| 2047 | male | 0.2387646 | 7885.6019 | 21.490552 | 709774.55 |
| 2048 | male | 0.2387785 | 7891.8841 | 21.491784 | 710340.26 |
| 2049 | male | 0.2387768 | 7894.7647 | 21.491612 | 710598.9 |
| 2050 | male | 0.2387641 | 7894.1537 | 21.490466 | 710542.75 |
| 2022 | female | 0.18167 | 5418.9915 | 16.350139 | 487701.08 |
| 2023 | female | 0.1856095 | 5593.3545 | 16.704988 | 503403.17 |
| 2024 | female | 0.1884536 | 5775.7538 | 16.961193 | 519825.56 |
| 2025 | female | 0.1919088 | 5985.0885 | 17.272269 | 538672.52 |
| 2026 | female | 0.1932988 | 6110.523 | 17.397364 | 549963.59 |
| 2027 | female | 0.1937163 | 6164.0687 | 17.434821 | 554782.47 |
| 2028 | female | 0.1926727 | 6126.6152 | 17.340733 | 551407.3 |
| 2029 | female | 0.191277 | 6040.0806 | 17.214959 | 543613.69 |
| 2030 | female | 0.1897796 | 5931.2249 | 17.080081 | 533810.41 |
| 2031 | female | 0.1888287 | 5840.456 | 16.994461 | 525636.68 |
| 2032 | female | 0.1884528 | 5786.2725 | 16.960666 | 520757.81 |
| 2033 | female | 0.1886836 | 5778.3548 | 16.981511 | 520045.85 |
| 2034 | female | 0.1892425 | 5807.8793 | 17.031908 | 522705.9 |
| 2035 | female | 0.1899045 | 5859.5474 | 17.091561 | 527360.23 |
| 2036 | female | 0.1904232 | 5913.6097 | 17.138285 | 532229.98 |
| 2037 | female | 0.1906939 | 5954.7011 | 17.162652 | 535931.25 |
| 2038 | female | 0.1906932 | 5973.7423 | 17.162556 | 537646.16 |
| 2039 | female | 0.1904992 | 5970.1451 | 17.145052 | 537321.73 |
| 2040 | female | 0.1902184 | 5949.5754 | 17.119733 | 535468.29 |
| 2041 | female | 0.1899606 | 5921.4456 | 17.096504 | 532933.8 |
| 2042 | female | 0.1897957 | 5895.2222 | 17.081652 | 530571.08 |
| 2043 | female | 0.1897494 | 5877.9138 | 17.077491 | 529011.62 |
| 2044 | female | 0.1898033 | 5872.5076 | 17.082358 | 528524.6 |
| 2045 | female | 0.1899147 | 5878.0398 | 17.092416 | 529023.22 |
| 2046 | female | 0.1900347 | 5890.6518 | 17.103231 | 530159.83 |
| 2047 | female | 0.190125 | 5905.312 | 17.111366 | 531481.04 |
| 2048 | female | 0.1901662 | 5917.4605 | 17.115082 | 532575.9 |
| 2049 | female | 0.1901593 | 5924.2054 | 17.114449 | 533183.79 |
| 2050 | female | 0.1901191 | 5924.7798 | 17.110815 | 533235.54 |

Table S8: ADF test of deaths and DALYs number for Air pollution

|  |  |  | Differencing | Dickey-Fuller | Lag order | p-value |
| --- | --- | --- | --- | --- | --- | --- |
| Air pollution | Deaths | Female | Before | 1.341 | 3 | 0.990 |
| Air pollution | Deaths | Female | After | -3.621 | 3 | 0.048 |
| Air pollution | Deaths | Male | Before | 1.142 | 3 | 0.990 |
| Air pollution | Deaths | Male | After | -3.552 | 3 | 0.054 |
| Air pollution | DALYs | Female | Before | 1.341 | 3 | 0.990 |
| Air pollution | DALYs | Female | After | -3.620 | 3 | 0.048 |
| Air pollution | DALYs | Male | Before | 1.143 | 3 | 0.990 |
| Air pollution | DALYs | Male | After | -3.552 | 3 | 0.054 |
