# Supplementary material for: The global burden of neonatal sepsis attributable to air pollution from 1990 to 2021: findings from the global burden of disease study 2021
Source: Front Public Health. 2025 Sep 24;13:1644191. doi: 10.3389/fpubh.2025.1644191 (PMC12504511; doi:10.3389/fpubh.2025.1644191)
Supplement: Supplementary file 2 [file Presentation_1.zip › Supplementary Figure/Supplementary figure.docx]

**
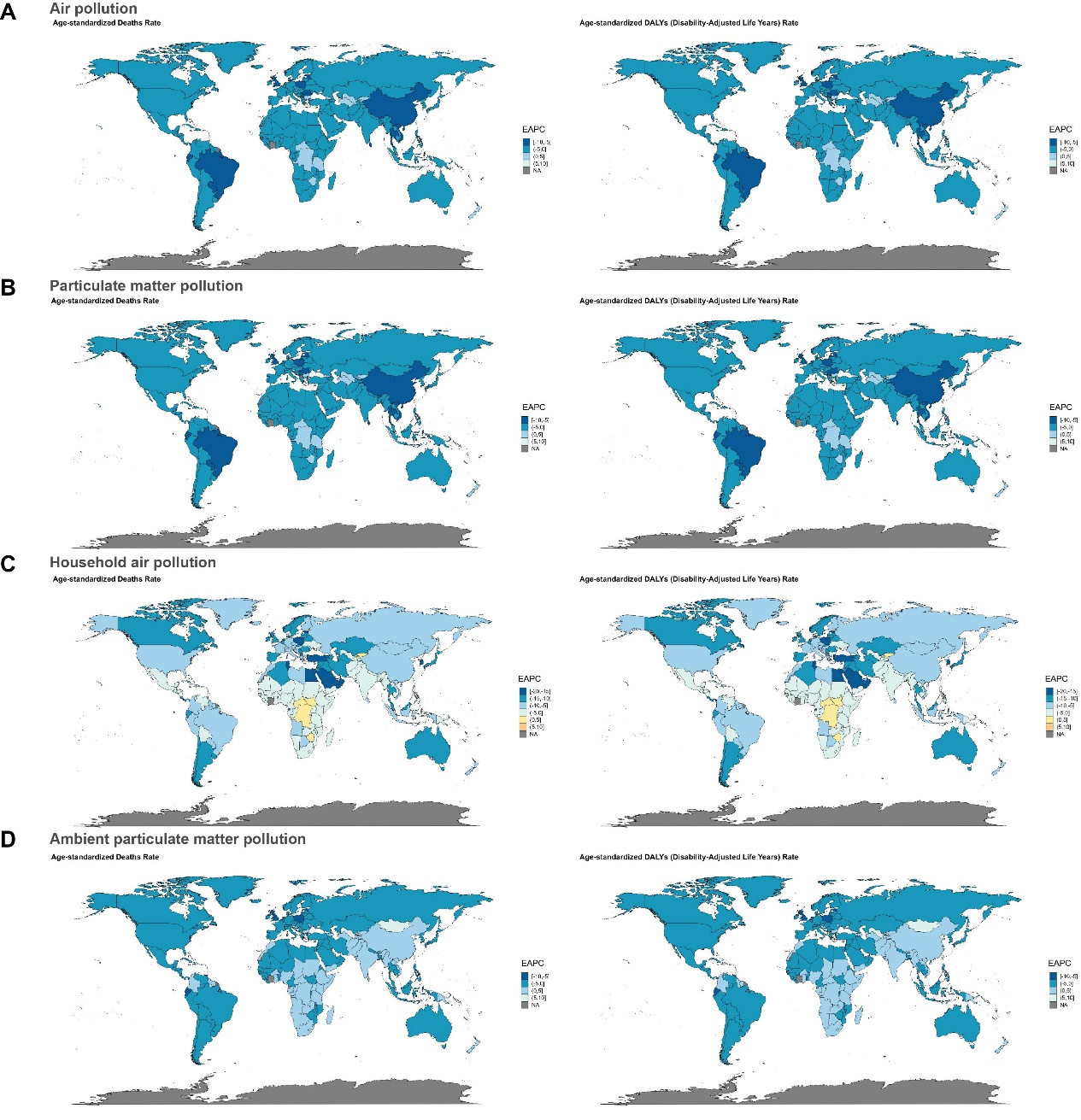
SUPPLEMENTARY FIGURE S1**

The EAPC of neonatal sepsis and other neonatal infections attributable to: (A) air pollution, (B) particulate matter pollution, (C) household air pollution, and (D) ambient particulate matter pollution-related ASR from 1990 to 2019.

**
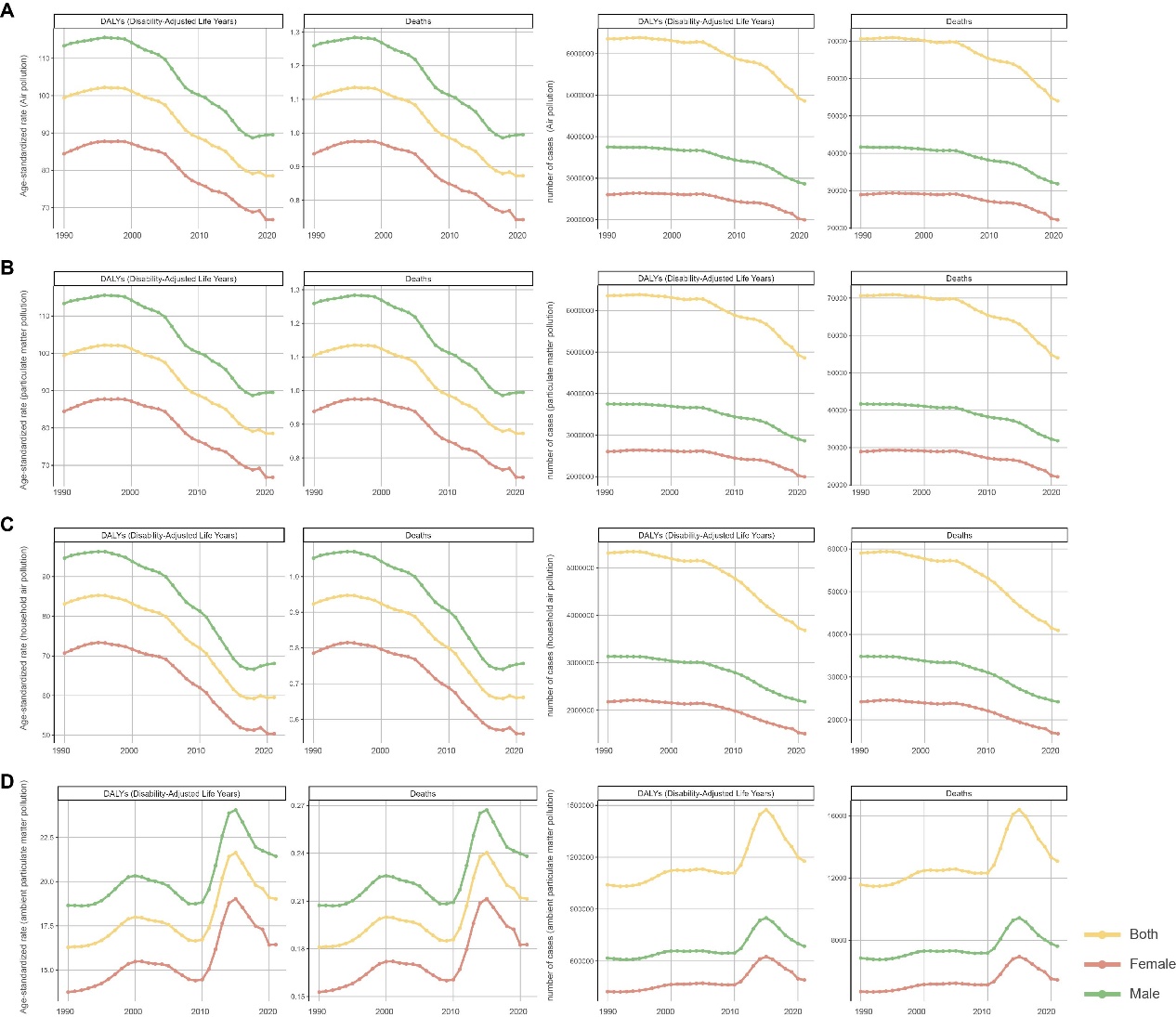
SUPPLEMENTARY FIGURE S2**

Trends in the numbers and age-standardized rates of neonatal sepsis and other neonatal infections deaths and DALYs globally by sex from 1990 to 2021. (A) air pollution, (B) particulate matter pollution, (C) household air pollution, (D) ambient particulate matter pollution.

**
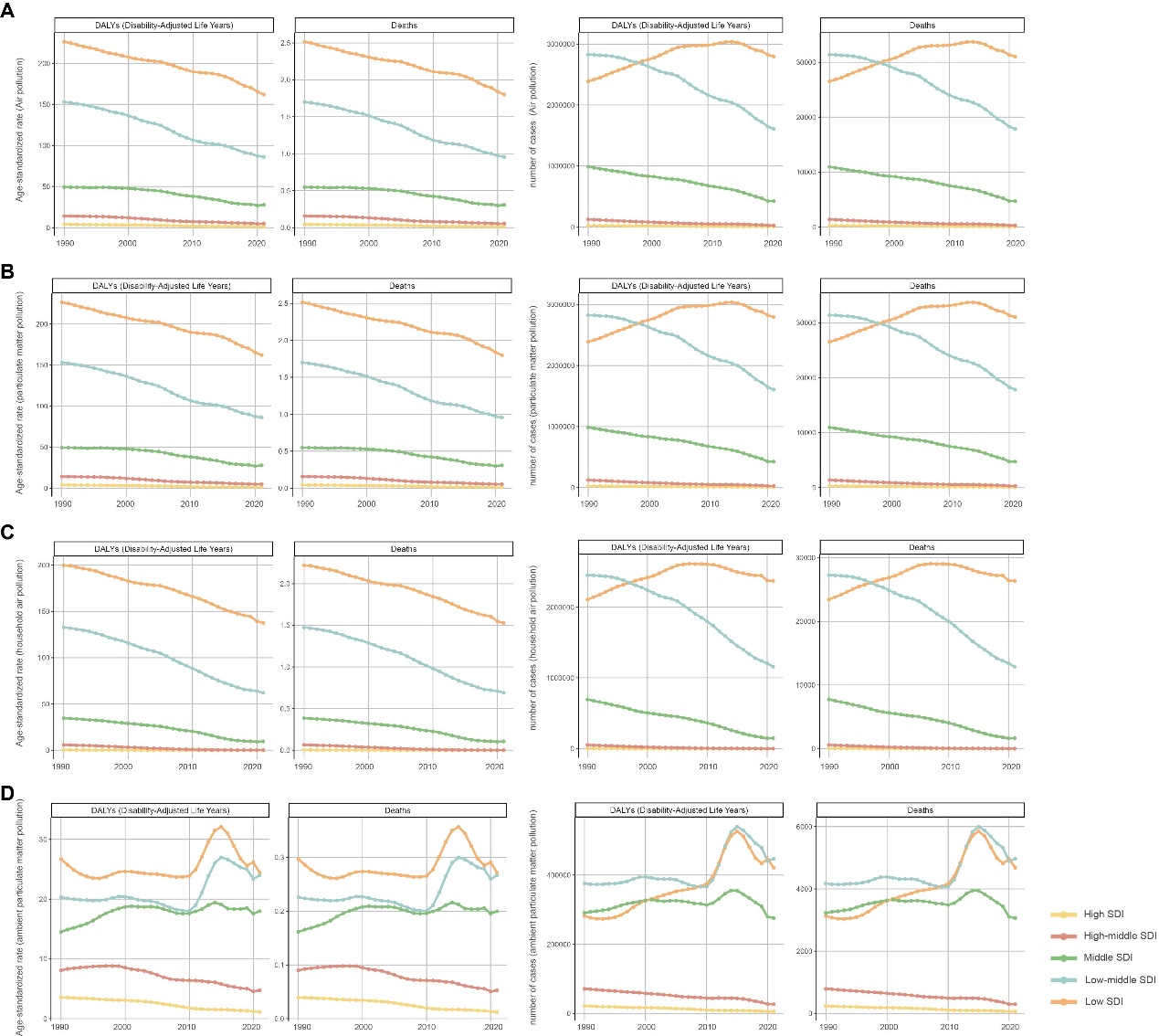
SUPPLEMENTARY FIGURE S3**

Trends in the numbers and age-standardized rates of neonatal sepsis and other neonatal infections deaths and DALYs globally by SDI region from 1990 to 2021. (A) air pollution, (B) particulate matter pollution, (C) household air pollution, (D) ambient particulate matter pollution.

**
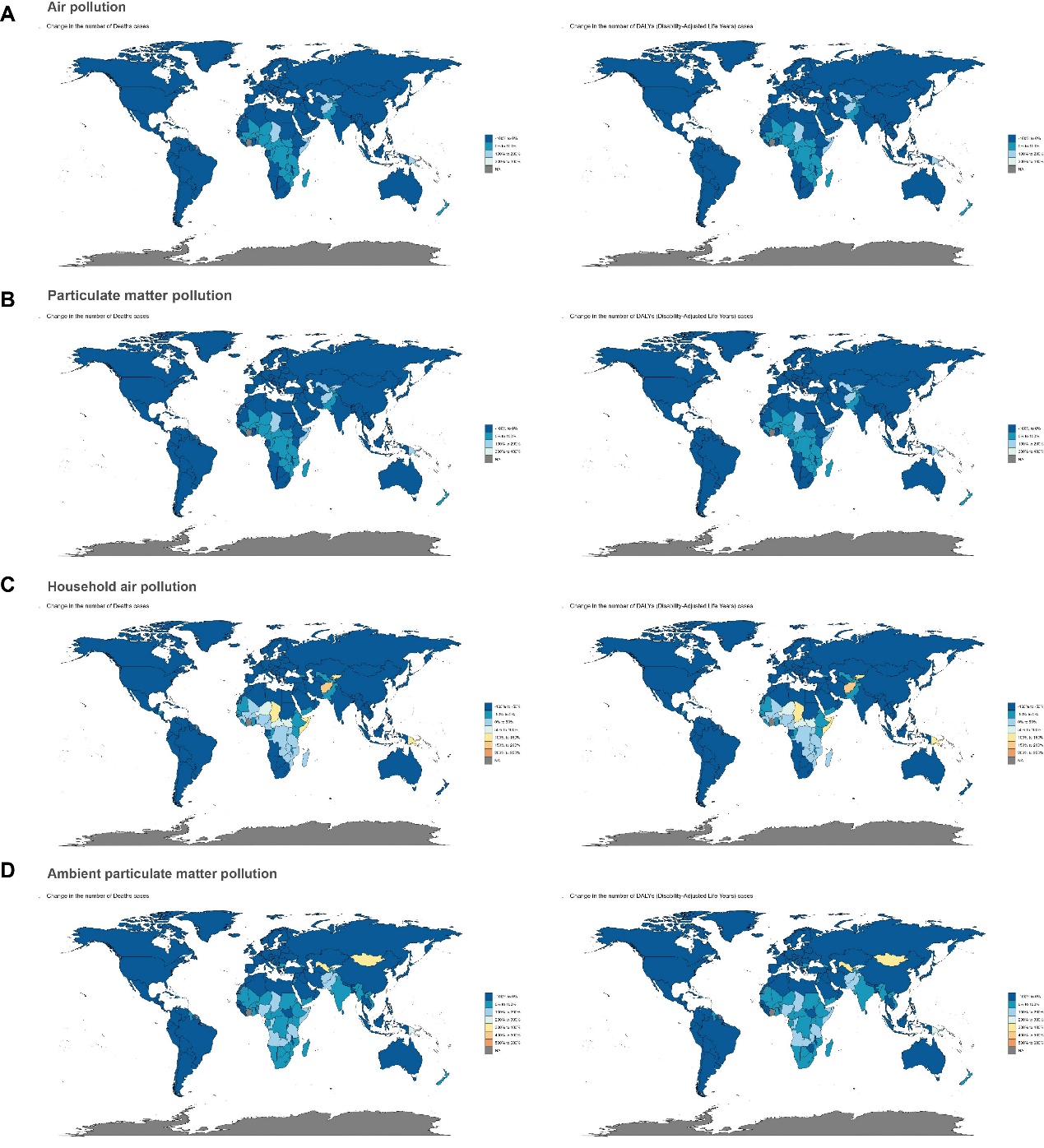
SUPPLEMENTARY FIGURE S4**

The relative change in the numbers of (A) air pollution, (B) particulate matter pollution, (C) household air pollution, and (D) ambient particulate matter pollution-related deaths and DALYs between 1990 and 2019.
