# Supplementary figures and images for: The global burden of neonatal sepsis attributable to air pollution from 1990 to 2021: findings from the global burden of disease study 2021
Source: Front Public Health. 2025 Sep 24;13:1644191. doi: 10.3389/fpubh.2025.1644191 (PMC12504511; doi:10.3389/fpubh.2025.1644191)

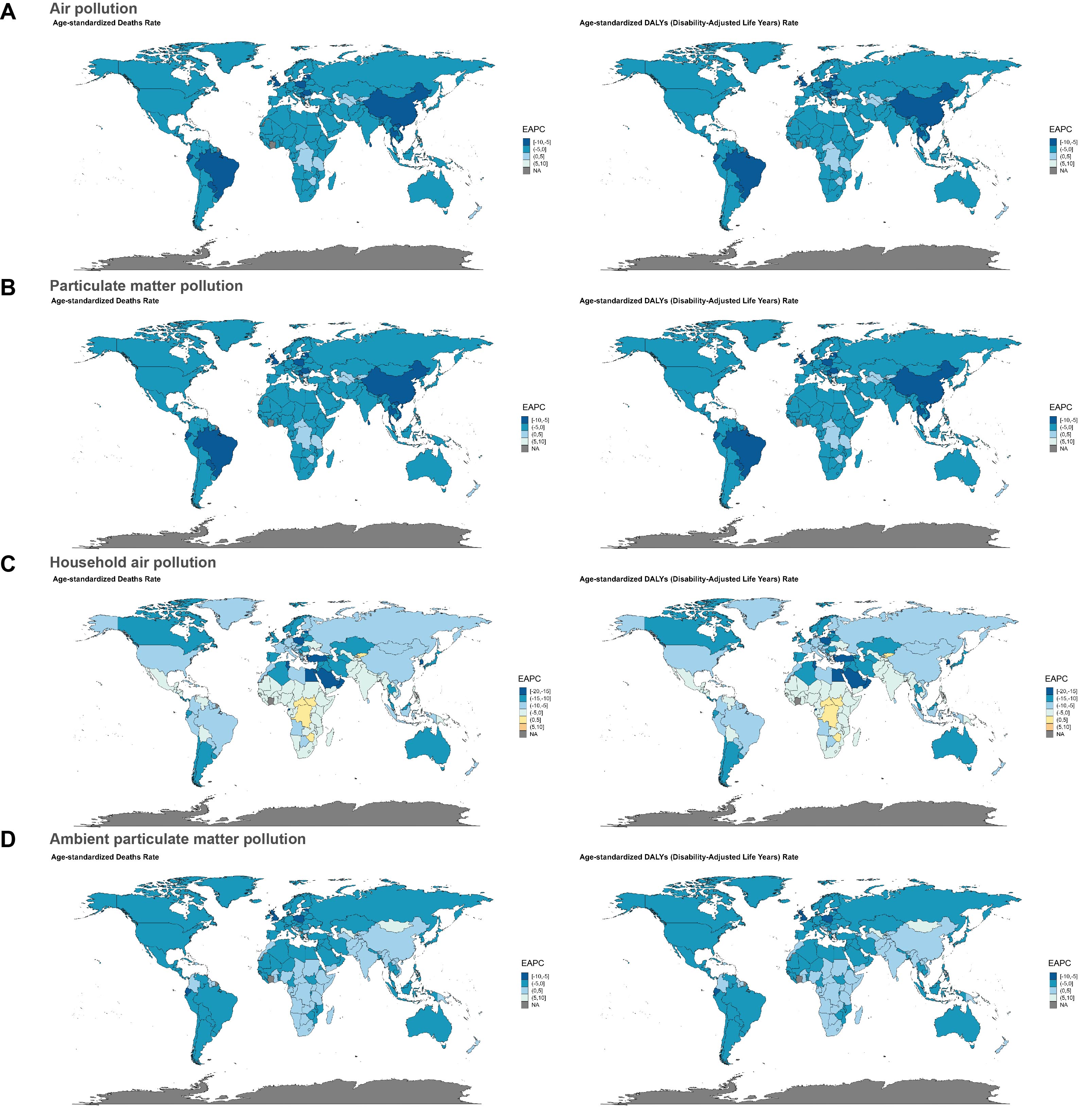

Supplement: Supplementary file 2 [file Presentation_1.zip › Supplementary Figure/Figure S1.jpg]

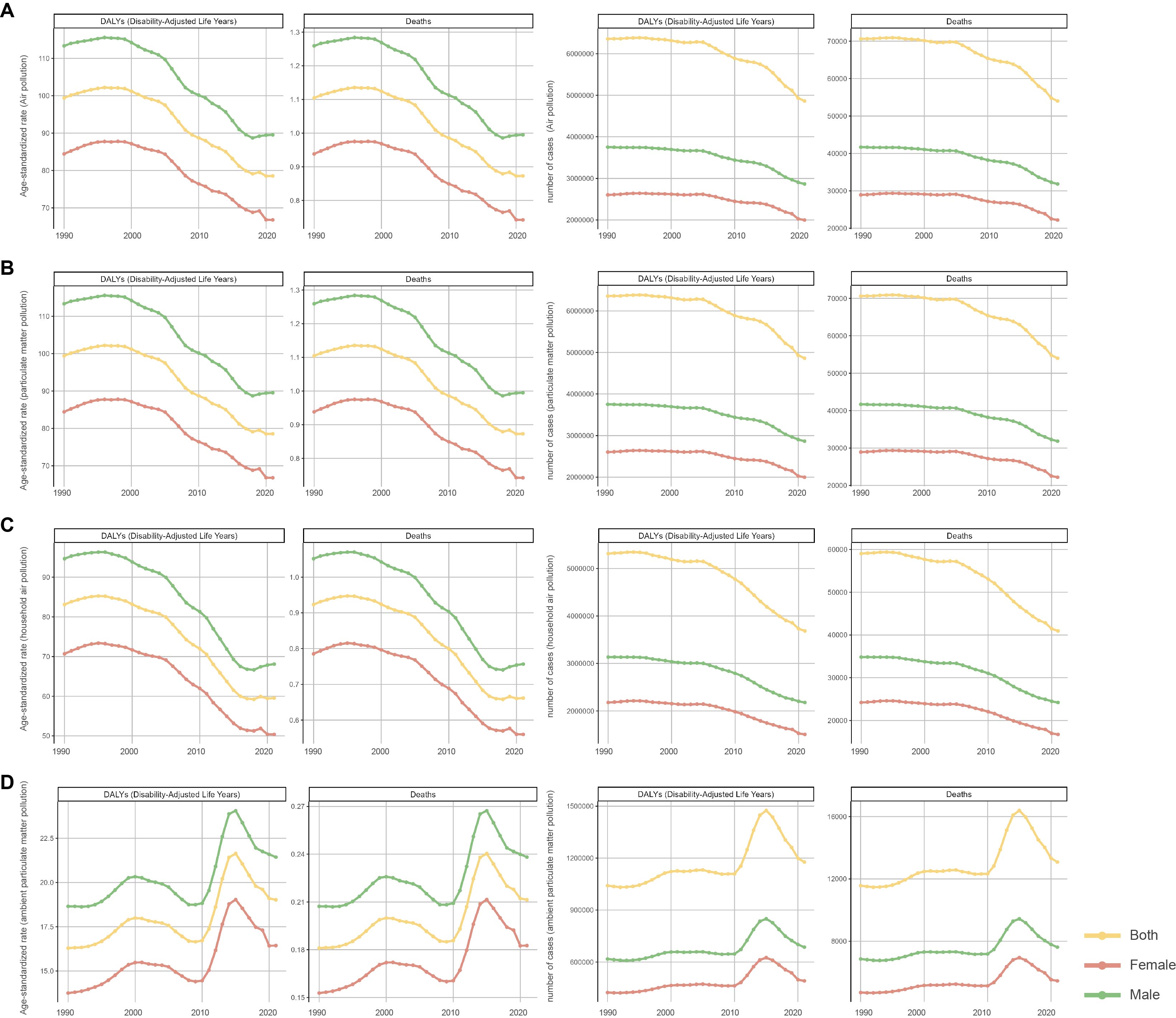

Supplement: Supplementary file 2 [file Presentation_1.zip › Supplementary Figure/Figure S2.jpg]

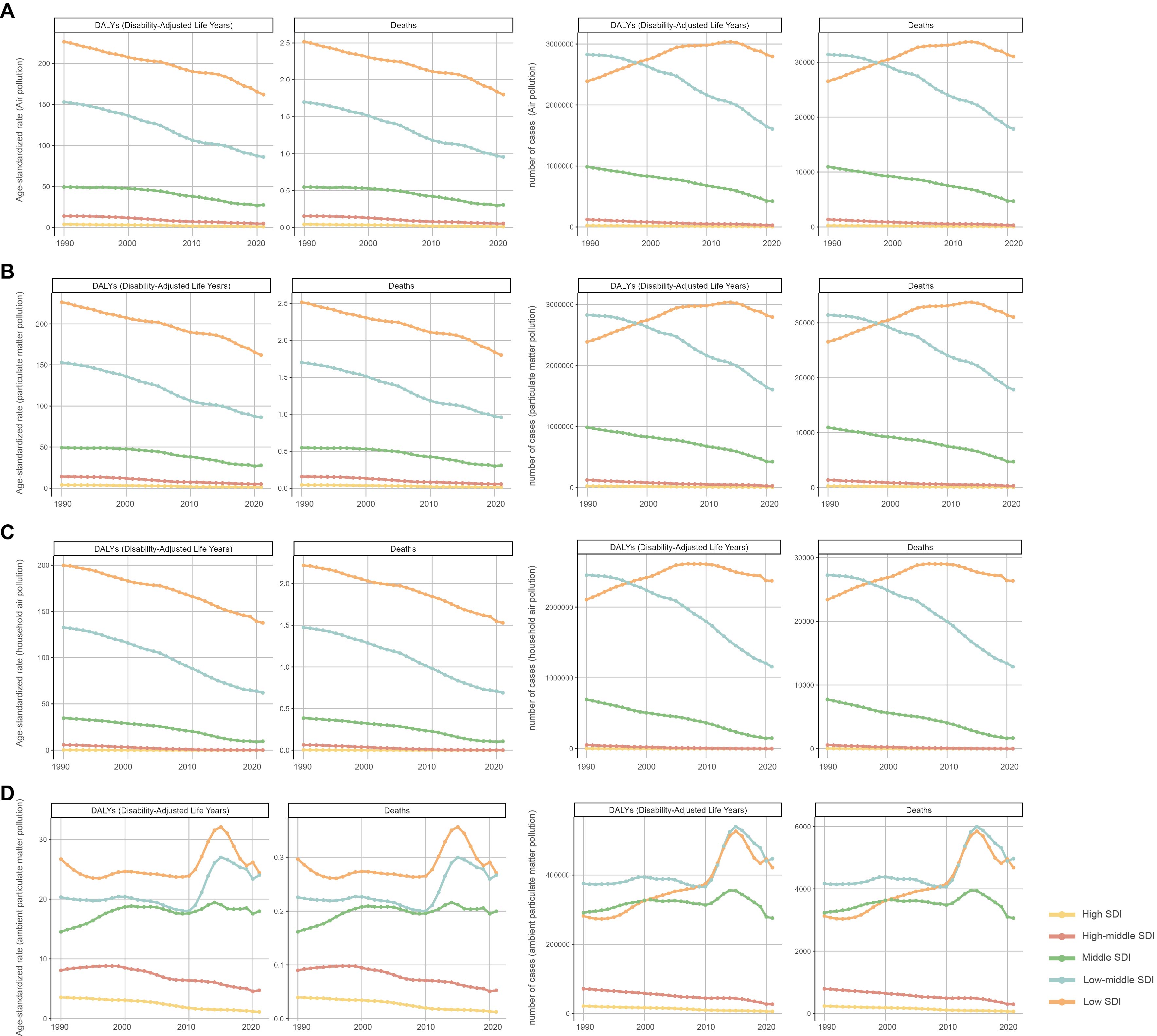

Supplement: Supplementary file 2 [file Presentation_1.zip › Supplementary Figure/Figure S3.jpg]

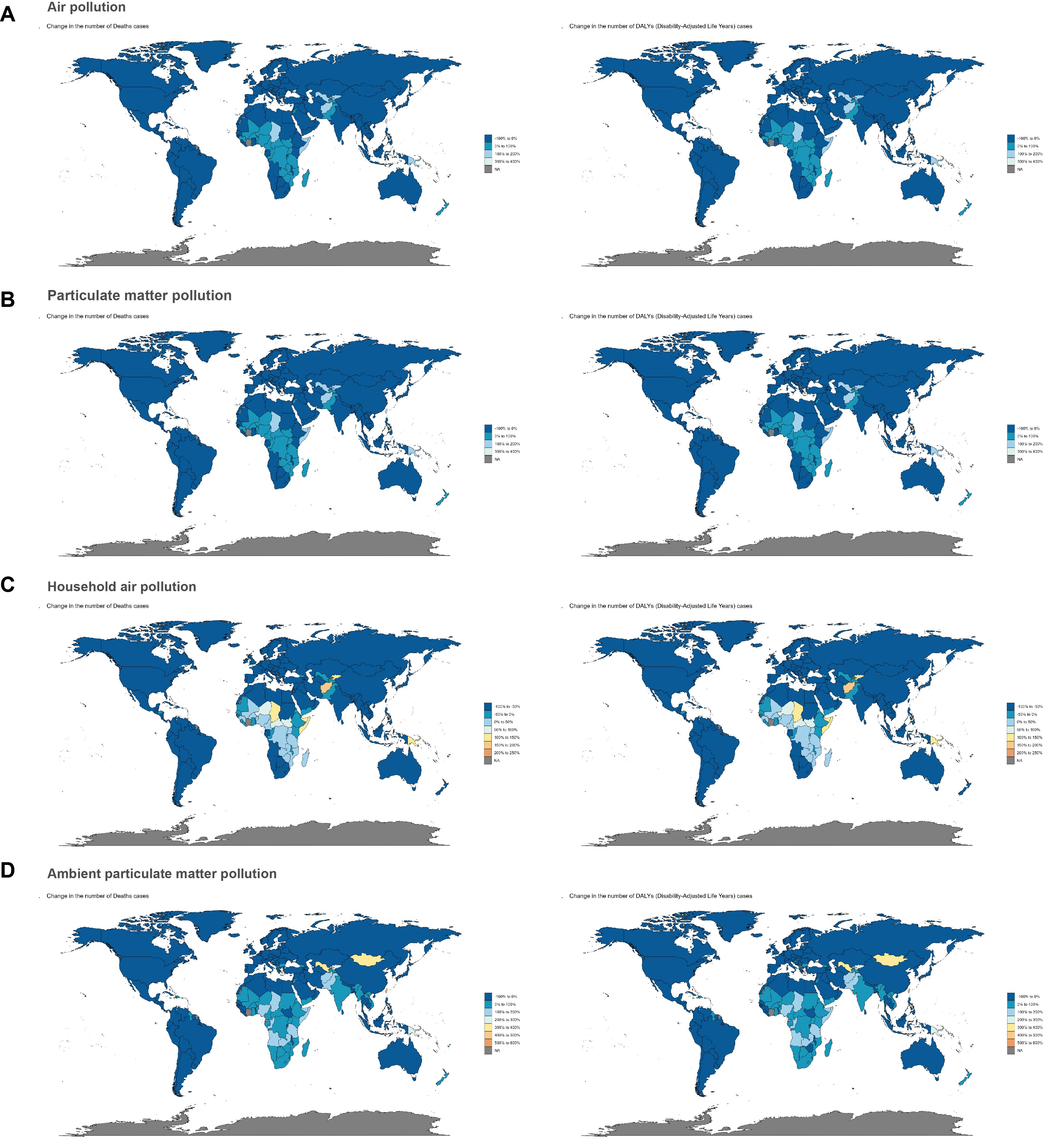

Supplement: Supplementary file 2 [file Presentation_1.zip › Supplementary Figure/Figure S4.jpg]

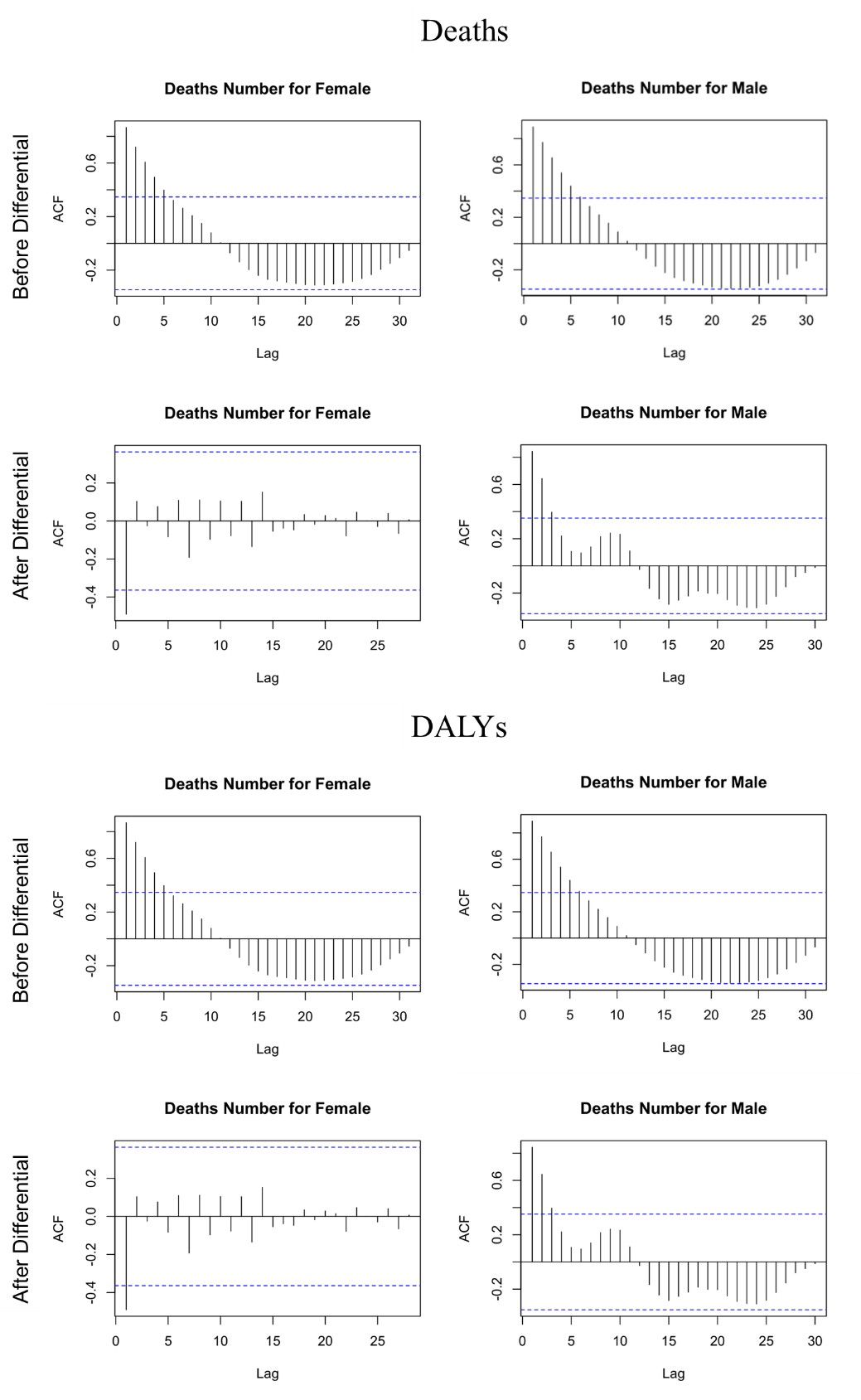

Supplement: Supplementary file 7 [file Image_5.jpeg]
